# Generic Platform for the Multiplexed Targeted Electrochemical Detection of Osteoporosis-Associated Single Nucleotide Polymorphisms Using Recombinase Polymerase Solid-Phase Primer Elongation and Ferrocene-Modified Nucleoside Triphosphates

**DOI:** 10.1021/acscentsci.3c00243

**Published:** 2023-07-19

**Authors:** Mayreli Ortiz, Miriam Jauset-Rubio, Olivia Trummer, Ines Foessl, David Kodr, Josep Lluís Acero, Mary Luz Botero, Phil Biggs, Daniel Lenartowicz, Katerina Trajanoska, Fernando Rivadeneira, Michal Hocek, Barbara Obermayer-Pietsch, Ciara K. O’Sullivan

**Affiliations:** 1 INTERFIBIO Research Group, Departament d’Enginyeria Química, Universitat Rovira i Virgili, 43007 Tarragona, Spain; 2 Division of Endocrinology and Diabetology, Department of Internal Medicine, Medical University of Graz, 8036 Graz, Austria; 3 Institute of Organic Chemistry and Biochemistry, Czech Academy of Sciences, Flemingovo namesti 2, CZ 16610 Prague 6, Czech Republic; 4 Labman Automation Ltd., Seamer Hill, Stokesley, North Yorkshire, TS9 5NQ U.K.; 5 Department of Internal Medicine, Erasmus MC, 40 3015 Rotterdam, The Netherlands; 6 Department of Organic Chemistry, Faculty of Science, Charles University, Hlavova 8, CZ-12843 Prague 2, Czech Republic; 7 Institució Catalana de Recerca i Estudis Avancats (ICREA), 08010 Barcelona, Spain

## Abstract

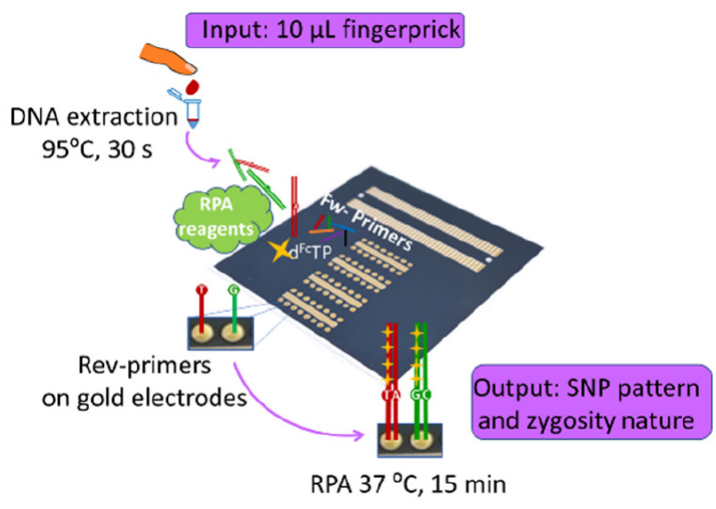

Osteoporosis is a multifactorial disease influenced by genetic and environmental factors, which contributes to an increased risk of bone fracture, but early diagnosis of this disease cannot be achieved using current techniques. We describe a generic platform for the targeted electrochemical genotyping of SNPs identified by genome-wide association studies to be associated with a genetic predisposition to osteoporosis. The platform exploits isothermal solid-phase primer elongation with ferrocene-labeled nucleoside triphosphates. Thiolated reverse primers designed for each SNP were immobilized on individual gold electrodes of an array. These primers are designed to hybridize to the SNP site at their 3′OH terminal, and primer elongation occurs only where there is 100% complementarity, facilitating the identification and heterozygosity of each SNP under interrogation. The platform was applied to real blood samples, which were thermally lysed and directly used without the need for DNA extraction or purification. The results were validated using Taqman SNP genotyping assays and Sanger sequencing. The assay is complete in just 15 min with a total cost of 0.3€ per electrode. The platform is completely generic and has immense potential for deployment at the point of need in an automated device for targeted SNP genotyping with the only required end-user intervention being sample addition.

## Abstract 2

Generic platform using isothermal solid-phase primer elongation for cost-effective, rapid electrochemical SNP genotyping from a fingerprick blood sample.

## Introduction

The possibility of reaching old age while sustaining a good quality of life is increasingly tangible, and the early detection of age-associated diseases that can eventually reduce autonomy is garnering interest. An example of such a disease is osteoporosis, the most common chronic skeletal metabolic disease, which is defined by a reduction in bone mass and microarchitectural deterioration in bone tissues, resulting in an increased risk of bone fracture.^1,2^ Bone mass is highly heritable, with heritability estimates of 0.5–0.85.^3,4^ Osteoporosis has a high worldwide incidence affecting ca. 200 million people,^5^ with the disease and its complications and fragility fractures incurring substantial global morbidity and mortality.^6^ Women are more susceptible to osteoporosis than men, but the prognosis for men to suffer hip or spine fractures is increasing.^7−9^ The disease is one of the most common among the elderly, affecting over 50% of women and 30% of men over the age of 50 in Europe,^10^ with the annual cost of osteoporotic fractures anticipated to increase to 106 billion euros by 2050.^11,12^

Measurement of bone mineral density (BMD) is widely used for the clinical diagnosis of osteoporosis,^13^ and dual-energy X-ray absorptiometry (DXA) is widely used for measuring BMD and is the most reliable clinical predictor of an osteoporotic fracture risk.^14,15^ As defined by the World Health Organization (WHO), a diagnosis of osteoporosis is reached when the BMD of an individual, measured by DXA, is 2.5 standard deviation or more below the average value for young, healthy individuals.^7^ However, the WHO also recognizes the diversification of diagnostic criteria for intermediate cases^7^ due to the multifactorial nature of the disease, which is conditioned by several environmental and genetic risk factors.^16^ Moreover, DXA devices have low sensitivity, detecting the loss of bone mineral density when a significant amount of bone is already lost.^17,18^

The discovery of genetic variation loci and the elucidation of their biological functions are critical to enable a further understanding of the etiology of osteoporosis and to thus facilitate the development of new approaches to screen for osteoporosis.^4,19^ In recent years, genome-wide association studies (GWAS) have been used to identify underlying genetic factors associated with various diseases.^9^ The aim of GWAS is the identification of robust (statistically significant and replicated) associations of single nucleotide polymorphisms (SNPs) with a specific phenotype.^9^ In the osteoporosis field, the Genetic Factors for Osteoporosis (GEFOS) consortium,^20−22^ the Genetic Markers for Osteoporosis (GENOMOS) consortium,^23^ and the UKBIOBANK have performed GWAS meta-analysis identifying hundreds of loci associated with BMD measured by DXA or estimated from heel ultrasounds.^16,24,25^ A panel of 15 fracture-associated loci with bone mineral density was identified and reported in 2018,^26^ and a Mendelian randomization approach of bone mineral density showed a causal effect on fracture risk. As demonstrated by clinical trials, there was no evidence of a causal effect for calcium or vitamin D supplementation.^27^ Out of the 15 identified fracture risk loci,^27^ we selected 4 SNPs that explained the largest proportion of fracture risk and mapped to genes implicated in bone biology which included WNT16 (rs2908007), RSPO3 (rs10457487), FAM210A (rs4635400), and SOST (rs2741856) as well as the well-established lactose intolerance marker (LCT(C/T-13910) polymorphism; rs4988235) to be implemented in an electrochemical platform for the detection of SNPs from a fingerpick blood sample, which could be employed at the point of care and used as a cost-effective screening tool, coupled with subsequent DXA analysis, for the early diagnosis of osteoporosis.

A single nucleotide polymorphism represents a variation in a single nucleotide that occurs at a specific position in the genome,^28^ and SNPs are the most significant contributors to genomic variation among individuals.^29^ A variety of techniques have been developed for SNP genotyping, including electrophoresis systems such as the cleaved amplified polymorphic sequence (CAPS), derived CAPS (dCAPS), and allele-specific (AS-PCR).^30^ High-throughput SNP-genotyping technologies have been developed and are employed, including the gene chip microarray and the competitive allele-specific PCR-based KAPS platform,^31^ and genotyping can also be achieved by sequencing.^32^ The first SNP array was developed by the Whiteside Institute together with Affymetrix and was designed to simultaneously detect almost 1500 SNPs,^33^ and there are now hundreds of customizable SNP chips available. However, the cost of these instruments is significant, and this upfront investment and subsequent maintenance costs cannot be met by a majority of laboratories. Furthermore, the slow turn-around times in obtaining results from centralized sequencing or microarray facilities is not compatible with rapid clinical decision making, and the possibility to detect SNPs at the point of care would facilitate prompt and informed treatment decisions. A cost-effective, robust, rapid, easy-to-use, and reliable technology for target-specific low- to middle-scale genotyping of genome-wide SNPs is desirable.

Isothermal amplification has been exploited for the detection of SNPs, including approaches exploiting loop-mediated isothermal amplification (LAMP) for the genotyping of blood and buccal cells^34^ as well as an approach using loop-primer endonuclease cleavage (LEC)-LAMP for the detection of SNPS in *Neisseria meningitidis*.^35^ Further examples include a competitive fluorophore-labeled probe hybridization assay following LAMP, which was applied to the detection of an SNP associated with the clinical response of personalized peptide vaccination in saliva samples.^36^ In another report, the use of loop-primer LAMP was used for the detection of SNPs in *Salmonella* enterica serovar Gallinarum biovars Pullorum and successfully applied in real sample testing of embryos, livers, and anal swabs from chickens in poultry farms.^37^ LAMP has also been used for the duplex detection of Factor V Leiden and Factor II G20210A variants in whole blood samples^38^ in a molecular beacon LAMP format for the detection of BRAF V600E,^39^ and further examples of the use of LAMP for the detection of SNPs has been comprehensively reviewed.^40^ Isothermal recombinase polymerase amplification (RPA) has also been exploited for the detection of SNPs, using allele-specific ligation to discriminate genetic variants related to cardiovascular diseases,^41^ which also has been used for the parallelized detection of the genotyping of four SNPs related to the treatment of tobacco addiction.^42^ A forward primer SNP detection approach using RPA has also been reported, where removing the reverse primer enhanced discrimination with single, double, and three scattered mismatches, and this approach was combined with lateral flow detection of an SNP that results in pyrethroid resistance in *Aedes aegypti* mosquitoes.^43^ Giant magnetoresistive (GMR) nanosensors using RPA have been applied to the genotyping of four SNPs (rs4633, rs4680, rs4818, and rs6269) along the catechol-*O*-methyltransferase gene (*COMT*) in salvia samples.^44^ Allelle-specific RPA has been used for the real-time fluorescence detection of the point mutation encoding hemoglobin S (HbS) in capillary blood, with the entire assay complete in <30 min at a cost of <$5.^45^

A wide range of diverse SNP detection methods have been developed, including single base extension using fluorescently labeled chain-terminating dideoxynucleotides (ddNTPs).^46−49^ Fluorescently labeled ddNTPs were also used in approaches using surface-tethered primers^50,51^ and in array-based primer extension (APEX)^52,53^ as well as alternate array-based technologies exploiting primer elongation^54^ and solid-phase polymerase chain reactions.^55^ As an alternative to fluorescence detection, SNP detection technologies based on electrochemical detection have been detailed, including an approach based on DNA-functionalized Cd-MOFs-74 as a cascade signal amplification probe under enzyme-free conditions for the detection of an SNP in the p53 tumor suppressor gene^56^ as well as the SNP detection based on silicon semiconductors^57^ and the use of electroactive rather than fluorescent labels for ddNTPs.^58^

Electrochemical devices are cost-effective, portable, and rapid, and we recently exploited these advantages to develop an approach for the electrochemical detection of an SNP directly from a fingerprick blood sample, without the need for DNA extraction or purification, which was completed in less than 20 min.^59^ This platform is based on isothermal solid-phase recombinase polymerase amplification, where four identical 5′-thiolated primers differing only at the terminal 3′ base are self-assembled onto individual gold electrodes of an array. The terminal base is designed to bind specifically to the base at the SNP site under interrogation, and following direct hybridization with genomic DNA, using carefully optimized conditions, only the primer with the complementary base is elongated. To facilitate electrochemical detection, ferrocene-labeled deoxynucleotides^60−62^ are incorporated during primer elongation, and square wave voltammetry is used to directly detect which primer has elongated and thus identify the allele present at the SNP site. In both differential pulse voltammetry (DPV) and square wave voltammetry (SWV), the faradaic to nonfaradaic current ratio is drastically increased and there is a very effective discrimination against the charging background current, achieving very low limits of detection compared to cyclic voltammetry.^63^ However, SWV has the added advantage that it provides currents up to 4 times higher and considerably faster responses than DPV. The scan rate of SWV (above 1 V/s) markedly decreases the analysis time, when compared to the commonly used DPV scan rate (around mV/s) where faster scan rates decrease the sensitivity and resolution of signals. This is of particular importance for multiplexed electrochemical detection.

In first demonstrations of the proof of concept, single SNPs were identified in DNA extracted and purified from sputum samples containing *Mycobacterium tuberculosis* to detect an SNP associated with rifampicin resistance^64^ and then in a fingerprick blood sample to identify an SNP linked with cardiomyopathy.^59^

In the present paper, we have extended our generic platform for the simultaneous detection of the five SNPs identified in the GEFOS/GENOMOS study.^15^ This system was first optimized using synthetic DNA and later tested using extracted genomic DNA. The effect of blood on the performance of the solid-phase primer elongation was evaluated, and the results were finally successfully validated with biobanked blood samples that had previously been genotyped using SNP-qPCR and further confirmed using Sanger sequencing. This platform can be facilely expanded to a plethora of further applications, and the number of SNPs can be vastly increased according to the end-user requirements, with the complete analysis from blood lysis to final read-out completed in less than 20 min.

## Results and Discussion

**Figure 1:**
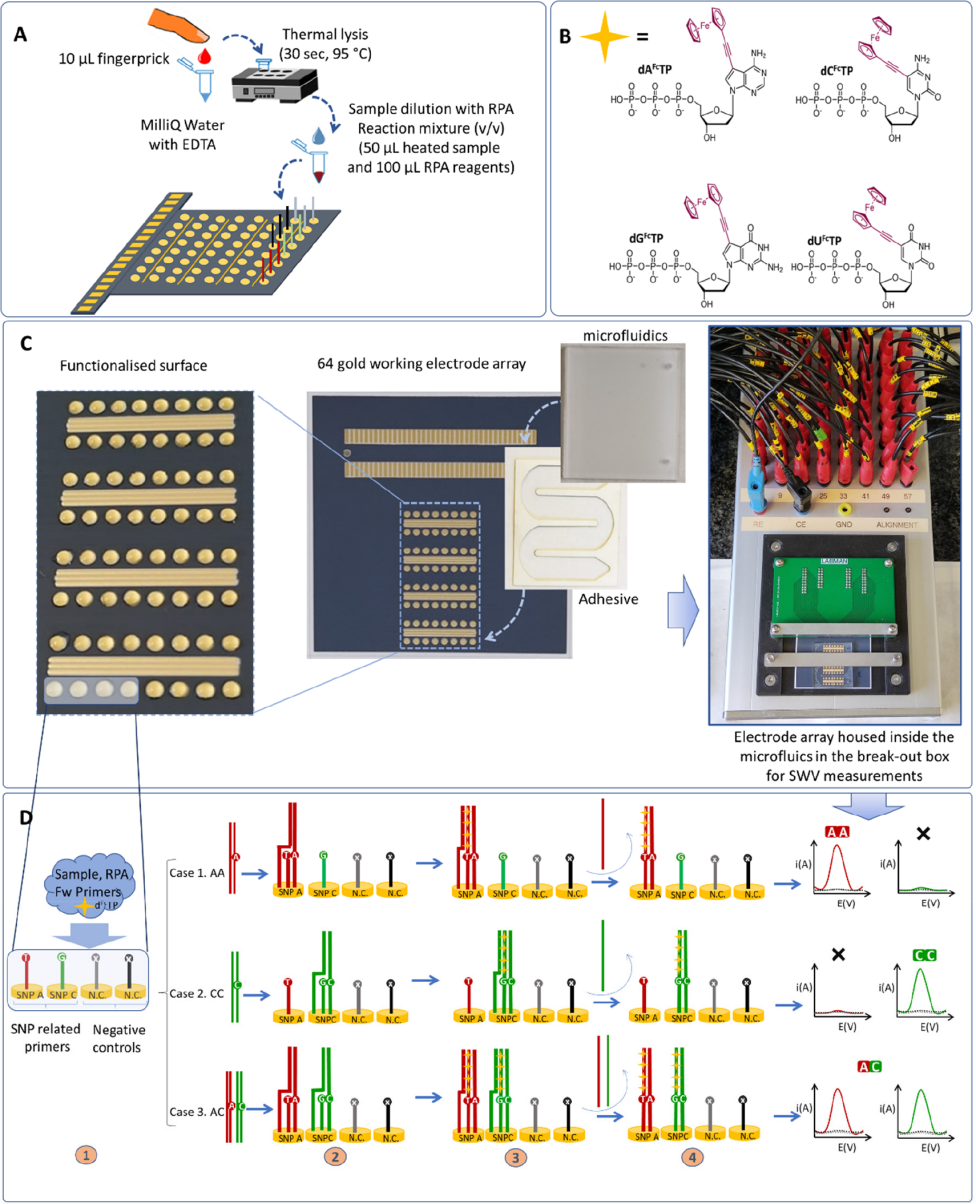
Schematic representation of the assay taking SNP 10 as an example for visualization of the different steps. (A) Rapid thermal lysis of the blood sample, which is combined with the RPA master mix containing the ferrocene-labeled dNTPs and added to the functionalized electrode array. (B) Schematic of the ferrocene-labeled dNTPs used in this work. (C) Actual picture of the setup: magnified image of the drops containing the thiolated primer and mercaptohexanol during surface functionalization, sequential assembly of the microfluidic cell, and break-out box fabricated for SWV measurements. (D) Visualization of the three different possible cases (cases 1 and 2 for homozygous samples and case 3 for heterozygous samples). The thiolated reverse primers are self-assembled on the gold electrode. Fully complementary base pairing occurs only at the primer with the terminal base complementary to the base at the SNP site on the genomic double-stranded DNA (dsDNA). Solid-phase primer elongation was performed using isothermal recombinase polymerase amplification and the electroactive ferrocene-labeled dNTPs (B) are enzymatically incorporated into the solid-phase amplification product. Following washing and denaturation, electrochemical detection using SWV is carried out.

To carry out the targeted SNP genotyping (Figure 1), a 10 μL blood sample is diluted 1:5, subjected to rapid thermal lysis,^59,65^ and directly used without the need for any further purification (Figure 1A). The lysed sample is then mixed with the RPA reagents (Figure 1A) and ferrocene-labeled dNTPs (Figure 1B) and injected into the channels of an PMMA microfluidic cell, integrated with a 64-electrode array previously functionalized with thiolated primers (Figure 1C). Solid-phase isothermal primer elongation is then allowed to proceed for a defined time (Figure 1D) at a fixed temperature. The use of ferrocene-labeled dNTPs (Figure 1B) results in a surface-tethered elongated primer containing multiple redox labels for direct electrochemical detection (Figure 1D), using a break-out box able to record the square wave voltammograms (SWV) from 64 working electrodes (Figure 1C). It should be highlighted that the number of electrodes in the array was defined to be 64 based on the number of channels available in the commercial Autolab multichannel potentiostat, and using a 64-electrode array and having an electrode for each allele, 2 negative electrodes for each SNP, the maximum number of SNPs that could be simultaneously used using the 64 electrode array would be 16. However, developmental work is ongoing with Labman Automation (U.K.) to produce a portable potentiostat with a higher number of channels, which would not only increase the number of SNPs that could be detected in one assay but also facilitate use at the point of need. Furthermore, the potentiostat under development has the potential to be battery operated using a 3000 mAh lithium ion battery, with an expected lifetime of >3h between charges.

For the present work, the SNPs identified by GEFOS/GENOMOS in previous GWAS analyses^26^ with the ultimate objective of constructing a genetic risk score (GRS) that can help to identify individuals at the extremes of the fracture risk distribution (i.e., and very high or very low risk of fracture) as well as an established Caucasian lactose intolerance marker were chosen to be detected.^66^ The subsets of SNPs are included in Table 1 and are referred to as SNP 10 (rs10457487), SNP 27 (rs2741856), SNP 29 (rs2908007), SNP 46 (rs4635400), and SNP 49 (rs4988235).

**Table 1:** Subset of Five SNPs Selected

SNP	CHR	POS	Closest Gene
rs10457487	6	127519234	RSPO3
rs2741856	17	41826839	SOS
rs2908007	7	120962164	WNT16
rs4635400	18	13719510	FAM210A
rs4988235	2	135851076	MCM6

Since humans are diploid organisms, two sets of homologous chromosomes have the same loci, one allele from the paternal and the other from the maternal parent. If both alleles are the same, then the organism is homozygous at that locus or SNP (e.g., AA or CC), and if they are different, then the organism is heterozygous at that locus (e.g., AC) (Figure S1). As schematically depicted in Figure 1D, the homo/heterozygous nature of the SNP under interrogation was elucidated via the use of two 5′-thiolated reverse primers, differing only in the base at the 3′-OH end which is complementary to the SNP to be detected. To further confirm the result, two additional 5′-thiolated reverse primers with the same sequence but carrying an unrelated base at the 3′-OH end were used as negative controls for each SNP. In the developed isothermal solid-phase approach, primer elongation is expected only with the primer(s) with the terminal base complementary to the allele present at the SNP sites. Following Figure 1D as an example, if the SNP10 is AA then elongation should be observed only from a primer terminating with T, and if the SNP10 is CC then only the primer terminating in G should be extended. If the SNP10 is heterozygous AC, then the elongation of both of the primers terminating in T and G should be observed. Negligible signal should be observed at electrodes modified with primers terminating in A and T, and these electrodes serve as negative controls.

### Evaluation of Primer and Target Sequences Designed for SNP Detection

The primers were primarily designed *in silico* with the aim of obtaining similar melting temperatures (*T*_m_) to facilitate equivalent amplification efficiency and avoid cross-reactivity between the primers. Two different software programs were used for the primer design: Primer Blast software to obtain primers with similar *T*_m_ values and GC content (Figure S2) and to check for cross-reactivity with nonspecific sequences found in the genome and Multiple Primer Analyzer software to screen for any potential self-dimer/primer-dimer formation (Figure S3). Finally, Table S1 summarizes the primer sets and DNA sequences used for SNP detection.

The specificity of the primers (Table S1) was then confirmed using PCR. Different PCR master mixes were prepared using the combined five forward primers with the individual reverse primer specific for each SNP to amplify the synthetic DNA sequences (Table S1). In all cases, a single band was observed via gel electrophoresis (Figure S4A), indicating that each synthetic DNA was amplified only with its specific reverse primer. When the target sequences were tested using the noncorresponding reverse primers, no band was observed in gel electrophoresis, confirming the specificity of the primers. Furthermore, no other bands were observed, demonstrating that no self-dimers or primer-dimers had formed during amplification (Figure S4A). This primer specificity was again achieved when these tests were performed using isothermal liquid-phase RPA, where only a single band was observed when each synthetic DNA was amplified with its specific reverse primer (Figure S4B).

### Proof of Concept of Multiplex Solid Phase Amplification

The specificity of primers was subsequently tested by using solid-phase RPA with colorimetric detection. An activated maleimide microtiter plate was modified with the 5′-thiolated reverse primers for the detection of five SNPs, including the β-globin housekeeping gene as a positive control. A reaction mixture containing the RPA reagents, the five forward primers, and the synthetic dsDNA targets was added to the wells, and primer elongation was achieved using biotinylated dNTPs. Following completion of the isothermal solid-phase primer elongation, streptavidin-modified HRP was added to bind to the incorporated biotinylated dNTPs, followed by addition of the substrate 3,3′,5,5′-tetramethylbenzidine. As can be seen in Figure S5, there was a clear differentiation in the signal obtained with the fully complementary primer as compared to the other primers. While this colorimetric assay demonstrated the viability of the approach and the specificity of the designed primers, it effectively comprises individual reactions for each of the SNPs, and the electrochemical platform (Figure S6) not only facilitates true simultaneous, parallelized detection of the SNPs from a single sample but also reduces the number of washing steps and avoids the use of sensitive reporter enzymes and substrates. Furthermore, the volume of the mixture of RPA reactants and the sample was decreased by ca. 27-fold, from approximately 4 mL to 150 μL (Figure S6B). The interelectrode reproducibility in the signal is excellent as evidenced by the signal obtained at each electrode of a 64-electrode array functionalized with 6-(ferrocenyl)hexanethiol (Figure S7).

The thiolated primers were self-assembled on the surface of individual gold electrodes of an array following the pattern outlined in Figure S8. Each set of primers is composed of two 5′-thiolated reverse primers complementary to the specific part of the genome containing the SNP under interrogation and differing only in the base at the 3′-OH end, with the terminal bases designed according to the SNP to be detected. Two additional negative controls of electrodes functionalized with 5′-thiolated reverse primers with terminal bases not specific to the SNP were also employed.

Additionally, two positive controls were implemented. The first positive control was an electrode functionalized with a 5′-thiolated reverse primer specific for the housekeeping β-globin gene, and a positive signal obtained at this electrode indicates correct cell lysis and solid-phase isothermal amplification. For the second positive control, an electrode was functionalized with the 5′-thiolated poly-A sequence that should simply hybridize with an Fc-poly T, which is added together with the forward primers. A positive signal obtained at this electrode indicates correct functioning of the electrodes, connectors, and fluidics.

### Optimization of the Simultaneous Detection of Five SNPs Using the RPA Reaction

In the assay reported here, the genomic DNA interacts with 20 surface-tethered primers, 4 primers for each of the 5 SNPs, requiring a careful optimization of the reaction time, temperature, and concentrations of the solution-phase forward primers. For optimization of the assay, several electrode arrays were functionalized and housed inside the microfluidic cell (Figure 1C and Figure S6B), with the RPA carried out with synthetic sequences using different parameters and the electrochemical signal measured in the setup shown in Figure 1C and Figure S6D.

**Figure 2:**
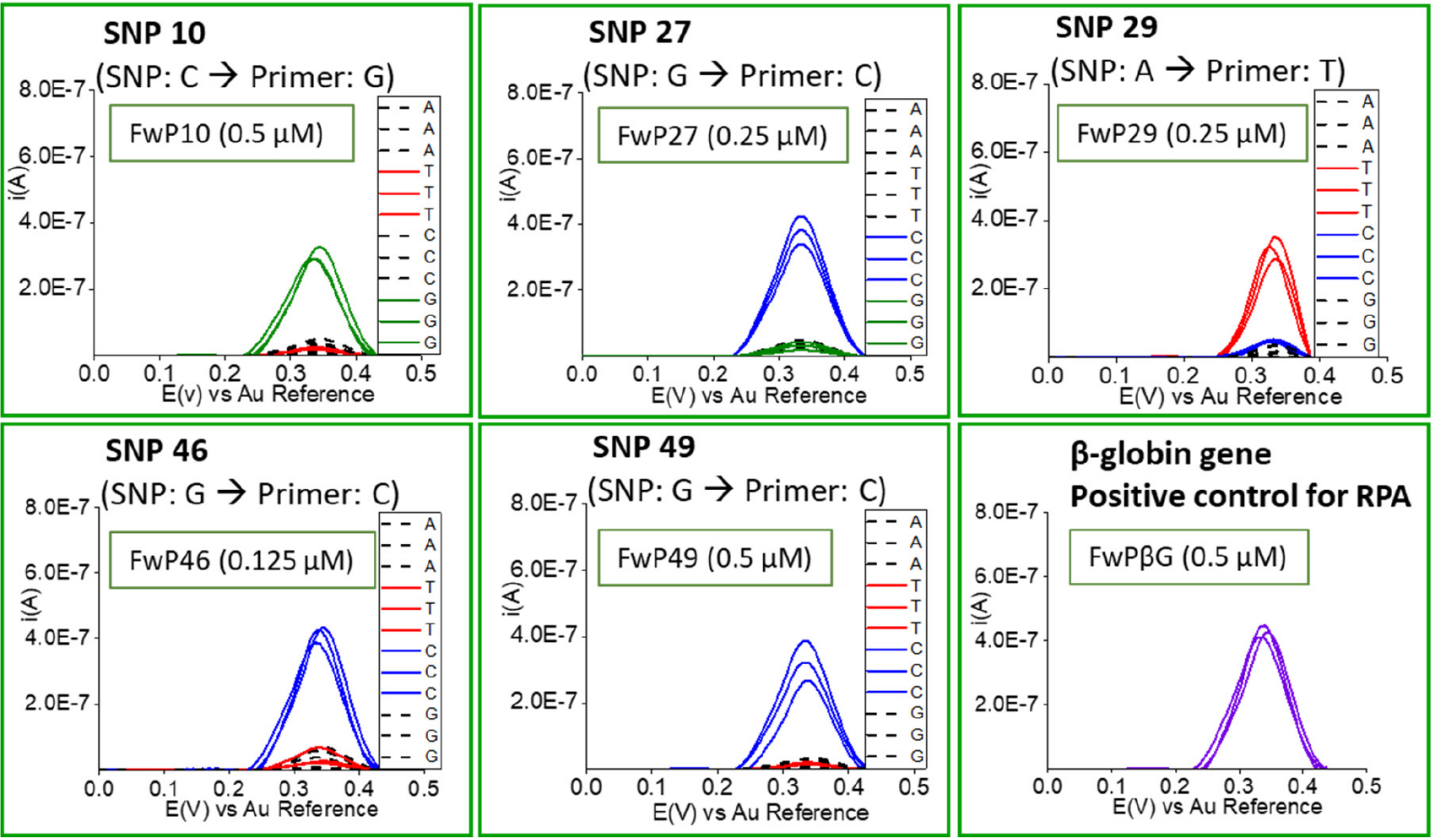
Square wave voltammograms obtained after optimization of the concentration of the five forward primers (FwP) for the simultaneous detection of the five SNPs related to osteoporosis using isothermal solid-phase primer elongation and dN^Fc^TPs. The β-globin gene was also included as a positive control of the RPA reaction.

The optimum duration of the solid-phase isothermal primer elongation was observed to be 15 min, with an optimum applied temperature of 37 °C. The forward primer concentration played an important role in the kinetics of the reaction, and the optimum concentrations were determined (Figure S8). As can be seen in test 1 (Figure S8), the kinetics of the solid-phase primer elongation reaction are not equal for every SNP. Using a 0.5 μM forward primer concentration, the positive primers for SNP 10 and 49 are fully discriminated from negative primers but not for SNP 27, SNP 29, and SNP 46. The differentiability was improved for SNP 27 and SNP 29 by decreasing the concentration of their forward primers to 0.25 μM (test 2) while maintaining the concentration of the forward primers of SNP 10 and 49 at 0.5 μM, and the concentration of the forward primer for SNP 46 had to be further decreased to 0.125 μM (test 3). The optimum concentration of forward primers (Figure 2) was thus 0.5 μM for SNP 10 and SNP 49, 0.25 μM for SNP 27 and SNP 49, and 0.125 μM for SNP 46, and these concentrations were used in all further experiments. In normal PCR/solution-phase RPA, either the salt concentration or primer concentration can be optimized to achieve equal amplification efficiency. Indeed, we have previously observed that with a range of diverse targets that there are some sequences that are amplified using RPA much more rapidly and efficiently than others, but we have not been able to find an explanation for this as there appears to be no correlation between amplification rates and the GC content of the melting temperature of target/primers.^67^ As with multiplexed amplification, a common salt concentration has to be used, and the concentrations of the forward primers were used to achieve equal amplification efficiency.

### Validation of Multiplexed Electrochemical Detection of SNPs Using Isothermal Solid-Phase Primer Elongation with Biobanked Samples

**Figure 3:**
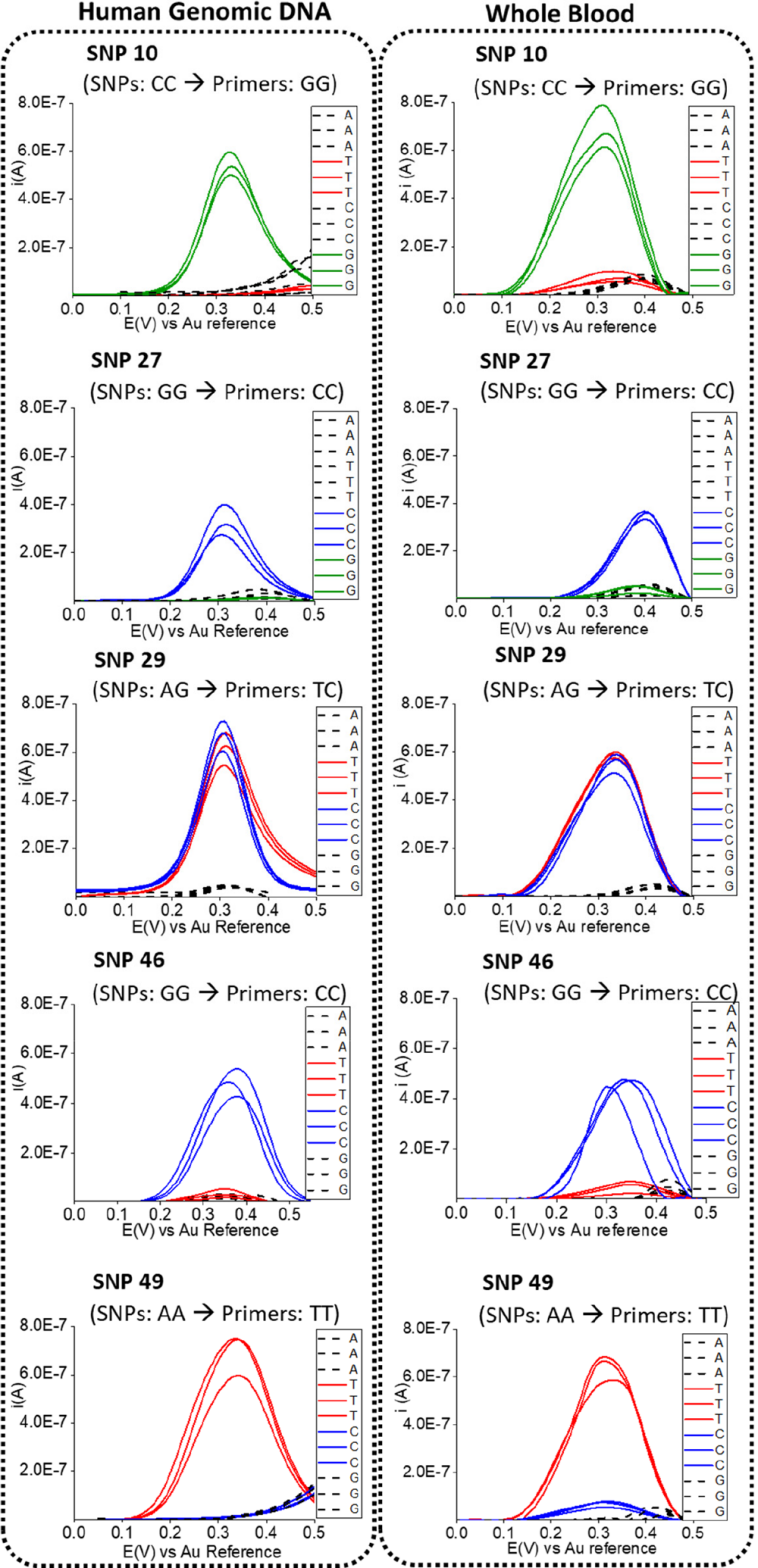
Square wave voltammograms recorded in 0.1 M Sr(NO_3_)_2_ + 0.1 M glycine pH 3 for the electrochemical detection of the five SNPs in extracted and purified genomic DNA from a human whole blood sample and in the same blood sample to evaluate the matrix effect. The SNP-related primers are highlighted in different colors (red for primers ending in T for SNPs A, blue for primers ending in C for SNPs G, and green for primers ending in G for SNPs C) while the negative primers are represented by black and discontinuous traces).

The effect of the blood matrix was primary evaluated. The genomic DNA was extracted and purified using a NucleoSpin blood kit to eliminate the complex blood matrix from a panel of five representative whole blood samples containing different SNPs. The signal obtained following solid-phase primer elongation was compared to that obtained with the original blood sample, which had been thermally lysed and used without purification. The results obtained fully agreed with each other in terms of SNP identification, and the intensity of the signals was very similar (Figure 3 is included as an example of one of the samples and Figure S9 is included for the other four), highlighting that there is negligible matrix effect of the whole blood on the solid-phase isothermal primer elongation reaction and electrochemical detection of the surface-tethered amplicon.

Having demonstrated that the whole blood matrix has a negligible effect on the signal and in no way affects the identification of the SNP, the remaining whole blood samples were processed using rapid thermal lysis, followed by isothermal solid-phase primer elongation, and the elongated primers were detected electrochemically (Figure S10).

### Corroboration of the Developed Approach with Two Reference Methods (TaqMan Fluorogenic 5-Exonuclease Assay and Sanger Sequencing)

The electrochemical platform for the detection of SNPs was primarily validated using the SNP-specific TaqMan fluorogenic 5-exonuclease assay, an assay routinely used in clinical laboratories for the detection of SNPs, and was the assay employed at the Medical University of Graz. This assay requires prior cell lysis, DNA extraction, and purification, and individual assays need to be carried out for each SNP to be studied. The assay mix contains the TaqMan probes specific for each SNP, which were labeled with 2′-chloro-7′-phenyl-1,4-dichloro-6-carboxy-fluorescein (VIC) for allele 1 and 6-carboxyfluorescein (FAM) for allele 2 and the allelic discrimination plots used for genotyping (Table S2, Figure S11).

**Figure 4:**
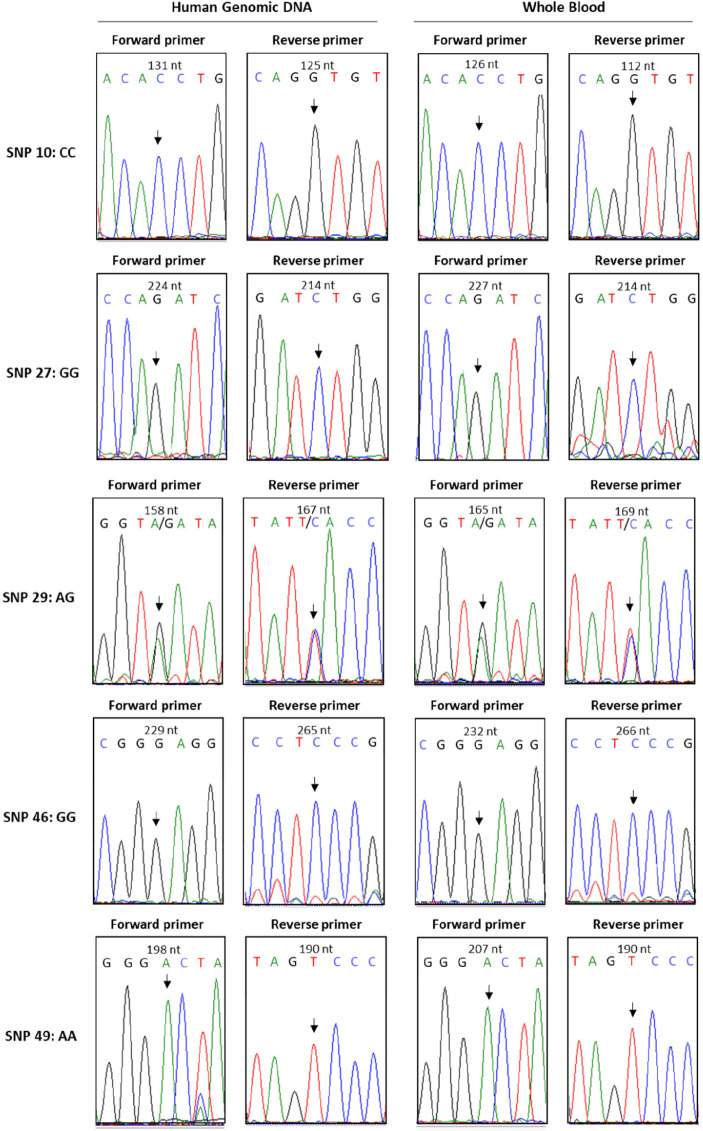
Chromatograms of Sanger sequencing results of the sample shown in Figure 3. The SNP is highlighted with an arrow. The presence of a single peak in the chromatogram represents a homozygous SNP (e.g., SNP 10 forward primer: CC), while the observation of two peaks indicates a heterozygous SNP (e.g., SNP 29 forward primer: AG).

To further confirm the identification of the SNP under interrogation, the whole blood samples were sequenced using automated Sanger sequencing. In order to demonstrate that the DNA obtained following thermal lysis matched the DNA extracted and purified, five samples were sequenced, and as can be seen in Figure 4, a perfect correlation in the sequencing results was obtained, which were in agreement with the results obtained using the developed electrochemical platform. In addition, using these five samples, both strands of the DNA were sequenced using forward and reverse primers to validate the SNP detected (Figure 4). The presence of a single peak in the chromatogram represents a homozygous SNP (e.g., SNP 10 forward primer: CC), while the two peaks indicate a heterozygous SNP (e.g., SNP 29 forward primer: AG). Finally, the SNP detected from the forward strand was corroborated by the complementary base identified from the reverse strand (e.g., SNP 10 forward primer: CC; SNP 10 reverse primer: GG).

Bioedit software was used to check the resulting sequences and the chromatograms (Figure S12), while Blastn software was used to align the obtained sequences with the reference sequence from the synthetic DNA, demonstrating greater than the 90% similarity between both sequences (Figure S13).

Table 2 summarizes the results obtained (shown visually in Figures 3, 4, S9, S10, S11, S12, and S13) with automated Sanger sequencing, the TaqMan fluorogenic 5-exonuclease assay, and electrochemical detection. As can be seen, there is a 100% correlation among the results obtained using the three techniques, thus validating the results obtained with the electrochemical platform developed for SNP genotyping.

**Table 2:** Correlation between the SNP Detected by the Genomic Sensor (EC) Using Whole Blood Samples and Reference Methods: TaqMan Fluorogenic 5-Exonuclease Assay (Taq-man) and Sanger Sequencing (S.Seq.)

	SNP														
	rs10457487			rs2741856			rs2908007			rs4635400			rs4988235		
Sample ID↓	EC	Taq-man	S.Seq	EC	Taq-man	S.Seq	EC	Taq-man	S.Seq	EC	Taq-man	S.Seq	EC	Taq-man	S.Seq
1	CC	CC	CC	GG	GG	GG	AG	AG	AG	GG	GG	GG	AA	AA	AA
2	AC	AC	AC	GC	GC	GC	AG	AG	AG	AA	AA	AA	AG	AG	AG
3	AC	AC	AC	GG	GG	GG	AA	AA	AA	AA	AA	AA	AG	AG	AG
4	CC	CC	CC	GC	GC	GC	AG	AG	AG	AG	AG	AG	AA	AA	AA
5	CC	CC	CC	GG	GG	GG	AA	AA	AA	AG	AG	AG	AG	AG	AG
6	AA	AA	AA	GG	GG	GG	AG	AG	AG	GG	GG	GG	AG	AG	AG
7	CC	CC	CC	GG	GG	GG	AG	AG	AG	GG	GG	GG	AA	AA	AA
8	CC	CC	CC	GG	GG	GG	AA	AA	AA	AA	AA	AA	AG	AG	AG
9	AA	AA	AA	GG	GG	GG	GG	GG	GG	AA	AA	AA	AA	AA	AA
10	AC	AC	AC	GG	GG	GG	AG	AG	AG	AG	AG	AG	AG	AG	AG
11	CC	CC	CC	GG	GG	GG	AA	AA	AA	AA	AA	AA	AG	AG	AG
12	AC	AC	AC	GG	GG	GG	AA	AA	AA	AG	AG	AG	AG	AG	AG
13	AA	AA	AA	GG	GG	GG	AG	AG	AG	AA	AA	AA	AG	AG	AG
14	AC	AC	AC	GG	GG	GG	AA	AA	AA	AG	AG	AG	GG	GG	GG
15	AC	AC	AC	CC	CC	CC	AA	AA	AA	GG	GG	GG	AG	AG	AG

Osteoporosis is highly polygenic and heritable, with heritability ranging from 50 to 80%; however, only a useful combination of genetic variants will be able to demonstrate clinical efficiency in the prediction of patients at risk.^68^ The four SNPs selected from the GWAS studies have been shown to be associated with up to a 10% increase in fracture risk and have been mapped to genes clustering in pathways known to be relevant to bone biology.^69^ For instance, SOST and WNT16 are two of the key regulators of bone remodeling,^70,71^ have been robustly associated with low bone mineral density, and have led to 10 and 6% increases in fracture risk, respectively.^69,72^ RSPO3 has been shown to regulate vertebral trabecular bone mass and bone strength in mice and fracture risk in humans^73^ and was associated with up to a 5% increase in fracture risk. FAM210A as a novel bone pathway has been shown to influence the structure and strength of both muscle and bone^74^ and has been shown to be associated with up to a 5% increase in fracture risk. Furthermore, the well-established lactose intolerance marker (LCT(C/T-13910)) is of immediate diagnostic as well as therapeutic value for the potential users, as clinical congruence with lactose malabsorption is very high.^75^ Therefore, peroral supplementation with lactose-free calcium in individuals with lactose intolerance can be immediately recommended to avoid gastrointestinal malabsorption by common milk products containing lactose in patients at risk for osteoporosis.

Finally, the use of genotyped SNPs is maximized when constructing a so-called polygenic risk score.^76^ Although the sum of trait-associated alleles was not weighted for their effective sizes, a simple quantitative index of genetic predisposition to bone-related risks was established. Table 3 summarizes the SNP detected with the risk alleles highlighted in bold and underlined. The higher number of present risk alleles can be considered to reflect a higher genetic predisposition. The polygenic risk score assists in the identification of individuals with high genetic susceptibility to developing osteoporosis (in this case, 2 of the 15 samples are shown in Table 3 and Figure S14).

**Table 3:** Simple Quantitative Index of the Genetic Predisposition to Bone-Related Risks (SQI)^a^

		SNP→						
			SNP10	SNP27	SNP29	SNP46	SNP49	
Sample ID↓	Sex (f = female/m = male)	Age (years)	rs10457487 R(A)=C	rs2741856 R(A)=G	rs2908007 R(A)=A	rs4635400 R(A)=A	rs4988235 R(A)=G	SQI
1	f	62	CC	GG	AG	GG	AA	5
2	m	65	AC	GC	AG	AA	AG	6
3	f	44	AC	GG	AA	AA	AG	8
4	f	61	CC	GC	AG	AG	AA	5
5	f	67	CC	GG	AA	AG	AG	8
6	m	67	AA	GG	AG	GG	AG	4
7	f	62	CC	GG	AG	GG	AA	5
8	f	58	CC	GG	AA	AA	AG	9
9	|f	56	AA	GG	GG	AA	AA	4
10	f	62	AC	GG	AG	AG	AG	6
11	m	72	CC	GG	AA	AA	AG	9
12	f	62	AC	GG	AA	AG	AG	7
13	f	60	AA	GG	AG	AA	AG	6
14	f	57	AC	GG	AA	AG	GG	8
15	f	63	AC	CC	AA	GG	AG	4

a Risk alleles are underlined. The SQI was not weighted for its allelic effect sizes. The higher the number of risk alleles, the higher the theoretical genetic predisposition.

## Conclusions

A generic platform for the simultaneous electrochemical detection of multiple single nucleotide polymorphisms (SNPs) via solid-phase isothermal primer elongation has been successfully implemented. Five SNPs associated with an increased risk of developing osteoporosis and a risk of fracture were detected in 15 blood samples, with the results validated using both a TaqMan SNP-specific fluorogenic 5-exonuclease assay and Sanger sequencing. It has been demonstrated that the blood matrix has no effect on the assay performance or the electrochemical detection. The generic platform is robust and can be used directly with thermally lysed blood samples with no need for DNA extraction or purification, with the entire assay from the addition of the lysed sample to the readout of the results being complete in just 15 min, with the cost per SNP, on a laboratory scale, including the cost of the electrode array, microfluidics, and all reagents being ca. 0.3€. The heterozygous versus homozygous nature of the SNP is robustly determined, with results further confirmed by negative and positive controls. While in the work reported here, the assay was carried out in triplicate for each of the immobilized primers, the use of the negative controls was demonstrated to be robust enough to eliminate the need for triplicate readings, and thus 4 electrodes per SNP is adequate, with 2 positive controls per array and using the 64 electrode array 15 SNPs could be simultaneously detected. The reported platform is completely generic in nature and can be used for any application requiring SNP genotyping. The number of SNPs can be facilely expanded to a higher number of SNPs simply by increasing the number of electrodes per array, which can be defined by the end-user requirements, with no impact on cost/SNP or assay time, and is currently limited by the number of channels available with commercial potentiostats. A potentially battery-operatable portable potentiostat, measuring L 146 × W 85 × H 88 mm^3^, capable of simultaneous detection of all 64 electrodes in less than 7 s has been developed by Labman Automation, which has the ability to scale up to 128, 256 electrodes, etc. and will be employed in future work for the implementation of the assay at the point of need.

## Supporting Information Available

Materials and methods used such as the reagents, DNA sequences, and detailed procedures in the paper; description of primer design and their evaluation for both the biosensor approach and Sanger sequencing; details of the break-out box, microfluidic cell, and screen-printed electrode array designs and fabrication followed by the procedures of electrode functionalization; protocols followed for the solid-phase RPA reaction and SWV measurements (both optimization procedures and final reaction) and whole blood sample treatment in the validation step; raw data of all SWV measurements (in triplicate) for optimization studies and blood samples as well as the experimental data of the TaqMan fluorogenic 5-exonuclease assay and Sanger sequencing for the biosensor validation (PDF)

## References

[ref1] Zhu W.; Xu C.; Zhang J.-G.; He H.; Wu K.-H.; Zhang L.; Zeng Y.; Zhou Y.; Su K.-J.; Deng H.-W. Gene-Based GWAS Analysis for Consecutive Studies of GEFOS. Osteoporos Int 2018, 29 (12), 2645–2658. 10.1007/s00198-018-4654-y. 30306226 PMC6279247

[ref2] Richards J. B.; Kavvoura F. K.; Rivadeneira F.; Styrkársdóttir U.; Estrada K.; Halldórsson B. V; Hsu Y.-H.; Zillikens M. C.; Wilson S. G.; Mullin B. H.; et al. Collaborative Meta-Analysis: Associations of 150 Candidate Genes with Osteoporosis and Osteoporotic Fracture. Ann. Intern. Med. 2009, 151 (8), 528–537. 10.7326/0003-4819-151-8-200910200-00006. 19841454 PMC2842981

[ref3] Ralston S. H.; Uitterlinden A. G. Genetics of Osteoporosis. Endocr Rev 2010, 31 (5), 629–662. 10.1210/er.2009-0044. 20431112

[ref4] Mei B.; Wang Y.; Ye W.; Huang H.; Zhou Q.; Chen Y.; Niu Y.; Zhang M.; Huang Q. LncRNA ZBTB40-IT1 Modulated by Osteoporosis GWAS Risk SNPs Suppresses Osteogenesis. Hum. Genet. 2019, 138 (2), 151–166. 10.1007/s00439-019-01969-y. 30661131

[ref5] Zhu X.; Bai W.; Zheng H. Twelve Years of GWAS Discoveries for Osteoporosis and Related Traits: Advances, Challenges and Applications. Bone Res 2021, 9 (1), 23 10.1038/s41413-021-00143-3. 33927194 PMC8085014

[ref6] Cummings S. R.; Melton L. J. Epidemiology and Outcomes of Osteoporotic Fractures. Lancet 2002, 359 (9319), 1761–1767. 10.1016/S0140-6736(02)08657-9. 12049882

[ref7] World Health Organization. Assessment of Osteoporosis at the Primary Health Care Level; Summary report of a WHO scientific group; WHO, Geneva, 2007.

[ref8] Tabatabaei-Malazy O.; Salari P.; Khashayar P.; Larijani B. New Horizons in Treatment of Osteoporosis. Daru 2017, 25 (1), 2 10.1186/s40199-017-0167-z. 28173850 PMC5297185

[ref9] Schuit S. C. E.; van der Klift M.; Weel A. E. A. M.; de Laet C. E. D. H.; Burger H.; Seeman E.; Hofman A.; Uitterlinden A. G.; van Leeuwen J. P. T. M.; Pols H. A. P. Fracture Incidence and Association with Bone Mineral Density in Elderly Men and Women: The Rotterdam Study. Bone 2004, 34 (1), 195–202. 10.1016/j.bone.2003.10.001. 14751578

[ref10] Johnell O.; Kanis J. Epidemiology of Osteoporotic Fractures. Osteoporos Int 2005, 16, S3–S7. 10.1007/s00198-004-1702-6. 15365697

[ref11] Wesselius A.; Bours M. J. L.; Henriksen Z.; Syberg S.; Petersen S.; Schwarz P.; Jørgensen N. R.; van Helden S.; Dagnelie P. C. Association of P2Y(2) Receptor SNPs with Bone Mineral Density and Osteoporosis Risk in a Cohort of Dutch Fracture Patients. Purinergic Signal 2013, 9 (1), 41–49. 10.1007/s11302-012-9326-3. 22773251 PMC3568433

[ref12] https://www.osteoporosis.foundation.

[ref13] Kanis J. A.; Borgstrom F.; De Laet C.; Johansson H.; Johnell O.; Jonsson B.; Oden A.; Zethraeus N.; Pfleger B.; Khaltaev N. Assessment of Fracture Risk. Osteoporos Int 2005, 16 (6), 581–589. 10.1007/s00198-004-1780-5. 15616758

[ref14] Zethraeus N.; Borgström F.; Ström O.; Kanis J. A.; Jönsson B. Cost-Effectiveness of the Treatment and Prevention of Osteoporosis-a Review of the Literature and a Reference Model. Osteoporos Int 2007, 18 (1), 9–23. 10.1007/s00198-006-0257-0. 17093892

[ref15] Karasik D.; Dupuis J.; Cho K.; Cupples L. A.; Zhou Y.; Kiel D. P.; Demissie S. Refined QTLs of Osteoporosis-Related Traits by Linkage Analysis with Genome-Wide SNPs: Framingham SHARe. Bone 2010, 46 (4), 1114–1121. 10.1016/j.bone.2010.01.001. 20064633 PMC2842472

[ref16] Trajanoska K.; Rivadeneira F. The Genetic Architecture of Osteoporosis and Fracture Risk. Bone 2019, 126, 2–10. 10.1016/j.bone.2019.04.005. 30980960

[ref17] Kanis J. A.; Glüer C. C. An Update on the Diagnosis and Assessment of Osteoporosis with Densitometry. Committee of Scientific Advisors, International Osteoporosis Foundation. Osteoporos Int 2000, 11 (3), 192–202. 10.1007/s001980050281. 10824234

[ref18] Kanis J. A.; Johnell O.; Oden A.; De Laet C.; Jonsson B.; Dawson A. Ten-Year Risk of Osteoporotic Fracture and the Effect of Risk Factors on Screening Strategies. Bone 2002, 30 (1), 251–258. 10.1016/S8756-3282(01)00653-6. 11792594

[ref19] Rivadeneira F.; Mäkitie O. Osteoporosis and Bone Mass Disorders: From Gene Pathways to Treatments. Trends Endocrinol Metab 2016, 27 (5), 262–281. 10.1016/j.tem.2016.03.006. 27079517

[ref20] Rivadeneira F.; Styrkársdottir U.; Estrada K.; Halldórsson B. V; Hsu Y.-H.; Richards J. B.; Zillikens M. C.; Kavvoura F. K.; Amin N.; Aulchenko Y. S.; et al. Twenty Bone-Mineral-Density Loci Identified by Large-Scale Meta-Analysis of Genome-Wide Association Studies. Nat. Genet. 2009, 41 (11), 1199–1206. 10.1038/ng.446. 19801982 PMC2783489

[ref21] Estrada K.; Styrkarsdottir U.; Evangelou E.; Hsu Y.-H.; Duncan E. L.; Ntzani E. E.; Oei L.; Albagha O. M. E.; Amin N.; Kemp J. P.; et al. Genome-Wide Meta-Analysis Identifies 56 Bone Mineral Density Loci and Reveals 14 Loci Associated with Risk of Fracture. Nat. Genet. 2012, 44 (5), 491–501. 10.1038/ng.2249. 22504420 PMC3338864

[ref22] Zheng H.-F.; Forgetta V.; Hsu Y.-H.; Estrada K.; Rosello-Diez A.; Leo P. J.; Dahia C. L.; Park-Min K. H.; Tobias J. H.; Kooperberg C.; et al. Whole-Genome Sequencing Identifies EN1 as a Determinant of Bone Density and Fracture. Nature 2015, 526 (7571), 112–117. 10.1038/nature14878. 26367794 PMC4755714

[ref23] Langdahl B. L.; Uitterlinden A. G.; Ralston S. H.; Trikalinos T. A.; Balcells S.; Brandi M. L.; Scollen S.; Lips P.; Lorenc R.; Obermayer-Pietsch B.; et al. Large-Scale Analysis of Association between Polymorphisms in the Transforming Growth Factor Beta 1 Gene (TGFB1) and Osteoporosis: The GENOMOS Study. Bone 2008, 42 (5), 969–981. 10.1016/j.bone.2007.11.007. 18284942

[ref24] Morris J. A.; Kemp J. P.; Youlten S. E.; Laurent L.; Logan J. G.; Chai R. C.; Vulpescu N. A.; Forgetta V.; Kleinman A.; Mohanty S. T.; et al. An Atlas of Genetic Influences on Osteoporosis in Humans and Mice. Nat. Genet. 2019, 51 (2), 258–266. 10.1038/s41588-018-0302-x. 30598549 PMC6358485

[ref25] Liu Y.-J.; Zhang L.; Papasian C. J.; Deng H.-W. Genome-Wide Association Studies for Osteoporosis: A 2013 Update. J Bone Metab 2014, 21 (2), 99–116. 10.11005/jbm.2014.21.2.99. 25006567 PMC4075273

[ref26] Trajanoska K.; Morris J. A.; Oei L.; Zheng H.-F.; Evans D. M.; Kiel D. P.; Ohlsson C.; Richards J. B.; Rivadeneira F.; et al. Assessment of the Genetic and Clinical Determinants of Fracture Risk: Genome Wide Association and Mendelian Randomisation Study. BMJ 2018, 362, k3225 10.1136/bmj.k3225. 30158200 PMC6113773

[ref27] Cummings S. R.; Rosen C. VITAL Findings - A Decisive Verdict on Vitamin D Supplementation. N Engl J Med 2022, 387 (4), 368–370. 10.1056/NEJMe2205993. 35939583

[ref28] Vignal A.; Milan D.; SanCristobal M.; Eggen A. A Review on SNP and Other Types of Molecular Markers and Their Use in Animal Genetics. Genet Sel Evol 2002, 34 (3), 275–305. 10.1186/1297-9686-34-3-275. 12081799 PMC2705447

[ref29] Liao P.-Y.; Lee K. H. From SNPs to Functional Polymorphism: The Insight into Biotechnology Applications. Biochem Eng J 2010, 49 (2), 149–158. 10.1016/j.bej.2009.12.021.

[ref30] Zhang J.; Yang J.; Zhang L.; Luo J.; Zhao H.; Zhang J.; Wen C. A New SNP Genotyping Technology Target SNP-Seq and Its Application in Genetic Analysis of Cucumber Varieties. Sci Rep 2020, 10 (1), 5623 10.1038/s41598-020-62518-6. 32221398 PMC7101363

[ref31] Semagn K.; Babu R.; Hearne S.; Olsen M. Single Nucleotide Polymorphism Genotyping Using Kompetitive Allele Specific PCR (KASP): Overview of the Technology and Its Application in Crop Improvement. Molecular Breeding 2014, 33 (1), 1–14. 10.1007/s11032-013-9917-x.

[ref32] Balagué-Dobón L.; Cáceres A.; González J. R. Fully Exploiting SNP Arrays: A Systematic Review on the Tools to Extract Underlying Genomic Structure. Brief Bioinform 2022, 23 (2), bbac043 10.1093/bib/bbac043. 35211719 PMC8921734

[ref33] Wang D. G.; Fan J. B.; Siao C. J.; Berno A.; Young P.; Sapolsky R.; Ghandour G.; Perkins N.; Winchester E.; Spencer J.; Kruglyak L.; Stein L.; Hsie L.; Topaloglou T.; Hubbell E.; Robinson E.; Mittmann M.; Morris M. S.; Shen N.; Kilburn D.; Rioux J.; Nusbaum C.; Rozen S.; Hudson T. J.; Lipshutz R.; Chee M.; Lander E. S. Large-Scale Identification, Mapping, and Genotyping of Single-Nucleotide Polymorphisms in the Human Genome. Science 1998, 280 (5366), 1077–1082. 10.1126/science.280.5366.1077. 9582121

[ref34] Drogou C.; Sauvet F.; Erblang M.; Detemmerman L.; Derbois C.; Erkel M. C.; Boland A.; Deleuze J. F.; Gomez-Merino D.; Chennaoui M. Genotyping on Blood and Buccal Cells Using Loop-Mediated Isothermal Amplification in Healthy Humans. Biotechnology Reports 2020, 26, e00468 10.1016/j.btre.2020.e00468. 32461926 PMC7240324

[ref35] Higgins O.; Smith T. J. Loop-Primer Endonuclease Cleavage-Loop-Mediated Isothermal Amplification Technology for Multiplex Pathogen Detection and Single-Nucleotide Polymorphism Identification. The Journal of Molecular Diagnostics 2020, 22 (5), 640–651. 10.1016/j.jmoldx.2020.02.002. 32409120

[ref36] Michiyuki S.; Tomita N.; Mori Y.; Kanda H.; Tashiro K.; Notomi T. Discrimination of a Single Nucleotide Polymorphism in the Haptoglobin Promoter Region, Rs5472, Using a Competitive Fluorophore-Labeled Probe Hybridization Assay Following Loop-Mediated Isothermal Amplification. Biosci Biotechnol Biochem 2021, 85 (2), 359–368. 10.1093/bbb/zbaa012. 33604636

[ref37] Shen H.; Wen J.; Liao X.; Lin Q.; Zhang J.; Chen K.; Wang S.; Zhang J. A Sensitive, Highly Specific Novel Isothermal Amplification Method Based on Single-Nucleotide Polymorphism for the Rapid Detection of Salmonella Pullorum. Front Microbiol 2020, 11, 10.3389/fmicb.2020.560791. PMC7575712 33117307

[ref38] Luca Tiscia G.; Colaizzo D.; Vergura P.; Favuzzi G.; Chinni E.; Vandermeulen C.; Detemmerman L.; Grandone E. Loop-Mediated Isothermal Amplification (LAMP)-Based Method for Detecting Factor V Leiden and Factor II G20210A Common Variants. J Thromb Thrombolysis 2020, 50 (4), 908–912. 10.1007/s11239-020-02183-8. 32557225

[ref39] Varona M.; Eitzmann D. R.; Pagariya D.; Anand R. K.; Anderson J. L. Solid-Phase Microextraction Enables Isolation of BRAF V600E Circulating Tumor DNA from Human Plasma for Detection with a Molecular Beacon Loop-Mediated Isothermal Amplification Assay. Anal. Chem. 2020, 92 (4), 3346–3353. 10.1021/acs.analchem.9b05323. 31950824 PMC7155775

[ref40] Gill P.; Hadian Amree A. AS-LAMP: A New and Alternative Method for Genotyping. Avicenna J. Med. Biotechnol. 2020, 12 (1), 2–8. 32153732 PMC7035465

[ref41] Lázaro A.; Yamanaka E.; Maquieira A.; Tortajada-Genaro L. Allele-Specific Ligation and Recombinase Polymerase Amplification for the Detection of Single Nucleotide Polymorphisms. Sens Actuators B Chem 2019, 298, 126877 10.1016/j.snb.2019.126877.

[ref42] Yamanaka E. S.; Tortajada-Genaro L. A.; Maquieira A. Low-Cost Genotyping Method Based on Allele-Specific Recombinase Polymerase Amplification and Colorimetric Microarray Detection. Microchimica Acta 2017, 184 (5), 1453–1462. 10.1007/s00604-017-2144-0.

[ref43] Ahmed M.; Pollak N. M.; Devine G. J.; Macdonald J. Detection of a Single Nucleotide Polymorphism for Insecticide Resistance Using Recombinase Polymerase Amplification and Lateral Flow Dipstick Detection. Sens Actuators B Chem 2022, 367, 132085 10.1016/j.snb.2022.132085.

[ref44] de Olazarra A. S.; Cortade D. L.; Wang S. X. From Saliva to SNP: Non-Invasive, Point-of-Care Genotyping for Precision Medicine Applications Using Recombinase Polymerase Amplification and Giant Magnetoresistive Nanosensors. Lab Chip 2022, 22 (11), 2131–2144. 10.1039/D2LC00233G. 35537344 PMC9156572

[ref45] Natoli M. E.; Chang M. M.; Kundrod K. A.; Coole J. B.; Airewele G. E.; Tubman V. N.; Richards-Kortum R. R. Allele-Specific Recombinase Polymerase Amplification to Detect Sickle Cell Disease in Low-Resource Settings. Anal. Chem. 2021, 93 (11), 4832–4840. 10.1021/acs.analchem.0c04191. 33689292 PMC7992048

[ref46] Sokolov B. P. Primer Extension Technique for the Detection of Single Nucleotide in Genomic DNA. Nucleic Acids Res. 1990, 18 (12), 3671 10.1093/nar/18.12.3671. 2194170 PMC331055

[ref47] Kuppuswamy M. N.; Hoffmann J. W.; Kasper C. K.; Spitzer S. G.; Groce S. L.; Bajaj S. P. Single Nucleotide Primer Extension to Detect Genetic Diseases: Experimental Application to Hemophilia B (Factor IX) and Cystic Fibrosis Genes. Proc Natl Acad Sci U S A 1991, 88 (4), 1143–1147. 10.1073/pnas.88.4.1143. 1671714 PMC50973

[ref48] Picketts D. J.; Cameron C.; Taylor S. A.; Deugau K. V; Lillicrap D. P. Differential Termination of Primer Extension: A Novel, Quantifiable Method for Detection of Point Mutations. Hum. Genet. 1992, 89 (2), 155–157. 10.1007/BF00217115. 1587524

[ref49] Sanger F.; Nicklen S.; Coulson A. R. DNA Sequencing with Chain-Terminating Inhibitors. Proc Natl Acad Sci U S A 1977, 74 (12), 5463–5467. 10.1073/pnas.74.12.5463. 271968 PMC431765

[ref50] Syvänen A. C.; Aalto-Setälä K.; Harju L.; Kontula K.; Söderlund H. A Primer-Guided Nucleotide Incorporation Assay in the Genotyping of Apolipoprotein E. Genomics 1990, 8 (4), 684–692. 10.1016/0888-7543(90)90255-S. 2276739

[ref51] Syvänen A. C.; Sajantila A.; Lukka M. Identification of Individuals by Analysis of Biallelic DNA Markers, Using PCR and Solid-Phase Minisequencing. Am. J. Hum. Genet. 1993, 52 (1), 46–59. 8434605 PMC1682118

[ref52] Pastinen T.; Partanen J.; Syvänen A. C. Multiplex, Fluorescent, Solid-Phase Minisequencing for Efficient Screening of DNA Sequence Variation. Clin Chem 1996, 42 (9), 1391–1397. 10.1093/clinchem/42.9.1391. 8787694

[ref53] Shumaker J. M.; Metspalu A.; Caskey C. T. Mutation Detection by Solid Phase Primer Extension. Hum Mutat 1996, 7 (4), 346–354. 10.1002/(SICI)1098-1004(1996)7:4<346::AID-HUMU9>3.0.CO;2-6. 8723685

[ref54] Erdogan F.; Kirchner R.; Mann W.; Ropers H. H.; Nuber U. A. Detection of Mitochondrial Single Nucleotide Polymorphisms Using a Primer Elongation Reaction on Oligonucleotide Microarrays. Nucleic Acids Res. 2001, 29 (7), e36 10.1093/nar/29.7.e36. 11266571 PMC31297

[ref55] Huber M.; Losert D.; Hiller R.; Harwanegg C.; Mueller M. W.; Schmidt W. M. Detection of Single Base Alterations in Genomic DNA by Solid Phase Polymerase Chain Reaction on Oligonucleotide Microarrays. Anal. Biochem. 2001, 299 (1), 24–30. 10.1006/abio.2001.5355. 11726180

[ref56] Liu J. L.; Ma Y. C.; Yang T.; Hu R.; Yang Y. H. A Single Nucleotide Polymorphism Electrochemical Sensor Based on DNA-Functionalized Cd-MOFs-74 as Cascade Signal Amplification Probes. Mikrochim. Acta 2021, 188 (8), 266 10.1007/s00604-021-04924-9. 34291388

[ref57] Trau D.; Lee T. M. H.; Lao A. I. K.; Lenigk R.; Hsing I.-M.; Ip N. Y.; Carles M. C.; Sucher N. J. Genotyping on a Complementary Metal Oxide Semiconductor Silicon Polymerase Chain Reaction Chip with Integrated DNA Microarray. Anal. Chem. 2002, 74 (13), 3168–3173. 10.1021/ac020053u. 12141679

[ref58] Brazill S.; Hebert N. E.; Kuhr W. G. Use of an Electrochemically Labeled Nucleotide Terminator for Known Point Mutation Analysis. Electrophoresis 2003, 24 (16), 2749–2757. 10.1002/elps.200305554. 12929170

[ref59] Ortiz M.; Jauset-Rubio M.; Kodr D.; Simonova A.; Hocek M.; O’Sullivan C. K. Solid-Phase Recombinase Polymerase Amplification Using Ferrocene-Labelled DNTPs for Electrochemical Detection of Single Nucleotide Polymorphisms. Biosens Bioelectron 2022, 198, 113825 10.1016/j.bios.2021.113825. 34838372

[ref60] Brázdilová P.; Vrábel M.; Pohl R.; Pivonková H.; Havran L.; Hocek M.; Fojta M. Ferrocenylethynyl Derivatives of Nucleoside Triphosphates: Synthesis, Incorporation, Electrochemistry, and Bioanalytical Applications. Chem.-Eur. J. 2007, 13 (34), 9527–9533. 10.1002/chem.200701249. 17896337

[ref61] Ménová P.; Raindlová V.; Hocek M. Scope and Limitations of the Nicking Enzyme Amplification Reaction for the Synthesis of Base-Modified Oligonucleotides and Primers for PCR. Bioconjug Chem 2013, 24 (6), 1081–1093. 10.1021/bc400149q. 23682869

[ref62] Simonova A.; Magriñá I.; Sýkorová V.; Pohl R.; Ortiz M.; Havran L.; Fojta M.; O’Sullivan C. K.; Hocek M. Tuning of Oxidation Potential of Ferrocene for Ratiometric Redox Labeling and Coding of Nucleotides and DNA. Chemistry 2020, 26 (6), 1286–1291. 10.1002/chem.201904700. 31725178 PMC7384099

[ref63] Wang J. Analytical Electrochemistry, 3rd ed.; Wiley-VCH: Hoboken, NJ, 2006.

[ref64] Ortiz M.; Jauset-Rubio M.; Skouridou V.; Machado D.; Viveiros M.; Clark T. G.; Simonova A.; Kodr D.; Hocek M.; O’Sullivan C. K. Electrochemical Detection of Single-Nucleotide Polymorphism Associated with Rifampicin Resistance in Mycobacterium Tuberculosis Using Solid-Phase Primer Elongation with Ferrocene-Linked Redox-Labeled Nucleotides. ACS Sens 2021, 6 (12), 4398–4407. 10.1021/acssensors.1c01710. 34797987 PMC8715531

[ref65] Jauset-Rubio M.; Ortiz M.; O’Sullivan C. K. Solid-Phase Primer Elongation Using Biotinylated DNTPs for the Detection of a Single Nucleotide Polymorphism from a Fingerprick Blood Sample. Anal. Chem. 2021, 93 (44), 14578–14585. 10.1021/acs.analchem.1c03419. 34704755 PMC8581964

[ref66] Koek W. N. H.; van Meurs J. B.; van der Eerden B. C. J.; Rivadeneira F.; Zillikens M. C.; Hofman A.; Obermayer-Pietsch B.; Lips P.; Pols H. A.; Uitterlinden A. G.; van Leeuwen J. P. T. M. The T-13910C Polymorphism in the Lactase Phlorizin Hydrolase Gene Is Associated with Differences in Serum Calcium Levels and Calcium Intake. J Bone Miner Res 2010, 25 (9), 1980–1987. 10.1002/jbmr.83. 20225268

[ref67] Jauset-Rubio M.; Tomaso H.; El-Shahawi M. S.; Bashammakh A. S.; Al-Youbi A. O.; O’Sullivan C. K. Duplex Lateral Flow Assay for the Simultaneous Detection of Yersinia Pestis and Francisella Tularensis. Anal. Chem. 2018, 90 (21), 12745–12751. 10.1021/acs.analchem.8b03105. 30296053

[ref68] Wu Q.; Jung J. Genome-Wide Polygenic Risk Score for Major Osteoporotic Fractures in Postmenopausal Women Using Associated Single Nucleotide Polymorphisms. J Transl Med 2023, 21 (1), 127 10.1186/s12967-023-03974-2. 36797788 PMC9933300

[ref69] Trajanoska K.; Morris J. A.; Oei L.; Zheng H.-F.; Evans D. M.; Kiel D. P.; Ohlsson C.; Richards J. B.; Rivadeneira F. GEFOS/GENOMOS consortium and the 23andMe research team. Assessment of the Genetic and Clinical Determinants of Fracture Risk: Genome Wide Association and Mendelian Randomisation Study. BMJ 2018, 362, k3225 10.1136/bmj.k3225. 30158200 PMC6113773

[ref70] Movérare-Skrtic S.; Henning P.; Liu X.; Nagano K.; Saito H.; Börjesson A. E.; Sjögren K.; Windahl S. H.; Farman H.; Kindlund B.; et al. Osteoblast-Derived WNT16 Represses Osteoclastogenesis and Prevents Cortical Bone Fragility Fractures. Nat Med 2014, 20 (11), 1279–1288. 10.1038/nm.3654. 25306233 PMC4392888

[ref71] van Bezooijen R. L.; ten Dijke P.; Papapoulos S. E.; Löwik C. W. G. M. SOST/Sclerostin, an Osteocyte-Derived Negative Regulator of Bone Formation. Cytokine Growth Factor Rev 2005, 16 (3), 319–327. 10.1016/j.cytogfr.2005.02.005. 15869900

[ref72] Estrada K.; Styrkarsdottir U.; Evangelou E.; Hsu Y.-H.; Duncan E. L.; Ntzani E. E.; Oei L.; Albagha O. M. E.; Amin N.; Kemp J. P.; et al. Genome-Wide Meta-Analysis Identifies 56 Bone Mineral Density Loci and Reveals 14 Loci Associated with Risk of Fracture. Nat. Genet. 2012, 44 (5), 491–501. 10.1038/ng.2249. 22504420 PMC3338864

[ref73] Nilsson K. H.; Henning P.; El Shahawy M.; Nethander M.; Andersen T. L.; Ejersted C.; Wu J.; Gustafsson K. L.; Koskela A.; Tuukkanen J.; et al. RSPO3 Is Important for Trabecular Bone and Fracture Risk in Mice and Humans. Nat Commun 2021, 12 (1), 4923 10.1038/s41467-021-25124-2. 34389713 PMC8363747

[ref74] Tanaka K.; Xue Y.; Nguyen-Yamamoto L.; Morris J. A.; Kanazawa I.; Sugimoto T.; Wing S. S.; Richards J. B.; Goltzman D. FAM210A Is a Novel Determinant of Bone and Muscle Structure and Strength. Proceedings of the National Academy of Sciences 2018, 115 (16), E3759–E3768. 10.1073/pnas.1719089115. PMC5910842 29618611

[ref75] Obermayer-Pietsch B. M.; Bonelli C. M.; Walter D. E.; Kuhn R. J.; Fahrleitner-Pammer A.; Berghold A.; Goessler W.; Stepan V.; Dobnig H.; Leb G.; Renner W. Genetic Predisposition for Adult Lactose Intolerance and Relation to Diet, Bone Density, and Bone Fractures. Journal of Bone and Mineral Research 2004, 19 (1), 42–47. 10.1359/jbmr.0301207. 14753735

[ref76] Torkamani A.; Wineinger N. E.; Topol E. J. The Personal and Clinical Utility of Polygenic Risk Scores. Nat Rev Genet 2018, 19 (9), 581–590. 10.1038/s41576-018-0018-x. 29789686

