# Supplementary material for: Generic Platform for the Multiplexed Targeted Electrochemical Detection of Osteoporosis-Associated Single Nucleotide Polymorphisms Using Recombinase Polymerase Solid-Phase Primer Elongation and Ferrocene-Modified Nucleoside Triphosphates
Source: ACS Cent Sci. 2023 Jul 19;9(8):1591–602. doi: 10.1021/acscentsci.3c00243 (PMC10450878; doi:10.1021/acscentsci.3c00243)
Supplement: Supplementary file 1 — oc3c00243_si_001.pdf [file oc3c00243_si_001.pdf]

Supporting Information

Generic platform for the multiplexed targeted electrochemical detection of osteoporosis-associated single nucleotide polymorphisms using recombinase polymerase solid-phase primer elongation and ferrocene modified nucleoside triphosphates

Mayreli Ortiz,[a]† Miriam Jauset-Rubio,[a]† Olivia Trummer,[b] Ines Foessl,[b] David Kodr,[c] Josep Lluis Acero,[a] Mary Luz Botero,[a] Phil Biggs,[d] Daniel Lenartowicz,[d] Katerina Trajanoska,[e] Fernando Rivadeneira,[e] Michal Hocek,[c,f] Barbara Obermayer-Pietsch,[b] and Ciara K. O`Sullivan[a,g]*

[a] Department d'Enginyeria Química, Universitat Rovira i Virgili, Avinguda Països Catalans 26, 43007 Tarragona, Spain

[b] Division of Endocrinology and Diabetology, Department of Internal Medicine, Medical University of Graz, 8036 Graz, Austria

[c] Institute of Organic Chemistry and Biochemistry, Czech Academy of Sciences, Flemingovo namesti 2, CZ-16610 Prague 6, Czech Republic

[d] Labman Automation Ltd. is Seamer Hill, Stokesley, North Yorkshire, TS9 5NQ, UK.

[e] Department of Internal Medicine Erasmus MC Rotterdam, The Netherlands

[f] Department of Organic Chemistry, Faculty of Science, Charles University, Hlavova 8, CZ-12843 Prague 2, Czech Republic

[g] ICREA, Passeig Lluis Companys 23, 08010 Barcelona, Spain

† These authors contributed equally to this work.

* Corresponding author at: Departament d'Enginyeria Química, Universitat Rovira i Virgili, Avinguda Països Catalans 26, 43007 Tarragona, Spain. E-mail address: ciara.osullivan@urv.cat (C.K. O´Sullivan).

Table Contents

1. Methods 4

Materials 4

General procedure for gel electrophoresis 4

Primer design and evaluation 5

Double stranded DNA targets from synthetic sequences for optimisation studies 5

Real samples used for genomic sensor validation 6

Solid phase RPA for simultaneous detection of 5 SNP related to osteoporosis on maleimide activated plate and colorimetric detection 6

Solid phase amplification based on electrode assay 6

Electrode array functionalisation 8

Electrochemical detection of solid-phase primer elongation 8

SNP detection using TaqMan fluorogenic 5-exonuclease assay. 9

Sanger sequencing 9

2. Tables 11

Table S1. Sequences of the primer sets used in the final assay and the sequences containing the SNP site. (There are two SNP-related specific primers and two other primers (with non-SNP specific terminal bases) used as negative controls.) 11

Table S2. TaqMan probes and the SNPs to be detected. 14

Table S3. Designed DNA sequences and primers for Sanger sequencing (primers are underlined and the SNP under interrogation is italicised). 15

3. Figures and Captions 18

Figure S1. Schematic representation of the SNP expected in a diploid organism. 18

Figure S2. Primer specificity evaluation using *Primer Blast* and nucleotide collection parameter for each set of primers. 22

Figure S3. Self-dimers and primer-dimers checked by Multiple Primer Analyzer software. All the forward primers were tested again each reverse primer. 24

Figure S4. Agarose gel demonstrating the correct design and specificity of all the SNPs sequences by (A) PCR and (B) Liquid-phase RPA. 25

Figure S5. Solid phase RPA (15 min at 37°C) for simultaneous detection of 5 SNP related to osteoporosis on maleimide activated plate and colorimetric detection using synthetic targets (Table S1). β-globin was used as positive control of the reaction. 25

Figure S6. A) Mapping of electrode array provided by LABMAN and the pattern of electrode array functionalisation. The SNP related primers are highlighted in different colours (red for primers ending in T for SNPs A, blue for primers ending in C for SNPs G and green for primers ending in G for SNPs C) while the negative control primers are represented in grey and black colours). Finally, the positive controls (β-globin and Poly-Fc) are in pink and magenta colours. B) Real picture of electrode array during functionalisation process containing the drops over the electrodes. 26

Figure S7. A) AUTOCAD view of the electrode array. B) Real picture of the electrode array, double adhesive gasket and PMMA. C) Schematic representation of the 64 break-out box connected to the potentionstat and electrode array. D) Laboratory set-up used for genomic sensor validation and vertical view of the electrode array inside the microfluidic cell and connected to the potentiostat. 27

Figure S8. SWVs obtained during the flow-through of the optimisation of the concentration of the five Forward Primers (FwP) for simultaneous detection of the five SNPs related to osteoporosis using solid phase RPA, $dN^{Fc}TPs$ and electrochemical detection. The targets were prepared from synthetic sequences from Table S1. 28

Figure S10. SWV recorded in 0.1 M $Sr(NO_3)_2$ + 0.1 M Glycine pH 3 for simultaneous electrochemical detection of 5 SNPs for 10 more human whole blood samples. The SNP related primers are highlighted in different colours (red for primers ended in T for SNPs A, blue for primers ended in C for SNPs G and green for primers ended in G for SNPs C) while the negative primers are represented black colour and discontinuous traces. 38

Figure S11. TaqMan fluorogenic 5-exonuclease assay for SNP 10, SNP 27, SNP 29, SNP 46 and SNP 49: A) Allelic discrimination plots show three clusters with assigned genotypes as well as a zone for the no template controls (NTCs), shown in red, near the origin. Fluorescent endpoint data points in each cluster are grouped closely together and each cluster is located well away from the other clusters as well as from the NTCs. Data points in the upper left corner show allele 1 (homozygote), labelled with VIC dyes. Data points in the lower right corner

display allele 2 (homozygote), labelled with FAM dyes. Data points approximately midway between allele 1 and allele 2 clusters show a mixed (heterozygote) signal for allele 1 and allele 2, double labelled with VIC and FAM dyes. No template controls are located in the lower left corner. B) Raw end-point fluorescent TaqMan PCR data. C) Evaluated genotypes according to allelic discrimination plots. 40

Figure S12. Chromatograms of the SNPs region obtained by Sanger sequencing using the *Bioedit* software. 44

Figure S13. Alignment of the obtained sequences by Sanger sequencing compared with their synthetic sequence DNA using *Blastn* software. 74

Figure S14: Plot representing data detailed in Table 3. Cases 9, 10 represent cases with a high risk of developing osteoporosis. 75

4. References 75

1. Methods

Materials

The chemical reagents were used as received. The solutions used for electrochemistry and gel electrophoresis were prepared with ultrapure water (18 MΩ·cm) purified by a Simplicity Water Purification System (Millipore, France) and DNase free water from Fisher Scientific (Spain) was used for the solutions required for enzymatic reactions. Strontium nitrate ($Sr(NO_3)_2$), sodium chloride (NaCl), potassium hydroxide (KOH), boric acid (H_3BO_3) and potassium dihydrogen phosphate (KH_2PO_4) were provided by Fisher Scientific (Spain), while hydrochloric acid (35% v/v) was purchased from Panreac, and Tween-20, disodium Na_2EDTA, skimmed milk powder, 3,3′,5,5′-tetramethylbenzidine (TMB), glycine-HCl and mercaptohexanol (MCH) from Merck (Sigma Aldrich, Spain).

Fisher Scientific (Spain) was the supplier of maleimide activated plates (8-well strips), Dream Taq polymerase, GeneRuler Low Range DNA Ladder, agarose broad separation range for DNA/RNA (genetic analysis grade) and unmodified dNTPs; while Biotin-16-dCTP was purchased from Jena Bioscience (Jena, Germany).

TwistDX (UK) was the provider of the Recombinase Polymerase Amplification (RPA) kit (TwistAmp® Liquid Basic kit TALQBAS01). Streptavidin Poly-HRP80 was supplied from SDT-reagents (Baesweiler, Germany) and the DNA Clean & Concentrator kit was received from Ecogen (Spain).

The ferrocene labelled deoxynucleotides $dA^{Fc}TP$ and $dU^{Fc}TP$ [1] $dG^{Fc}TP$ [2] and $dC^{Fc}TP$ [3] were synthesized as previously reported. The nucleotides $dA^{Fc}TP$ and $dC^{Fc}TP$ are also commercially available from Santiago-Labs, Check Republic (https://www.santiago-lab.com) *(Warning: If these compounds are stored as powder at -20ºC, before opening, the bottle must be transferred to a vacuum system for at least 20 min to reach room temperature, to avoid hydration. Once dissolved in water, these compounds should be aliquoted to avoid sequential freezing and thawing).* The sequences of oligonucleotides were purchased from Biomers.net (Germany).

General procedure for gel electrophoresis

As general procedure to evaluate the amplification reaction after PCR and RPA liquid phase, 5 µL of PCR / RPA products were mixed with 4 µL of 6 x loading buffer and run in 2.6% (w/v) agarose gel prepared in 1 x TBE buffer (Tris-Borate-EDTA, pH 8) at 110 mV for 20 min. The gel was pre-stained with GelRed nucleic acid stain (VWR, Spain) and imaged with a UV lamp (λ = 254 nm).

Primer design and evaluation

The sequences of the corresponding SNPs were found using the SNPedia website (https://www.snpedia.com/index.php/SNPedia).

The primer design was performed using two different open resource softwares, Primer blast software (https://www.ncbi.nlm.nih.gov/tools/primer-blast/) and Multiple Primer Analyzer software (https://www.thermofisher.com/es/es/home/brands/thermo-scientific/molecular-biology/molecular-biology-learning-center/molecular-biology-resource-library/thermo-scientific-web-tools/multiple-primer-analyzer.html). Primer Blast was used to obtain primers with similar Tm, GC content and to produce amplicons ranging from 80 to 150 bp (Table S1). The specificity of the designed primers was confirmed using the "nr" parameter (Figure S2). The Multiple Primer Analyzer software, was used to ensure that no self-dimers or primer-dimers would be obtained (Figure S3).

For the experimental evaluation of the primers, PCR and liquid phase RPA were performed. PCR was carried out with 50 µL of master mix reagents containing 1 × Dream Taq buffer, 200 nM of each forward primer, 200 nM of desired reverse primer, 0.2 µM dNTPs, 1 U Dream Taq polymerase and 100 pM of the desired synthetic DNA. The program used was 95 °C for 2 min, followed by 25 rounds of PCR with 30 s of denaturation at 95 °C, 30 s of annealing at 60 °C, and 30 s of elongation at 72 °C, with a final elongation step at 72 °C for 5 min. RPA was performed according to the manufacturer's instructions (TwistAmp Liquid Basic kit, TwistDx, Cambridge, UK). Briefly, 50 µL of RPA reagents (1 × rehydration buffer, 1 × basic E-mix, 1 × core reaction mix, 0.5 µM of each forward primer, 0.5 µM of the desired reverse primer, 0.2 mM dNTPs, 10 mM $Mg(OAc)_2$), 100 pM of dsDNA of the desired target (dsDNA prepared by PCR as described in the next section) were mixed and incubated for 15 min at 37 °C. Prior to running the samples in a 2.6% agarose gel electrophoresis the RPA reaction was stopped by heating the samples to 80 °C for 10 min.

Double stranded DNA targets from synthetic sequences for optimisation studies

For optimisation of the RPA parameters, double stranded DNA sequences produced using PCR were used to mimic the amplicons that would be obtained from the genomic DNA target. [4] Using the PCR protocol described above, five individual PCR reactions were performed using the specific pair of forward and reverse primers for each synthetic amplicon (Table S1). The products were purified using the DNA Clean and Concentrator

kit, visualised by gel electrophoresis and quantified by SimpliNano spectrophotomer. The generated double stranded DNAs were stored at -20°C until use.

Real samples used for genomic sensor validation

Fifteen human whole blood samples were selected from the Biobank of the Medical University of Graz, Austria. Genomic DNA was extracted and purified from five of these samples following NucleoSpin Blood Kit (Machery and Nagel 740951.250) to evaluate the effect of the blood matrix on the isothermal solid-phase primer elongation approach used for SNP detection.

Solid phase RPA for simultaneous detection of 5 SNP related to osteoporosis on maleimide activated plate and colorimetric detection

Firstly, the maleimide activated plate was washed three times with PBS-Tween. The five sets of thiolated reverse primers (Table S1: 4 primers per each SNP) were accommodated in individual wells of the plate by adding one hundred microlitres of each primer solution (200 nM in PBS) in triplicate, and the thiolated reverse primers of β-globin housekeeping gene as a positive control. After overnight incubation at 4 ^{o}C, the wells were washed with 200 µL PBS-tween, treated with 200 µL of 5% w/v skimmed milk in PBS-Tween for 1 hour at 22°C, and washed again with PBS-tween. Fifty µL of RPA reaction mixture (1x Rehydration buffer, 1x Basic E-mix, 1x Core Reaction mix, 10 mM $Mg(OAc)_2$, 0.2 mM dNTPs (containing dATP, dGTP, dTTP with 20% of biotin-16-dCTP/dCTP), and the mixture of the five Fw primers (Table S1) and 100 pM of dsDNA targets) were added to each well and incubated at 37°C for 15 min. After reaction, the wells were treated with 200 µL of 100 mM NaOH and washed with PBS-Tween before the addition of SA-poly-HRP80 (50 µL of dilution 1 in 20000 of 1 mg/mL) in PBS-tween. After 30 min of incubation at 22 ºC, the wells were washed with PBS-Tween. Finally, 50 µL of TMB substrate was added, and after 5 min, the reaction was stopped by the addition of an equal volume of 1 M H_2SO_4. The absorbance was read at 450 nm (Figure S5).

Solid phase amplification based on electrode assay

The electrode arrays were designed at URV using AUTOCAD software (Figure S7). They are one-layer screen printed electrodes with a board layout size of 54.93 x 55.89 mm and composed by 64 gold working electrodes (WE) (1.0 mm diameter) and distributed in 4 channels (16 electrodes/channel), and common reference (RE) and

counter electrodes (CE) also printed of gold. The electrical contacts were made of gold while the tracks were made of silver.

For electrode fabrication, the screens were ordered from ASM DEK printing solutions (https://psp.smt.asmpt.com/en/asm-stencils/). The screen was a 45 deg SD90/40 SST mesh, mesh size 230 mm, thinnest emulsion and framed in a DEK260 frame. The inks cured at a low temperature (gold paste: Ref.C2090908D1; silver termination paste: Ref. C2050926P2 and grey dielectric paste: Ref: D2070423P5) were ordered from Gwent Group Ltd, UK. The electrodes were then fabricated at C-MAC Electronics Solutions, Belgium, based on the ink manufacturer guidelines (bake: 30 min at 80 °C for all three inks and the following conditions for screen printing: for gold layer: print speed: 50 mm/s, 50 N print pressure, snap-off: 1.2 mm and no flood printing; for silver layer: print speed: 200 mm/s, print pressure: 0 N, snap-off: 1.2 mm and flood printing applied; for insulation layer: print speed: 50 mm/s; print pressure: 50 N, snap-off: 1.0 mm and flood printing applied) and 200 μm was suggested as the smallest feature to be reproducibly printed using these screens and inks.

The PMMA microfluidics and double adhesive tapes were also designed at URV (Figure S7B) and cut using the CO_2 laser at URV facilities. The double adhesive tape (Ref. ARcare 90106) was purchased from Adhesive Research, Ireland) while the PMMA for microfluidics fabrication was received from Industria de la Goma, Spain).

Finally, the electrode array, once housed in the microfluidics, was connected to the potentiostat via a break-out box manufactured at Labman, UK (Figure S7C and S7D). This 'break-out' box (Figure S7D) allows the connection of each individual working electrode as well as the common reference and counter electrodes to be routed to the corresponding potentiostat connection. Electrically, the break-out box is straight forward, and provides a 1 to 1 connection from each electrode fabricated on the array to the corresponding connection provided by the Autolab potentiostat and multiplexer arrangement. The electrode arrays were designed to mate with a Samtec FSI- 140-03-G-D-AD one-piece interface in order to connect to external systems. The electrode array incorporated a 2 x 40 matrix of gold connector pads arranged on a pitch of 1.0 mm with a pad width of 0.6 mm. In view of the tight tolerances resulting from the fine pitch, a key requirement of the break-out box was therefore the ability to align the electrode array pads with the break-out connector quickly, repeatably and reliably. To this end a printed adapter plate was incorporated into the breakout-box that enabled precise alignment of

the electrode arrays with the connector. The adapter accommodates both the T shaped FR4 based arrays as well as the square ceramic substrate screen printed electrodes. The Samtec connector is soldered to a carrier PCB to enable wired connections. Inside the break-out enclosure, wired connections were made from the Samtec connector PCB to individual standard 4 mm sockets. As well as WE, CE and RE connections and alignment test points, a chassis ground was produced to reduce extraneous electrical noise.

All the electrochemical measurements were carried out using an Autolab model PGSTAT 12 potentiostat/galvanostat controlled by the General Purpose Electrochemical System (GPES) software (Eco Chemie B.V., the Netherlands) and a Multiplexer module for 64 electrodes.

Electrode array functionalisation

The electrode array was washed with 50 % v/v isopropanol in water, followed by washing with MilliQ water and drying with nitrogen. The electrode functionalisation was carried out following the pattern outlined in Figure S6A and using the primers listed in Table S1. The functionalisation of individual electrodes of the array was carried out by drop-casting 0.5 μL of 5 μM primer with 50 μM 6-mercaptohexanol in 1 M KH_2PO_4 solution, and incubating for 3 h at 37 °C inside a humidity chamber (Figure S6B). Finally, the electrode array was washed with water, dried under nitrogen and stored at 4 °C until use.

Electrochemical detection of solid-phase primer elongation

As previously explained, three types of targets were used in different steps of this study, amplicons generated from synthetic DNA targets (Table S1), genomic DNA extracted and purified from blood samples as well as whole blood samples. The amplicons generated from synthetic DNA targets were quantified and added to the reaction mixture to achieve a final concentration of 100 pM. The extracted and purified genomic DNA was previously quantified using the SimpliNano spectrophotometer and adjusted to a final concentration of 1 ng/mL in the RPA master mix. Finally, for the whole blood samples, a thermal treatment for cell lysis was used, as previously described. [4,5] Briefly, 10 μL of blood was diluted with 40 μL of water and heated for 30 sec at 95ºC. Finally, the target was mixed with 100 μL RPA master mix containing 10 mM of $Mg(OAc)_2$, 200 nM of dNTP (a mixture composed by 30% of mixture of the ferrocene labelled dNTPs ($dA^{Fc}TP$ + $dU^{Fc}TP$ + $dC^{Fc}TP$ + $dG^{Fc}TP$) and 70% of the natural dNTPs (dATP + dUTP + dCTP + dGTP) and the five 5 forward primers. For final approach the concentrations of the

forward primers in the reaction mixture were 0.5 μM for SNP 10 and SNP 49; 0.25 μM for SNP 27 and SNP 49 and 0.125 μM for SNP 46.

Finally, the reaction mixture was injected to the functionalised electrode array via the channels of the patterned PMMA microfluidic cell (Figure S7B). The electrode array was maintained at 37°C for 15 minutes, then gently washed with a solution of 0.1 M glycine in 0.1 M $Sr(NO_3)_2$ (pH 3) and the ferrocene present at the electrodes was measured using square wave voltammetry (SWV). SWVs were recorded from 0 to 0.5 V vs a Au quasi-reference electrode using a pulse amplitude of 0.1 V, a step potential of 10 mV and a frequency of 25 Hz.

SNP detection using TaqMan fluorogenic 5-exonuclease assay.

TaqMan SNP genotyping assays were used as a reference method. In order to achieve optimal discrimination results, PCR reaction mixes were pre-tested with two mastermixes (Gene Expression Master Mix and Universal Master Mix). Reaction mixes were prepared for 5 μL reactions. Briefly, 3 μL of Mastermix for each reaction (2.5 μL of 2 x Gene Expression Mastermix, 0.42 μL purified water and 0.08 μL of 40x Assay mix) were mixed with 2 μL of sample containing approx. 30 ng/μL DNA for SNP 10, SNP 27, SNP 29 and SNP 46. Purified water served as a non-target control for each polymorphism. For SNP 49 PCR pretests show more accurate results with Universal Master Mix. This polymorphism was therefore amplified as described above with the only difference that Universal Master Mix was used instead of the Gene Expression Master Mix. The plate was sealed and briefly centrifuged prior to be transferred to the thermocycler and the TaqMan® cycling protocol was applied (95°C for 10 min following by 40 cycles of 92°C for 15 sec and 60°C for 1 min, with a final step of 4°C). The 40x assay mix contains two allele specific TaqMan probes containing distinct fluorescent dyes (2′-chloro-7′phenyl-1,4-dichloro-6-carboxy-fluorescein (VIC) or 6-carboxyfluorescein (FAM)) as well as a PCR Primer pair to detect specific SNP targets (Table S2). Endpoint fluorescence was measured in a Polarstar Optima plate reader (BMG Labtech, Ortenberg, Germany). Fluorescent data were exported into excel format and analyzed as scatter plots (Figure S11).

Sanger sequencing

PCR products were required to carry out Sanger sequencing. The conditions followed were the same as explained in the primer design section. The amplicons were purified

using a DNA clean & concentrator kit following the manufacturer's instructions. The primer design was carried out as outlined above using Primer Blast and Multiple Primer Analyzer software. The main factor taken into consideration when designing the primers for Sanger sequencing is that the amplicon should start more than 100 bp from the region of interest (SNP) for a correct reading. Primers to produce amplicons ranging from 250 to 600 bp were thus designed. The resulting primers and the expected sequences to be amplified are detailed in Table S3. All the samples were sequenced using the forward primer (Figure S12).

2. Tables

Table S1. Sequences of the primer sets used in the final assay and the sequences containing the SNP site. (There are two SNP-related specific primers and two other primers (with non-SNP specific terminal bases) used as negative controls.)

SNP	Sequence ID	Length (bp)	Sequence
10	rs10457487	124	5'-<br>GTGTTACCCAGTCAAGTATAAGTAGCCAAATTATTT<br>TTGCACATCTTTCTGTTTCTCATGTCTTCATTTATTCA<br>ACAAGCACTTACTGGGAAGGTCTACA***[A/C]***CTGCATA<br>GGCAATGCTGGAAAAA-3’
	Fw 10 primer	29	5’-GTGTTACCCAGTCAAGTATAAGTAGCCAA-3’
	Rev T primer	39	5’-THIOL-C6-TTTTTTTTTTTTTTT-<br>TTTTTCCAGCATTGCCTATGCAG**T**-3’
	Rev G primer	39	5’-THIOL-C6-TTTTTTTTTTTTTTT-<br>TTTTTCCAGCATTGCCTATGCAG**G**-3’
	Non-specific rev A primer	39	5’-THIOL-C6-TTTTTTTTTTTTTTT-<br>TTTTTCCAGCATTGCCTATGCAG**A**-3’
	Non-specific rev C primer	39	5’-THIOL-C6-TTTTTTTTTTTTTTT-<br>TTTTTCCAGCATTGCCTATGCAG**C**-3’
27	rs2741856	107	5'-<br>TTCAATCACACACACACACACACACACACACACACA<br>CACACACACCCCTCTTCACTATAATTATAATTACTAT<br>GTTGGCTTCCA***[C/G]***ATCAGGGGTTAGAGCCTTGGCA<br>-3'
	Fw 27 primer	26	5’-TTCAATCACACACACACACACACACA-3’

	Rev C primer	38	5’-THIOL-C6-TTTTTTTTTTTTTTT-TGCCAAGGCTCTAACCCCTGAT**C**-3’
	Rev G primer	38	5’-THIOL-C6-TTTTTTTTTTTTTTT-TGCCAAGGCTCTAACCCCTGAT**G**-3’
	Non-specific rev A primer	38	5’-THIOL-C6-TTTTTTTTTTTTTTT-TGCCAAGGCTCTAACCCCTGAT**A**-3’
	Non-specific rev T primer	238	5’-THIOL-C6-TTTTTTTTTTTTTTT-TGCCAAGGCTCTAACCCCTGAT**T**-3’
29	rs2908007	127	5'-GATGTTTTATAATCTAATTGGTGACTGACAACTAAGGTACGTTAGACCATTAGCAAAAGTAAATTAAGTGATAAGTATTATGGTAGGGACCTCAGGT***[A/G]***ATATAGGAATTGGGCAGAGAGAGACTTAA-3'
	Fw 29 primer	32	5’-GATGTTTTATAATCTAATTGGTGACTGACAAC-3
	Rev T primer	45	THIOL-C6-TTTTTTTTTTTTTTT-TTAAGTCTCTCTCTGCCCAATTCCTATAT**T**-3’
	Rev C primer	45	THIOL-C6-TTTTTTTTTTTTTTT-TTAAGTCTCTCTCTGCCCAATTCCTATAT**C**-3’
	Non-specific rev A primer	45	THIOL-C6-TTTTTTTTTTTTTTT-TTAAGTCTCTCTCTGCCCAATTCCTATAT**A**-3’
	Non-specific rev G primer	45	THIOL-C6-TTTTTTTTTTTTTTT-TTAAGTCTCTCTCTGCCCAATTCCTATAT**G**-3’
46	rs4635400	101	5'-CAATGAGACTTTCTAATAGGACTCATCTTGCCATTGGCACCCACAAAATCTAACCACCCACCTCCACTTCTCCGG***[A/G]***AGGATCCCTTCCTAATGTCTGATGC-3'
	Fw 46 primer	32	5’-CAATGAGACTTTCTAATAGGACTCATCTTGCC-3’

	Rev T primer	41	5’-THIOL-C6-TTTTTTTTTTTTTTT-GCATCAGACATTAGGAAGGGATCCT<u>**T**</u>-3’
	Rev C primer	41	5’-THIOL-C6-TTTTTTTTTTTTTTT-GCATCAGACATTAGGAAGGGATCCT<u>**C**</u>-3’
	Non-specific rev A primer	41	5’-THIOL-C6-TTTTTTTTTTTTTTT-GCATCAGACATTAGGAAGGGATCCT<u>**A**</u>-3’
	Non-specific rev G primer	41	5’-THIOL-C6-TTTTTTTTTTTTTTT-GCATCAGACATTAGGAAGGGATCCT<u>**G**</u>-3’
49	rs4988235	118	5'-CGTGGAATGCAGGGCTCAAAGAACAATCTAAAAATCAAACATTATACAAATGCAACCTAAGGAGGAGAGTTCCTTTGAGGCCAGGG<u>***[A/G]***</u>CTACATTATCTTATCTGTATT GCCAGCGCAG-3’
	Fw 49 primer	20	5’-CGTGGAATGCAGGGCTCAAA-3’
	Rev T primer	47	5’-THIOL-C6-TTTTTTTTTTTTTTT-CTGCGCTGGCAATACAGATAAGATAATGTAG<u>**T**</u>-3’
	Rev C primer	47	5’-THIOL-C6-TTTTTTTTTTTTTTT-CTGCGCTGGCAATACAGATAAGATAATGTAG<u>**C**</u>-3’
	Non-specific rev A primer	47	5’-THIOL-C6-TTTTTTTTTTTTTTT-CTGCGCTGGCAATACAGATAAGATAATGTAG<u>**A**</u>-3’
	Non-specific rev G primer	47	5’-THIOL-C6-TTTTTTTTTTTTTTT-CTGCGCTGGCAATACAGATAAGATAATGTAG<u>**G**</u>-3’

* The SNP region is underlined.

Table S2. TaqMan probes and the SNPs to be detected.

**rs Number**	**Assay ID****	**Context Sequence [VIC/FAM]**	**SNP Type**	**MA(F)***	**Gene**	**Gene Name**
rs10457487	C__30110289_10	CAAGCACTTACTGGGAAGGTCTACA[A/C]CTGCATAGGCAATGCTGGAAAAAGG	Transversion Substitution, Intragenetic	A (0,45)	RSPO3	R-spondin 3
rs2741856	C__16278893_20	ATTATAATTACTATGTTGGCTTCCA[G/C]ATCAGGGGTTAGAGCCTTGGCATGG	Transversion Substitution	G (0,08)	SOST	Sclerostin
rs2908007	C__15857734_20	AAGTATTATGGTAGGGACCTCAGGT[A/G]ATATAGGAATTGGGCAGAGAGAGAC	Transition Substitution	G (0,4)	WNT16	Wnt family member 16
rs4635400	C__28028819_10	TAACCACCCACCTCCACTTCTCCGG[A/G]AGGATCCCTTCCTAATGTCTGATGC	Transition Substitution	A (0,36)	RNMT/ FAM210A	RNA guanine-7 methyltransferase/ family with sequence similarity 210 member A
rs4988235	C___2104745_10	GAGGAGAGTTCCTTTGAGGCCAGGG[A/G]CTACATTATCTTATCTGTATTGCCA	Transition Substitution	C(0,49)	MCM6/ LCT-13910	minichromosome maintenance complex component 6

** Thermo Fisher Scientific

Table S3. Designed DNA sequences and primers for Sanger sequencing (primers are underlined and the SNP under interrogation is italicised).

SNP	Sequence ID	Length (bp)	Sequence
10	rs10457487	293	5’-<br>ATGTCCCCAGGTTTGAGACCTTTCGGATGATTTC<br>ATATACCATCTTTCTTCTGAGTGTTACCCAGTCA<br>AGTATAAGTAGCCAAATTATTTTGCACATCTTT<br>CTGTTTCTCATGTCTTCATTTATTCAACAAGCACT<br>TACTGGGAAGGTCTACA***[A/C]***CTGCATAGGCAAT<br>GCTGGAAAAAGGGTTAAGTAAACCAGGACATGA<br>CAATGGTGGCAAATGACTATCAGGTCTTCCCATG<br>TGTTTGACTCAAACTTATTACCCTATGGTCCTTCT<br>GACAATGGCAGAAGGTCTGAATC-3’
	Fw10 primer	22	5’-ATGTCCCCAGGTTTGAGACCTT-3’
	Rev10 primer	24	5’-GATTCAGACCTTCTGCCATTGTCA-3’
27	rs2741856	505	5’-<br>GTGAGCCATGATTGCTGCCACTGCACTCCAGTCT<br>GGGTGACAAAGCAAGACTTTGTCTCAAAAAAAA<br>CATGTATAGCTACATAATTAATAATATGCAAATA<br>TTCTTCTTTAGGGTCACTTAGGTTTTCAACATAC<br>AGACCCAAGAGAAGGTACAGAGACTCATTCTCT<br>CTCTCTTTCAATCACACACACACACACACACACA<br>CACACACACACACACACACACCCCTCTTCACTAT<br>AATTATAATTACTATGTTGGCTTCCA***[C/G]***ATCAG<br>GGGTTAGAGCCTTGGCATGGAGACGCCTGAAAG<br>GCACCCAAGGCAATTAGTGGTGTCCCTTCTCCAC<br>CCCCTACATACCTTCAGGCCCTTTGCACTTGCTG<br>CTCCCCCTCCCAGTGCACACACCCCCAGATCCTT<br>GTGTGGCTCCCTCTCCCACCACATTTTGGTCTTA<br>GCTCAAATGCCACCTCCTCAGAAATGCCTGGCCT<br>GGCCGCCTTCAGGGCTGTCTATATGCTCACCAGC<br>-3’

	Fw27 primer	22	5’-GTGAGCCATGATTGCTGCCACT-3’
	Rev27 primer	24	5’-GCTGGTGAGCATATAGACAGCCCT-3’
29	rs2908007	384	5’-AGCAAACTACCCTGGCACGTGTATACCTATGTAACAAACCTGCCCATTCTGCACACGTATCCCAGAACTTAAAGTATAATTTTAAAAAAGATGTTTTATAATCTAATTGGTGACTGACAACTAAGGTACGTTAGACCATTAGCAAAAGTAAATTAAGTGATAAGTATTATGGTAGGGACCTCAGGT***[A/G]***ATATAGGAATTGGGCAGAGAGAGACGTTAACATGGTTAACTCCAAAAGTGGTATGACCTAGAAAATGGGTAGATAGGTTTGAGTAAGTGGAAGGAGAGAAAAGAAATTTCTAGGCATAGACGCAGTGTTTCTCAAGAAACTGAAAGACAAATATGCAGGTCCTGCTTATGGAAAAGGGAAGACAGAAGGTGTCTTGC-3’
	Fw29 primer	20	5’-AGCAAACTACCCTGGCACGT-3’
	Rev29 primer	21	5’-GCAAGACACCTTCTGTCTTCC-3’
46	rs4635400	551	5’-CAGATAGCAAGGAAGAGTTACTATAATTCATGCTTACATTAAAAATCAAGAAATCCAAATAAAATACCACGTTTAATTCAATTTATCACGTCCAAGAAGTTACAAAGGCAATGTAGAAGAAGTAAGTAAAAATGGGGAAGCCCTAATTTTTAAGTCTGCCTTGAAAACAATTTTTTCTTATCAATGAGACTTTCTAATAGGACTCATCTTGCCATTGGCACCCACAAAATCTAACCACCCACCTCCACTTCTCCGG***[A/G]***AGGATCCCTTCCTAATGTCTGATGCCTCTTCCCTCACCTCCATTCTTTGCCCACTGCTGCACATGCTTAAAGCCCAGCCACCCTTTCACCCACAGAGGTCATAGACCACTGCTACTATGGTTAATTCTCAGAAGTAATAAACTCAGAACCTGAAAGCACATAATGCCTACAAGACTGTTCACTATTTCAAACTTCTGCTCTGGTTTACATGAATTAAAAAAAAAAAAAATCACTCTATTTTTTCTGCT

			GTACCAAGGCAAAAAAATTCTACAATCACATTT<br>AAAGGCATGGACT-3’
	Fw46 primer	21	5’-CAGATAGCAAGGAAGAGTTAC-3’
	Rev46 primer	20	5’-AGTCCATGCCTTTAAATGTG-3’
49	rs4988235	439	5’-<br>CAAAGACGACCTTACATCAAACCTATTAATAAA<br>ACTAGGAAAACGCAGGGCTGCTTTGGTTGAAGC<br>GAAGATGGGACGCTTGAATGCCCTTTCGTACTAC<br>TCCCCTTTTACCTCGTTAATACCCACTGACCTATC<br>CTCGTGGAATGCAGGGCTCAAAGAACAATCTAA<br>AAATCAAACATTATACAAATGCAACCTAAGGAG<br>GAGAGTTCCTTTGAGGCCAGGG***[A/G]***CTACATTA<br>TCTTATCTGTATTGCCAGCGCAGAGGCCTACTAG<br>TACATTGTAGGGTCTAAGTACATTTTTCCTGAAT<br>GAAAGGTATTAAATGGTAACTTACGTCTTTATGC<br>ACTCTATAAACTATGACGTGATCGTCTCCGTCTA<br>ACAACTACACTCAAATGCTTACCAAGCTCTTTAA<br>AGGGAAGAATTCCATGGTCGTATGAGCATTCAA<br>CAGT-3’
	Fw49 primer	22	5’-CAAAGACGACCTTACATCAAAC-3’
	Rev49 primer	21	5’-ACTGTTGAATGCTCATACGAC-3’

* The primer region is underlined and the SNP region is in italics.

3. Figures and Captions

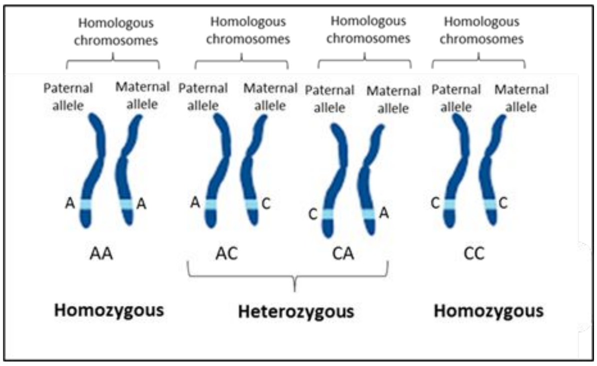

Figure S1. Schematic representation of the SNP expected in a diploid organism.

SNP 10

Products on potentially unintended templates

>NM_032784.5 Homo sapiens R-spondin 3 (RSPO3), mRNA
product length = 124
Forward primer 1 GTGTTACCCAGTCAAGTATAAGTAGCCAA 29
Template 3323 3351
Reverse primer 1 TTTTTCCAGCATTGCCTATGCAG 23
Template 3446 3424

>AL590733.8 Human DNA sequence from clone RP11-193D23 on chromosome 6, complete sequence
product length = 124
Forward primer 1 GTGTTACCCAGTCAAGTATAAGTAGCCAA 29
Template 67738 67766
Reverse primer 1 TTTTTCCAGCATTGCCTATGCAG 23
Template 67861 67839

SNP 27

Products on potentially unintended templates

>NC_000017.11 Homo sapiens chromosome 17, GRCh38.p14 Primary Assembly
product length = 107
Forward primer 1 TTCAATCACACACACACACACACAC 25
Template 43749387 A...CA................... 43749411
Reverse primer 1 TGCCAAGGCTCTAACCCCTGAT 22
Template 43749493 43749472

SNP 29

Products on potentially unintended templates

>NG_029242.1 Homo sapiens Wnt family member 16 (WNT16), RefSeqGene on chromosome 7
product length = 127
Forward primer 1 GATGTTTTATAATCTAATTGGTGACTGACAAC 32
Template 1647 1678
Reverse primer 1 TTAAGTCTCTCTCTGCCCAATTCCTATAT 29
Template 1773 .A.C.......................... 1745

>AC006364.4 Homo sapiens BAC clone GS1-146J4 from 7, complete sequence
product length = 127
Forward primer 1 GATGTTTTATAATCTAATTGGTGACTGACAAC 32
Template 59651 59682
Reverse primer 1 TTAAGTCTCTCTCTGCCCAATTCCTATAT 29
Template 59777 .A.C.......................... 59749

SNP 46

Products on potentially unintended templates

>CP068260.2 Homo sapiens isolate CHM13 chromosome 18
product length = 101
Forward primer 1 CAATGAGACTTTCTAATAGGACTCATCTTGCC 32
Template 13881754 13881785
Reverse primer 1 GCATCAGACATTAGGAAGGGATCCT 25
Template 13881854 13881830

>AP023478.1 Homo sapiens DNA, chromosome 18, nearly complete genome
product length = 101
Forward primer 1 CAATGAGACTTTCTAATAGGACTCATCTTGCC 32
Template 13729456 13729487
Reverse primer 1 GCATCAGACATTAGGAAGGGATCCT 25
Template 13729556 13729532

Template 13709535 13709511

>AP001525.6 Homo sapiens genomic DNA, chromosome 18 clone:RP11-720L3, complete sequence
product length = 101
Forward primer 1 CAATGAGACTTTCTAATAGGACTCATCTTGCC 32
Template 57167 57198
Reverse primer 1 GCATCAGACATTAGGAAGGGATCCT 25
Template 57267 57243

SNP 49

Products on potentially unintended templates

>MZ362856.1 Homo sapiens isolate SC18 – SC01 minichromosome maintenance deficient 6 (MCM6) gene, intron 13
product length = 118
Forward primer 1 CGTGGAATGCAGGGCTCAAA 20
Template 294 313
Reverse primer 1 CTGCGCTGGCAATACAGATAAGATAATGTAG 31
Template 411 381

>MZ362838.1 Homo sapiens isolate PR99-PR01 minichromosome maintenance deficient 6 (MCM6) gene, intron 13
product length = 118
Forward primer 1 CGTGGAATGCAGGGCTCAAA 20
Template 300 319
Reverse primer 1 CTGCGCTGGCAATACAGATAAGATAATGTAG 31
Template 417 387

>KU661853.1 Homo sapiens isolate MOZ_VBP-0018 MCM6 gene, intron
product length = 118
Forward primer 1 CGTGGAATGCAGGGCTCAAA 20
Template 260 241
Reverse primer 1 CTGCGCTGGCAATACAGATAAGATAATGTAG 31
Template 143 173

>KU661772.1 Homo sapiens isolate ANGH99 MCM6 gene, intron
product length = 118
Forward primer 1 CGTGGAATGCAGGGCTCAAA 20
Template 260 241
Reverse primer 1 CTGCGCTGGCAATACAGATAAGATAATGTAG 31
Template 143 173

>JQ395134.1 Homo sapiens isolate N_Tib2810 MCM6 gene, intron 13
product length = 118
Forward primer 1 CGTGGAATGCAGGGCTCAAA 20
Template 221 202
Reverse primer 1 CTGCGCTGGCAATACAGATAAGATAATGTAG 31
Template 104 134

>JQ395073.1 Homo sapiens isolate N_Tib2749 MCM6 gene, intron 13
product length = 118
Forward primer 1 CGTGGAATGCAGGGCTCAAA 20
Template 221 202
Reverse primer 1 CTGCGCTGGCAATACAGATAAGATAATGTAG 31
Template 104 134

>NG_008104.2 Homo sapiens lactase (LCT), RefSeqGene (LRG_338) on chromosome 2
product length = 118
Forward primer 1 CGTGGAATGCAGGGCTCAAA 20
Template 9180 9161
Reverse primer 1 CTGCGCTGGCAATACAGATAAGATAATGTAG 31
Template 9063 9093

>AC238958.2 Homo sapiens FOSMID clone ABC16-1709C3 from chromosome 2, complete sequence
product length = 118
Forward primer 1 CGTGGAATGCAGGGCTCAAA 20
Template 7101 7082
Reverse primer 1 CTGCGCTGGCAATACAGATAAGATAATGTAG 31
Template 6984 7014

>NG_008958.1 Homo sapiens minichromosome maintenance complex component 6 (MCM6), RefSeqGene on chromosome 2
product length = 118
Forward primer 1 CGTGGAATGCAGGGCTCAAA 20
Template 30452 30433
Reverse primer 1 CTGCGCTGGCAATACAGATAAGATAATGTAG 31
Template 30335 30365

>AY220757.1 Homo sapiens MCM6 minichromosome maintenance deficient 6 (MIS5 homolog, S. pombe) (S. cerevisiae) (MCM6) gene, complete cds
product length = 118
Forward primer 1 CGTGGAATGCAGGGCTCAAA 20
Template 26724 26705
Reverse primer 1 CTGCGCTGGCAATACAGATAAGATAATGTAG 31
Template 26607 26637

>AC011893.7 Homo sapiens BAC clone RP11-34L23 from 2, complete sequence
product length = 118
Forward primer 1 CGTGGAATGCAGGGCTCAAA 20
Template 155009 155028
Reverse primer 1 CTGCGCTGGCAATACAGATAAGATAATGTAG 31
Template 155126 155096

>JQ395159.1 Homo sapiens isolate N_Tib2835 MCM6 gene, intron 13
product length = 118
Forward primer 1   CGTGGAATGCAGGGCTCAAA 20
Template   221 ....T..... 202
Reverse primer 1   CTGCGCTGGCAATACAGATAAGATAATGTAG 31
Template   104 134

>JQ395072.1 Homo sapiens isolate N_Tib2748 MCM6 gene, intron 13
product length = 118
Forward primer 1   CGTGGAATGCAGGGCTCAAA 20
Template   221 ....Y..... 202
Reverse primer 1   CTGCGCTGGCAATACAGATAAGATAATGTAG 31
Template   104 134

Figure S2. Primer specificity evaluation using *Primer Blast* and nucleotide collection parameter for each set of primers.

All Forward primer with Reverse 10 primer

Name	Sequence	Tm°C	CG%	nt	A	T	C
Fw10	gtgttacccagtcaagtataagtagccaa	66.0	41.4	29	10.0	7.0	6.0
Fw27	ttcaatcacacacacacacacacaca	70.8	42.3	26	12.0	3.0	11.0
Fw29	gatgttttataatctaattggtgactgacaac	65.3	31.3	32	10.0	12.0	4.0
Fw46	caatgagactttctaataggactcatcttgcc	69.7	40.6	32	9.0	10.0	8.0
Fw49	cgtggaatgcagggctcaaa	70.7	55.0	20	6.0	3.0	4.0
Rev10	tttttccagcattgcctatgcag	69.0	43.5	23	4.0	9.0	6.0

Self-Dimers:

Cross Primer Dimers:

All Forward primer with Reverse 27 primer

Name	Sequence	Tm°C	CG%	nt	A	T	C
Fw10	gtgttacccagtcaagtataagtagccaa	66.0	41.4	29	10.0	7.0	6.0
Fw27	ttcaatcacacacacacacacacaca	70.8	42.3	26	12.0	3.0	11.0
Fw29	gatgttttataatctaattggtgactgacaac	65.3	31.3	32	10.0	12.0	4.0
Fw46	caatgagactttctaataggactcatcttgcc	69.7	40.6	32	9.0	10.0	8.0
Fw49	cgtggaatgcagggctcaaa	70.7	55.0	20	6.0	3.0	4.0
Rev27	tgccaaggctctaacccctgat	70.0	54.5	22	5.0	5.0	8.0

Self-Dimers:

Cross Primer Dimers:

All Forward primer with Reverse 29 primer

Name	Sequence	Tm°C	CG%	nt	A	T	C
Fw10	gtgttacccagtcaagtataagtagccaa	66.0	41.4	29	10.0	7.0	6.0
Fw27	ttcaatcacacacacacacacacaca	70.8	42.3	26	12.0	3.0	11.0
Fw29	gatgttttataatctaattggtgactgacaac	65.3	31.3	32	10.0	12.0	4.0
Fw46	caatgagactttctaataggactcatcttgcc	69.7	40.6	32	9.0	10.0	8.0
Fw49	cgtggaatgcagggctcaaa	70.7	55.0	20	6.0	3.0	4.0
Rev29	ttaagtctctctctgcccaattcctatat	65.9	37.9	29	6.0	12.0	9.0

Self-Dimers:

Cross Primer Dimers:

All Forward primer with Reverse 46 primer

Name	Sequence	Tm°C	CG%	nt	A	T	C
Fw10	gtgttacccagtcaagtataagtagccaa	66.0	41.4	29	10.0	7.0	6.0
Fw27	ttcaatcacacacacacacacacaca	70.8	42.3	26	12.0	3.0	11.0
Fw29	gatgttttataatctaattggtgactgacaac	65.3	31.3	32	10.0	12.0	4.0
Fw46	caatgagactttctaataggactcatcttgcc	69.7	40.6	32	9.0	10.0	8.0
Fw49	cgtggaatgcagggctcaaa	70.7	55.0	20	6.0	3.0	4.0
Rev46	gcatcagacattaggaagggatcct	68.3	48.0	25	8.0	5.0	5.0

Self-Dimers:

Cross Primer Dimers:

All Forward primer with Reverse 49 primer

Name	Sequence	Tm°C	CG%	nt	A	T	C
Fw10	gtgttacccagtcaagtataagtagccaa	66.0	41.4	29	10.0	7.0	6.0
Fw27	ttcaatcacacacacacacacacaca	70.8	42.3	26	12.0	3.0	11.0
Fw29	gatgttttataatctaattggtgactgacaac	65.3	31.3	32	10.0	12.0	4.0
Fw46	caatgagactttctaataggactcatcttgcc	69.7	40.6	32	9.0	10.0	8.0
Fw49	cgtggaatgcagggctcaaa	70.7	55.0	20	6.0	3.0	4.0
Rev49	ctgcgctggcaatacagataagataatgtag	70.0	41.9	31	11.0	7.0	5.0

Self-Dimers:

Cross Primer Dimers:

Figure S3. Self-dimers and primer-dimers checked by Multiple Primer Analyzer software. All the forward primers were tested again each reverse primer.

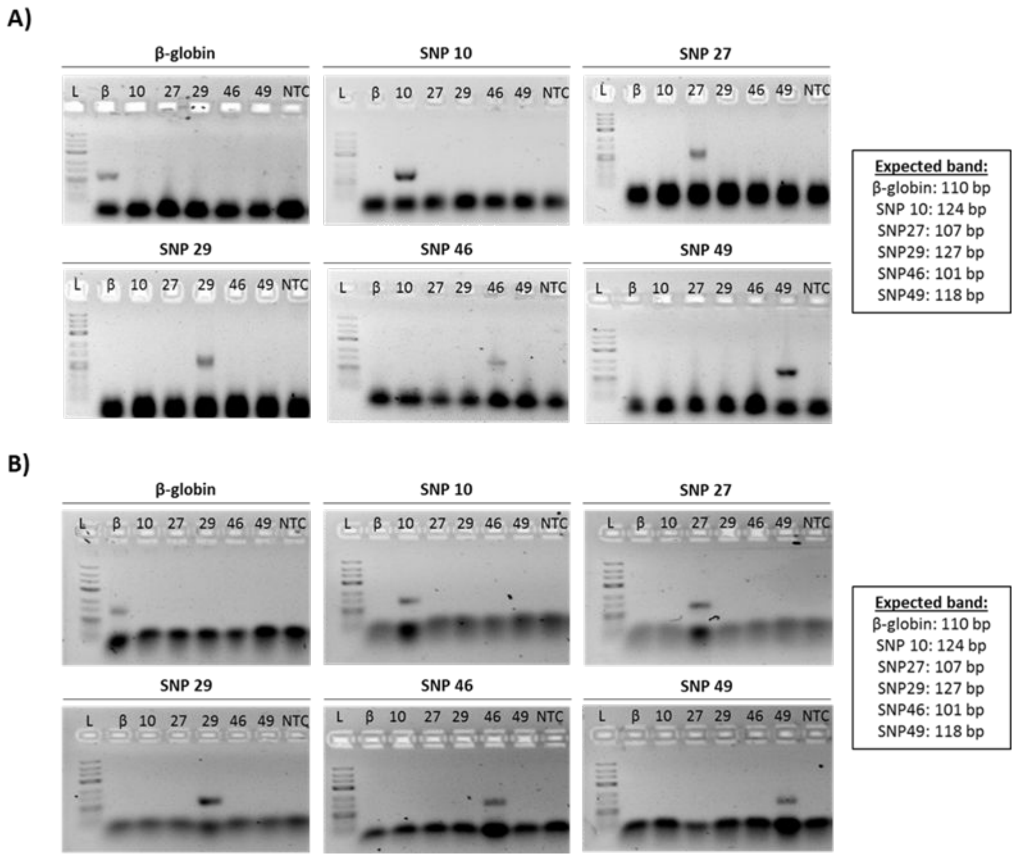

Figure S4. Agarose gel demonstrating the correct design and specificity of all the SNPs sequences by (A) PCR and (B) Liquid-phase RPA.

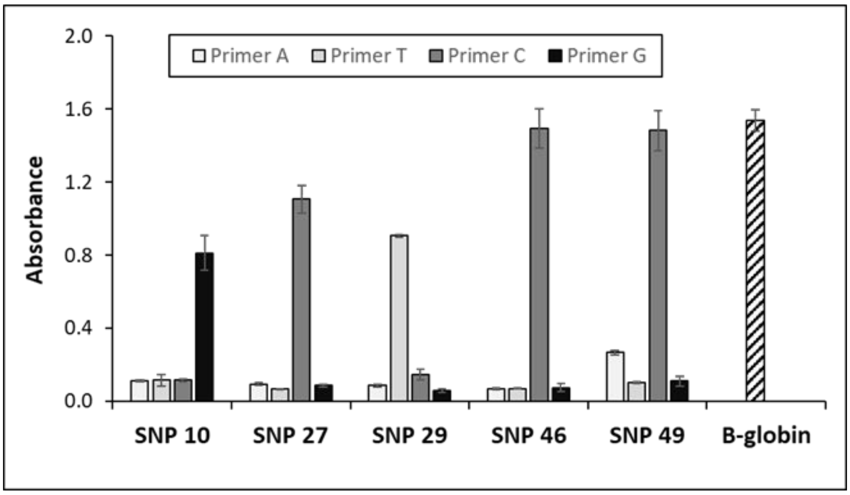

Figure S5. Solid phase RPA (15 min at 37^oC) for simultaneous detection of 5 SNP related to osteoporosis on maleimide activated plate and colorimetric detection using synthetic targets (Table S1). β-globin was used as positive control of the reaction.

A)

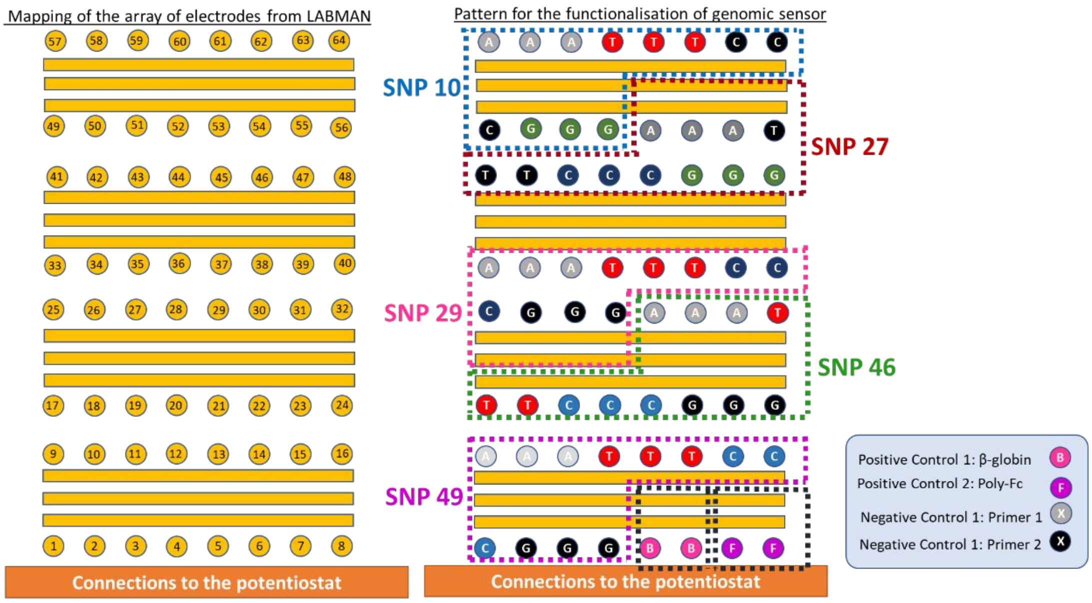

B)

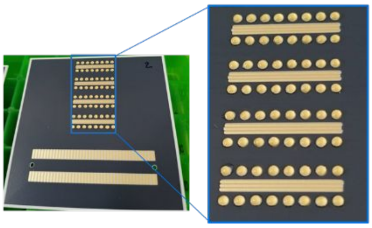

Figure S6. A) Mapping of electrode array provided by LABMAN and the pattern of electrode array functionalisation. The SNP related primers are highlighted in different colours (red for primers ending in T for SNPs A, blue for primers ending in C for SNPs G and green for primers ending in G for SNPs C) while the negative control primers are represented in grey and black colours). Finally, the positive controls (β-globin and Poly-Fc) are in pink and magenta colours. B) Real picture of electrode array during functionalisation process containing the drops over the electrodes.

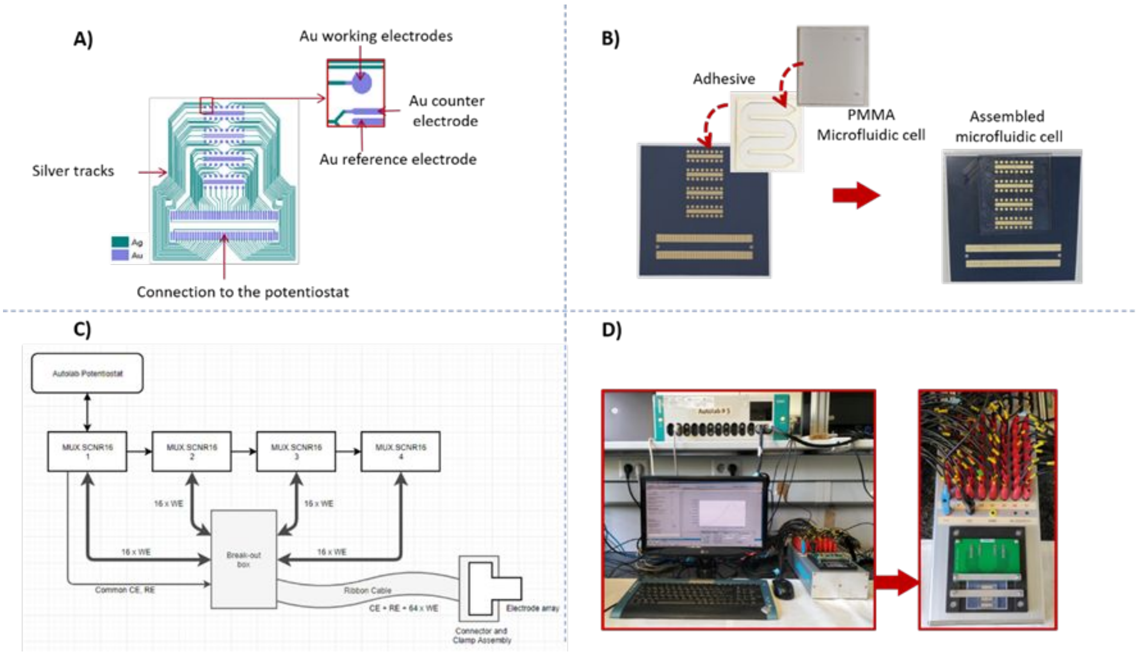

Figure S7. A) AUTOCAD view of the electrode array. B) Real picture of the electrode array, double adhesive gasket and PMMA. C) Schematic representation of the 64 break-out box connected to the potentionstat and electrode array. D) Laboratory set-up used for genomic sensor validation and vertical view of the electrode array inside the microfluidic cell and connected to the potentiostat.

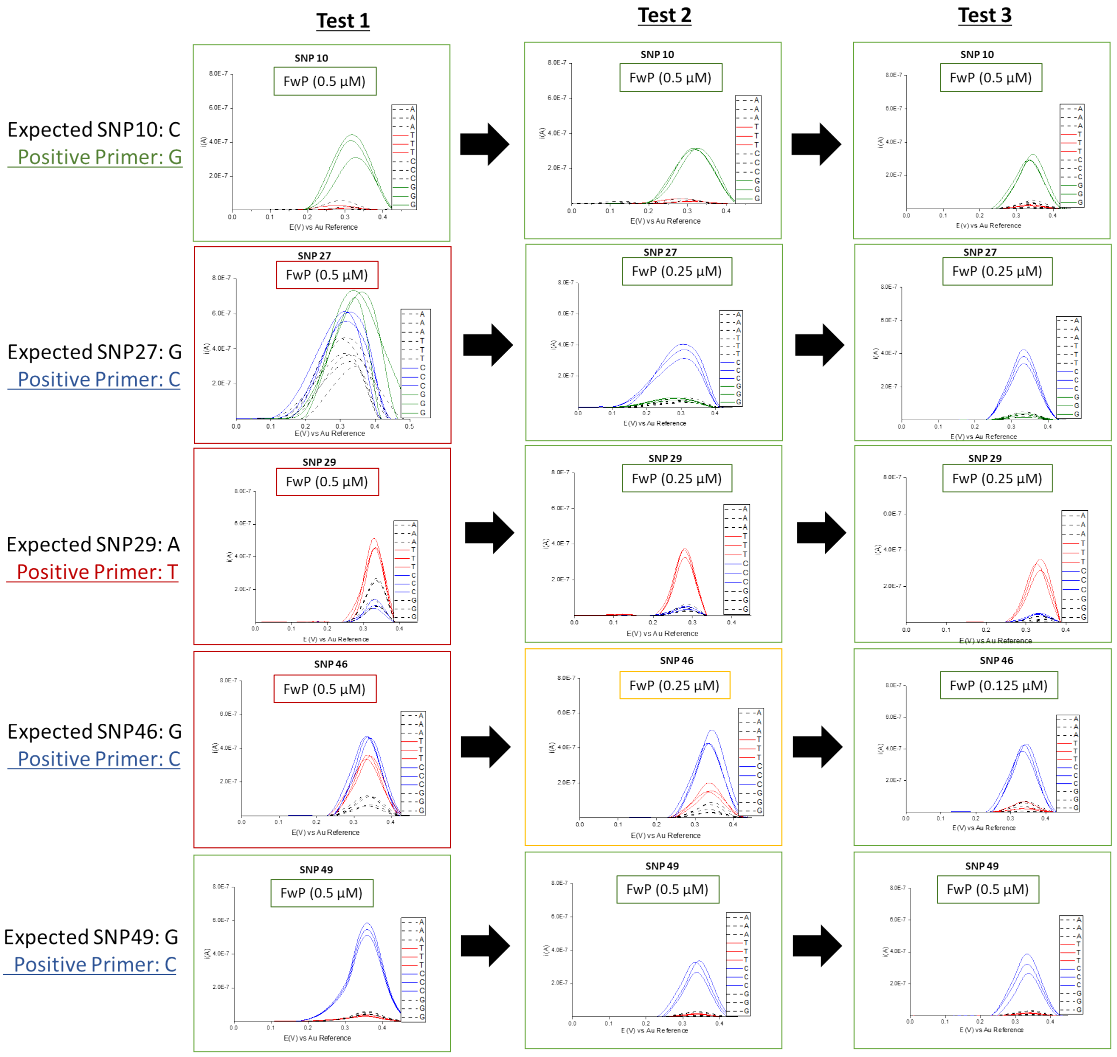

Figure S8. SWVs obtained during the flow-through of the optimisation of the concentration of the five Forward Primers (FwP) for simultaneous detection of the five SNPs related to osteoporosis using solid phase RPA, $dN^{Fc}TPs$ and electrochemical detection. The targets were prepared from synthetic sequences from Table S1.

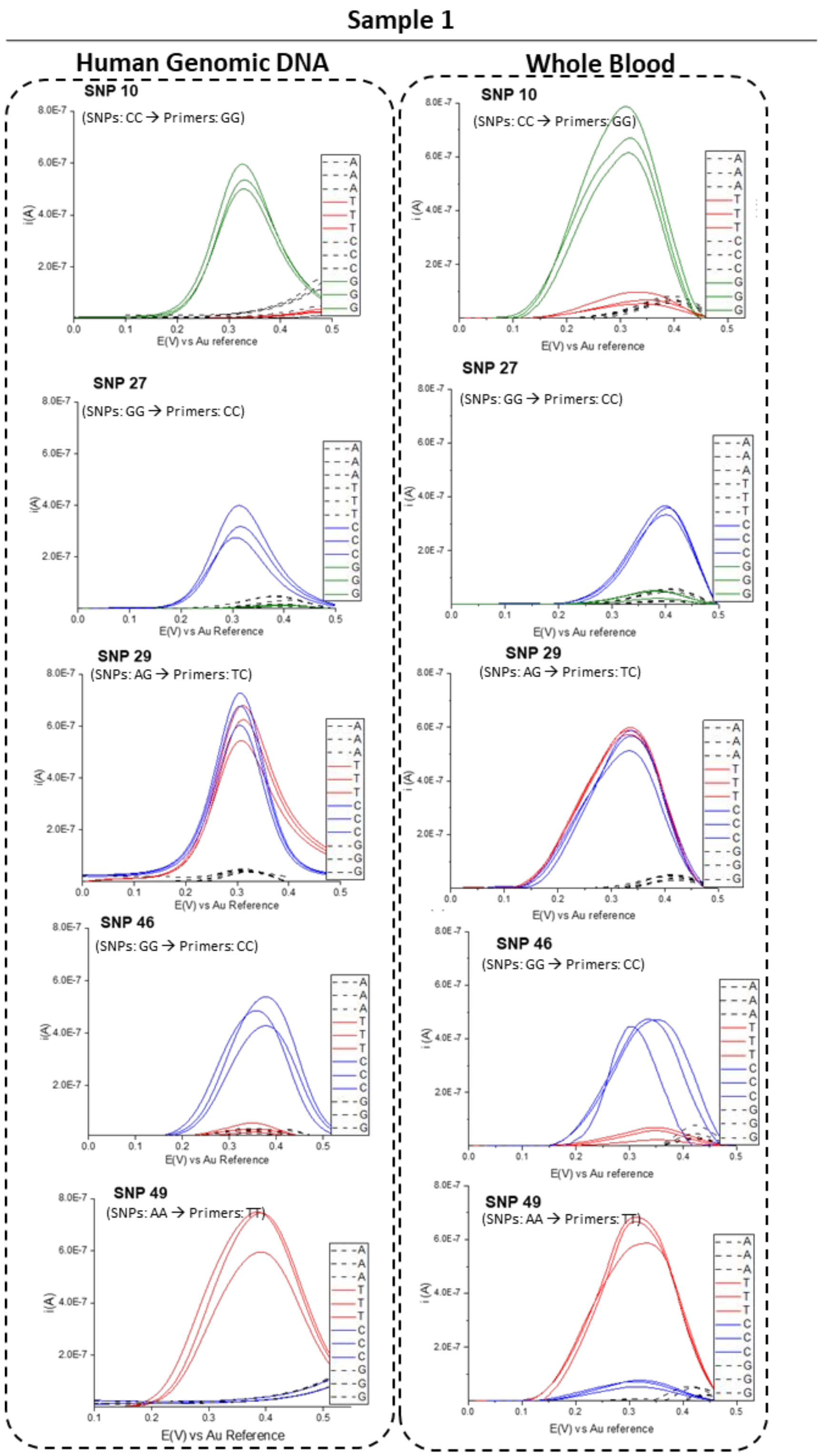
Sample 1
Human Genomic DNA
Whole Blood
SNP 10
(SNPs: CC → Primers: GG)
SNP 27
(SNPs: GG → Primers: CC)
SNP 29
(SNPs: AG → Primers: TC)
SNP 46
(SNPs: GG → Primers: CC)
SNP 49
(SNPs: AA → Primers: TT)
i (A)
E(V) vs Au reference
E(V) vs Au Reference

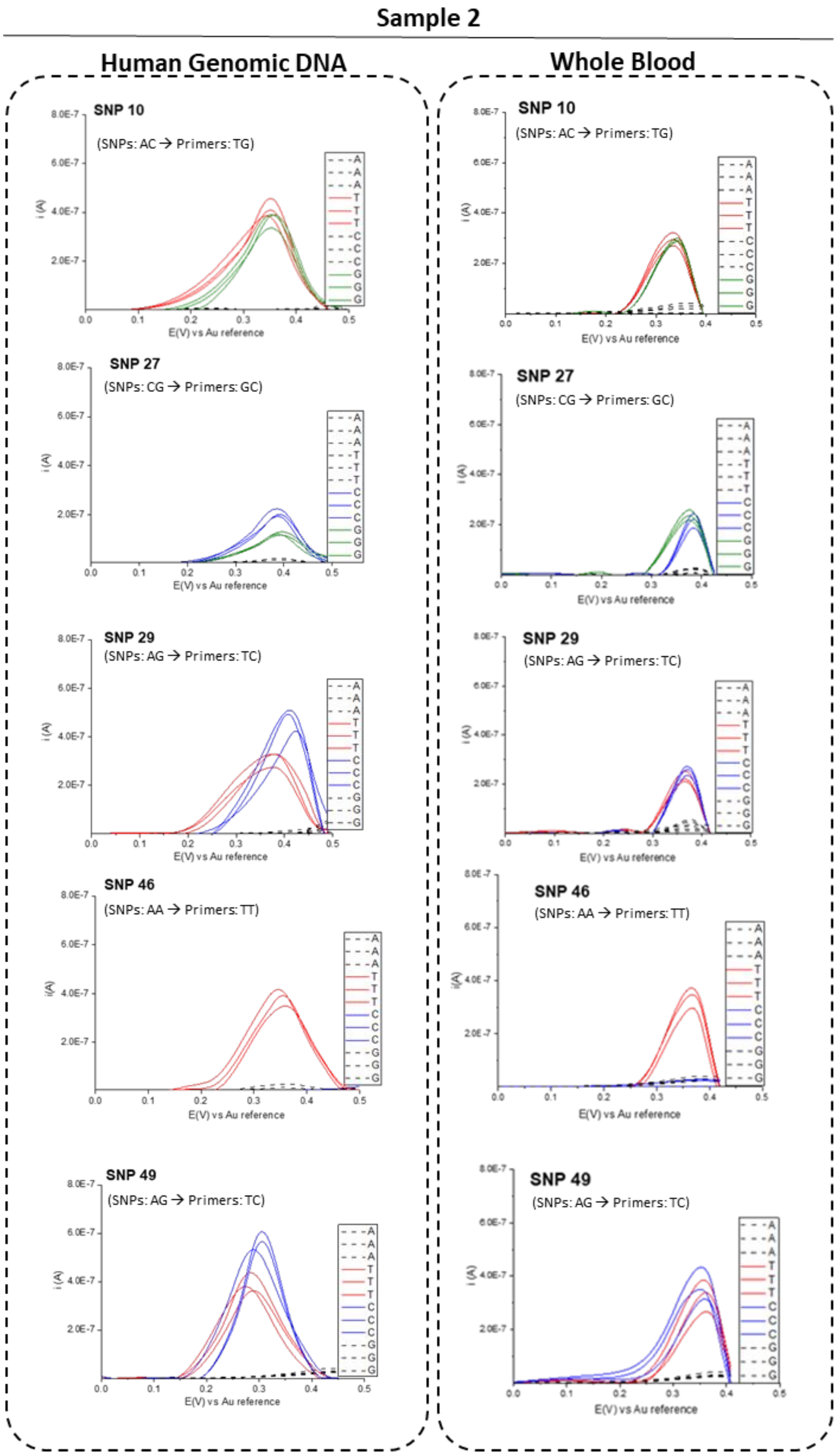
Sample 2
Human Genomic DNA
Whole Blood
SNP 10
(SNPs: AC → Primers: TG)
SNP 27
(SNPs: CG → Primers: GC)
SNP 29
(SNPs: AG → Primers: TC)
SNP 46
(SNPs: AA → Primers: TT)
SNP 49
(SNPs: AG → Primers: TC)
i (A)
E(V) vs Au reference
8.0E-7
6.0E-7
4.0E-7
2.0E-7
0.0
0.1
0.2
0.3
0.4
0.5
A
T
C
G

Sample 3

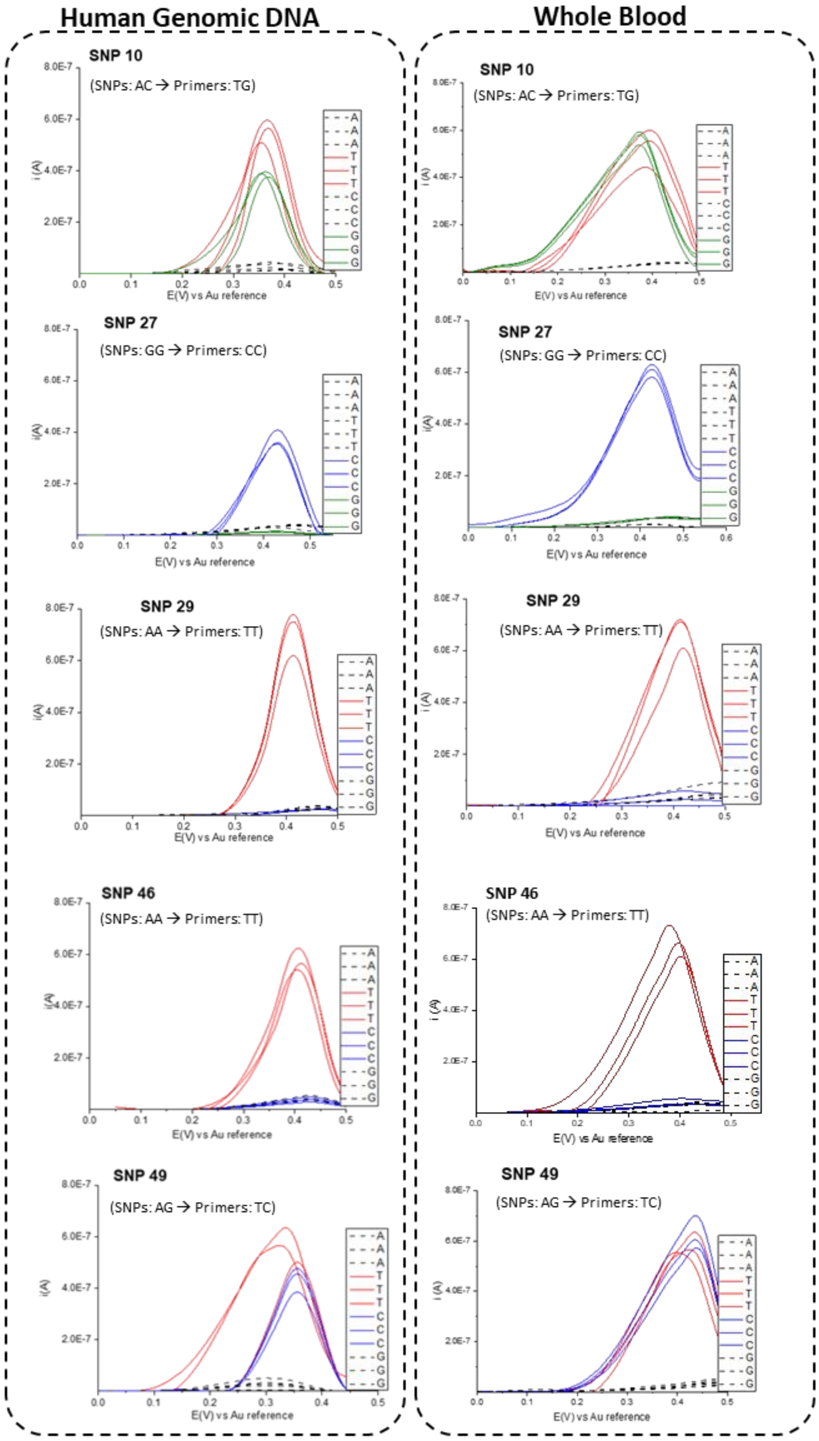
Human Genomic DNA
Whole Blood
SNP 10
(SNPs: AC → Primers: TG)
SNP 27
(SNPs: GG → Primers: CC)
SNP 29
(SNPs: AA → Primers: TT)
SNP 46
(SNPs: AA → Primers: TT)
SNP 49
(SNPs: AG → Primers: TC)
i (A)
E(V) vs Au reference

Sample 4

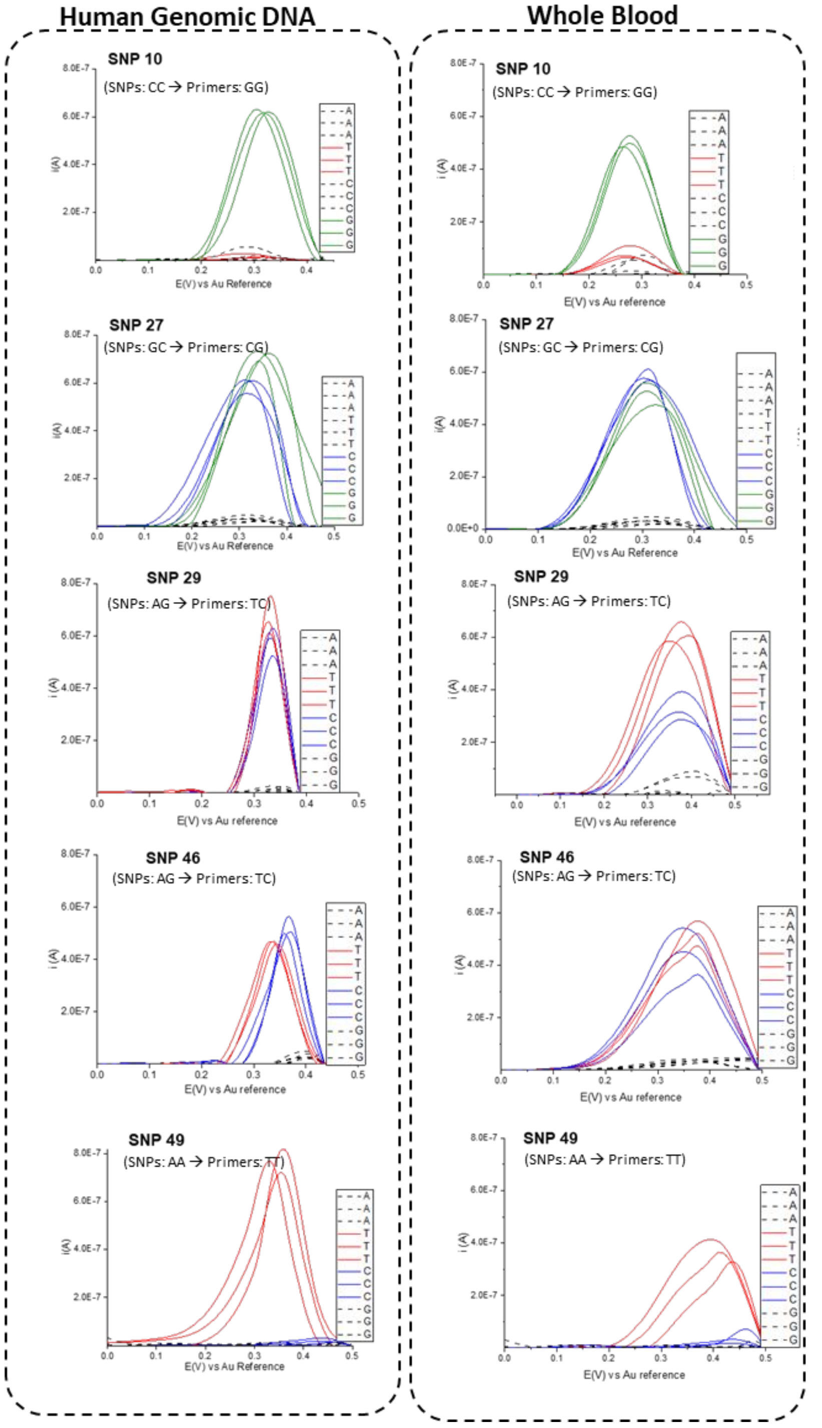

Human Genomic DNA
Whole Blood
SNP 10
(SNPs: CC → Primers: GG)
SNP 27
(SNPs: GC → Primers: CG)
SNP 29
(SNPs: AG → Primers: TC)
SNP 46
(SNPs: AG → Primers: TC)
SNP 49
(SNPs: AA → Primers: TT)
E(V) vs Au Reference
E(V) vs Au reference
i (A)

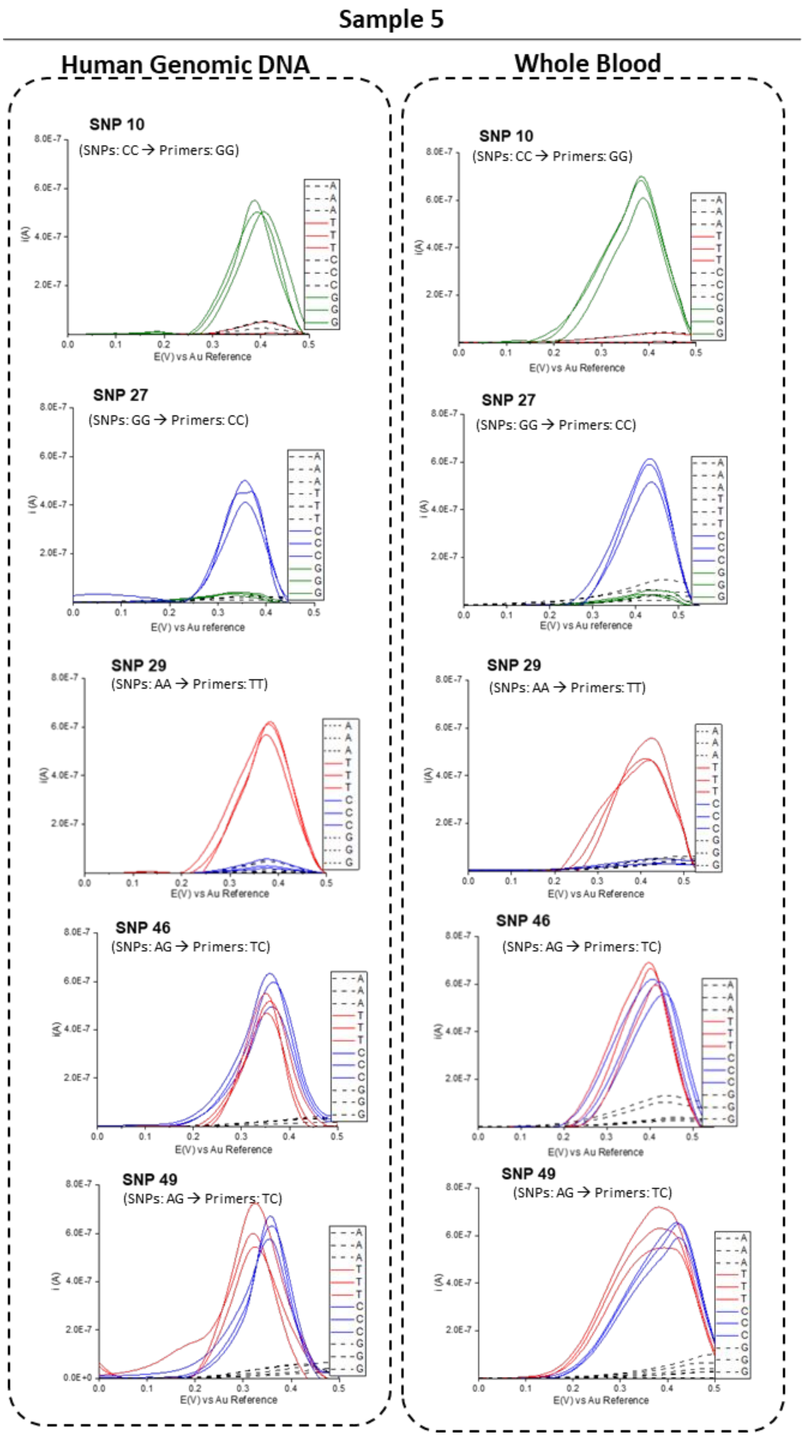

Figure S9. SWV recorded in 0.1 M $Sr(NO_3)_2$ + 0.1 M Glycine pH 3 for the simultaneous electrochemical detection of 5 SNPs in 5 real samples: (left column) with the genomic DNA previously extracted and purified, and (right column) using the human whole blood, to evaluate the matrix effect. The SNP related primers are highlighted in different colours (red for primers ended in T for SNPs A, blue for primers ended in C for SNPs G and green for primers ended in G for SNPs C) while the negative primers are represented black colour and discontinuous traces.

Sample 6

Human Whole blood

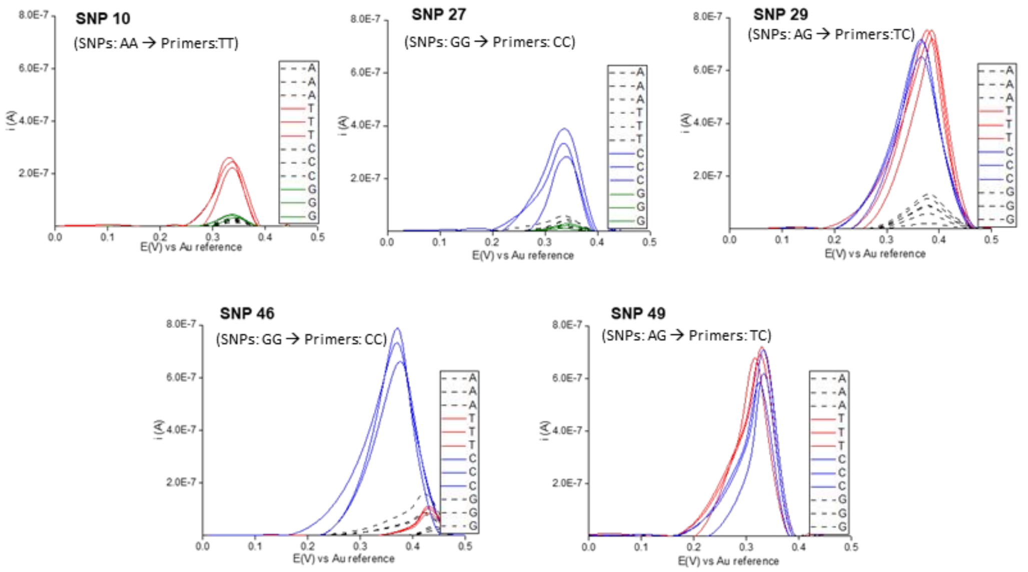

Sample 7

Human Whole blood

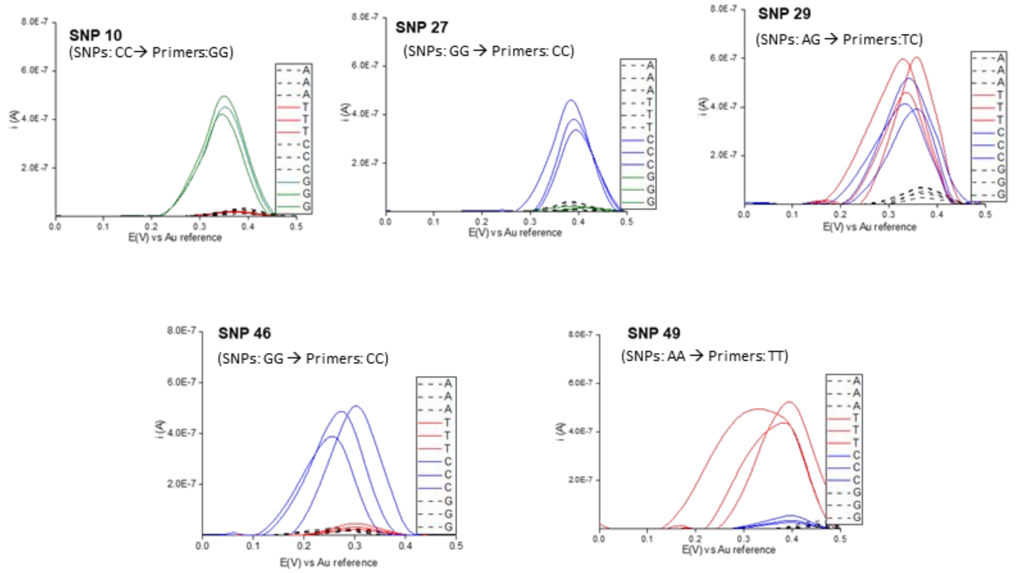

Sample 8

Human Whole blood

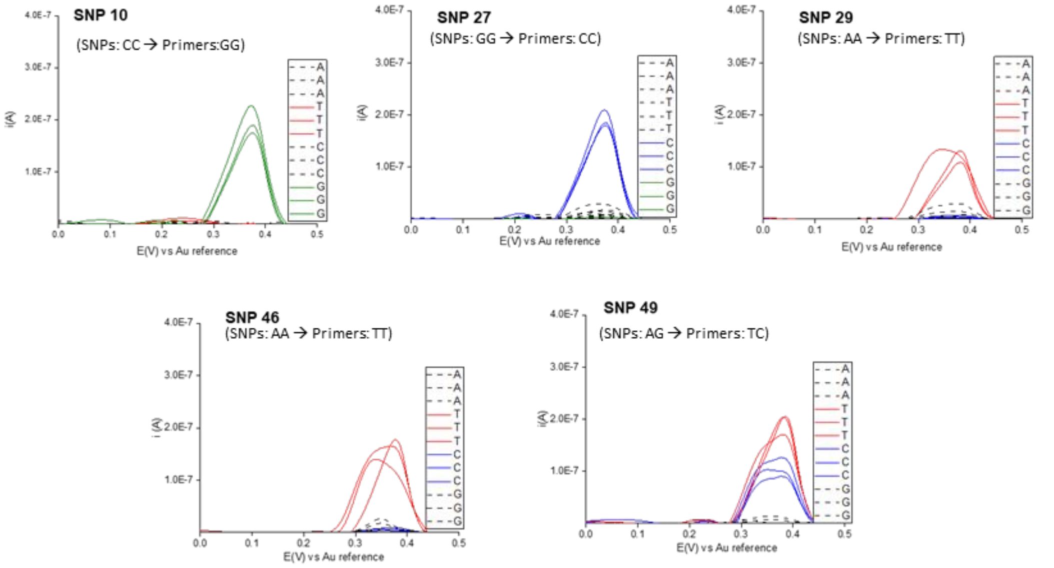

Sample 9

Human Whole blood

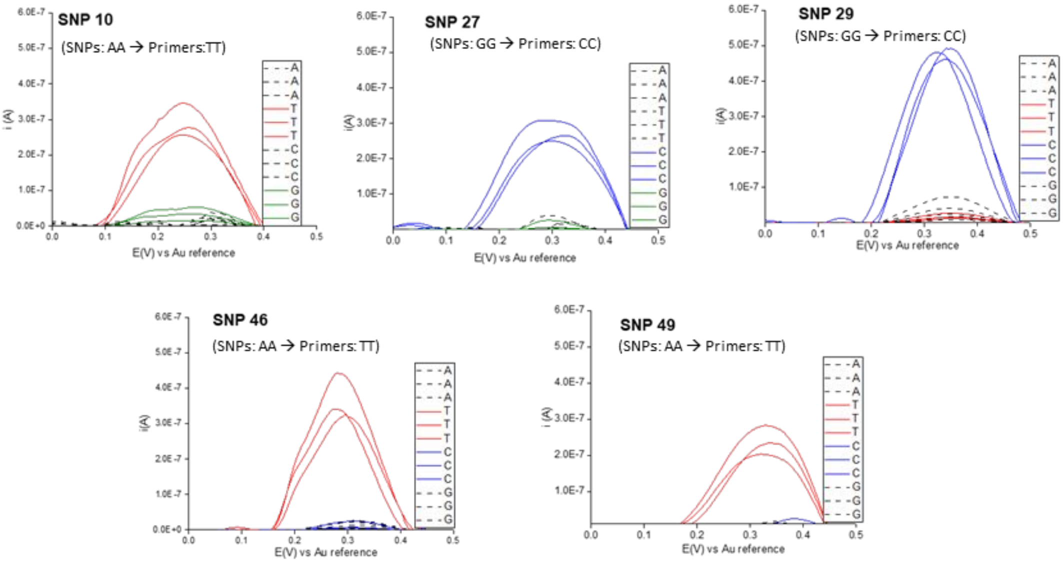

Sample 10

Human Whole blood

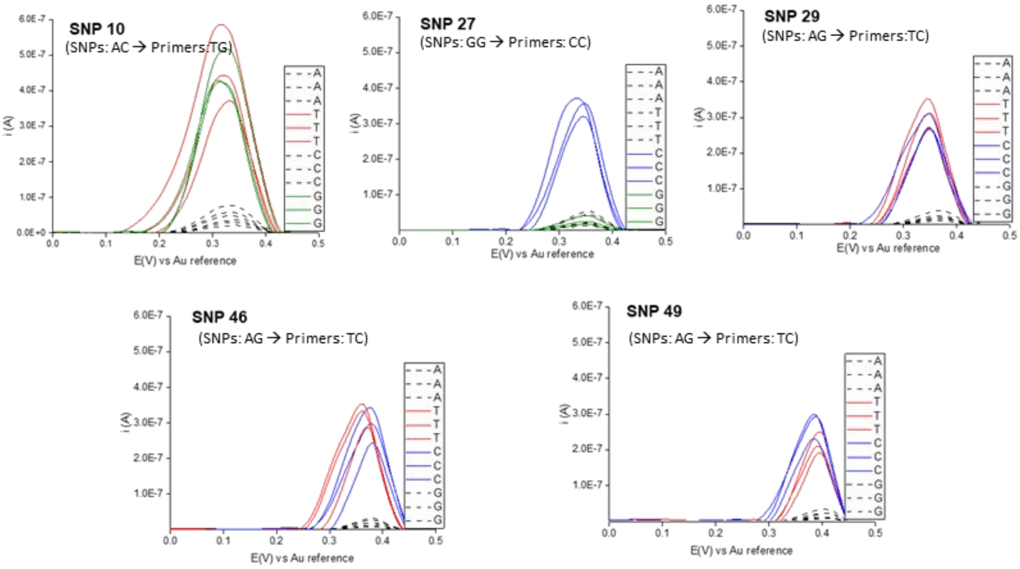

Sample 11

Human Whole blood

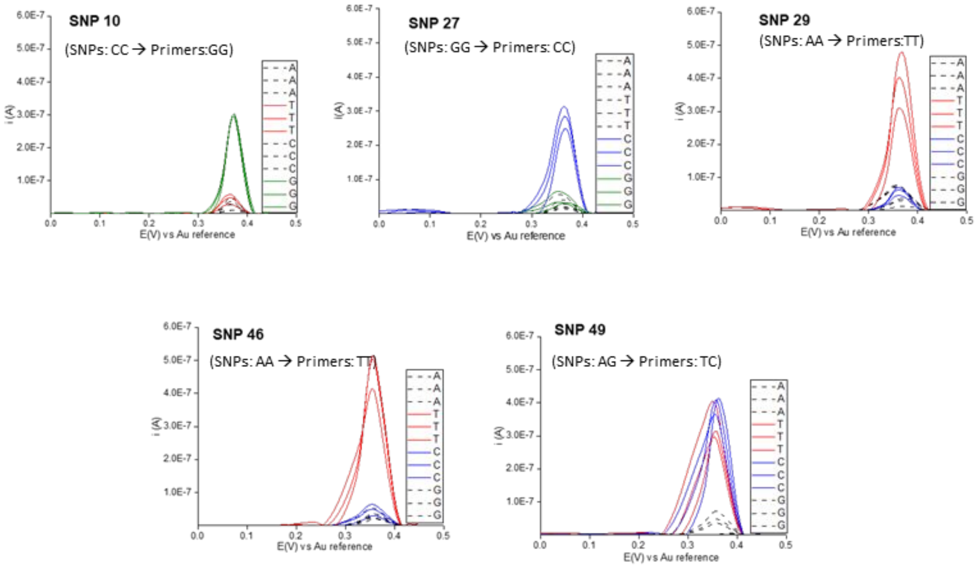

Sample 12

Human Whole blood

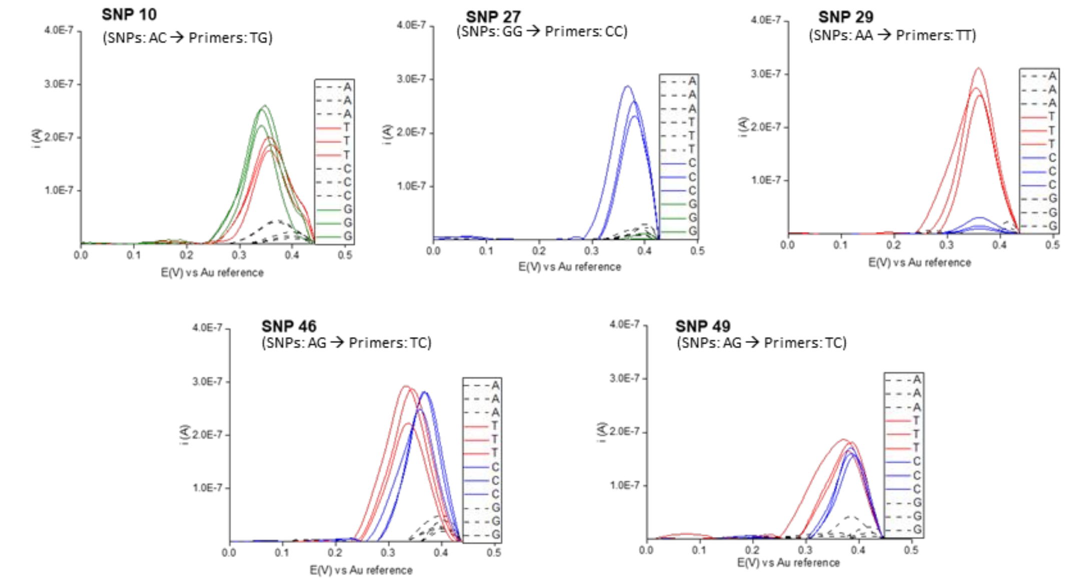

Sample 13

Human Whole blood

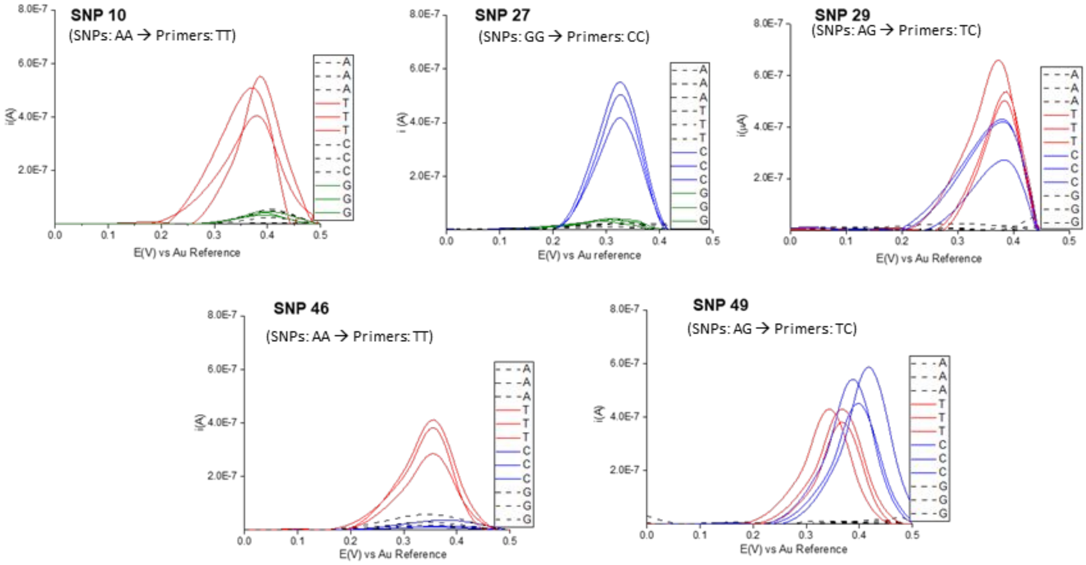

Sample 14

Human Whole blood

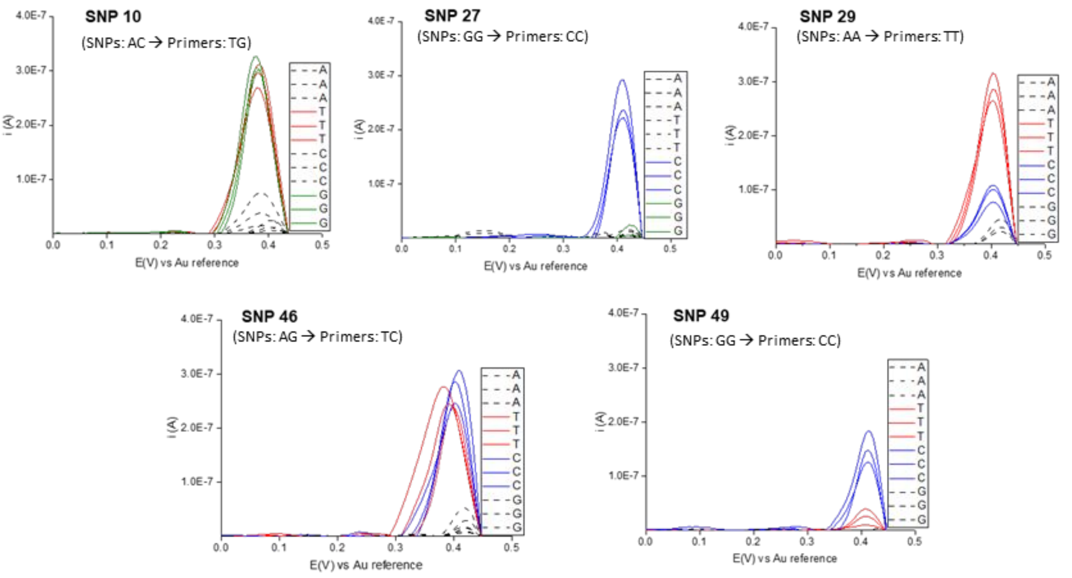

Sample 15

Human Whole blood

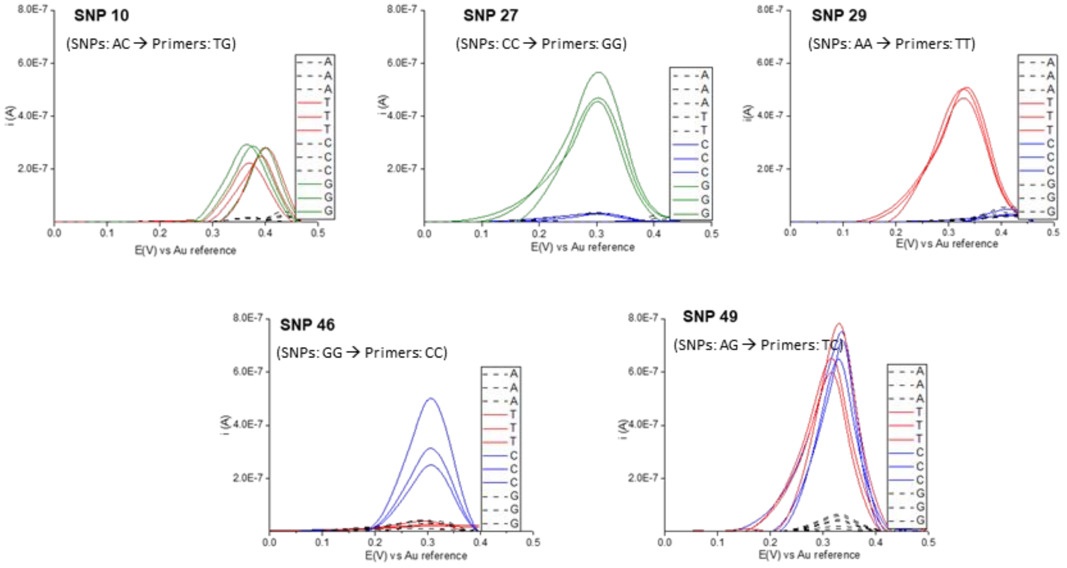

Figure S10. SWV recorded in 0.1 M $Sr(NO_3)_2$ + 0.1 M Glycine pH 3 for simultaneous electrochemical detection of 5 SNPs for 10 more human whole blood samples. The SNP related primers are highlighted in different colours (red for primers ended in T for SNPs A, blue for primers ended in C for SNPs G and green for primers ended in G for SNPs C) while the negative primers are represented black colour and discontinuous traces.

A)

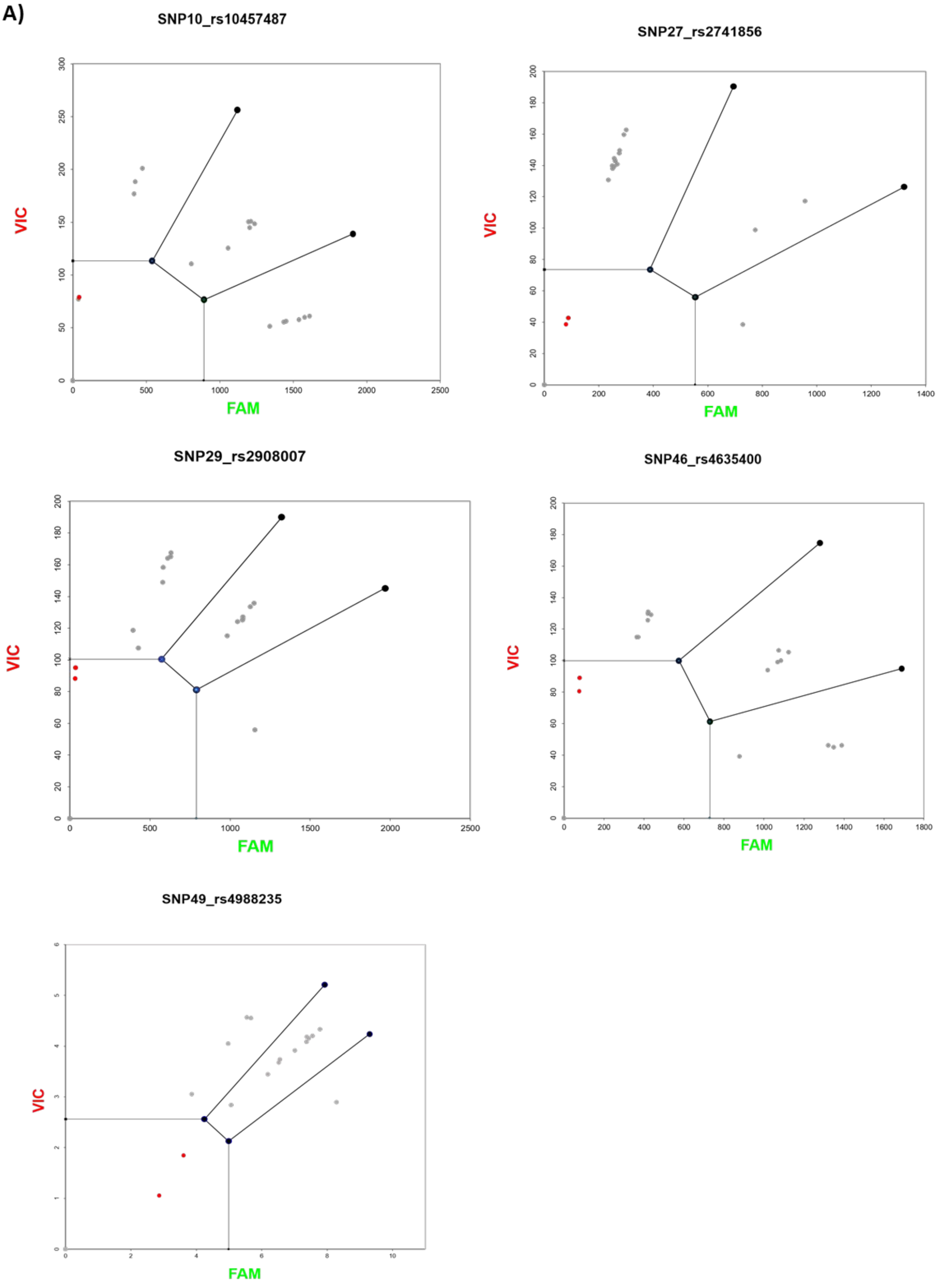
SNP10_rs10457487
VIC
FAM
SNP27_rs2741856
VIC
FAM
SNP29_rs2908007
VIC
FAM
SNP46_rs4635400
VIC
FAM
SNP49_rs4988235
VIC
FAM

B)

Sample	VIC SNP 10	FAM SNP 10	VIC SNP 27	FAM SNP 27	VIC SNP 29	FAM SNP 29	VIC SNP 46	FAM SNP 46	VIC SNP 49	FAM SNP 49
1	56.097	1452.783	142.421	260.612	133.432	1128.858	46.177	1322.522	4.55	5.68
2	150.303	1197.73	98.828	775.023	115.089	982.946	114.903	372.208	3.73	6.56
3	144.691	1205.102	139.171	256.557	107.359	429.098	125.544	419.416	3.44	6.19
4	57.68	1539.021	117.208	957.399	125.86	1081.362	99.836	1085.868	3.05	3.87
5	51.192	1340.462	159.483	292.895	148.832	581.029	98.866	1068.906	3.67	6.53
6	200.901	473.013	162.538	301.801	124.904	1077.696	46.142	1389.652	2.84	5.07
7	60.877	1612.897	147.629	276.278	127.007	1080.43	44.941	1349.024	4.56	5.55
8	55.496	1436.484	139.696	250.041	158.29	583.803	114.79	365.379	3.91	7.02
9	188.13	426.237	143.214	260.368	55.694	1157.51	130.895	420.927	4.05	4.98
10	125.445	1057.277	149.491	277.003	124.037	1049.063	105.281	1124.109	4.18	7.39
11	59.842	1577.814	140.738	268.034	167.377	631.288	128.989	435.94	4.33	7.79
12	150.722	1212.802	144.371	257.172	164.038	611.547	106.421	1073.592	4.2	7.56
13	176.595	417.526	130.701	235.877	135.784	1150.009	129.67	419.479	4.08	7.38
14	148.532	1237.179	137.755	251.382	164.902	630.297	93.898	1018.72	2.89	8.29
15	110.281	807.671	38.428	729.216	118.477	393.99	39.159	877.887	4.15	7.44
NTC	76.947	38.549	38.549	80.305	87.939	35.221	80.305	76.947	1.84	3.62
NTC	78.652	42.555	42.555	88.866	94.94	36.566	88.866	78.652	1.05	2.87

C)

	SNP 10	SNP 27	SNP 29	SNP 46	SNP 49
Sample	rs10457487	rs2741856	rs2908007	rs4635400	rs4988235
1	CC	GG	AG	GG	AA
2	AC	CG	AG	AA	AG
3	AC	GG	AA	AA	AG
4	CC	CG	AG	AG	AA
5	CC	GG	AA	AG	AG
6	AA	GG	AG	GG	AG
7	CC	GG	AG	GG	AA
8	CC	GG	AA	AA	AG
9	AA	GG	GG	AA	AA
10	AC	GG	AG	AG	AG
11	CC	GG	AA	AA	AG
12	AC	GG	AA	AG	AG
13	AA	GG	AG	AA	AG
14	AC	GG	AA	AG	GG
15	AC	CC	AA	GG	AG

Figure S11. TaqMan fluorogenic 5-exonuclease assay for SNP 10, SNP 27, SNP 29, SNP 46 and SNP 49: A) Allelic discrimination plots show three clusters with assigned genotypes as well as a zone for the no template controls (NTCs), shown in red, near the origin. Fluorescent endpoint data points in each cluster are grouped closely together and each cluster is located well away from the other clusters as well as from the NTCs. Data points in the upper left corner show allele 1 (homozygote), labelled with VIC dyes. Data points in the lower right corner display allele 2 (homozygote), labelled with FAM dyes. Data points approximately midway between allele 1 and allele 2 clusters show a mixed (heterozygote) signal for allele 1 and allele 2, double labelled with VIC and FAM dyes. No template controls are located in the lower left corner. B) Raw end-point fluorescent TaqMan PCR data. C) Evaluated genotypes according to allelic discrimination plots.

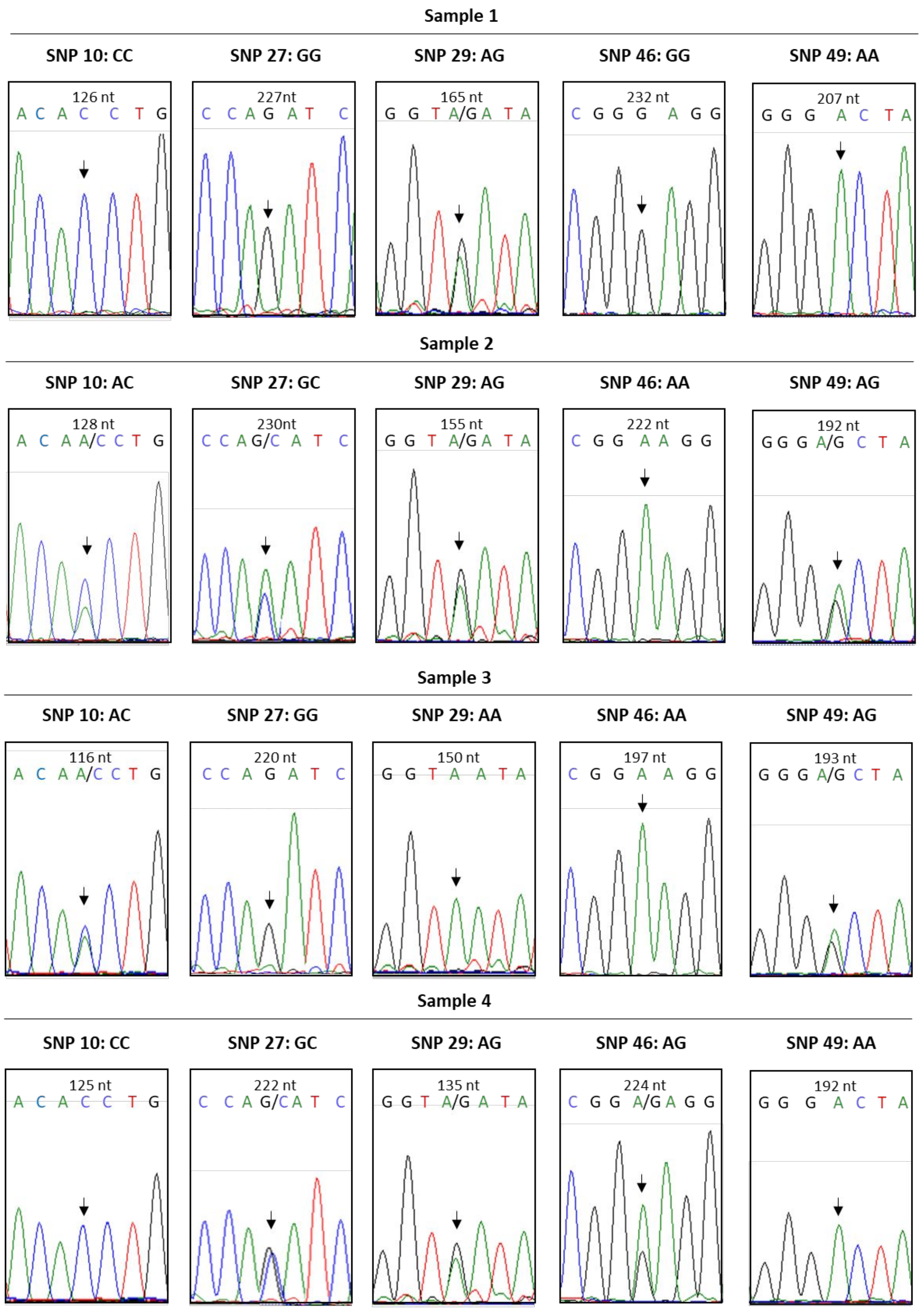
Sample 1
SNP 10: CC
SNP 27: GG
SNP 29: AG
SNP 46: GG
SNP 49: AA
126 nt
A C A C C T G
227nt
C C A G A T C
165 nt
G G T A/G A T A
232 nt
C G G G A G G
207 nt
G G G A C T A
Sample 2
SNP 10: AC
SNP 27: GC
SNP 29: AG
SNP 46: AA
SNP 49: AG
128 nt
A C A A/C C T G
230nt
C C A G/C A T C
155 nt
G G T A/G A T A
222 nt
C G G A A G G
192 nt
G G G A/G C T A
Sample 3
SNP 10: AC
SNP 27: GG
SNP 29: AA
SNP 46: AA
SNP 49: AG
116 nt
A C A A/C C T G
220 nt
C C A G A T C
150 nt
G G T A A T A
197 nt
C G G A A G G
193 nt
G G G A/G C T A
Sample 4
SNP 10: CC
SNP 27: GC
SNP 29: AG
SNP 46: AG
SNP 49: AA
125 nt
A C A C C T G
222 nt
C C A G/C A T C
135 nt
G G T A/G A T A
224 nt
C G G A/G A G G
192 nt
G G G A C T A

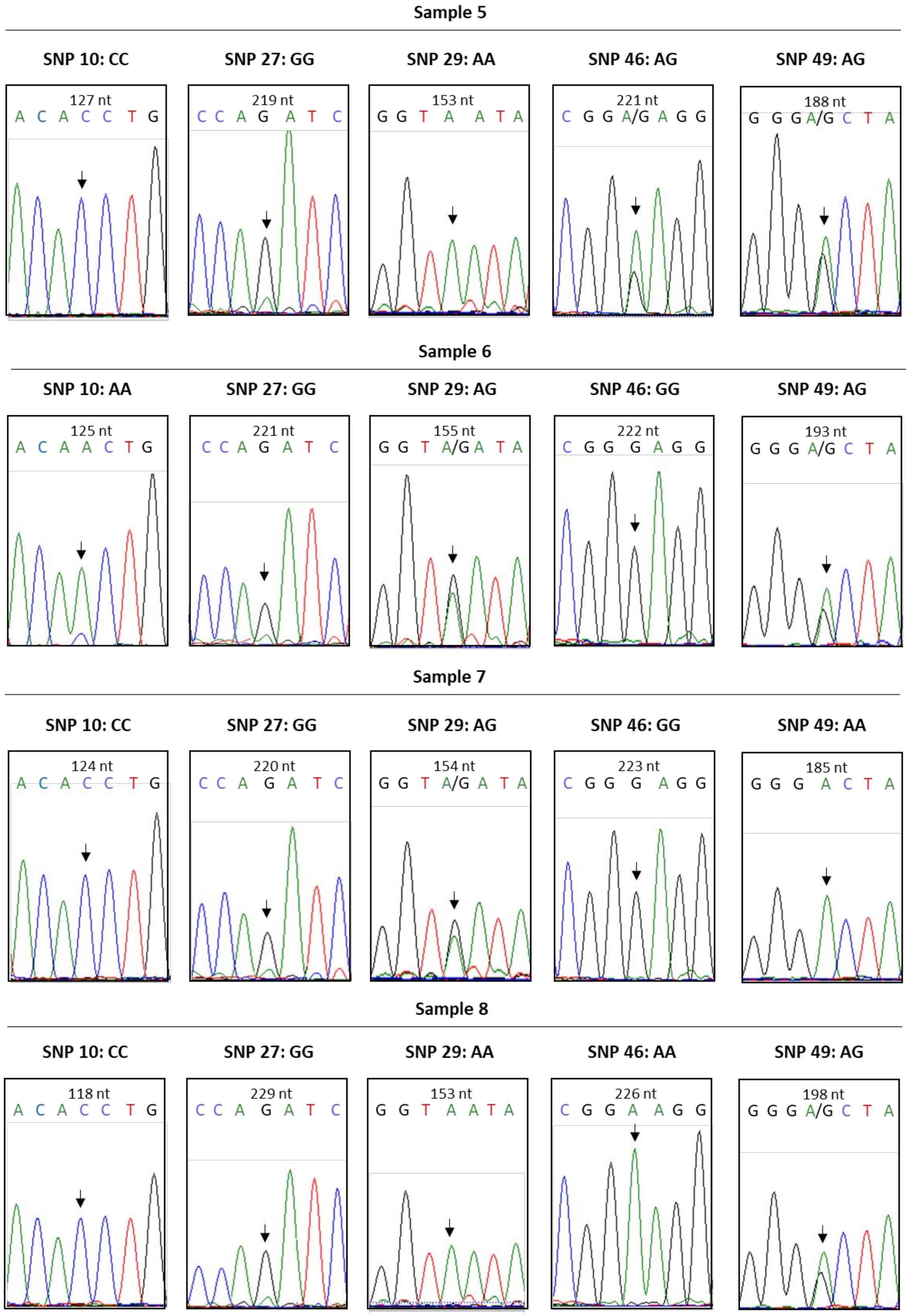
Sample 5
SNP 10: CC
127 nt
A C A C C T G
SNP 27: GG
219 nt
C C A G A T C
SNP 29: AA
153 nt
G G T A A T A
SNP 46: AG
221 nt
C G G A/G A G G
SNP 49: AG
188 nt
G G G A/G C T A
Sample 6
SNP 10: AA
125 nt
A C A A C T G
SNP 27: GG
221 nt
C C A G A T C
SNP 29: AG
155 nt
G G T A/G A T A
SNP 46: GG
222 nt
C G G G A G G
SNP 49: AG
193 nt
G G G A/G C T A
Sample 7
SNP 10: CC
124 nt
A C A C C T G
SNP 27: GG
220 nt
C C A G A T C
SNP 29: AG
154 nt
G G T A/G A T A
SNP 46: GG
223 nt
C G G G A G G
SNP 49: AA
185 nt
G G G A C T A
Sample 8
SNP 10: CC
118 nt
A C A C C T G
SNP 27: GG
229 nt
C C A G A T C
SNP 29: AA
153 nt
G G T A A T A
SNP 46: AA
226 nt
C G G A A G G
SNP 49: AG
198 nt
G G G A/G C T A

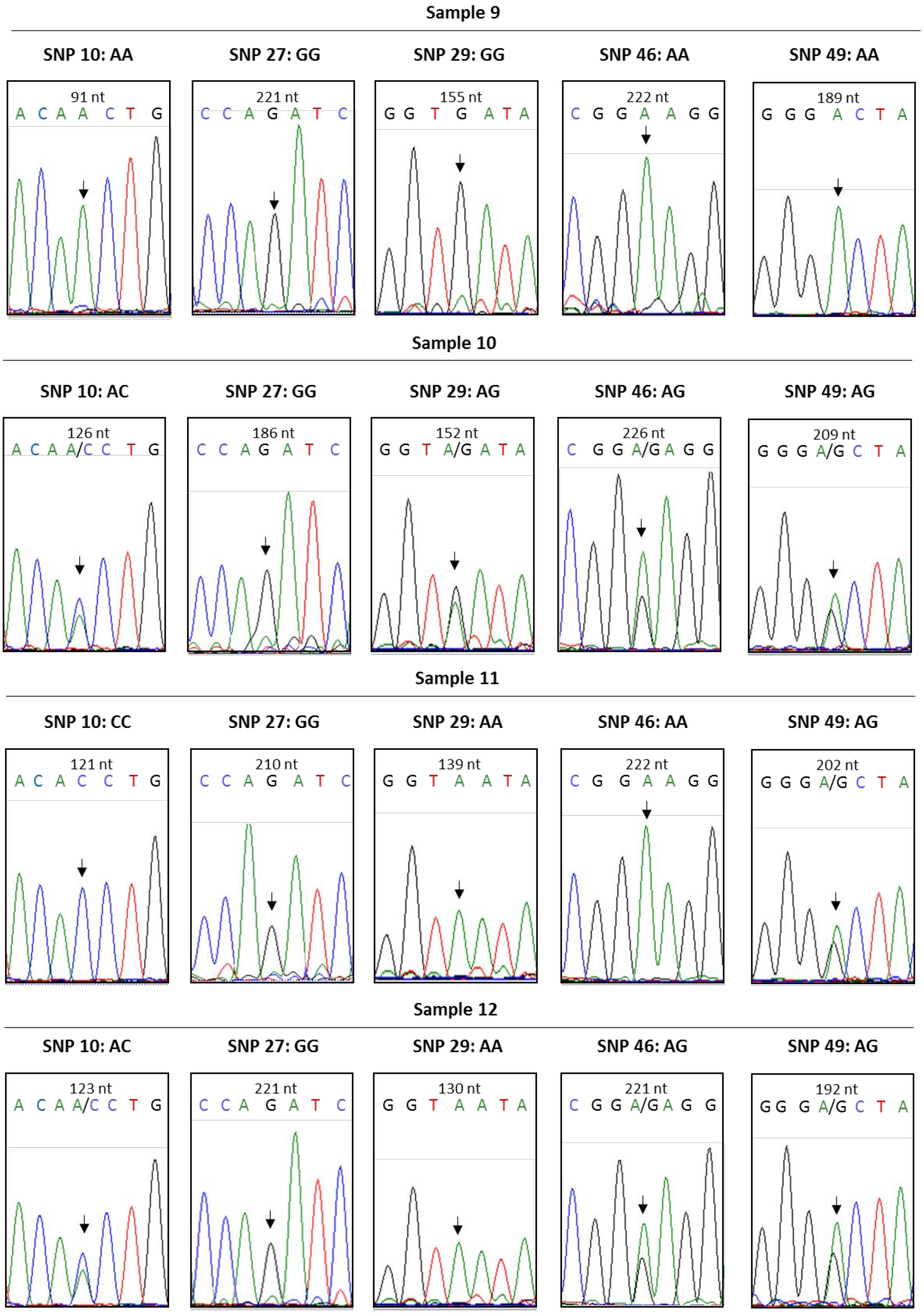

Sample 9
SNP 10: AA
SNP 27: GG
SNP 29: GG
SNP 46: AA
SNP 49: AA
91 nt
A C A A C T G
221 nt
C C A G A T C
155 nt
G G T G A T A
222 nt
C G G A A G G
189 nt
G G G A C T A
Sample 10
SNP 10: AC
SNP 27: GG
SNP 29: AG
SNP 46: AG
SNP 49: AG
126 nt
A C A A/C C T G
186 nt
C C A G A T C
152 nt
G G T A/G A T A
226 nt
C G G A/G A G G
209 nt
G G G A/G C T A
Sample 11
SNP 10: CC
SNP 27: GG
SNP 29: AA
SNP 46: AA
SNP 49: AG
121 nt
A C A C C T G
210 nt
C C A G A T C
139 nt
G G T A A T A
222 nt
C G G A A G G
202 nt
G G G A/G C T A
Sample 12
SNP 10: AC
SNP 27: GG
SNP 29: AA
SNP 46: AG
SNP 49: AG
123 nt
A C A A/C C T G
221 nt
C C A G A T C
130 nt
G G T A A T A
221 nt
C G G A/G A G G
192 nt
G G G A/G C T A

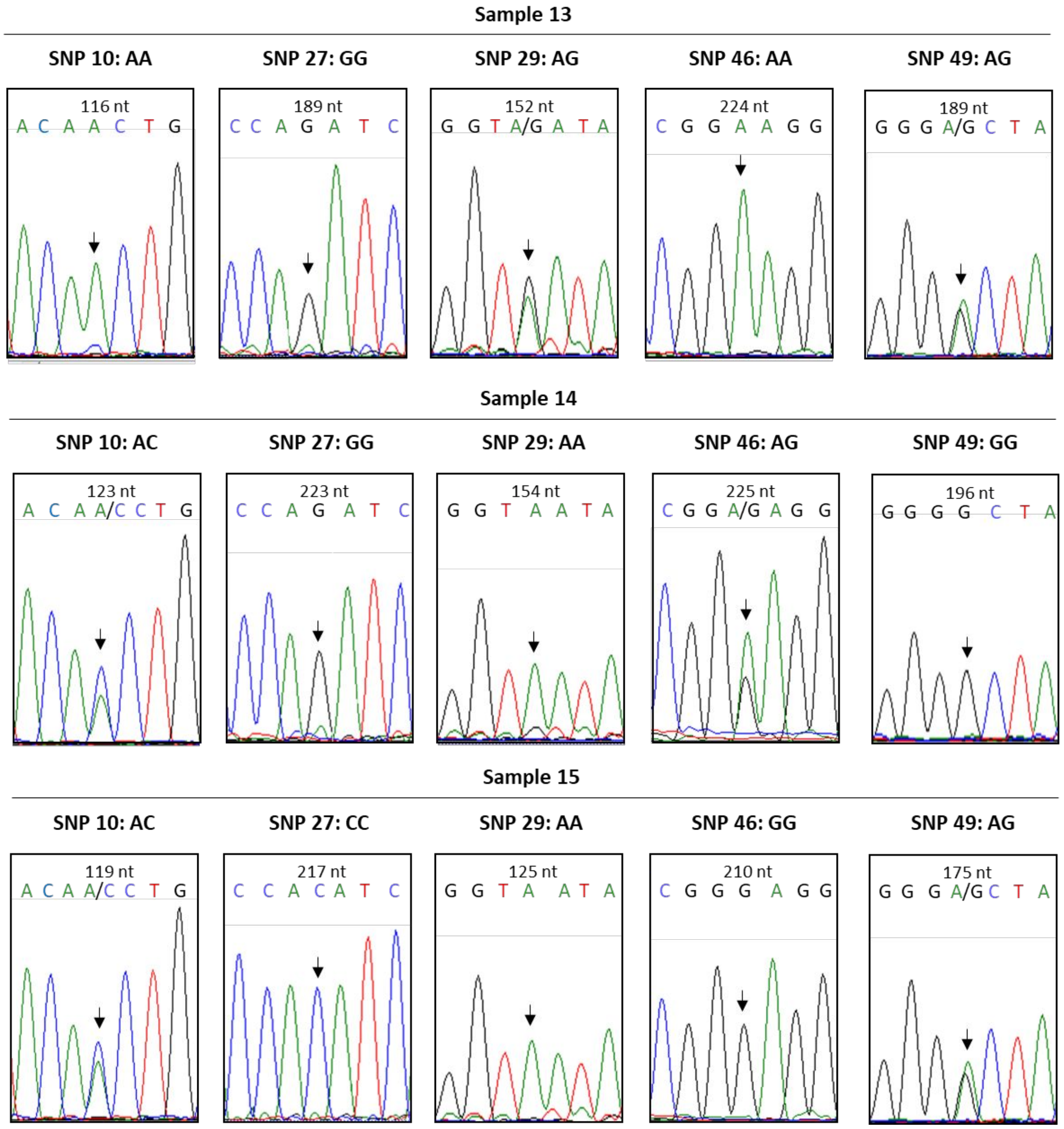

Figure S12. Chromatograms of the SNPs region obtained by Sanger sequencing using the *Bioedit* software.

Sample 1

SNP 10

Sequence ID: **Query_240063** Length: **293** Number of Matches: **1**

Range 1: 37 to 292 Graphics ▼ Next Match ▲ P

Score	Expect	Identities	Gaps	Strand
436 bits(236)	2e-127	253/260(97%)	6/260(2%)	Plus/Plus

```
Query  11   ATA-CATACTTTGCTTCCTGA-TGTTACCACACTCAAGTATAAGTAGCCAAATTATTTTT  68
            ||| ||| |||| ||| |||| ||||||| || |||||||||||||||||||||||||||
Sbjct  37   ATACCAT-CTTT-CTT-CTGAGTGTTACC-CAGTCAAGTATAAGTAGCCAAATTATTTTT  92

Query  69   GCACATCTTTCTGTTTCTCATGTCTTCATTTATTCAACAAGCACTTACTGGGAAGGTCTA  128
            ||||||||||||||||||||||||||||||||||||||||||||||||||||||||||||
Sbjct  93   GCACATCTTTCTGTTTCTCATGTCTTCATTTATTCAACAAGCACTTACTGGGAAGGTCTA  152

Query  129  CACCTGCATAGGCAATGCTGGAAAAAGGGTTAAGTAAACCAGGACATGACAATGGTGGCA  188
            ||||||||||||||||||||||||||||||||||||||||||||||||||||||||||||
Sbjct  153  CACCTGCATAGGCAATGCTGGAAAAAGGGTTAAGTAAACCAGGACATGACAATGGTGGCA  212

Query  189  AATGACTATCAGGTCTTCCCATGTGTTTGACTCAAACTTATTACCCTATGGTCCTTCTGA  248
            ||||||||||||||||||||||||||||||||||||||||||||||||||||||||||||
Sbjct  213  AATGACTATCAGGTCTTCCCATGTGTTTGACTCAAACTTATTACCCTATGGTCCTTCTGA  272

Query  249  CAATGGCAGAAGGTCTGAAT  268
            ||||||||||||||||||||
Sbjct  273  CAATGGCAGAAGGTCTGAAT  292
```

SNP 27

Sequence ID: **Query_3223** Length: **505** Number of Matches: **1**

Range 1: 31 to 505 Graphics ▼ Next Match ▲ Pr

Score	Expect	Identities	Gaps	Strand
782 bits(423)	0.0	460/475(97%)	13/475(2%)	Plus/Plus

```
Query  5    GTCT-GGTG--AAAGCAAGA-TTTGTCTC-aaaaaaaCATGTATAGCTACATAATTAATA  59
            |||| ||||  ||||||||| |||||||| ||||||||||||||||||||||||||||||
Sbjct  31   GTCTGGGTGACAAAGCAAGACTTTGTCTCAAAAAAAACATGTATAGCTACATAATTAATA  90

Query  60   ATATGCAAATATTCTTCTTTAGGGTCACTTAGGTTTTCAACATACAGACCCAAGAGAAGG  119
            ||||||||||||||||||||||||||||||||||||||||||||||||||||||||||||
Sbjct  91   ATATGCAAATATTCTTCTTTAGGGTCACTTAGGTTTTCAACATACAGACCCAAGAGAAGG  150

Query  120  TACAGAGACTCATTCTCTCTCTTTCAAT--------cacacacacacacacacacacaca  171
            ||||||||||||||||||||||||||||        ||||||||||||||||||||||||
Sbjct  151  TACAGAGACTCATTCTCTCTCTTTCAATCACACACACACACACACACACACACACACACA  210

Query  172  cacacacacacacCCCTCTTCAATATATTTATAATTACTATGTTGGCTTCCAGATCAGGG  231
            |||||||||||||||||||||| |||| ||||||||||||||||||||||||||||||||
Sbjct  211  CACACACACACACCCCTCTTCACTATAATTATAATTACTATGTTGGCTTCCAGATCAGGG  270

Query  232  GTTAGAGCCTTGGCATGGAGACGCCTGAAAGGCACCCAAGGCAATTAGTGGTGTCCCTTC  291
            ||||||||||||||||||||||||||||||||||||||||||||||||||||||||||||
Sbjct  271  GTTAGAGCCTTGGCATGGAGACGCCTGAAAGGCACCCAAGGCAATTAGTGGTGTCCCTTC  330

Query  292  TCCACCCCCTACATACCTTCAGGCCCTTTGCACTTGCTGCTCCCCCTCCCAGTGCACACA  351
            ||||||||||||||||||||||||||||||||||||||||||||||||||||||||||||
Sbjct  331  TCCACCCCCTACATACCTTCAGGCCCTTTGCACTTGCTGCTCCCCCTCCCAGTGCACACA  390

Query  352  CCCCCAGATCCTTGTGTGGCTCCCTCTCCCACCACATTTTGGTCTTAGCTCAAATGCCAC  411
            ||||||||||||||||||||||||||||||||||||||||||||||||||||||||||||
Sbjct  391  CCCCCAGATCCTTGTGTGGCTCCCTCTCCCACCACATTTTGGTCTTAGCTCAAATGCCAC  450

Query  412  CTCCTCAGAAATGCCTGGCCTGGCCGCCTTCAGGGCTGTCTATATGCTCACCAGC  466
            |||||||||||||||||||||||||||||||||||||||||||||||||||||||
Sbjct  451  CTCCTCAGAAATGCCTGGCCTGGCCGCCTTCAGGGCTGTCTATATGCTCACCAGC  505
```

SNP 29

Sequence ID: **Query_24961** Length: **384** Number of Matches: **1**

Range 1: 27 to 384 Graphics ▼ Next Match ▲ Pr

Score	Expect	Identities	Gaps	Strand
632 bits(342)	0.0	354/359(99%)	4/359(1%)	Plus/Plus

```
Query  1    CTATGT-ACATACCTGCCC-TTCTGCACACGTATCCCAG-ACTTAAAGTATAATTTTAAA  57
            |||||| ||| |||||||| ||||||||||||||||||| ||||||||||||||||||||
Sbjct  27   CTATGTAACAAACCTGCCCATTCTGCACACGTATCCCAGAACTTAAAGTATAATTTTAAA  86

Query  58   AAAGATGTTTTATAATCTAATTGGTGACTGACAACTAAGGTACGTTAGACCATTAGCAAA  117
            ||||||||||||||||||||||||||||||||||||||||||||||||||||||||||||
Sbjct  87   AAAGATGTTTTATAATCTAATTGGTGACTGACAACTAAGGTACGTTAGACCATTAGCAAA  146

Query  118  AGTAAATTAAGTGATAAGTATTATGGTAGGGACCTCAGGTGAATATAGGAATTGGGCAGA  177
            |||||||||||||||||||||||||||||||||||||||| |||||||||||||||||||
Sbjct  147  AGTAAATTAAGTGATAAGTATTATGGTAGGGACCTCAGGT-AATATAGGAATTGGGCAGA  205

Query  178  GAGAGACGTTAACATGGTTAACTCCAAAAGTGGTATGACCTAGAAAATGGGTAGATAGGT  237
            ||||||||||||||||||||||||||||||||||||||||||||||||||||||||||||
Sbjct  206  GAGAGACGTTAACATGGTTAACTCCAAAAGTGGTATGACCTAGAAAATGGGTAGATAGGT  265

Query  238  TTGAGTAAGTGGAAGGAGAGAAAAGAAATTTCTAGGCATAGACGCAGTGTTTCTCAAGAA  297
            ||||||||||||||||||||||||||||||||||||||||||||||||||||||||||||
Sbjct  266  TTGAGTAAGTGGAAGGAGAGAAAAGAAATTTCTAGGCATAGACGCAGTGTTTCTCAAGAA  325

Query  298  ACTGAAAGACAAATATGCAGGTCCTGCTTATGGAAAAGGGAAGACAGAAGGTGTCTTGC  356
            |||||||||||||||||||||||||||||||||||||||||||||||||||||||||||
Sbjct  326  ACTGAAAGACAAATATGCAGGTCCTGCTTATGGAAAAGGGAAGACAGAAGGTGTCTTGC  384
```

SNP 46

Sequence ID: **Query_9775** Length: **551** Number of Matches: **1**

Range 1: 32 to 548 Graphics ▼ Next Match ▲ P

Score	Expect	Identities	Gaps	Strand
883 bits(478)	0.0	505/517(98%)	5/517(0%)	Plus/Plus

```
Query  7    GCTTACGTT-AAAATC-AG-AATCCAAATAAAATACCACGTTTAATTCAATTTATCACGT  63
            |||||| || |||||| || ||||||||||||||||||||||||||||||||||||||||
Sbjct  32   GCTTACATTAAAAATCAAGAAATCCAAATAAAATACCACGTTTAATTCAATTTATCACGT  91

Query  64   CCAAGAAGTTACAAAGGCAATGTAGAAGAAGTAAGTAAAAATGGGGAAGCCCTAATTTTT  123
            ||||||||||||||||||||||||||||||||||||||||||||||||||||||||||||
Sbjct  92   CCAAGAAGTTACAAAGGCAATGTAGAAGAAGTAAGTAAAAATGGGGAAGCCCTAATTTTT  151

Query  124  AAGTCTGCCTTGAAAACAATTTTTTCTTATCAATGAGACTTTCTAATAGGACTCATCTTG  183
            ||||||||||||||||||||||||||||||||||||||||||||||||||||||||||||
Sbjct  152  AAGTCTGCCTTGAAAACAATTTTTTCTTATCAATGAGACTTTCTAATAGGACTCATCTTG  211

Query  184  CCATTGGCACCCACAAAATCTAACCACCCACCTCCACTTCTCCGGGAGGATCCCTTCCTA  243
            ||||||||||||||||||||||||||||||||||||||||||||||||||||||||||||
Sbjct  212  CCATTGGCACCCACAAAATCTAACCACCCACCTCCACTTCTCCGGGAGGATCCCTTCCTA  271

Query  244  ATGTCTGATGCCTCTTCCCTCACCTCCATTCTTTGCCCACTGCTGCACATGCTTAAAGCC  303
            ||||||||||||||||||||||||||||||||||||||||||||||||||||||||||||
Sbjct  272  ATGTCTGATGCCTCTTCCCTCACCTCCATTCTTTGCCCACTGCTGCACATGCTTAAAGCC  331

Query  304  CAGCCACCCTTTCACCCACAGAGGTCATAGACCACTGCTACTATGGTTAATTCTCAGAAG  363
            ||||||||||||||||||||||||||||||||||||||||||||||||||||||||||||
Sbjct  332  CAGCCACCCTTTCACCCACAGAGGTCATAGACCACTGCTACTATGGTTAATTCTCAGAAG  391

Query  364  TAATAAACTCAGAACCTGAAAGCACATAATGCCTACAAGACTGTTCACTATTTCAAACTT  423
            ||||||||||||||||||||||||||||||||||||||||||||||||||||||||||||
Sbjct  392  TAATAAACTCAGAACCTGAAAGCACATAATGCCTACAAGACTGTTCACTATTTCAAACTT  451

Query  424  CTGCTCTGGTTTACATGAATT-aaaaaaaaaaaaaTCACTCttttttttCTGCTGTCCCA  482
            ||||||||||||||||||||| |||||||||||||||||||| ||||||||||||| |||
Sbjct  452  CTGCTCTGGTTTACATGAATTAAAAAAAAAAAAAATCACTCTATTTTTTCTGCTGTACCA  511

Query  483  GGGCaaaaaaaTTCTACAATCCCTTTTAAGGGC-TGG  518
             |||||||||||||||||||| | ||||| ||| |||
Sbjct  512  AGGCAAAAAAATTCTACAATCACATTTAAAGGCATGG  548
```

SNP 49

Sequence ID: **Query_19171** Length: **439** Number of Matches: **1**

Range 1: 30 to 439 Graphics ▼ Next Match ▲ Prev

Score	Expect	Identities	Gaps	Strand
621 bits(336)	0.0	388/413(94%)	3/413(0%)	Plus/Plus

```
Query  1    TATAACCAGGTAAAAGCAGGGCTTGCTTTTGGTCTGAAGCGAAGATGGGACGCTTGAATG  60
            || ||| ||| ||| ||||||| ||| |||||| ||||||||||||||||||||||||||
Sbjct  30   TAAAACTAGGAAAACGCAGGGC-TGC-TTTGGT-TGAAGCGAAGATGGGACGCTTGAATG  86

Query  61   CCCTTTCGTACTACTCCCCTCCTACCTCGTTAATACCCACTGACCTATCCTCGTGGAATG  120
            ||||||||||||||||||||  ||||||||||||||||||||||||||||||||||||||
Sbjct  87   CCCTTTCGTACTACTCCCCTTTTACCTCGTTAATACCCACTGACCTATCCTCGTGGAATG  146

Query  121  CAGGGCTCAAAGAACAATCTAAAAATCAAACATCATACAAATGCAACCTAAGGAGGAGAG  180
            ||||||||||||||||||||||||||||||||| ||||||||||||||||||||||||||
Sbjct  147  CAGGGCTCAAAGAACAATCTAAAAATCAAACATTATACAAATGCAACCTAAGGAGGAGAG  206

Query  181  CCCCTCCGAGGCCAGGGACTACATCATCTTATCTGTATCGCCAGCGCAGAGGCCTACTAG  240
              |||  |||||||||| |||||| ||||||||||||| |||||||||||||||||||||
Sbjct  207  TTCCTTTGAGGCCAGGGGCTACATTATCTTATCTGTATTGCCAGCGCAGAGGCCTACTAG  266

Query  241  TACATCGTAGGGTCTAAGCACATCCCTCCTGAATGAAAGGTATTAAATGGTAACTTACGT  300
            ||||| |||||||||||| ||||   ||||||||||||||||||||||||||||||||||
Sbjct  267  TACATTGTAGGGTCTAAGTACATTTTTCCTGAATGAAAGGTATTAAATGGTAACTTACGT  326

Query  301  CTTTATGCACTCTATAAACTATGACGTGATCGTCTCCGTCTAACAACTACACTCAAATGC  360
            ||||||||||||||||||||||||||||||||||||||||||||||||||||||||||||
Sbjct  327  CTTTATGCACTCTATAAACTATGACGTGATCGTCTCCGTCTAACAACTACACTCAAATGC  386

Query  361  TTACCAAGCTCTTTAAAGGGAAGAATCCCATGGTCGTATGAGCATAAAACAGT  413
            |||||||||||||||||||||||||| ||||||||||||||||||  ||||||
Sbjct  387  TTACCAAGCTCTTTAAAGGGAAGAATTCCATGGTCGTATGAGCATTCAACAGT  439
```

Sample 2

SNP 10

Sequence ID: **Query_55787** Length: **293** Number of Matches: **1**

Range 1: 47 to 293 Graphics ▼ Next Match ▲ Prev

Score	Expect	Identities	Gaps	Strand
448 bits(242)	1e-130	247/249(99%)	2/249(0%)	Plus/Plus

```
Query  19   TCTTCTGAGTGTTACCCAGATCAAGTATAAGTAGCCAAATTATTTTTGCACATCTTTCTG  78
            ||||||||||||||||||| ||||||||||||||||||||||||||||||||||||||||
Sbjct  47   TCTTCTGAGTGTTACCCAG-TCAAGTATAAGTAGCCAAATTATTTTTGCACATCTTTCTG  105

Query  79   TTTCTCATGTCTTCATTTATTCAACAAGCACTTACTGGGAAGGTCTACAACCTGCATAGG  138
            |||||||||||||||||||||||||||||||||||||||||||||||| |||||||||||
Sbjct  106  TTTCTCATGTCTTCATTTATTCAACAAGCACTTACTGGGAAGGTCTAC-ACCTGCATAGG  164

Query  139  CAATGCTGGAAAAAGGGTTAAGTAAACCAGGACATGACAATGGTGGCAAATGACTATCAG  198
            ||||||||||||||||||||||||||||||||||||||||||||||||||||||||||||
Sbjct  165  CAATGCTGGAAAAAGGGTTAAGTAAACCAGGACATGACAATGGTGGCAAATGACTATCAG  224

Query  199  GTCTTCCCATGTGTTTGACTCAAACTTATTACCCTATGGTCCTTCTGACAATGGCAGAAG  258
            ||||||||||||||||||||||||||||||||||||||||||||||||||||||||||||
Sbjct  225  GTCTTCCCATGTGTTTGACTCAAACTTATTACCCTATGGTCCTTCTGACAATGGCAGAAG  284

Query  259  GTCTGAATC  267
            |||||||||
Sbjct  285  GTCTGAATC  293
```

SNP 27

Sequence ID: **Query_501361** Length: **505** Number of Matches: **1**

Range 1: 52 to 505 Graphics ▼ Next Match ▲

Score	Expect	Identities	Gaps	Strand
776 bits(420)	0.0	445/455(98%)	9/455(1%)	Plus/Plus

```
Query  27   TTTGTCTCaaaaaaaaCATGTATAGCTACATAATTAATAATATGCAAATATTCTTCTTTA  86
            ||||||||||||||||||||||||||||||||||||||||||||||||||||||||||||
Sbjct  52   TTTGTCTCAAAAAAAACATGTATAGCTACATAATTAATAATATGCAAATATTCTTCTTTA  111

Query  87   GGGTCACTTAGGTTTTCAACATACAGACCCAAGAGAAGGTACAGAGACTCATTCTCTCTC  146
            ||||||||||||||||||||||||||||||||||||||||||||||||||||||||||||
Sbjct  112  GGGTCACTTAGGTTTTCAACATACAGACCCAAGAGAAGGTACAGAGACTCATTCTCTCTC  171

Query  147  TCTTTCAAT--------cacacacacacacacacacacacacacacacacacCCCTCTTC  198
            |||||||||        |||||||||||||||||||||||||||||||||||||||||||
Sbjct  172  TCTTTCAATCACACACACACACACACACACACACACACACACACACACACACCCCTCTTC  231

Query  199  ACTATAATTATAATTACTATGTTGGCTTCCAACATCAGGGGTTAGAGCCTTGGCATGGAG  258
            |||||||||||||||||||||||||||||||  |||||||||||||||||||||||||||
Sbjct  232  ACTATAATTATAATTACTATGTTGGCTTCCAG-ATCAGGGGTTAGAGCCTTGGCATGGAG  290

Query  259  ACGCCTGAAAGGCACCCAAGGCAATTAGTGGTGTCCCTTCTCCACCCCCTACATACCTTC  318
            ||||||||||||||||||||||||||||||||||||||||||||||||||||||||||||
Sbjct  291  ACGCCTGAAAGGCACCCAAGGCAATTAGTGGTGTCCCTTCTCCACCCCCTACATACCTTC  350

Query  319  AGGCCCTTTGCACTTGCTGCTCCCCCTCCCAGTGCACACACCCCCAGATCCTTGTGTGGC  378
            ||||||||||||||||||||||||||||||||||||||||||||||||||||||||||||
Sbjct  351  AGGCCCTTTGCACTTGCTGCTCCCCCTCCCAGTGCACACACCCCCAGATCCTTGTGTGGC  410

Query  379  TCCCTCTCCCACCACATTTTGGTCTTAGCTCAAATGCCACCTCCTCAGAAATGCCTGGCC  438
            ||||||||||||||||||||||||||||||||||||||||||||||||||||||||||||
Sbjct  411  TCCCTCTCCCACCACATTTTGGTCTTAGCTCAAATGCCACCTCCTCAGAAATGCCTGGCC  470

Query  439  TGGCCGCCTTCAGGGCTGTCTATATGCTCACCAGC  473
            |||||||||||||||||||||||||||||||||||
Sbjct  471  TGGCCGCCTTCAGGGCTGTCTATATGCTCACCAGC  505
```

SNP 29

Sequence ID: **Query_9257** Length: **384** Number of Matches: **1**

Range 1: 35 to 384 Graphics ▼ Next Match ▲

Score	Expect	Identities	Gaps	Strand
636 bits(344)	0.0	349/351(99%)	2/351(0%)	Plus/Plus

```
Query  4    CAAACCTG-CCATTCTGCACACGTATCCCAGAACTTAAAGTATAATTTTAAAAAAGATGT  62
            |||||||| |||||||||||||||||||||||||||||||||||||||||||||||||||
Sbjct  35   CAAACCTGCCCATTCTGCACACGTATCCCAGAACTTAAAGTATAATTTTAAAAAAGATGT  94

Query  63   TTTATAATCTAATTGGTGACTGACAACTAAGGTACGTTAGACCATTAGCAAAAGTAAATT  122
            ||||||||||||||||||||||||||||||||||||||||||||||||||||||||||||
Sbjct  95   TTTATAATCTAATTGGTGACTGACAACTAAGGTACGTTAGACCATTAGCAAAAGTAAATT  154

Query  123  AAGTGATAAGTATTATGGTAGGACCTCAGGTAGATATAGGAATTGGGCAGAGAGAGACG  182
            |||||||||||||||||||||||||||||||| |||||||||||||||||||||||||||
Sbjct  155  AAGTGATAAGTATTATGGTAGGACCTCAGGTA-ATATAGGAATTGGGCAGAGAGAGACG  213

Query  183  TTAACATGGTTAACTCCAAAAGTGGTATGACCTAGAAAATGGGTAGATAGGTTTGAGTAA  242
            ||||||||||||||||||||||||||||||||||||||||||||||||||||||||||||
Sbjct  214  TTAACATGGTTAACTCCAAAAGTGGTATGACCTAGAAAATGGGTAGATAGGTTTGAGTAA  273

Query  243  GTGGAAGGAGAGAAAAGAAATTTCTAGGCATAGACGCAGTGTTTCTCAAGAAACTGAAAG  302
            ||||||||||||||||||||||||||||||||||||||||||||||||||||||||||||
Sbjct  274  GTGGAAGGAGAGAAAAGAAATTTCTAGGCATAGACGCAGTGTTTCTCAAGAAACTGAAAG  333

Query  303  ACAAATATGCAGGTCCTGCTTATGGAAAAGGGAAGACAGAAGGTGTCTTGC  353
            |||||||||||||||||||||||||||||||||||||||||||||||||||
Sbjct  334  ACAAATATGCAGGTCCTGCTTATGGAAAAGGGAAGACAGAAGGTGTCTTGC  384
```

SNP 46

Sequence ID: **Query_50919** Length: **551** Number of Matches: **1**

Range 1: 43 to 549 Graphics ▼ Next Match ▲

Score	Expect	Identities	Gaps	Strand
900 bits(487)	0.0	501/507(99%)	3/507(0%)	Plus/Plus

```
Query  10   AAATC-AG-AATCCAAATAAAATACCACGTTTAATTCAATTTATCACGTCCAAGAAGTTA  67
            ||||| || ||||||||||||||||||||||||||||||||||||||||||||||||||| 
Sbjct  43   AAATCAAGAAATCCAAATAAAATACCACGTTTAATTCAATTTATCACGTCCAAGAAGTTA  102

Query  68   CAAAGGCAATGTAGAAGAAGTAAGTAAAAATGGGGAAGCCCTAATTTTTAAGTCTGCCTT  127
            ||||||||||||||||||||||||||||||||||||||||||||||||||||||||||||
Sbjct  103  CAAAGGCAATGTAGAAGAAGTAAGTAAAAATGGGGAAGCCCTAATTTTTAAGTCTGCCTT  162

Query  128  GAAAACAATTTTTTCTTATCAATGAGACTTTCTAATAGGACTCATCTTGCCATTGGCACC  187
            ||||||||||||||||||||||||||||||||||||||||||||||||||||||||||||
Sbjct  163  GAAAACAATTTTTTCTTATCAATGAGACTTTCTAATAGGACTCATCTTGCCATTGGCACC  222

Query  188  CACAAAATCTAACCACCCACCTCCACTTCTCCGGAAGGATCCCTTCCTAATGTCTGATGC  247
            |||||||||||||||||||||||||||||||||| |||||||||||||||||||||||||
Sbjct  223  CACAAAATCTAACCACCCACCTCCACTTCTCCGGGAGGATCCCTTCCTAATGTCTGATGC  282

Query  248  CTCTTCCCTCACCTCCATTCTTTGCCCACTGCTGCACATGCTTAAAGCCCAGCCACCCTT  307
            ||||||||||||||||||||||||||||||||||||||||||||||||||||||||||||
Sbjct  283  CTCTTCCCTCACCTCCATTCTTTGCCCACTGCTGCACATGCTTAAAGCCCAGCCACCCTT  342

Query  308  TCACCCACAGAGGTCATAGACCACTGCTACTATGGTTAATTCTCAGAAGTAATAAACTCA  367
            ||||||||||||||||||||||||||||||||||||||||||||||||||||||||||||
Sbjct  343  TCACCCACAGAGGTCATAGACCACTGCTACTATGGTTAATTCTCAGAAGTAATAAACTCA  402

Query  368  GAACCTGAAAGCACATAATGCCTACAAGACTGTTCACTATTTCAAACTTCTGCTCTGGTT  427
            ||||||||||||||||||||||||||||||||||||||||||||||||||||||||||||
Sbjct  403  GAACCTGAAAGCACATAATGCCTACAAGACTGTTCACTATTTCAAACTTCTGCTCTGGTT  462

Query  428  TACATGAATT-aaaaaaaaaaaaaTCACTCTATTTTTTCTGCTGTACCAAGGCaaaaaaa  486
            |||||||||| |||||||||||||||||||||||||||||||||||||||||||||||||
Sbjct  463  TACATGAATTAAAAAAAAAAAAAATCACTCTATTTTTTCTGCTGTACCAAGGCAAAAAAA  522

Query  487  TTCTACAATCCCTTTTAAAGGCATGGA  513
            |||||||||| | ||||||||||||||
Sbjct  523  TTCTACAATCACATTTAAAGGCATGGA  549
```

SNP 49

Sequence ID: **Query_16069** Length: **439** Number of Matches: **1**

Range 1: 40 to 439 Graphics ▼ Next Match

Score	Expect	Identities	Gaps	Strand
712 bits(385)	0.0	397/402(99%)	3/402(0%)	Plus/Plus

```
Query  8    AAAAAGCATAGGCTGCTTTGGTTG-AGCGAAGATGGGACGCTTGAATGCCCTTTCGTACT  66
            |||| |||  |||||||||||||| |||||||||||||||||||||||||||||||||||
Sbjct  40   AAAACGCA-GGGCTGCTTTGGTTGAAGCGAAGATGGGACGCTTGAATGCCCTTTCGTACT  98

Query  67   ACTCCCCTTTTACCTCGTTAATACCCACTGACCTATCCTCGTGGAATGCAGGGCTCAAAG  126
            ||||||||||||||||||||||||||||||||||||||||||||||||||||||||||||
Sbjct  99   ACTCCCCTTTTACCTCGTTAATACCCACTGACCTATCCTCGTGGAATGCAGGGCTCAAAG  158

Query  127  AACAATCTAAAAATCAAACATTATACAAATGCAACCTAAGGAGGAGAGTTCCTTTGAGGC  186
            ||||||||||||||||||||||||||||||||||||||||||||||||||||||||||||
Sbjct  159  AACAATCTAAAAATCAAACATTATACAAATGCAACCTAAGGAGGAGAGTTCCTTTGAGGC  218

Query  187  CAGGGAGCTACATTATCTTATCTGTATTGCCAGCGCAGAGGCCTACTAGTACATTGTAGG  246
            ||||| ||||||||||||||||||||||||||||||||||||||||||||||||||||||
Sbjct  219  CAGGG-GCTACATTATCTTATCTGTATTGCCAGCGCAGAGGCCTACTAGTACATTGTAGG  277

Query  247  GTCTAAGTACATTTTTCCTGAATGAAAGGTATTAAATGGTAACTTACGTCTTTATGCACT  306
            ||||||||||||||||||||||||||||||||||||||||||||||||||||||||||||
Sbjct  278  GTCTAAGTACATTTTTCCTGAATGAAAGGTATTAAATGGTAACTTACGTCTTTATGCACT  337

Query  307  CTATAAACTATGACGTGATCGTCTCCGTCTAACAACTACACTCAAATGCTTACCAAGCTC  366
            ||||||||||||||||||||||||||||||||||||||||||||||||||||||||||||
Sbjct  338  CTATAAACTATGACGTGATCGTCTCCGTCTAACAACTACACTCAAATGCTTACCAAGCTC  397

Query  367  TTTAAAGGGAAGAATTCCATGGTCGTATGAGCATTCAACAGT  408
            ||||||||||||||||||||||||||||||||||||||||||
Sbjct  398  TTTAAAGGGAAGAATTCCATGGTCGTATGAGCATTCAACAGT  439
```

Sample 3

SNP 10

Sequence ID: **Query_42195** Length: **293** Number of Matches: **1**

Range 1: 41 to 293 Graphics ▼ Next Match ▲ Prev

Score	Expect	Identities	Gaps	Strand
459 bits(248)	4e-134	253/255(99%)	2/255(0%)	Plus/Plus

```
Query  1    CATCATTTCTTCTGAGTGTTACCCAGTCAAGTATAAGTAGCCAAATTATTTTTGCACATC  60
            |||| |||||||||||||||||||||||||||||||||||||||||||||||||||||||
Sbjct  41   CATC-TTTCTTCTGAGTGTTACCCAGTCAAGTATAAGTAGCCAAATTATTTTTGCACATC  99

Query  61   TTTCTGTTTCTCATGTCTTCATTTATTCAACAAGCACTTACTGGGAAGGTCTACAACCTG  120
            |||||||||||||||||||||||||||||||||||||||||||||||||||||| |||||
Sbjct  100  TTTCTGTTTCTCATGTCTTCATTTATTCAACAAGCACTTACTGGGAAGGTCTAC-ACCTG  158

Query  121  CATAGGCAATGCTGGAAAAAGGGTTAAGTAAACCAGGACATGACAATGGTGGCAAATGAC  180
            ||||||||||||||||||||||||||||||||||||||||||||||||||||||||||||
Sbjct  159  CATAGGCAATGCTGGAAAAAGGGTTAAGTAAACCAGGACATGACAATGGTGGCAAATGAC  218

Query  181  TATCAGGTCTTCCCATGTGTTTGACTCAAACTTATTACCCTATGGTCCTTCTGACAATGG  240
            ||||||||||||||||||||||||||||||||||||||||||||||||||||||||||||
Sbjct  219  TATCAGGTCTTCCCATGTGTTTGACTCAAACTTATTACCCTATGGTCCTTCTGACAATGG  278

Query  241  CAGAAGGTCTGAATC  255
            |||||||||||||||
Sbjct  279  CAGAAGGTCTGAATC  293
```

SNP 27

Sequence ID: **Query_62885** Length: **505** Number of Matches: **1**

Range 1: 44 to 505 Graphics ▼ Next Match ▲

Score	Expect	Identities	Gaps	Strand
782 bits(423)	0.0	451/462(98%)	11/462(2%)	Plus/Plus

```
Query  12   AGC-AGACTTTGTCTC--AAAAAACATGTATAGCTACATAATTAATAATATGCAAATATT  68
            ||| ||||||||||||  ||||||||||||||||||||||||||||||||||||||||||
Sbjct  44   AGCAAGACTTTGTCTCAAAAAAAACATGTATAGCTACATAATTAATAATATGCAAATATT  103

Query  69   CTTCTTTAGGGTCACTTAGGTTTTCAACATACAGACCCAAGAGAAGGTACAGAGACTCAT  128
            ||||||||||||||||||||||||||||||||||||||||||||||||||||||||||||
Sbjct  104  CTTCTTTAGGGTCACTTAGGTTTTCAACATACAGACCCAAGAGAAGGTACAGAGACTCAT  163

Query  129  TCTCTCTCTCTTTCAAT--------cacacacacacacacacacacacacacacacacac  180
            |||||||||||||||||        |||||||||||||||||||||||||||||||||||
Sbjct  164  TCTCTCTCTCTTTCAATCACACACACACACACACACACACACACACACACACACACACAC  223

Query  181  CCCTCTTCACTATAATTATAATTACTATGTTGGCTTCCAGATCAGGGGTTAGAGCCTTGG  240
            ||||||||||||||||||||||||||||||||||||||||||||||||||||||||||||
Sbjct  224  CCCTCTTCACTATAATTATAATTACTATGTTGGCTTCCAGATCAGGGGTTAGAGCCTTGG  283

Query  241  CATGGAGACGCCTGAAAGGCACCCAAGGCAATTAGTGGTGTCCCTTCTCCACCCCCTACA  300
            ||||||||||||||||||||||||||||||||||||||||||||||||||||||||||||
Sbjct  284  CATGGAGACGCCTGAAAGGCACCCAAGGCAATTAGTGGTGTCCCTTCTCCACCCCCTACA  343

Query  301  TACCTTCAGGCCCTTTGCACTTGCTGCTCCCCCTCCCAGTGCACACACCCCCAGATCCTT  360
            ||||||||||||||||||||||||||||||||||||||||||||||||||||||||||||
Sbjct  344  TACCTTCAGGCCCTTTGCACTTGCTGCTCCCCCTCCCAGTGCACACACCCCCAGATCCTT  403

Query  361  GTGTGGCTCCCTCTCCCACCACATTTTGGTCTTAGCTCAAATGCCACCTCCTCAGAAATG  420
            ||||||||||||||||||||||||||||||||||||||||||||||||||||||||||||
Sbjct  404  GTGTGGCTCCCTCTCCCACCACATTTTGGTCTTAGCTCAAATGCCACCTCCTCAGAAATG  463

Query  421  CCTGGCCTGGCCGCCTTCAGGGCTGTCTATATGCTCACCAGC  462
            ||||||||||||||||||||||||||||||||||||||||||
Sbjct  464  CCTGGCCTGGCCGCCTTCAGGGCTGTCTATATGCTCACCAGC  505
```

SNP 29

Sequence ID: **Query_49195** Length: **384** Number of Matches: **1**

Range 1: 42 to 384 Graphics ▼ Next Match ▲

Score	Expect	Identities	Gaps	Strand
628 bits(340)	0.0	343/344(99%)	1/344(0%)	Plus/Plus

```
Query  5    GCCCATTCTGCACACGTATCCCAGAACTTAAAGTATAATTTTAAAAAAGATGTTTTATAA  64
            ||||||||||||||||||||||||||||||||||||||||||||||||||||||||||||
Sbjct  42   GCCCATTCTGCACACGTATCCCAGAACTTAAAGTATAATTTTAAAAAAGATGTTTTATAA  101

Query  65   TCTAATTGGTGACTGACAACTAAGGTACGTTAGACCATTAGCAAAAGTAAATTAAGTGAT  124
            ||||||||||||||||||||||||||||||||||||||||||||||||||||||||||||
Sbjct  102  TCTAATTGGTGACTGACAACTAAGGTACGTTAGACCATTAGCAAAAGTAAATTAAGTGAT  161

Query  125  AAGTATTATGGTAGGGACCTCAGGTAATATAGGAATTGGGCAGAGAGAGACGTTAACATG  184
            ||||||||||||||||||||||||||||||||||||||||||||||||||||||||||||
Sbjct  162  AAGTATTATGGTAGGGACCTCAGGTAATATAGGAATTGGGCAGAGAGAGACGTTAACATG  221

Query  185  GTTAACTCCAAAAGTGGTATGACCTAGAAAATGGGTAGATAGGTTTGAGTAAGTGGAAGG  244
            ||||||||||||||||||||||||||||||||||||||||||||||||||||||||||||
Sbjct  222  GTTAACTCCAAAAGTGGTATGACCTAGAAAATGGGTAGATAGGTTTGAGTAAGTGGAAGG  281

Query  245  AGAGAAAAGAAATTTCTAGGCATAGACGCAGTGTTTCTCAAGAAACTGAAAGACAAATAT  304
            ||||||||||||||||||||||||||||||||||||||||||||||||||||||||||||
Sbjct  282  AGAGAAAAGAAATTTCTAGGCATAGACGCAGTGTTTCTCAAGAAACTGAAAGACAAATAT  341

Query  305  GCAGGTCCTGCTTATGGAAAAGGGAAGACAGAAGGGTGTCTTGC  348
            ||||||||||||||||||||||||||||||||||| ||||||||
Sbjct  342  GCAGGTCCTGCTTATGGAAAAGGGAAGACAGAAGG-TGTCTTGC  384
```

SNP 46

```
Sequence ID: Query_62729  Length: 551  Number of Matches: 1

Range 1: 63 to 539  Graphics                                          ▼ Next Match ▲

Score           Expect   Identities        Gaps          Strand
859 bits(465)   0.0      475/479(99%)      3/479(0%)     Plus/Plus

Query  1    AATAACCACGGTTTAATTCAATTTATCACGTCCAAGAAGTTACAAAGGCAATGTAGAAGA  60
            ||| ||||| ||||||||||||||||||||||||||||||||||||||||||||||||||
Sbjct  63   AAT-ACCAC-GTTTAATTCAATTTATCACGTCCAAGAAGTTACAAAGGCAATGTAGAAGA  120

Query  61   AGTAAGTAAAAATGGGGAAGCCCTAATTTTTAAGTCTGCCTTGAAAACAATTTTTTCTTA  120
            ||||||||||||||||||||||||||||||||||||||||||||||||||||||||||||
Sbjct  121  AGTAAGTAAAAATGGGGAAGCCCTAATTTTTAAGTCTGCCTTGAAAACAATTTTTTCTTA  180

Query  121  TCAATGAGACTTTCTAATAGGACTCATCTTGCCATTGGCACCCACAAAATCTAACCACCC  180
            ||||||||||||||||||||||||||||||||||||||||||||||||||||||||||||
Sbjct  181  TCAATGAGACTTTCTAATAGGACTCATCTTGCCATTGGCACCCACAAAATCTAACCACCC  240

Query  181  ACCTCCACTTCTCCGGAAGGATCCCTTCCTAATGTCTGATGCCTCTTCCCTCACCTCCAT  240
            |||||||||||||||| |||||||||||||||||||||||||||||||||||||||||||
Sbjct  241  ACCTCCACTTCTCCGGGAGGATCCCTTCCTAATGTCTGATGCCTCTTCCCTCACCTCCAT  300

Query  241  TCTTTGCCCACTGCTGCACATGCTTAAAGCCCAGCCACCCTTTCACCCACAGAGGTCATA  300
            ||||||||||||||||||||||||||||||||||||||||||||||||||||||||||||
Sbjct  301  TCTTTGCCCACTGCTGCACATGCTTAAAGCCCAGCCACCCTTTCACCCACAGAGGTCATA  360

Query  301  GACCACTGCTACTATGGTTAATTCTCAGAAGTAATAAACTCAGAACCTGAAAGCACATAA  360
            ||||||||||||||||||||||||||||||||||||||||||||||||||||||||||||
Sbjct  361  GACCACTGCTACTATGGTTAATTCTCAGAAGTAATAAACTCAGAACCTGAAAGCACATAA  420

Query  361  TGCCTACAAGACTGTTCACTATTTCAAACTTCTGCTCTGGTTTACATGAATT-aaaaaaa  419
            |||||||||||||||||||||||||||||||||||||||||||||||||||| |||||||
Sbjct  421  TGCCTACAAGACTGTTCACTATTTCAAACTTCTGCTCTGGTTTACATGAATTAAAAAAAA  480

Query  420  aaaaaaTCACTCTATTTTTTCTGCTGTACCAAGGCaaaaaaaTTCTACAATCACATTTA  478
            |||||||||||||||||||||||||||||||||||||||||||||||||||||||||||
Sbjct  481  AAAAAATCACTCTATTTTTTCTGCTGTACCAAGGCAAAAAAATTCTACAATCACATTTA  539
```

SNP 49

```
Sequence ID: Query_775  Length: 439  Number of Matches: 1

Range 1: 49 to 439  Graphics                                          ▼ Next Match ▲

Score           Expect   Identities        Gaps          Strand
712 bits(385)   0.0      390/392(99%)      2/392(0%)     Plus/Plus

Query  19   GGCTGCTTTGGTTG-AGCGAAGATGGGACGCTTGAATGCCCTTTCGTACTACTCCCCTTT  77
            |||||||||||||| |||||||||||||||||||||||||||||||||||||||||||||
Sbjct  49   GGCTGCTTTGGTTGAAGCGAAGATGGGACGCTTGAATGCCCTTTCGTACTACTCCCCTTT  108

Query  78   TACCTCGTTAATACCCACTGACCTATCCTCGTGGAATGCAGGGCTCAAAGAACAATCTAA  137
            ||||||||||||||||||||||||||||||||||||||||||||||||||||||||||||
Sbjct  109  TACCTCGTTAATACCCACTGACCTATCCTCGTGGAATGCAGGGCTCAAAGAACAATCTAA  168

Query  138  AAATCAAACATTATACAAATGCAACCTAAGGAGGAGAGTTCCTTTGAGGCCAGGGAGCTA  197
            ||||||||||||||||||||||||||||||||||||||||||||||||||||||| ||||
Sbjct  169  AAATCAAACATTATACAAATGCAACCTAAGGAGGAGAGTTCCTTTGAGGCCAGGG-GCTA  227

Query  198  CATTATCTTATCTGTATTGCCAGCGCAGAGGCCTACTAGTACATTGTAGGGTCTAAGTAC  257
            ||||||||||||||||||||||||||||||||||||||||||||||||||||||||||||
Sbjct  228  CATTATCTTATCTGTATTGCCAGCGCAGAGGCCTACTAGTACATTGTAGGGTCTAAGTAC  287

Query  258  ATTTTTCCTGAATGAAAGGTATTAAATGGTAACTTACGTCTTTATGCACTCTATAAACTA  317
            ||||||||||||||||||||||||||||||||||||||||||||||||||||||||||||
Sbjct  288  ATTTTTCCTGAATGAAAGGTATTAAATGGTAACTTACGTCTTTATGCACTCTATAAACTA  347

Query  318  TGACGTGATCGTCTCCGTCTAACAACTACACTCAAATGCTTACCAAGCTCTTTAAAGGGA  377
            ||||||||||||||||||||||||||||||||||||||||||||||||||||||||||||
Sbjct  348  TGACGTGATCGTCTCCGTCTAACAACTACACTCAAATGCTTACCAAGCTCTTTAAAGGGA  407

Query  378  AGAATTCCATGGTCGTATGAGCATTCAACAGT  409
            ||||||||||||||||||||||||||||||||
Sbjct  408  AGAATTCCATGGTCGTATGAGCATTCAACAGT  439
```

Sample 4

SNP 10

```
Sequence ID: Query_54171  Length: 293  Number of Matches: 1

Range 1: 46 to 293  Graphics                                   ▼ Next Match  ▲

Score           Expect     Identities        Gaps          Strand
459 bits(248)   5e-134     248/248(100%)     0/248(0%)     Plus/Plus

Query  16   TTCTTCTGAGTGTTACCCAGTCAAGTATAAGTAGCCAAATTATTTTTGCACATCTTTCTG  75
            ||||||||||||||||||||||||||||||||||||||||||||||||||||||||||||
Sbjct  46   TTCTTCTGAGTGTTACCCAGTCAAGTATAAGTAGCCAAATTATTTTTGCACATCTTTCTG  105

Query  76   TTTCTCATGTCTTCATTTATTCAACAAGCACTTACTGGGAAGGTCTACACCTGCATAGGC  135
            ||||||||||||||||||||||||||||||||||||||||||||||||||||||||||||
Sbjct  106  TTTCTCATGTCTTCATTTATTCAACAAGCACTTACTGGGAAGGTCTACACCTGCATAGGC  165

Query  136  AATGCTGGAAAAAGGGTTAAGTAAACCAGGACATGACAATGGTGGCAAATGACTATCAGG  195
            ||||||||||||||||||||||||||||||||||||||||||||||||||||||||||||
Sbjct  166  AATGCTGGAAAAAGGGTTAAGTAAACCAGGACATGACAATGGTGGCAAATGACTATCAGG  225

Query  196  TCTTCCCATGTGTTTGACTCAAACTTATTACCCTATGGTCCTTCTGACAATGGCAGAAGG  255
            ||||||||||||||||||||||||||||||||||||||||||||||||||||||||||||
Sbjct  226  TCTTCCCATGTGTTTGACTCAAACTTATTACCCTATGGTCCTTCTGACAATGGCAGAAGG  285

Query  256  TCTGAATC  263
            ||||||||
Sbjct  286  TCTGAATC  293
```

SNP 27

```
Sequence ID: Query_189057  Length: 505  Number of Matches: 1

Range 1: 43 to 505  Graphics                                   ▼ Next Match  ▲ Pre

Score           Expect     Identities        Gaps          Strand
785 bits(425)   0.0        453/464(98%)      11/464(2%)    Plus/Plus

Query  12   AAGC-AGACTTTGTCTC-aaaaaaaCATGTATAGCTACATAATTAATAATATGCAAATAT  69
            |||| |||||||||||| ||||||||||||||||||||||||||||||||||||||||||
Sbjct  43   AAGCAAGACTTTGTCTCAAAAAAAACATGTATAGCTACATAATTAATAATATGCAAATAT  102

Query  70   TCTTCTTTAGGGTCACTTAGGTTTTCAACATACAGACCCAAGAGAAGGTACAGAGACTCA  129
            ||||||||||||||||||||||||||||||||||||||||||||||||||||||||||||
Sbjct  103  TCTTCTTTAGGGTCACTTAGGTTTTCAACATACAGACCCAAGAGAAGGTACAGAGACTCA  162

Query  130  TTCTCTCTCTCTTTCAAT--------cacacacacacacacacacacacacacacacaca  181
            ||||||||||||||||||        ||||||||||||||||||||||||||||||||||
Sbjct  163  TTCTCTCTCTCTTTCAATCACACACACACACACACACACACACACACACACACACACACA  222

Query  182  cCCCTCTTCACTATAATTATAATTACTATGTTGGCTTCCACGATCAGGGGTTAGAGCCTT  241
            |||||||||||||||||||||||||||||||||||||||| |||||||||||||||||||
Sbjct  223  CCCCTCTTCACTATAATTATAATTACTATGTTGGCTTCCA-GATCAGGGGTTAGAGCCTT  281

Query  242  GGCATGGAGACGCCTGAAAGGCACCCAAGGCAATTAGTGGTGTCCCTTCTCCACCCCCTA  301
            ||||||||||||||||||||||||||||||||||||||||||||||||||||||||||||
Sbjct  282  GGCATGGAGACGCCTGAAAGGCACCCAAGGCAATTAGTGGTGTCCCTTCTCCACCCCCTA  341

Query  302  CATACCTTCAGGCCCTTTGCACTTGCTGCTCCCCCTCCCAGTGCACACACCCCCAGATCC  361
            ||||||||||||||||||||||||||||||||||||||||||||||||||||||||||||
Sbjct  342  CATACCTTCAGGCCCTTTGCACTTGCTGCTCCCCCTCCCAGTGCACACACCCCCAGATCC  401

Query  362  TTGTGTGGCTCCCTCTCCCACCACATTTTGGTCTTAGCTCAAATGCCACCTCCTCAGAAA  421
            ||||||||||||||||||||||||||||||||||||||||||||||||||||||||||||
Sbjct  402  TTGTGTGGCTCCCTCTCCCACCACATTTTGGTCTTAGCTCAAATGCCACCTCCTCAGAAA  461

Query  422  TGCCTGGCCTGGCCGCCTTCAGGGCTGTCTATATGCTCACCAGC  465
            ||||||||||||||||||||||||||||||||||||||||||||
Sbjct  462  TGCCTGGCCTGGCCGCCTTCAGGGCTGTCTATATGCTCACCAGC  505
```

SNP 29

```
Sequence ID: Query_52545  Length: 384  Number of Matches: 1

Range 1: 54 to 384  Graphics                                   ▼ Next Match

Score           Expect     Identities        Gaps          Strand
603 bits(326)   3e-177     331/333(99%)      2/333(0%)     Plus/Plus

Query  2    CACGTATCCCAGAACTTAAAGTATAATTTTAAAAAAGATGTTTTATAATCTAATTGGTGA  61
            ||||||||||||||||||||||||||||||||||||||||||||||||||||||||||||
Sbjct  54   CACGTATCCCAGAACTTAAAGTATAATTTTAAAAAAGATGTTTTATAATCTAATTGGTGA  113

Query  62   CTGACAACTAAGGTACGTTAGACCATTAGCAAAAGTAAATTAAGTGATAAGTATTATGGT  121
            ||||||||||||||||||||||||||||||||||||||||||||||||||||||||||||
Sbjct  114  CTGACAACTAAGGTACGTTAGACCATTAGCAAAAGTAAATTAAGTGATAAGTATTATGGT  173

Query  122  AGGGACCTCAGGTAGATATAGGAATTGGGCAGAGAGAGACGTTAACATGGTTAACTCCAA  181
            |||||||||||||| |||||||||||||||||||||||||||||||||||||||||||||
Sbjct  174  AGGGACCTCAGGTA-ATATAGGAATTGGGCAGAGAGAGACGTTAACATGGTTAACTCCAA  232

Query  182  AAGTGGTATGACCTAGAAAATGGGTAGATAGGTTTGAGTAAGTGGAAGGAGAGAAAAGAA  241
            ||||||||||||||||||||||||||||||||||||||||||||||||||||||||||||
Sbjct  233  AAGTGGTATGACCTAGAAAATGGGTAGATAGGTTTGAGTAAGTGGAAGGAGAGAAAAGAA  292

Query  242  ATTTCTAGGCATAGACGCAGTGTTTCTCAAGAAACTGAAAGACAAATATGCAGGTCCTGC  301
            ||||||||||||||||||||||||||||||||||||||||||||||||||||||||||||
Sbjct  293  ATTTCTAGGCATAGACGCAGTGTTTCTCAAGAAACTGAAAGACAAATATGCAGGTCCTGC  352

Query  302  TTATGGAAAAGGGAAGACAGAAGGGTGTCTTGC  334
            |||||||||||||||||||||||| ||||||||
Sbjct  353  TTATGGAAAAGGGAAGACAGAAGG-TGTCTTGC  384
```

SNP 46

```
Sequence ID: Query_24169  Length: 551  Number of Matches: 1

Range 1: 42 to 543 Graphics                                        ▼ Next Match ▲

Score            Expect   Identities      Gaps         Strand
900 bits(487)    0.0      498/503(99%)    2/503(0%)    Plus/Plus

Query  9    AAAATCAAGGAATCCAAATAAAATACCACGTTTAATTCAATTTATCACGTCCAAGAAGTT  68
            ||||||||| ||||||||||||||||||||||||||||||||||||||||||||||||||
Sbjct  42   AAAATCAAGAAATCCAAATAAAATACCACGTTTAATTCAATTTATCACGTCCAAGAAGTT  101

Query  69   ACAAAGGCAATGTAGAAGAAGTAAGTAAAAATGGGGAAGCCCTAATTTTTAAGTCTGCCT  128
            ||||||||||||||||||||||||||||||||||||||||||||||||||||||||||||
Sbjct  102  ACAAAGGCAATGTAGAAGAAGTAAGTAAAAATGGGGAAGCCCTAATTTTTAAGTCTGCCT  161

Query  129  TGAAAACAATTTTTTCTTATCAATGAGACTTTCTAATAGGACTCATCTTGCCATTGGCAC  188
            ||||||||||||||||||||||||||||||||||||||||||||||||||||||||||||
Sbjct  162  TGAAAACAATTTTTTCTTATCAATGAGACTTTCTAATAGGACTCATCTTGCCATTGGCAC  221

Query  189  CCACAAAATCTAACCACCCACCTCCACTTCTCCGGAGAGGATCCCTTCCTAATGTCTGAT  248
            ||||||||||||||||||||||||||||||||||| ||||||||||||||||||||||||
Sbjct  222  CCACAAAATCTAACCACCCACCTCCACTTCTCCGG-GAGGATCCCTTCCTAATGTCTGAT  280

Query  249  GCCTCTTCCCTCACCTCCATTCTTTGCCCACTGCTGCACATGCTTAAAGCCCAGCCACCC  308
            ||||||||||||||||||||||||||||||||||||||||||||||||||||||||||||
Sbjct  281  GCCTCTTCCCTCACCTCCATTCTTTGCCCACTGCTGCACATGCTTAAAGCCCAGCCACCC  340

Query  309  TTTCACCCACAGAGGTCATAGACCACTGCTACTATGGTTAATTCTCAGAAGTAATAAACT  368
            ||||||||||||||||||||||||||||||||||||||||||||||||||||||||||||
Sbjct  341  TTTCACCCACAGAGGTCATAGACCACTGCTACTATGGTTAATTCTCAGAAGTAATAAACT  400

Query  369  CAGAACCTGAAAGCACATAATGCCTACAAGACTGTTCACTATTTCAAACTTCTGCTCTGG  428
            ||||||||||||||||||||||||||||||||||||||||||||||||||||||||||||
Sbjct  401  CAGAACCTGAAAGCACATAATGCCTACAAGACTGTTCACTATTTCAAACTTCTGCTCTGG  460

Query  429  TTTACATGAATT-aaaaaaaaaaaaaTCACTCTATTTTTTCTGCTGTACCAAGGCaaaaa  487
            |||||||||||| |||||||||||||||||||||||||||||||||||||||||||||||
Sbjct  461  TTTACATGAATTAAAAAAAAAAAAAAATCACTCTATTTTTTCTGCTGTACCAAGGCAAAAA  520

Query  488  aaTTCTACAATCCCTTTTAAAGG  510
            |||||||||||| | ||||||||
Sbjct  521  AATTCTACAATCACATTTAAAGG  543
```

SNP 49

```
Sequence ID: Query_17083  Length: 439  Number of Matches: 1

Range 1: 41 to 439 Graphics                                        ▼ Next Match ▲

Score            Expect   Identities      Gaps         Strand
699 bits(378)    0.0      394/401(98%)    4/401(0%)    Plus/Plus

Query  5    AAA-GCAGAGCCTGCTTTGGTTG-AGCGAAGATGGACGCTTGAATGCCCTTTCGTACTA  62
            ||| |||| | |||||||||||| ||||||||||||||||||||||||||||||||||||
Sbjct  41   AAACGCAG-GGCTGCTTTGGTTGAAGCGAAGATGGACGCTTGAATGCCCTTTCGTACTA  99

Query  63   CTCCCCTTTTACCTCGTTAATACCCACTGACCTATCCTCGTGGAATGCAGGGCTCAAAGA  122
            ||||||||||||||||||||||||||||||||||||||||||||||||||||||||||||
Sbjct  100  CTCCCCTTTTACCTCGTTAATACCCACTGACCTATCCTCGTGGAATGCAGGGCTCAAAGA  159

Query  123  ACAATCTAAAAATCAAACATTATACAAATGCAACCTAAGGAGGAGAGTTCCTTTGAGGCC  182
            ||||||||||||||||||||||||||||||||||||||||||||||||||||||||||||
Sbjct  160  ACAATCTAAAAATCAAACATTATACAAATGCAACCTAAGGAGGAGAGTTCCTTTGAGGCC  219

Query  183  AGGGACTACATTATCTTATCTGTATTGCCAGCGCAGAGGCCTACTAGTACATTGTAGGGT  242
            |||| |||||||||||||||||||||||||||||||||||||||||||||||||||||||
Sbjct  220  AGGGGCTACATTATCTTATCTGTATTGCCAGCGCAGAGGCCTACTAGTACATTGTAGGGT  279

Query  243  CTAAGTACATTTTTCCTGAATGAAAGGTATTAAATGGTAACTTACGTCTTTATGCACTCT  302
            ||||||||||||||||||||||||||||||||||||||||||||||||||||||||||||
Sbjct  280  CTAAGTACATTTTTCCTGAATGAAAGGTATTAAATGGTAACTTACGTCTTTATGCACTCT  339

Query  303  ATAAACTATGACGTGATCGTCTCCGTCTAACAACTACACTCAAATGCTTACCAAGCTCTT  362
            ||||||||||||||||||||||||||||||||||||||||||||||||||||||||||||
Sbjct  340  ATAAACTATGACGTGATCGTCTCCGTCTAACAACTACACTCAAATGCTTACCAAGCTCTT  399

Query  363  TAAAGGGAAGAATTCCATGGTCGTATGAGCTTTTCAACAGT  403
            |||||||||||||||||||||||||||||| || |||||||
Sbjct  400  TAAAGGGAAGAATTCCATGGTCGTATGAGCATT-CAACAGT  439
```

Sample 5

SNP 10

Sequence ID: **Query_298443** **Length:** **293** **Number of Matches:** **1**

Range 1: 47 to 293 Graphics ▼ Next Match ▲

Score	Expect	Identities	Gaps	Strand
457 bits(247)	2e-133	247/247(100%)	0/247(0%)	Plus/Plus

```
Query  19   TCTTCTGAGTGTTACCCAGTCAAGTATAAGTAGCCAAATTATTTTTGCACATCTTTCTGT  78
            ||||||||||||||||||||||||||||||||||||||||||||||||||||||||||||
Sbjct  47   TCTTCTGAGTGTTACCCAGTCAAGTATAAGTAGCCAAATTATTTTTGCACATCTTTCTGT  106

Query  79   TTCTCATGTCTTCATTTATTCAACAAGCACTTACTGGGAAGGTCTACACCTGCATAGGCA  138
            ||||||||||||||||||||||||||||||||||||||||||||||||||||||||||||
Sbjct  107  TTCTCATGTCTTCATTTATTCAACAAGCACTTACTGGGAAGGTCTACACCTGCATAGGCA  166

Query  139  ATGCTGGAAAAAGGGTTAAGTAAACCAGGACATGACAATGGTGGCAAATGACTATCAGGT  198
            ||||||||||||||||||||||||||||||||||||||||||||||||||||||||||||
Sbjct  167  ATGCTGGAAAAAGGGTTAAGTAAACCAGGACATGACAATGGTGGCAAATGACTATCAGGT  226

Query  199  CTTCCCATGTGTTTGACTCAAACTTATTACCCTATGGTCCTTCTGACAATGGCAGAAGGT  258
            ||||||||||||||||||||||||||||||||||||||||||||||||||||||||||||
Sbjct  227  CTTCCCATGTGTTTGACTCAAACTTATTACCCTATGGTCCTTCTGACAATGGCAGAAGGT  286

Query  259  CTGAATC  265
            |||||||
Sbjct  287  CTGAATC  293
```

SNP 27

Sequence ID: **Query_51991** **Length:** **505** **Number of Matches:** **1**

Range 1: 43 to 505 Graphics ▼ Next Match ▲

Score	Expect	Identities	Gaps	Strand
791 bits(428)	0.0	453/463(98%)	10/463(2%)	Plus/Plus

```
Query  9    AAGC-AGACTTTGTCTC-aaaaaaaCATGTATAGCTACATAATTAATAATATGCAAATAT  66
            |||| |||||||||||| ||||||||||||||||||||||||||||||||||||||||||
Sbjct  43   AAGCAAGACTTTGTCTCAAAAAAAACATGTATAGCTACATAATTAATAATATGCAAATAT  102

Query  67   TCTTCTTTAGGGTCACTTAGGTTTTCAACATACAGACCCAAGAGAAGGTACAGAGACTCA  126
            ||||||||||||||||||||||||||||||||||||||||||||||||||||||||||||
Sbjct  103  TCTTCTTTAGGGTCACTTAGGTTTTCAACATACAGACCCAAGAGAAGGTACAGAGACTCA  162

Query  127  TTCTCTCTCTCTTTCAAT--------cacacacacacacacacacacacacacacacaca  178
            ||||||||||||||||||        ||||||||||||||||||||||||||||||||||
Sbjct  163  TTCTCTCTCTCTTTCAATCACACACACACACACACACACACACACACACACACACACACA  222

Query  179  cCCCTCTTCACTATAATTATAATTACTATGTTGGCTTCCAGATCAGGGGTTAGAGCCTTG  238
            ||||||||||||||||||||||||||||||||||||||||||||||||||||||||||||
Sbjct  223  CCCCTCTTCACTATAATTATAATTACTATGTTGGCTTCCAGATCAGGGGTTAGAGCCTTG  282

Query  239  GCATGGAGACGCCTGAAAGGCACCCAAGGCAATTAGTGGTGTCCCTTCTCCACCCCCTAC  298
            ||||||||||||||||||||||||||||||||||||||||||||||||||||||||||||
Sbjct  283  GCATGGAGACGCCTGAAAGGCACCCAAGGCAATTAGTGGTGTCCCTTCTCCACCCCCTAC  342

Query  299  ATACCTTCAGGCCCTTTGCACTTGCTGCTCCCCCTCCCAGTGCACACACCCCCAGATCCT  358
            ||||||||||||||||||||||||||||||||||||||||||||||||||||||||||||
Sbjct  343  ATACCTTCAGGCCCTTTGCACTTGCTGCTCCCCCTCCCAGTGCACACACCCCCAGATCCT  402

Query  359  TGTGTGGCTCCCTCTCCCACCACATTTTGGTCTTAGCTCAAATGCCACCTCCTCAGAAAT  418
            ||||||||||||||||||||||||||||||||||||||||||||||||||||||||||||
Sbjct  403  TGTGTGGCTCCCTCTCCCACCACATTTTGGTCTTAGCTCAAATGCCACCTCCTCAGAAAT  462

Query  419  GCCTGGCCTGGCCGCCTTCAGGGCTGTCTATATGCTCACCAGC  461
            |||||||||||||||||||||||||||||||||||||||||||
Sbjct  463  GCCTGGCCTGGCCGCCTTCAGGGCTGTCTATATGCTCACCAGC  505
```

SNP 29

Sequence ID: **Query_53473** **Length:** **384** **Number of Matches:** **1**

Range 1: 36 to 384 Graphics ▼ Next Match ▲

Score	Expect	Identities	Gaps	Strand
632 bits(342)	0.0	347/349(99%)	1/349(0%)	Plus/Plus

```
Query  3    AAACCTG-ACATTCTGCACACGTATCCCAGAACTTAAAGTATAATTTTAAAAAAGATGTT  61
            |||||||  |||||||||||||||||||||||||||||||||||||||||||||||||||
Sbjct  36   AAACCTGCCCATTCTGCACACGTATCCCAGAACTTAAAGTATAATTTTAAAAAAGATGTT  95

Query  62   TTATAATCTAATTGGTGACTGACAACTAAGGTACGTTAGACCATTAGCAAAAGTAAATTA  121
            ||||||||||||||||||||||||||||||||||||||||||||||||||||||||||||
Sbjct  96   TTATAATCTAATTGGTGACTGACAACTAAGGTACGTTAGACCATTAGCAAAAGTAAATTA  155

Query  122  AGTGATAAGTATTATGGTAGGGACCTCAGGTAATATAGGAATTGGGCAGAGAGAGACGTT  181
            ||||||||||||||||||||||||||||||||||||||||||||||||||||||||||||
Sbjct  156  AGTGATAAGTATTATGGTAGGGACCTCAGGTAATATAGGAATTGGGCAGAGAGAGACGTT  215

Query  182  AACATGGTTAACTCCAAAAGTGGTATGACCTAGAAAATGGGTAGATAGGTTTGAGTAAGT  241
            ||||||||||||||||||||||||||||||||||||||||||||||||||||||||||||
Sbjct  216  AACATGGTTAACTCCAAAAGTGGTATGACCTAGAAAATGGGTAGATAGGTTTGAGTAAGT  275

Query  242  GGAAGGAGAGAAAAGAAATTTCTAGGCATAGACGCAGTGTTTCTCAAGAAACTGAAAGAC  301
            ||||||||||||||||||||||||||||||||||||||||||||||||||||||||||||
Sbjct  276  GGAAGGAGAGAAAAGAAATTTCTAGGCATAGACGCAGTGTTTCTCAAGAAACTGAAAGAC  335

Query  302  AAATATGCAGGTCCTGCTTATGGAAAAGGGAAGACAGAAGGTGTCTTGC  350
            |||||||||||||||||||||||||||||||||||||||||||||||||
Sbjct  336  AAATATGCAGGTCCTGCTTATGGAAAAGGGAAGACAGAAGGTGTCTTGC  384
```

SNP 46

Sequence ID: **Query_13145** **Length:** **551** **Number of Matches:** **1**

Range 1: 45 to 543 Graphics ▼ Next Match ▲ P

Score	Expect	Identities	Gaps	Strand
830 bits(449)	0.0	484/500(97%)	5/500(1%)	Plus/Plus

```
Query  12     ATC-AG-AATCC-AATAAAATACCACGTTTAATTCAATTTATCACGTCCAAGAAGTTACA  68
              ||| || ||||| ||||||||||||||||||||||||||||||||||||||||||||||||
Sbjct  45     ATCAAGAAATCCAAATAAAATACCACGTTTAATTCAATTTATCACGTCCAAGAAGTTACA  104

Query  69     AAGGCAATGTAGAAGAAGTAAGTAAAAATGGGGAAGCCCTAATTTTTAAGTCTGCCTTGA  128
              ||||||||||||||||||||||||||||||||||||||||||||||||||||||||||||
Sbjct  105    AAGGCAATGTAGAAGAAGTAAGTAAAAATGGGGAAGCCCTAATTTTTAAGTCTGCCTTGA  164

Query  129    AAACAATTTTTTCTTATCAATGAGACTTTCTAATAGGACTCATCTTGCCATTGGCACCCA  188
              ||||||||||||||||||||||||||||||||||||||||||||||||||||||||||||
Sbjct  165    AAACAATTTTTTCTTATCAATGAGACTTTCTAATAGGACTCATCTTGCCATTGGCACCCA  224

Query  189    CAAAATCTAACCACCCACCTCCACTTCTCCGGAGAGGATCCCTTCCTAATGTCTGATGCC  248
              |||||||||||||||||||||||||||||||| |||||||||||||||||||||||||||
Sbjct  225    CAAAATCTAACCACCCACCTCCACTTCTCCGG-GAGGATCCCTTCCTAATGTCTGATGCC  283

Query  249    TCTTCCCTCACCTCCATTCTTTGCCCACTGCTGCACATGCTTAAAGCCCAGCCACCCTTT  308
              ||||||||||||||||||||||||||||||||||||||||||||||||||||||||||||
Sbjct  284    TCTTCCCTCACCTCCATTCTTTGCCCACTGCTGCACATGCTTAAAGCCCAGCCACCCTTT  343

Query  309    CACCCACAGAGGTCATAGACCACTGCTACTATGGTTAATTCTCAGAAGTAATAAACTCAG  368
              ||||||||||||||||||||||||||||||||||||||||||||||||||||||||||||
Sbjct  344    CACCCACAGAGGTCATAGACCACTGCTACTATGGTTAATTCTCAGAAGTAATAAACTCAG  403

Query  369    AACCTGAAAGCACATAATGCCTACAAGACTGTTCACTATTTCAAACTTCTGCTCTGGTTT  428
              ||||||||||||||||||||||||||||||||||||||||||||||||||||||||||||
Sbjct  404    AACCTGAAAGCACATAATGCCTACAAGACTGTTCACTATTTCAAACTTCTGCTCTGGTTT  463

Query  429    ACATGAATTaaaaaaaaaaaaaaCCCC-CttttttttttGGTGGACCCAGGGaaaaaaaT  487
              ||||||||||||||||||||||| | | || |||||| || || ||| ||| ||||||||
Sbjct  464    ACATGAATTAAAAAAAAAAAAAATCACTCTATTTTTTCTGCTGTACCAAGGCAAAAAAAT  523

Query  488    TTTACAATCCCCTTTAAAGG  507
              | ||||||| | ||||||||
Sbjct  524    TCTACAATCACATTTAAAGG  543
```

SNP 49

Sequence ID: **Query_40767** **Length:** **439** **Number of Matches:** **1**

Range 1: 50 to 439 Graphics ▼ Next Match ▲

Score	Expect	Identities	Gaps	Strand
704 bits(381)	0.0	388/391(99%)	2/391(0%)	Plus/Plus

```
Query  15     GCTGCTTTGGTTG-AGCGAAGATGGGACGCTTGAATGCCCTTTCGTACTACTCCCCTTTT  73
              ||||||||||||| ||||||||||||||||||||||||||||||||||||||||||||||
Sbjct  50     GCTGCTTTGGTTGAAGCGAAGATGGGACGCTTGAATGCCCTTTCGTACTACTCCCCTTTT  109

Query  74     ACCTCGTTAATACCCACTGACCTATCCTCGTGGAATGCAGGGCTCAAAGAACAATCTAAA  133
              ||||||||||||||||||||||||||||||||||||||||||||||||||||||||||||
Sbjct  110    ACCTCGTTAATACCCACTGACCTATCCTCGTGGAATGCAGGGCTCAAAGAACAATCTAAA  169

Query  134    AATCAAACATTATACAAATGCAACCTAAGGAGGAGAGTTCCTTTGAGGCCAGGGAGCTAC  193
              |||||||||||||||||||||||||||||||||||||||||||||||||||||| |||||
Sbjct  170    AATCAAACATTATACAAATGCAACCTAAGGAGGAGAGTTCCTTTGAGGCCAGGG-GCTAC  228

Query  194    ATTATCTTATCTGTATTGCCAGCGCAGAGGCCTACTAGTACATTGTAGGGTCTAAGTACA  253
              ||||||||||||||||||||||||||||||||||||||||||||||||||||||||||||
Sbjct  229    ATTATCTTATCTGTATTGCCAGCGCAGAGGCCTACTAGTACATTGTAGGGTCTAAGTACA  288

Query  254    TTTTTCCTGAATGAAAGGTATTAAATGGTAACTTACGTCTTTATGCACTCTATAAACTAT  313
              ||||||||||||||||||||||||||||||||||||||||||||||||||||||||||||
Sbjct  289    TTTTTCCTGAATGAAAGGTATTAAATGGTAACTTACGTCTTTATGCACTCTATAAACTAT  348

Query  314    GACGTGATCGTCTCCGTCTAACAACTACACTCAAATGCTTACCAAGCTCTTTAAAGGGAA  373
              ||||||||||||||||||||||||||||||||||||||||||||||||||||||||||||
Sbjct  349    GACGTGATCGTCTCCGTCTAACAACTACACTCAAATGCTTACCAAGCTCTTTAAAGGGAA  408

Query  374    GAATTCCATGGTCGTATGAGCTTTCAACAGT  404
              ||||||||||||||||||||| |||||||||
Sbjct  409    GAATTCCATGGTCGTATGAGCATTCAACAGT  439
```

Sample 6

SNP 10

```
Sequence ID: Query_122731  Length: 293  Number of Matches: 1

Range 1: 40 to 293  Graphics                                  ▼ Next Match  ▲ P

Score            Expect     Identities       Gaps          Strand
455 bits(246)    6e-133     253/256(99%)     2/256(0%)     Plus/Plus

Query  8    CCAGTCTTATCTTCTGAGTGTTACCCAGTCAAGTATAAGTAGCCAAATTATTTTTGCACA  67
            ||| |||| |||||||||||||||||||||||||||||||||||||||||||||||||||
Sbjct  40   CCA-TCTT-TCTTCTGAGTGTTACCCAGTCAAGTATAAGTAGCCAAATTATTTTTGCACA  97

Query  68   TCTTTCTGTTTCTCATGTCTTCATTTATTCAACAAGCACTTACTGGGAAGGTCTACAACT  127
            ||||||||||||||||||||||||||||||||||||||||||||||||||||||||| ||
Sbjct  98   TCTTTCTGTTTCTCATGTCTTCATTTATTCAACAAGCACTTACTGGGAAGGTCTACACCT  157

Query  128  GCATAGGCAATGCTGGAAAAAGGGTTAAGTAAACCAGGACATGACAATGGTGGCAAATGA  187
            ||||||||||||||||||||||||||||||||||||||||||||||||||||||||||||
Sbjct  158  GCATAGGCAATGCTGGAAAAAGGGTTAAGTAAACCAGGACATGACAATGGTGGCAAATGA  217

Query  188  CTATCAGGTCTTCCCATGTGTTTGACTCAAACTTATTACCCTATGGTCCTTCTGACAATG  247
            ||||||||||||||||||||||||||||||||||||||||||||||||||||||||||||
Sbjct  218  CTATCAGGTCTTCCCATGTGTTTGACTCAAACTTATTACCCTATGGTCCTTCTGACAATG  277

Query  248  GCAGAAGGTCTGAATC  263
            ||||||||||||||||
Sbjct  278  GCAGAAGGTCTGAATC  293
```

SNP 27

```
Sequence ID: Query_27579  Length: 505  Number of Matches: 1

Range 1: 43 to 505  Graphics                                  ▼ Next Match  ▲ Prev

Score            Expect     Identities       Gaps          Strand
784 bits(424)    0.0        452/463(98%)     11/463(2%)    Plus/Plus

Query  12   AAGC-AGACTTTGTCTC--AAAAAACATGTATAGCTACATAATTAATAATATGCAAATAT  68
            |||| ||||||||||||  |||||||||||||||||||||||||||||||||||||||||
Sbjct  43   AAGCAAGACTTTGTCTCAAAAAAAACATGTATAGCTACATAATTAATAATATGCAAATAT  102

Query  69   TCTTCTTTAGGGTCACTTAGGTTTTCAACATACAGACCCAAGAGAAGGTACAGAGACTCA  128
            ||||||||||||||||||||||||||||||||||||||||||||||||||||||||||||
Sbjct  103  TCTTCTTTAGGGTCACTTAGGTTTTCAACATACAGACCCAAGAGAAGGTACAGAGACTCA  162

Query  129  TTCTCTCTCTCTTTCAAT--------cacacacacacacacacacacacacacacacaca  180
            ||||||||||||||||||        ||||||||||||||||||||||||||||||||||
Sbjct  163  TTCTCTCTCTCTTTCAATCACACACACACACACACACACACACACACACACACACACACA  222

Query  181  cCCCTCTTCACTATAATTATAATTACTATGTTGGCTTCCAGATCAGGGGTTAGAGCCTTG  240
            ||||||||||||||||||||||||||||||||||||||||||||||||||||||||||||
Sbjct  223  CCCCTCTTCACTATAATTATAATTACTATGTTGGCTTCCAGATCAGGGGTTAGAGCCTTG  282

Query  241  GCATGGAGACGCCTGAAAGGCACCCAAGGCAATTAGTGGTGTCCCTTCTCCACCCCCTAC  300
            ||||||||||||||||||||||||||||||||||||||||||||||||||||||||||||
Sbjct  283  GCATGGAGACGCCTGAAAGGCACCCAAGGCAATTAGTGGTGTCCCTTCTCCACCCCCTAC  342

Query  301  ATACCTTCAGGCCCTTTGCACTTGCTGCTCCCCCTCCCAGTGCACACACCCCCAGATCCT  360
            ||||||||||||||||||||||||||||||||||||||||||||||||||||||||||||
Sbjct  343  ATACCTTCAGGCCCTTTGCACTTGCTGCTCCCCCTCCCAGTGCACACACCCCCAGATCCT  402

Query  361  TGTGTGGCTCCCTCTCCCACCACATTTTGGTCTTAGCTCAAATGCCACCTCCTCAGAAAT  420
            ||||||||||||||||||||||||||||||||||||||||||||||||||||||||||||
Sbjct  403  TGTGTGGCTCCCTCTCCCACCACATTTTGGTCTTAGCTCAAATGCCACCTCCTCAGAAAT  462

Query  421  GCCTGGCCTGGCCGCCTTCAGGGCTGTCTATATGCTCACCAGC  463
            |||||||||||||||||||||||||||||||||||||||||||
Sbjct  463  GCCTGGCCTGGCCGCCTTCAGGGCTGTCTATATGCTCACCAGC  505
```

SNP 29

```
Sequence ID: Query_9933  Length: 384  Number of Matches: 1

Range 1: 36 to 383  Graphics                                  ▼ Next Match  ▲ Pr

Score            Expect     Identities       Gaps          Strand
614 bits(332)    2e-180     344/349(99%)     3/349(0%)     Plus/Plus

Query  1    AAAACTG-CCATTCTGCACACGTATCCCAGAACTTAAAGTATAATTTTAAAAAAGATGTT  59
            ||| ||| ||||||||||||||||||||||||||||||||||||||||||||||||||||
Sbjct  36   AAACCTGCCCATTCTGCACACGTATCCCAGAACTTAAAGTATAATTTTAAAAAAGATGTT  95

Query  60   TTATAATCTAATTGGTGACTGACAACTAAGGTACGTTAGACCATTAGCAAAAGTAAATTA  119
            ||||||||||||||||||||||||||||||||||||||||||||||||||||||||||||
Sbjct  96   TTATAATCTAATTGGTGACTGACAACTAAGGTACGTTAGACCATTAGCAAAAGTAAATTA  155

Query  120  AGTGATAAGTATTATGGTAGGGACCTCAGGTAGATATAGGAATTGGGCAGAGAGAGACGT  179
            |||||||||||||||||||||||||||||||| |||||||||||||||||||||||||||
Sbjct  156  AGTGATAAGTATTATGGTAGGGACCTCAGGTA-ATATAGGAATTGGGCAGAGAGAGACGT  214

Query  180  TAACATGGTTAACTCCAAAAGTGGTATGACCTAGAAAATGGGTAGATAGGTTTGAGTAAG  239
            ||||||||||||||||||||||||||||||||||||||||||||||||||||||||||||
Sbjct  215  TAACATGGTTAACTCCAAAAGTGGTATGACCTAGAAAATGGGTAGATAGGTTTGAGTAAG  274

Query  240  TGGAAGGAGAGAAAAGAAATTTCTAGGCATAGACGCAGTGTTTCTCAAGAAACTGAAAGA  299
            ||||||||||||||||||||||||||||||||||||||||||||||||||||||||||||
Sbjct  275  TGGAAGGAGAGAAAAGAAATTTCTAGGCATAGACGCAGTGTTTCTCAAGAAACTGAAAGA  334

Query  300  CAAATATGCAGGTCCTGCTTATGGAAAAGGGAAGACAGAAT-TGTCTTG  347
            ||||||||||||||||||||||||||||||||||||||||  |||||||
Sbjct  335  CAAATATGCAGGTCCTGCTTATGGAAAAGGGAAGACAGAAGGTGTCTTG  383
```

SNP 46

Sequence ID: **Query_64865** Length: **551** Number of Matches: **1**

Range 1: 43 to 548 Graphics ▼ Next Match ▲

Score	Expect	Identities	Gaps	Strand
898 bits(486)	0.0	500/506(99%)	4/506(0%)	Plus/Plus

```
Query  10   AAATC-AG-AATCCAAATAAAATACCACGTTTAATTCAATTTATCACGTCCAAGAAGTTA  67
            ||||| || |||||||||||||||||||||||||||||||||||||||||||||||||||
Sbjct  43   AAATCAAGAAATCCAAATAAAATACCACGTTTAATTCAATTTATCACGTCCAAGAAGTTA  102

Query  68   CAAAGGCAATGTAGAAGAAGTAAGTAAAAATGGGGAAGCCCTAATTTTTAAGTCTGCCTT  127
            ||||||||||||||||||||||||||||||||||||||||||||||||||||||||||||
Sbjct  103  CAAAGGCAATGTAGAAGAAGTAAGTAAAAATGGGGAAGCCCTAATTTTTAAGTCTGCCTT  162

Query  128  GAAAACAATTTTTTCTTATCAATGAGACTTTCTAATAGGACTCATCTTGCCATTGGCACC  187
            ||||||||||||||||||||||||||||||||||||||||||||||||||||||||||||
Sbjct  163  GAAAACAATTTTTTCTTATCAATGAGACTTTCTAATAGGACTCATCTTGCCATTGGCACC  222

Query  188  CACAAAATCTAACCACCCACCTCCACTTCTCCGGGAGGATCCCTTCCTAATGTCTGATGC  247
            ||||||||||||||||||||||||||||||||||||||||||||||||||||||||||||
Sbjct  223  CACAAAATCTAACCACCCACCTCCACTTCTCCGGGAGGATCCCTTCCTAATGTCTGATGC  282

Query  248  CTCTTCCCTCACCTCCATTCTTTGCCCACTGCTGCACATGCTTAAAGCCCAGCCACCCTT  307
            ||||||||||||||||||||||||||||||||||||||||||||||||||||||||||||
Sbjct  283  CTCTTCCCTCACCTCCATTCTTTGCCCACTGCTGCACATGCTTAAAGCCCAGCCACCCTT  342

Query  308  TCACCCACAGAGGTCATAGACCACTGCTACTATGGTTAATTCTCAGAAGTAATAAACTCA  367
            ||||||||||||||||||||||||||||||||||||||||||||||||||||||||||||
Sbjct  343  TCACCCACAGAGGTCATAGACCACTGCTACTATGGTTAATTCTCAGAAGTAATAAACTCA  402

Query  368  GAACCTGAAAGCACATAATGCCTACAAGACTGTTCACTATTTCAAACTTCTGCTCTGGTT  427
            ||||||||||||||||||||||||||||||||||||||||||||||||||||||||||||
Sbjct  403  GAACCTGAAAGCACATAATGCCTACAAGACTGTTCACTATTTCAAACTTCTGCTCTGGTT  462

Query  428  TACATGAATT-aaaaaaaaaaaaaTCACTCTATTTTTTCTGCTGTACCAAGGCaaaaaaa  486
            |||||||||| |||||||||||||||||||||||||||||||||||||||||||||||||
Sbjct  463  TACATGAATTAAAAAAAAAAAAAATCACTCTATTTTTTCTGCTGTACCAAGGCAAAAAAA  522

Query  487  TTCTACAATCCCTTTTAAAGGC-TGG  511
            |||||||||| | ||||||||| |||
Sbjct  523  TTCTACAATCACATTTAAAGGCATGG  548
```

SNP 49

Sequence ID: **Query_59213** Length: **439** Number of Matches: **1**

Range 1: 40 to 439 Graphics ▼ Next Match ▲

Score	Expect	Identities	Gaps	Strand
715 bits(387)	0.0	399/404(99%)	4/404(0%)	Plus/Plus

```
Query  6    AAAAAGCCAGAGGCTGCCTTTGGTTGAAGCGAAGATGGGACGCTTGAATGCCCTTTCGTA  65
            |||| | ||| ||||| |||||||||||||||||||||||||||||||||||||||||||
Sbjct  40   AAAACG-CAG-GGCTG-CTTTGGTTGAAGCGAAGATGGGACGCTTGAATGCCCTTTCGTA  96

Query  66   CTACTCCCCTTTTACCTCGTTAATACCCACTGACCTATCCTCGTGGAATGCAGGGCTCAA  125
            ||||||||||||||||||||||||||||||||||||||||||||||||||||||||||||
Sbjct  97   CTACTCCCCTTTTACCTCGTTAATACCCACTGACCTATCCTCGTGGAATGCAGGGCTCAA  156

Query  126  AGAACAATCTAAAAATCAAACATTATACAAATGCAACCTAAGGAGGAGAGTTCCTTTGAG  185
            ||||||||||||||||||||||||||||||||||||||||||||||||||||||||||||
Sbjct  157  AGAACAATCTAAAAATCAAACATTATACAAATGCAACCTAAGGAGGAGAGTTCCTTTGAG  216

Query  186  GCCAGGGAGCTACATTATCTTATCTGTATTGCCAGCGCAGAGGCCTACTAGTACATTGTA  245
            ||||||| ||||||||||||||||||||||||||||||||||||||||||||||||||||
Sbjct  217  GCCAGGG-GCTACATTATCTTATCTGTATTGCCAGCGCAGAGGCCTACTAGTACATTGTA  275

Query  246  GGGTCTAAGTACATTTTTCCTGAATGAAAGGTATTAAATGGTAACTTACGTCTTTATGCA  305
            ||||||||||||||||||||||||||||||||||||||||||||||||||||||||||||
Sbjct  276  GGGTCTAAGTACATTTTTCCTGAATGAAAGGTATTAAATGGTAACTTACGTCTTTATGCA  335

Query  306  CTCTATAAACTATGACGTGATCGTCTCCGTCTAACAACTACACTCAAATGCTTACCAAGC  365
            ||||||||||||||||||||||||||||||||||||||||||||||||||||||||||||
Sbjct  336  CTCTATAAACTATGACGTGATCGTCTCCGTCTAACAACTACACTCAAATGCTTACCAAGC  395

Query  366  TCTTTAAAGGGAAGAATTCCATGGTCGTATGAGCATTCAACAGT  409
            ||||||||||||||||||||||||||||||||||||||||||||
Sbjct  396  TCTTTAAAGGGAAGAATTCCATGGTCGTATGAGCATTCAACAGT  439
```

Sample 7

SNP 10

Sequence ID: **Query_140837** Length: **293** Number of Matches: **1**

Range 1: 33 to 293 Graphics ▼ Next Match ▲

Score	Expect	Identities	Gaps	Strand
472 bits(255)	6e-138	259/261(99%)	0/261(0%)	Plus/Plus

```
Query  2    TCAAATACCGTCTTTCTTCTGAGTGTTACCCAGTCAAGTATAAGTAGCCAAATTATTTTT  61
            ||| ||||| ||||||||||||||||||||||||||||||||||||||||||||||||||
Sbjct  33   TCATATACCATCTTTCTTCTGAGTGTTACCCAGTCAAGTATAAGTAGCCAAATTATTTTT  92

Query  62   GCACATCTTTCTGTTTCTCATGTCTTCATTTATTCAACAAGCACTTACTGGGAAGGTCTA  121
            ||||||||||||||||||||||||||||||||||||||||||||||||||||||||||||
Sbjct  93   GCACATCTTTCTGTTTCTCATGTCTTCATTTATTCAACAAGCACTTACTGGGAAGGTCTA  152

Query  122  CACCTGCATAGGCAATGCTGGAAAAAGGGTTAAGTAAACCAGGACATGACAATGGTGGCA  181
            ||||||||||||||||||||||||||||||||||||||||||||||||||||||||||||
Sbjct  153  CACCTGCATAGGCAATGCTGGAAAAAGGGTTAAGTAAACCAGGACATGACAATGGTGGCA  212

Query  182  AATGACTATCAGGTCTTCCCATGTGTTTGACTCAAACTTATTACCCTATGGTCCTTCTGA  241
            ||||||||||||||||||||||||||||||||||||||||||||||||||||||||||||
Sbjct  213  AATGACTATCAGGTCTTCCCATGTGTTTGACTCAAACTTATTACCCTATGGTCCTTCTGA  272

Query  242  CAATGGCAGAAGGTCTGAATC  262
            |||||||||||||||||||||
Sbjct  273  CAATGGCAGAAGGTCTGAATC  293
```

SNP 27

Sequence ID: **Query_50263** Length: **505** Number of Matches: **1**

Range 1: 42 to 505 Graphics ▼ Next Match ▲

Score	Expect	Identities	Gaps	Strand
806 bits(436)	0.0	456/464(98%)	8/464(1%)	Plus/Plus

```
Query  7    AAAGC-AGACTTTGTCTC-aaaaaaaCATGTATAGCTACATAATTAATAATATGCAAATA  64
            ||||| |||||||||||| |||||||||||||||||||||||||||||||||||||||||
Sbjct  42   AAAGCAAGACTTTGTCTCAAAAAAAACATGTATAGCTACATAATTAATAATATGCAAATA  101

Query  65   TTCTTCTTTAGGGTCACTTAGGTTTTCAACATACAGACCCAAGAGAAGGTACAGAGACTC  124
            ||||||||||||||||||||||||||||||||||||||||||||||||||||||||||||
Sbjct  102  TTCTTCTTTAGGGTCACTTAGGTTTTCAACATACAGACCCAAGAGAAGGTACAGAGACTC  161

Query  125  ATTCTCTCTCTCTTTCAAT------cacacacacacacacacacacacacacacacacac  178
            |||||||||||||||||||      |||||||||||||||||||||||||||||||||||
Sbjct  162  ATTCTCTCTCTCTTTCAATCACACACACACACACACACACACACACACACACACACACAC  221

Query  179  acCCCTCTTCACTATAATTATAATTACTATGTTGGCTTCCAGATCAGGGGTTAGAGCCTT  238
            ||||||||||||||||||||||||||||||||||||||||||||||||||||||||||||
Sbjct  222  ACCCCTCTTCACTATAATTATAATTACTATGTTGGCTTCCAGATCAGGGGTTAGAGCCTT  281

Query  239  GGCATGGAGACGCCTGAAAGGCACCCAAGGCAATTAGTGGTGTCCCTTCTCCACCCCCTA  298
            ||||||||||||||||||||||||||||||||||||||||||||||||||||||||||||
Sbjct  282  GGCATGGAGACGCCTGAAAGGCACCCAAGGCAATTAGTGGTGTCCCTTCTCCACCCCCTA  341

Query  299  CATACCTTCAGGCCCTTTGCACTTGCTGCTCCCCCTCCCAGTGCACACACCCCCAGATCC  358
            ||||||||||||||||||||||||||||||||||||||||||||||||||||||||||||
Sbjct  342  CATACCTTCAGGCCCTTTGCACTTGCTGCTCCCCCTCCCAGTGCACACACCCCCAGATCC  401

Query  359  TTGTGTGGCTCCCTCTCCCACCACATTTTGGTCTTAGCTCAAATGCCACCTCCTCAGAAA  418
            ||||||||||||||||||||||||||||||||||||||||||||||||||||||||||||
Sbjct  402  TTGTGTGGCTCCCTCTCCCACCACATTTTGGTCTTAGCTCAAATGCCACCTCCTCAGAAA  461

Query  419  TGCCTGGCCTGGCCGCCTTCAGGGCTGTCTATATGCTCACCAGC  462
            ||||||||||||||||||||||||||||||||||||||||||||
Sbjct  462  TGCCTGGCCTGGCCGCCTTCAGGGCTGTCTATATGCTCACCAGC  505
```

SNP 29

Sequence ID: **Query_48835** Length: **384** Number of Matches: **1**

Range 1: 36 to 384 Graphics ▼ Next Match ▲

Score	Expect	Identities	Gaps	Strand
636 bits(344)	0.0	349/351(99%)	2/351(0%)	Plus/Plus

```
Query  3    AAACCTGCCCATTCTGCACACGTATCCCAGAACTTAAAGTATAATTTTAAAAAAGATGTT  62
            ||||||||||||||||||||||||||||||||||||||||||||||||||||||||||||
Sbjct  36   AAACCTGCCCATTCTGCACACGTATCCCAGAACTTAAAGTATAATTTTAAAAAAGATGTT  95

Query  63   TTATAATCTAATTGGTGACTGACAACTAAGGTACGTTAGACCATTAGCAAAAGTAAATTA  122
            ||||||||||||||||||||||||||||||||||||||||||||||||||||||||||||
Sbjct  96   TTATAATCTAATTGGTGACTGACAACTAAGGTACGTTAGACCATTAGCAAAAGTAAATTA  155

Query  123  AGTGATAAGTATTATGGTAGGACCTCAGGTAGATATAGGAATTGGCAGAGAGAGACGT  182
            ||||||||||||||||||||||||||||||| ||||||||||||||||||||||||||||
Sbjct  156  AGTGATAAGTATTATGGTAGGACCTCAGGTA-ATATAGGAATTGGCAGAGAGAGACGT  214

Query  183  TAACATGGTTAACTCCAAAAGTGGTATGACCTAGAAAATGGGTAGATAGGTTTGAGTAAG  242
            ||||||||||||||||||||||||||||||||||||||||||||||||||||||||||||
Sbjct  215  TAACATGGTTAACTCCAAAAGTGGTATGACCTAGAAAATGGGTAGATAGGTTTGAGTAAG  274

Query  243  TGGAAGGAGAGAAAAGAAATTTCTAGGCATAGACGCAGTGTTTCTCAAGAAACTGAAAGA  302
            ||||||||||||||||||||||||||||||||||||||||||||||||||||||||||||
Sbjct  275  TGGAAGGAGAGAAAAGAAATTTCTAGGCATAGACGCAGTGTTTCTCAAGAAACTGAAAGA  334

Query  303  CAAATATGCAGGTCCTGCTTATGGAAAAGGGAAGACAGAAGGGTGTCTTGC  353
            |||||||||||||||||||||||||||||||||||||||||| ||||||||
Sbjct  335  CAAATATGCAGGTCCTGCTTATGGAAAAGGGAAGACAGAAGG-TGTCTTGC  384
```

SNP 46

```
Sequence ID: Query_58837  Length: 551  Number of Matches: 1

Range 1: 47 to 551  Graphics                                        ▼ Next Match

Score           Expect   Identities       Gaps          Strand
893 bits(483)   0.0      499/506(99%)     4/506(0%)     Plus/Plus

Query  14    CAAGAGATCCAGAT-ATATACCACGTTTAATTCAATTTATCACGTCCAAGAAGTTACAAA  72
             ||||| ||||| || | |||||||||||||||||||||||||||||||||||||||||||
Sbjct  47    CAAGAAATCCAAATAAAATACCACGTTTAATTCAATTTATCACGTCCAAGAAGTTACAAA  106

Query  73    GGCAATGTAGAAGAAGTAAGTAAAAATGGGGAAGCCCTAATTTTTAAGTCTGCCTTGAAA  132
             ||||||||||||||||||||||||||||||||||||||||||||||||||||||||||||
Sbjct  107   GGCAATGTAGAAGAAGTAAGTAAAAATGGGGAAGCCCTAATTTTTAAGTCTGCCTTGAAA  166

Query  133   ACAATTTTTTCTTATCAATGAGACTTTCTAATAGGACTCATCTTGCCATTGGCACCCACA  192
             ||||||||||||||||||||||||||||||||||||||||||||||||||||||||||||
Sbjct  167   ACAATTTTTTCTTATCAATGAGACTTTCTAATAGGACTCATCTTGCCATTGGCACCCACA  226

Query  193   AAATCTAACCACCCACCTCCACTTCTCCGGGAGGATCCCTTCCTAATGTCTGATGCCTCT  252
             ||||||||||||||||||||||||||||||||||||||||||||||||||||||||||||
Sbjct  227   AAATCTAACCACCCACCTCCACTTCTCCGGGAGGATCCCTTCCTAATGTCTGATGCCTCT  286

Query  253   TCCCTCACCTCCATTCTTTGCCCACTGCTGCACATGCTTAAAGCCCAGCCACCCTTTCAC  312
             ||||||||||||||||||||||||||||||||||||||||||||||||||||||||||||
Sbjct  287   TCCCTCACCTCCATTCTTTGCCCACTGCTGCACATGCTTAAAGCCCAGCCACCCTTTCAC  346

Query  313   CCACAGAGGTCATAGACCACTGCTACTATGGTTAATTCTCAGAAGTAATAAACTCAGAAC  372
             ||||||||||||||||||||||||||||||||||||||||||||||||||||||||||||
Sbjct  347   CCACAGAGGTCATAGACCACTGCTACTATGGTTAATTCTCAGAAGTAATAAACTCAGAAC  406

Query  373   CTGAAAGCACATAATGCCTACAAGACTGTTCACTATTTCAAACTTCTGCTCTGGTTTACA  432
             ||||||||||||||||||||||||||||||||||||||||||||||||||||||||||||
Sbjct  407   CTGAAAGCACATAATGCCTACAAGACTGTTCACTATTTCAAACTTCTGCTCTGGTTTACA  466

Query  433   TGAATT--aaaaaaaaaaaaTCACTCTATTTTTTCTGCTGTACCAAGGCaaaaaaaTTCT  490
             ||||||  ||||||||||||||||||||||||||||||||||||||||||||||||||||
Sbjct  467   TGAATTAAAAAAAAAAAAAATCACTCTATTTTTTCTGCTGTACCAAGGCAAAAAAATTCT  526

Query  491   ACAATCACATTTAAAGGGCATGGACT  516
             ||||||||||||||||| ||||||||
Sbjct  527   ACAATCACATTTAAAGG-CATGGACT  551
```

SNP 49

```
Sequence ID: Query_57613  Length: 439  Number of Matches: 1

Range 1: 41 to 439  Graphics                                        ▼ Next Match

Score           Expect   Identities       Gaps          Strand
699 bits(378)   0.0      394/401(98%)     4/401(0%)     Plus/Plus

Query  2     AAA-GCAGAGTCTGCCTTTGGTTG-AGCGAAGATGGGACGCTTGAATGCCCTTTCGTACT  59
             ||| |||| | ||| ||||||||| |||||||||||||||||||||||||||||||||||
Sbjct  41    AAACGCAG-GGCTG-CTTTGGTTGAAGCGAAGATGGGACGCTTGAATGCCCTTTCGTACT  98

Query  60    ACTCCCCTTTTACCTCGTTAATACCCACTGACCTATCCTCGTGGAATGCAGGGCTCAAAG  119
             ||||||||||||||||||||||||||||||||||||||||||||||||||||||||||||
Sbjct  99    ACTCCCCTTTTACCTCGTTAATACCCACTGACCTATCCTCGTGGAATGCAGGGCTCAAAG  158

Query  120   AACAATCTAAAAATCAAACATTATACAAATGCAACCTAAGGAGGAGAGTTCCTTTGAGGC  179
             ||||||||||||||||||||||||||||||||||||||||||||||||||||||||||||
Sbjct  159   AACAATCTAAAAATCAAACATTATACAAATGCAACCTAAGGAGGAGAGTTCCTTTGAGGC  218

Query  180   CAGGGACTACATTATCTTATCTGTATTGCCAGCGCAGAGGCCTACTAGTACATTGTAGGG  239
             ||||| ||||||||||||||||||||||||||||||||||||||||||||||||||||||
Sbjct  219   CAGGGGCTACATTATCTTATCTGTATTGCCAGCGCAGAGGCCTACTAGTACATTGTAGGG  278

Query  240   TCTAAGTACATTTTTCCTGAATGAAAGGTATTAAATGGTAACTTACGTCTTTATGCACTC  299
             ||||||||||||||||||||||||||||||||||||||||||||||||||||||||||||
Sbjct  279   TCTAAGTACATTTTTCCTGAATGAAAGGTATTAAATGGTAACTTACGTCTTTATGCACTC  338

Query  300   TATAAACTATGACGTGATCGTCTCCGTCTAACAACTACACTCAAATGCTTACCAAGCTCT  359
             ||||||||||||||||||||||||||||||||||||||||||||||||||||||||||||
Sbjct  339   TATAAACTATGACGTGATCGTCTCCGTCTAACAACTACACTCAAATGCTTACCAAGCTCT  398

Query  360   TTAAAGGGAAGAATTCCATGGTCGTATGAGCTTTCAACAGT  400
             ||||||||||||||||||||||||||||||| |||||||||
Sbjct  399   TTAAAGGGAAGAATTCCATGGTCGTATGAGCATTCAACAGT  439
```

Sample 8

SNP 10

Sequence ID: **Query_48527** Length: **293** Number of Matches: **1**

Range 1: 42 to 293 Graphics ▼ Next Match

Score	Expect	Identities	Gaps	Strand
460 bits(249)	1e-134	252/253(99%)	1/253(0%)	Plus/Plus

```
Query  4     ATCTTTTCTTCTGAGTGTTACCCAGTCAAGTATAAGTAGCCAAATTATTTTTGCACATCT  63
             ||| ||||||||||||||||||||||||||||||||||||||||||||||||||||||||
Sbjct  42    ATC-TTTCTTCTGAGTGTTACCCAGTCAAGTATAAGTAGCCAAATTATTTTTGCACATCT  100

Query  64    TTCTGTTTCTCATGTCTTCATTTATTCAACAAGCACTTACTGGGAAGGTCTACACCTGCA  123
             ||||||||||||||||||||||||||||||||||||||||||||||||||||||||||||
Sbjct  101   TTCTGTTTCTCATGTCTTCATTTATTCAACAAGCACTTACTGGGAAGGTCTACACCTGCA  160

Query  124   TAGGCAATGCTGGAAAAAGGGTTAAGTAAACCAGGACATGACAATGGTGGCAAATGACTA  183
             ||||||||||||||||||||||||||||||||||||||||||||||||||||||||||||
Sbjct  161   TAGGCAATGCTGGAAAAAGGGTTAAGTAAACCAGGACATGACAATGGTGGCAAATGACTA  220

Query  184   TCAGGTCTTCCCATGTGTTTGACTCAAACTTATTACCCTATGGTCCTTCTGACAATGGCA  243
             ||||||||||||||||||||||||||||||||||||||||||||||||||||||||||||
Sbjct  221   TCAGGTCTTCCCATGTGTTTGACTCAAACTTATTACCCTATGGTCCTTCTGACAATGGCA  280

Query  244   GAAGGTCTGAATC  256
             |||||||||||||
Sbjct  281   GAAGGTCTGAATC  293
```

SNP 27

Sequence ID: **Query_497805** Length: **505** Number of Matches: **1**

Range 1: 35 to 505 Graphics ▼ Next Match

Score	Expect	Identities	Gaps	Strand
804 bits(435)	0.0	460/471(98%)	6/471(1%)	Plus/Plus

```
Query  7     GGGAGAAAAGGAAAGAATTTGTCTC-aaaaaaaCATGTATAGCTACATAATTAATAATAT  65
             ||| || || | |||| |||||||| ||||||||||||||||||||||||||||||||||
Sbjct  35    GGGTGACAAAGCAAGACTTTGTCTCAAAAAAAACATGTATAGCTACATAATTAATAATAT  94

Query  66    GCAAATATTCTTCTTTAGGGTCACTTAGGTTTTCAACATACAGACCCAAGAGAAGGTACA  125
             ||||||||||||||||||||||||||||||||||||||||||||||||||||||||||||
Sbjct  95    GCAAATATTCTTCTTTAGGGTCACTTAGGTTTTCAACATACAGACCCAAGAGAAGGTACA  154

Query  126   GAGACTCATTCTCTCTCTCTTTCAATcacacacacacacacacacacacacacacacaca  185
             ||||||||||||||||||||||||||||||||||||||||||||||||||||||||||||
Sbjct  155   GAGACTCATTCTCTCTCTCTTTCAATCACACACACACACACACACACACACACACACACA  214

Query  186   cac-----CCCCTCTTCACTATAATTATAATTACTATGTTGGCTTCCAGATCAGGGGTTA  240
             |||     ||||||||||||||||||||||||||||||||||||||||||||||||||||
Sbjct  215   CACACACACCCCTCTTCACTATAATTATAATTACTATGTTGGCTTCCAGATCAGGGGTTA  274

Query  241   GAGCCTTGGCATGGAGACGCCTGAAAGGCACCCAAGGCAATTAGTGGTGTCCCTTCTCCA  300
             ||||||||||||||||||||||||||||||||||||||||||||||||||||||||||||
Sbjct  275   GAGCCTTGGCATGGAGACGCCTGAAAGGCACCCAAGGCAATTAGTGGTGTCCCTTCTCCA  334

Query  301   CCCCCTACATACCTTCAGGCCCTTTGCACTTGCTGCTCCCCCTCCCAGTGCACACACCCC  360
             ||||||||||||||||||||||||||||||||||||||||||||||||||||||||||||
Sbjct  335   CCCCCTACATACCTTCAGGCCCTTTGCACTTGCTGCTCCCCCTCCCAGTGCACACACCCC  394

Query  361   CAGATCCTTGTGTGGCTCCCTCTCCCACCACATTTTGGTCTTAGCTCAAATGCCACCTCC  420
             ||||||||||||||||||||||||||||||||||||||||||||||||||||||||||||
Sbjct  395   CAGATCCTTGTGTGGCTCCCTCTCCCACCACATTTTGGTCTTAGCTCAAATGCCACCTCC  454

Query  421   TCAGAAATGCCTGGCCTGGCCGCCTTCAGGGCTGTCTATATGCTCACCAGC  471
             |||||||||||||||||||||||||||||||||||||||||||||||||||
Sbjct  455   TCAGAAATGCCTGGCCTGGCCGCCTTCAGGGCTGTCTATATGCTCACCAGC  505
```

SNP 29

Sequence ID: **Query_65153** Length: **384** Number of Matches: **1**

Range 1: 38 to 384 Graphics ▼ Next Match ▲ P

Score	Expect	Identities	Gaps	Strand
634 bits(343)	0.0	346/347(99%)	1/347(0%)	Plus/Plus

```
Query  5     ACCTG-CCATTCTGCACACGTATCCCAGAACTTAAAGTATAATTTTAAAAAAGATGTTTT  63
             ||||| ||||||||||||||||||||||||||||||||||||||||||||||||||||||
Sbjct  38    ACCTGCCCATTCTGCACACGTATCCCAGAACTTAAAGTATAATTTTAAAAAAGATGTTTT  97

Query  64    ATAATCTAATTGGTGACTGACAACTAAGGTACGTTAGACCATTAGCAAAAGTAAATTAAG  123
             ||||||||||||||||||||||||||||||||||||||||||||||||||||||||||||
Sbjct  98    ATAATCTAATTGGTGACTGACAACTAAGGTACGTTAGACCATTAGCAAAAGTAAATTAAG  157

Query  124   TGATAAGTATTATGGTAGGGACCTCAGGTAATATAGGAATTGGGCAGAGAGAGACGTTAA  183
             ||||||||||||||||||||||||||||||||||||||||||||||||||||||||||||
Sbjct  158   TGATAAGTATTATGGTAGGGACCTCAGGTAATATAGGAATTGGGCAGAGAGAGACGTTAA  217

Query  184   CATGGTTAACTCCAAAAGTGGTATGACCTAGAAAATGGGTAGATAGGTTTGAGTAAGTGG  243
             ||||||||||||||||||||||||||||||||||||||||||||||||||||||||||||
Sbjct  218   CATGGTTAACTCCAAAAGTGGTATGACCTAGAAAATGGGTAGATAGGTTTGAGTAAGTGG  277

Query  244   AAGGAGAGAAAAGAAATTTCTAGGCATAGACGCAGTGTTTCTCAAGAAACTGAAAGACAA  303
             ||||||||||||||||||||||||||||||||||||||||||||||||||||||||||||
Sbjct  278   AAGGAGAGAAAAGAAATTTCTAGGCATAGACGCAGTGTTTCTCAAGAAACTGAAAGACAA  337

Query  304   ATATGCAGGTCCTGCTTATGGAAAAGGGAAGACAGAAGGTGTCTTGC  350
             |||||||||||||||||||||||||||||||||||||||||||||||
Sbjct  338   ATATGCAGGTCCTGCTTATGGAAAAGGGAAGACAGAAGGTGTCTTGC  384
```

SNP 46

Sequence ID: **Query_44997** Length: **551** Number of Matches: **1**

Range 1: 40 to 551 Graphics ▼ Next Match ▲

Score	Expect	Identities	Gaps	Strand
894 bits(484)	0.0	504/513(98%)	3/513(0%)	Plus/Plus

```
Query  9    TATAAATCAAG-AATCCAAAATAAAATACCACGTTTAATTCAATTTATCACGTCCAAGAA  67
            || |||||||| ||||| ||||||||||||||||||||||||||||||||||||||||||
Sbjct  40   TAAAAATCAAGAAATCC-AAATAAAATACCACGTTTAATTCAATTTATCACGTCCAAGAA  98

Query  68   GTTACAAAGGCAATGTAGAAGAAGTAAGTAAAAATGGGGAAGCCCTAATTTTTAAGTCTG  127
            ||||||||||||||||||||||||||||||||||||||||||||||||||||||||||||
Sbjct  99   GTTACAAAGGCAATGTAGAAGAAGTAAGTAAAAATGGGGAAGCCCTAATTTTTAAGTCTG  158

Query  128  CCTTGAAAACAATTTTTTCTTATCAATGAGACTTTCTAATAGGACTCATCTTGCCATTGG  187
            ||||||||||||||||||||||||||||||||||||||||||||||||||||||||||||
Sbjct  159  CCTTGAAAACAATTTTTTCTTATCAATGAGACTTTCTAATAGGACTCATCTTGCCATTGG  218

Query  188  CACCCACAAAATCTAACCACCCACCTCCACTTCTCCGGAAGGATCCCTTCCTAATGTCTG  247
            |||||||||||||||||||||||||||||||||||||| |||||||||||||||||||||
Sbjct  219  CACCCACAAAATCTAACCACCCACCTCCACTTCTCCGGGAGGATCCCTTCCTAATGTCTG  278

Query  248  ATGCCTCTTCCCTCACCTCCATTCTTTGCCCACTGCTGCACATGCTTAAAGCCCAGCCAC  307
            ||||||||||||||||||||||||||||||||||||||||||||||||||||||||||||
Sbjct  279  ATGCCTCTTCCCTCACCTCCATTCTTTGCCCACTGCTGCACATGCTTAAAGCCCAGCCAC  338

Query  308  CCTTTCACCCACAGAGGTCATAGACCACTGCTACTATGGTTAATTCTCAGAAGTAATAAA  367
            ||||||||||||||||||||||||||||||||||||||||||||||||||||||||||||
Sbjct  339  CCTTTCACCCACAGAGGTCATAGACCACTGCTACTATGGTTAATTCTCAGAAGTAATAAA  398

Query  368  CTCAGAACCTGAAAGCACATAATGCCTACAAGACTGTTCACTATTTCAAACTTCTGCTCT  427
            ||||||||||||||||||||||||||||||||||||||||||||||||||||||||||||
Sbjct  399  CTCAGAACCTGAAAGCACATAATGCCTACAAGACTGTTCACTATTTCAAACTTCTGCTCT  458

Query  428  GGTTTACATGAATT-aaaaaaaaaaaaaTCACTCttttttttCTGCTGTACCAAGGCaaa  486
            |||||||||||||| |||||||||||||||||||| ||||||||||||||||||||||||
Sbjct  459  GGTTTACATGAATTAAAAAAAAAAAAAAATCACTCTATTTTTTCTGCTGTACCAAGGCAAA  518

Query  487  aaaaTTCTACAATCCCTTTTAAAGGCTTGGACT  519
            |||||||||||||| | ||||||||| ||||||
Sbjct  519  AAAATTCTACAATCACATTTAAAGGCATGGACT  551
```

SNP 49

Sequence ID: **Query_52013** Length: **439** Number of Matches: **1**

Range 1: 59 to 439 Graphics ▼ Next Match ▲

Score	Expect	Identities	Gaps	Strand
693 bits(375)	0.0	380/382(99%)	2/382(0%)	Plus/Plus

```
Query  34   GTTG-AGCGAAGATGGGACGCTTGAATGCCCTTTCGTACTACTCCCCTTTTACCTCGTTA  92
            |||| |||||||||||||||||||||||||||||||||||||||||||||||||||||||
Sbjct  59   GTTGAAGCGAAGATGGGACGCTTGAATGCCCTTTCGTACTACTCCCCTTTTACCTCGTTA  118

Query  93   ATACCCACTGACCTATCCTCGTGGAATGCAGGGCTCAAAGAACAATCTAAAAATCAAACA  152
            ||||||||||||||||||||||||||||||||||||||||||||||||||||||||||||
Sbjct  119  ATACCCACTGACCTATCCTCGTGGAATGCAGGGCTCAAAGAACAATCTAAAAATCAAACA  178

Query  153  TTATACAAATGCAACCTAAGGAGGAGAGTTCCTTTGAGGCCAGGGAGCTACATTATCTTA  212
            ||||||||||||||||||||||||||||||||||||||||||||| ||||||||||||||
Sbjct  179  TTATACAAATGCAACCTAAGGAGGAGAGTTCCTTTGAGGCCAGGG-GCTACATTATCTTA  237

Query  213  TCTGTATTGCCAGCGCAGAGGCCTACTAGTACATTGTAGGGTCTAAGTACATTTTTCCTG  272
            ||||||||||||||||||||||||||||||||||||||||||||||||||||||||||||
Sbjct  238  TCTGTATTGCCAGCGCAGAGGCCTACTAGTACATTGTAGGGTCTAAGTACATTTTTCCTG  297

Query  273  AATGAAAGGTATTAAATGGTAACTTACGTCTTTATGCACTCTATAAACTATGACGTGATC  332
            ||||||||||||||||||||||||||||||||||||||||||||||||||||||||||||
Sbjct  298  AATGAAAGGTATTAAATGGTAACTTACGTCTTTATGCACTCTATAAACTATGACGTGATC  357

Query  333  GTCTCCGTCTAACAACTACACTCAAATGCTTACCAAGCTCTTTAAAGGGAAGAATTCCAT  392
            ||||||||||||||||||||||||||||||||||||||||||||||||||||||||||||
Sbjct  358  GTCTCCGTCTAACAACTACACTCAAATGCTTACCAAGCTCTTTAAAGGGAAGAATTCCAT  417

Query  393  GGTCGTATGAGCATTCAACAGT  414
            ||||||||||||||||||||||
Sbjct  418  GGTCGTATGAGCATTCAACAGT  439
```

Sample 9

SNP 10

Sequence ID: **Query_65021** Length: **293** Number of Matches: **1**

Range 1: 74 to 293 Graphics ▼ Next Match ▲ Previ

Score	Expect	Identities	Gaps	Strand
392 bits(212)	4e-114	219/222(99%)	2/222(0%)	Plus/Plus

```
Query  8    AAGTAAGCCAAAATTATTTTTGCACATCTTTCTGTTTCTCATGTCTTCATTTATTCAACA  67
            |||| |||| ||||||||||||||||||||||||||||||||||||||||||||||||||
Sbjct  74   AAGT-AGCC-AAATTATTTTTGCACATCTTTCTGTTTCTCATGTCTTCATTTATTCAACA  131

Query  68   AGCACTTACTGGGAAGGTCTACAACTGCATAGGCAATGCTGGAAAAAGGGTTAAGTAAAC  127
            ||||||||||||||||||||||| ||||||||||||||||||||||||||||||||||||
Sbjct  132  AGCACTTACTGGGAAGGTCTACACCTGCATAGGCAATGCTGGAAAAAGGGTTAAGTAAAC  191

Query  128  CAGGACATGACAATGGTGGCAAATGACTATCAGGTCTTCCCATGTGTTTGACTCAAACTT  187
            ||||||||||||||||||||||||||||||||||||||||||||||||||||||||||||
Sbjct  192  CAGGACATGACAATGGTGGCAAATGACTATCAGGTCTTCCCATGTGTTTGACTCAAACTT  251

Query  188  ATTACCCTATGGTCCTTCTGACAATGGCAGAAGGTCTGAATC  229
            ||||||||||||||||||||||||||||||||||||||||||
Sbjct  252  ATTACCCTATGGTCCTTCTGACAATGGCAGAAGGTCTGAATC  293
```

SNP 27

Sequence ID: **Query_221465** Length: **505** Number of Matches: **1**

Range 1: 35 to 505 Graphics ▼ Next Match ▲ Pr

Score	Expect	Identities	Gaps	Strand
787 bits(426)	0.0	458/471(97%)	12/471(2%)	Plus/Plus

```
Query  5    GGGGGA-AAA-CAAGACTTTGTCTC--AAAAAACATGTATAGCTACATAATTAATAATAT  60
            ||| || ||| ||||||||||||||  |||||||||||||||||||||||||||||||||
Sbjct  35   GGGTGACAAAGCAAGACTTTGTCTCAAAAAAAACATGTATAGCTACATAATTAATAATAT  94

Query  61   GCAAATATTCTTCTTTAGGGTCACTTAGGTTTTCAACATACAGACCCAAGAGAAGGTACA  120
            ||||||||||||||||||||||||||||||||||||||||||||||||||||||||||||
Sbjct  95   GCAAATATTCTTCTTTAGGGTCACTTAGGTTTTCAACATACAGACCCAAGAGAAGGTACA  154

Query  121  GAGACTCATTCTCTCTCTCTTTCAAT--------cacacacacacacacacacacacaca  172
            ||||||||||||||||||||||||||        ||||||||||||||||||||||||||
Sbjct  155  GAGACTCATTCTCTCTCTCTTTCAATCACACACACACACACACACACACACACACACACA  214

Query  173  cacacacacCCCTCTTCACTATAATTATAATTACTATGTTGGCTTCCAGATCAGGGGTTA  232
            ||||||||||||||||||||||||||||||||||||||||||||||||||||||||||||
Sbjct  215  CACACACACCCCTCTTCACTATAATTATAATTACTATGTTGGCTTCCAGATCAGGGGTTA  274

Query  233  GAGCCTTGGCATGGAGACGCCTGAAAGGCACCCAAGGCAATTAGTGGTGTCCCTTCTCCA  292
            ||||||||||||||||||||||||||||||||||||||||||||||||||||||||||||
Sbjct  275  GAGCCTTGGCATGGAGACGCCTGAAAGGCACCCAAGGCAATTAGTGGTGTCCCTTCTCCA  334

Query  293  CCCCCTACATACCTTCAGGCCCTTTGCACTTGCTGCTCCCCCTCCCAGTGCACACACCCC  352
            ||||||||||||||||||||||||||||||||||||||||||||||||||||||||||||
Sbjct  335  CCCCCTACATACCTTCAGGCCCTTTGCACTTGCTGCTCCCCCTCCCAGTGCACACACCCC  394

Query  353  CAGATCCTTGTGTGGCTCCCTCTCCCACCACATTTTGGTCTTAGCTCAAATGCCACCTCC  412
            ||||||||||||||||||||||||||||||||||||||||||||||||||||||||||||
Sbjct  395  CAGATCCTTGTGTGGCTCCCTCTCCCACCACATTTTGGTCTTAGCTCAAATGCCACCTCC  454

Query  413  TCAGAAATGCCTGGCCTGGCCGCCTTCAGGGCTGTCTATATGCTCACCAGC  463
            |||||||||||||||||||||||||||||||||||||||||||||||||||
Sbjct  455  TCAGAAATGCCTGGCCTGGCCGCCTTCAGGGCTGTCTATATGCTCACCAGC  505
```

SNP 29

Sequence ID: **Query_49495** Length: **384** Number of Matches: **1**

Range 1: 37 to 384 Graphics ▼ Next Match ▲

Score	Expect	Identities	Gaps	Strand
630 bits(341)	0.0	346/348(99%)	1/348(0%)	Plus/Plus

```
Query  4    AACCTG-CCATTCTGCACACGTATCCCAGAACTTAAAGTATAATTTTAAAAAAGATGTTT  62
            |||||| |||||||||||||||||||||||||||||||||||||||||||||||||||||
Sbjct  37   AACCTGCCCATTCTGCACACGTATCCCAGAACTTAAAGTATAATTTTAAAAAAGATGTTT  96

Query  63   TATAATCTAATTGGTGACTGACAACTAAGGTACGTTAGACCATTAGCAAAAGTAAATTAA  122
            ||||||||||||||||||||||||||||||||||||||||||||||||||||||||||||
Sbjct  97   TATAATCTAATTGGTGACTGACAACTAAGGTACGTTAGACCATTAGCAAAAGTAAATTAA  156

Query  123  GTGATAAGTATTATGGTAGGACCTCAGGTGATATAGGAATTGGGCAGAGAGAGACGTTA  182
            ||||||||||||||||||||||||||||| ||||||||||||||||||||||||||||||
Sbjct  157  GTGATAAGTATTATGGTAGGACCTCAGGTAATATAGGAATTGGGCAGAGAGAGACGTTA  216

Query  183  ACATGGTTAACTCCAAAAGTGGTATGACCTAGAAAATGGGTAGATAGGTTTGAGTAAGTG  242
            ||||||||||||||||||||||||||||||||||||||||||||||||||||||||||||
Sbjct  217  ACATGGTTAACTCCAAAAGTGGTATGACCTAGAAAATGGGTAGATAGGTTTGAGTAAGTG  276

Query  243  GAAGGAGAGAAAAGAAATTTCTAGGCATAGACGCAGTGTTTCTCAAGAAACTGAAAGACA  302
            ||||||||||||||||||||||||||||||||||||||||||||||||||||||||||||
Sbjct  277  GAAGGAGAGAAAAGAAATTTCTAGGCATAGACGCAGTGTTTCTCAAGAAACTGAAAGACA  336

Query  303  AATATGCAGGTCCTGCTTATGGAAAAGGGAAGACAGAAGGTGTCTTGC  350
            ||||||||||||||||||||||||||||||||||||||||||||||||
Sbjct  337  AATATGCAGGTCCTGCTTATGGAAAAGGGAAGACAGAAGGTGTCTTGC  384
```

SNP 46

Sequence ID: **Query_41361** Length: **551** Number of Matches: **1**

Range 1: 36 to 503 Graphics ▼ Next Match ▲

Score	Expect	Identities	Gaps	Strand
824 bits(446)	0.0	462/469(99%)	3/469(0%)	Plus/Plus

```
Query  9    ACA-TAAAAATCAAGGAAATCCAAATAAAATACCACGTTTAATTCAATTTATCACGTCCA  67
            ||| |||||||||| |||||||||||||||||||||||||||||||||||||||||||||
Sbjct  36   ACATTAAAAATCAA-GAAATCCAAATAAAATACCACGTTTAATTCAATTTATCACGTCCA  94

Query  68   AGAAGTTACAAAGGCAATGTAGAAGAAGTAAGTAAAAATGGGGAAGCCCTAATTTTTAAG  127
            ||||||||||||||||||||||||||||||||||||||||||||||||||||||||||||
Sbjct  95   AGAAGTTACAAAGGCAATGTAGAAGAAGTAAGTAAAAATGGGGAAGCCCTAATTTTTAAG  154

Query  128  TCTGCCTTGAAAACAATTTTTTCTTATCAATGAGACTTTCTAATAGGACTCATCTTGCCA  187
            ||||||||||||||||||||||||||||||||||||||||||||||||||||||||||||
Sbjct  155  TCTGCCTTGAAAACAATTTTTTCTTATCAATGAGACTTTCTAATAGGACTCATCTTGCCA  214

Query  188  TTGGCACCCACAAAATCTAACCACCCACCTCCACTTCTCCGGAAGGATCCCTTCCTAATG  247
            |||||||||||||||||||||||||||||||||||||||||| |||||||||||||||||
Sbjct  215  TTGGCACCCACAAAATCTAACCACCCACCTCCACTTCTCCGGGAGGATCCCTTCCTAATG  274

Query  248  TCTGATGCCTCTTCCCTCACCTCCATTCTTTGCCCACTGCTGCACATGCTTAAAGCCCAG  307
            ||||||||||||||||||||||||||||||||||||||||||||||||||||||||||||
Sbjct  275  TCTGATGCCTCTTCCCTCACCTCCATTCTTTGCCCACTGCTGCACATGCTTAAAGCCCAG  334

Query  308  CCACCCTTTCACCCACAGAGGTCATAGACCACTGCTACTATGGTTAATTCTCAGAAGTAA  367
            ||||||||||||||||||||||||||||||||||||||||||||||||||||||||||||
Sbjct  335  CCACCCTTTCACCCACAGAGGTCATAGACCACTGCTACTATGGTTAATTCTCAGAAGTAA  394

Query  368  TAAACTCAGAACCTGAAAGCACATAATGCCTACAAAACTGTTCACTATTTCAAACTTCTG  427
            ||||||||||||||||||||||||||||||||||| ||||||||||||||||||||||||
Sbjct  395  TAAACTCAGAACCTGAAAGCACATAATGCCTACAAGACTGTTCACTATTTCAAACTTCTG  454

Query  428  CTCTGGGTTACATGAATT-aaaaaaaaaaaaaTCACTCAATTTTTTCTG  475
            |||||| ||||||||||| ||||||||||||||||||| ||||||||||
Sbjct  455  CTCTGGTTTACATGAATTAAAAAAAAAAAAAATCACTCTATTTTTTCTG  503
```

SNP 49

Sequence ID: **Query_48125** Length: **439** Number of Matches: **1**

Range 1: 51 to 439 Graphics ▼ Next Match ▲

Score	Expect	Identities	Gaps	Strand
706 bits(382)	0.0	387/389(99%)	1/389(0%)	Plus/Plus

```
Query  17   CTGCTTTGGTTG-AGCGAAGATGGGACGCTTGAATGCCCTTTCGTACTACTCCCCTTTTA  75
            |||||||||||| |||||||||||||||||||||||||||||||||||||||||||||||
Sbjct  51   CTGCTTTGGTTGAAGCGAAGATGGGACGCTTGAATGCCCTTTCGTACTACTCCCCTTTTA  110

Query  76   CCTCGTTAATACCCACTGACCTATCCTCGTGGAATGCAGGGCTCAAAGAACAATCTAAAA  135
            ||||||||||||||||||||||||||||||||||||||||||||||||||||||||||||
Sbjct  111  CCTCGTTAATACCCACTGACCTATCCTCGTGGAATGCAGGGCTCAAAGAACAATCTAAAA  170

Query  136  ATCAAACATTATACAAATGCAACCTAAGGAGGAGAGTTCCTTTGAGGCCAGGGACTACAT  195
            ||||||||||||||||||||||||||||||||||||||||||||||||||||| ||||||
Sbjct  171  ATCAAACATTATACAAATGCAACCTAAGGAGGAGAGTTCCTTTGAGGCCAGGGGCTACAT  230

Query  196  TATCTTATCTGTATTGCCAGCGCAGAGGCCTACTAGTACATTGTAGGGTCTAAGTACATT  255
            ||||||||||||||||||||||||||||||||||||||||||||||||||||||||||||
Sbjct  231  TATCTTATCTGTATTGCCAGCGCAGAGGCCTACTAGTACATTGTAGGGTCTAAGTACATT  290

Query  256  TTTCCTGAATGAAAGGTATTAAATGGTAACTTACGTCTTTATGCACTCTATAAACTATGA  315
            ||||||||||||||||||||||||||||||||||||||||||||||||||||||||||||
Sbjct  291  TTTCCTGAATGAAAGGTATTAAATGGTAACTTACGTCTTTATGCACTCTATAAACTATGA  350

Query  316  CGTGATCGTCTCCGTCTAACAACTACACTCAAATGCTTACCAAGCTCTTTAAAGGGAAGA  375
            ||||||||||||||||||||||||||||||||||||||||||||||||||||||||||||
Sbjct  351  CGTGATCGTCTCCGTCTAACAACTACACTCAAATGCTTACCAAGCTCTTTAAAGGGAAGA  410

Query  376  ATTCCATGGTCGTATGAGCATTCAACAGT  404
            |||||||||||||||||||||||||||||
Sbjct  411  ATTCCATGGTCGTATGAGCATTCAACAGT  439
```

Sample 10

SNP 10

```
Sequence ID: Query_5401  Length: 293  Number of Matches: 1

Range 1: 47 to 293  Graphics                                   ▼ Next Match  ▲ P

Score            Expect    Identities      Gaps         Strand
451 bits(244)    8e-132    247/248(99%)    1/248(0%)    Plus/Plus

Query  18   TCTTCTGAGTGTTACCCAGTCAAGTATAAGTAGCCAAATTATTTTTGCACATCTTTCTGT  77
            ||||||||||||||||||||||||||||||||||||||||||||||||||||||||||||
Sbjct  47   TCTTCTGAGTGTTACCCAGTCAAGTATAAGTAGCCAAATTATTTTTGCACATCTTTCTGT  106

Query  78   TTCTCATGTCTTCATTTATTCAACAAGCACTTACTGGGAAGGTCTACAACCTGCATAGGC  137
            ||||||||||||||||||||||||||||||||||||||||||||||| ||||||||||||
Sbjct  107  TTCTCATGTCTTCATTTATTCAACAAGCACTTACTGGGAAGGTCTAC-ACCTGCATAGGC  165

Query  138  AATGCTGGAAAAAGGGTTAAGTAAACCAGGACATGACAATGGTGGCAAATGACTATCAGG  197
            ||||||||||||||||||||||||||||||||||||||||||||||||||||||||||||
Sbjct  166  AATGCTGGAAAAAGGGTTAAGTAAACCAGGACATGACAATGGTGGCAAATGACTATCAGG  225

Query  198  TCTTCCCATGTGTTTGACTCAAACTTATTACCCTATGGTCCTTCTGACAATGGCAGAAGG  257
            ||||||||||||||||||||||||||||||||||||||||||||||||||||||||||||
Sbjct  226  TCTTCCCATGTGTTTGACTCAAACTTATTACCCTATGGTCCTTCTGACAATGGCAGAAGG  285

Query  258  TCTGAATC  265
            ||||||||
Sbjct  286  TCTGAATC  293
```

SNP 27

```
Sequence ID: Query_529989  Length: 505  Number of Matches: 1

Range 1: 87 to 505  Graphics                                   ▼ Next Match  ▲ P

Score            Expect    Identities      Gaps          Strand
717 bits(388)    0.0       413/423(98%)    10/423(2%)    Plus/Plus

Query  12   AATAAATATGGCAAATATTCCTTCTTTAGGGGTCACTTAGGTTTTCAACATACAGACCCA  71
            ||| ||||| ||||||||| |||||||| |||||||||||||||||||||||||||||||
Sbjct  87   AAT-AATAT-GCAAATATT-CTTCTTTA-GGGTCACTTAGGTTTTCAACATACAGACCCA  142

Query  72   AGAGAAGGTACAGAGACTCATTCTCTCTCTCTTTCAAT------cacacacacacacaca  125
            ||||||||||||||||||||||||||||||||||||||      ||||||||||||||||
Sbjct  143  AGAGAAGGTACAGAGACTCATTCTCTCTCTCTTTCAATCACACACACACACACACACACA  202

Query  126  cacacacacacacacacacacCCCTCTTCACTATAATTATAATTACTATGTTGGCTTCCA  185
            ||||||||||||||||||||||||||||||||||||||||||||||||||||||||||||
Sbjct  203  CACACACACACACACACACACCCCTCTTCACTATAATTATAATTACTATGTTGGCTTCCA  262

Query  186  GATCAGGGGTTAGAGCCTTGGCATGGAGACGCCTGAAAGGCACCCAAGGCAATTAGTGGT  245
            ||||||||||||||||||||||||||||||||||||||||||||||||||||||||||||
Sbjct  263  GATCAGGGGTTAGAGCCTTGGCATGGAGACGCCTGAAAGGCACCCAAGGCAATTAGTGGT  322

Query  246  GTCCCTTCTCCACCCCCTACATACCTTCAGGCCCTTTGCACTTGCTGCTCCCCCTCCCAG  305
            ||||||||||||||||||||||||||||||||||||||||||||||||||||||||||||
Sbjct  323  GTCCCTTCTCCACCCCCTACATACCTTCAGGCCCTTTGCACTTGCTGCTCCCCCTCCCAG  382

Query  306  TGCACACACCCCCAGATCCTTGTGTGGCTCCCTCTCCCACCACATTTTGGTCTTAGCTCA  365
            ||||||||||||||||||||||||||||||||||||||||||||||||||||||||||||
Sbjct  383  TGCACACACCCCCAGATCCTTGTGTGGCTCCCTCTCCCACCACATTTTGGTCTTAGCTCA  442

Query  366  AATGCCACCTCCTCAGAAATGCCTGGCCTGGCCGCCTTCAGGGCTGTCTATATGCTCACC  425
            ||||||||||||||||||||||||||||||||||||||||||||||||||||||||||||
Sbjct  443  AATGCCACCTCCTCAGAAATGCCTGGCCTGGCCGCCTTCAGGGCTGTCTATATGCTCACC  502

Query  426  AGC  428
            |||
Sbjct  503  AGC  505
```

SNP 29

```
Sequence ID: Query_47901  Length: 384  Number of Matches: 1

Range 1: 40 to 384  Graphics                                   ▼ Next Match

Score            Expect    Identities      Gaps         Strand
621 bits(336)    0.0       344/347(99%)    3/347(0%)    Plus/Plus

Query  6    CTG-CCATTCTGCACACGTATCCCAGAACTTAAAGTATAATTTTAAAAAAGATGTTTTAT  64
            ||| ||||||||||||||||||||||||||||||||||||||||||||||||||||||||
Sbjct  40   CTGCCCATTCTGCACACGTATCCCAGAACTTAAAGTATAATTTTAAAAAAGATGTTTTAT  99

Query  65   AATCTAATTGGTGACTGACAACTAAGGTACGTTAGACCATTAGCAAAAGTAAATTAAGTG  124
            ||||||||||||||||||||||||||||||||||||||||||||||||||||||||||||
Sbjct  100  AATCTAATTGGTGACTGACAACTAAGGTACGTTAGACCATTAGCAAAAGTAAATTAAGTG  159

Query  125  ATAAGTATTATGGTAGGGACCTCAGGTAGATATAGGAATTGGGCAGAGAGAGACGTTAAC  184
            |||||||||||||||||||||||||||| |||||||||||||||||||||||||||||||
Sbjct  160  ATAAGTATTATGGTAGGGACCTCAGGTA-ATATAGGAATTGGGCAGAGAGAGACGTTAAC  218

Query  185  ATGGTTAACTCCAAAAGTGGTATGACCTAGAAAATGGGTAGATAGGTTTGAGTAAGTGGA  244
            ||||||||||||||||||||||||||||||||||||||||||||||||||||||||||||
Sbjct  219  ATGGTTAACTCCAAAAGTGGTATGACCTAGAAAATGGGTAGATAGGTTTGAGTAAGTGGA  278

Query  245  AGGAGAGAAAAGAAATTTCTAGGCATAGACGCAGTGTTTCTCAAGAAACTGAAAGACAAA  304
            ||||||||||||||||||||||||||||||||||||||||||||||||||||||||||||
Sbjct  279  AGGAGAGAAAAGAAATTTCTAGGCATAGACGCAGTGTTTCTCAAGAAACTGAAAGACAAA  338

Query  305  TATGCAGGTCCTGCTTATGGAAAAGGGAAGACAGAAGGGTGTCTTGC  351
            |||||||||||||||||||||||||||||||||||||| ||||||||
Sbjct  339  TATGCAGGTCCTGCTTATGGAAAAGGGAAGACAGAAGG-TGTCTTGC  384
```

SNP 46

Sequence ID: **Query_25169** Length: **551** Number of Matches: **1**

Range 1: 42 to 543 Graphics ▼ Next Match ▲

Score	Expect	Identities	Gaps	Strand
832 bits(450)	0.0	486/503(97%)	4/503(0%)	Plus/Plus

```
Query  12   AAAATC-AGGAATCCAAATAAAATACCACGTTTAATTCAATTTATCACGTCCAAGAAGTT  70
            |||||| || ||||||||||||||||||||||||||||||||||||||||||||||||||
Sbjct  42   AAAATCAAGAAATCCAAATAAAATACCACGTTTAATTCAATTTATCACGTCCAAGAAGTT  101

Query  71   ACAAAGGCAATGTAGAAGAAGTAAGTAAAAATGGGGAAGCCCTAATTTTTAAGTCTGCCT  130
            ||||||||||||||||||||||||||||||||||||||||||||||||||||||||||||
Sbjct  102  ACAAAGGCAATGTAGAAGAAGTAAGTAAAAATGGGGAAGCCCTAATTTTTAAGTCTGCCT  161

Query  131  TGAAAACAATTTTTTCTTATCAATGAGACTTTCTAATAGGACTCATCTTGCCATTGGCAC  190
            ||||||||||||||||||||||||||||||||||||||||||||||||||||||||||||
Sbjct  162  TGAAAACAATTTTTTCTTATCAATGAGACTTTCTAATAGGACTCATCTTGCCATTGGCAC  221

Query  191  CCACAAAATCTAACCACCCACCTCCACTTCTCCGGAGAGGATCCCTTCCTAATGTCTGAT  250
            ||||||||||||||||||||||||||||||||||| ||||||||||||||||||||||||
Sbjct  222  CCACAAAATCTAACCACCCACCTCCACTTCTCCGG-GAGGATCCCTTCCTAATGTCTGAT  280

Query  251  GCCTCTTCCCTCACCTCCATTCTTTGCCCACTGCTGCACATGCTTAAAGCCCAGCCACCC  310
            ||||||||||||||||||||||||||||||||||||||||||||||||||||||||||||
Sbjct  281  GCCTCTTCCCTCACCTCCATTCTTTGCCCACTGCTGCACATGCTTAAAGCCCAGCCACCC  340

Query  311  TTTCACCCACAGAGGTCATAGACCACTGCTACTATGGTTAATTCTCAGAAGTAATAAACT  370
            ||||||||||||||||||||||||||||||||||||||||||||||||||||||||||||
Sbjct  341  TTTCACCCACAGAGGTCATAGACCACTGCTACTATGGTTAATTCTCAGAAGTAATAAACT  400

Query  371  CAGAACCTGAAAGCACATAATGCCTACAAGACTGTTCACTATTTCAAACTTCTGCTCTGG  430
            ||||||||||||||||||||||||||||||||||||||||||||||||||||||||||||
Sbjct  401  CAGAACCTGAAAGCACATAATGCCTACAAGACTGTTCACTATTTCAAACTTCTGCTCTGG  460

Query  431  TTTACATGAATTaaaaaaaaaaaaaC-C-CTCTAtttttttCGGTGGACCCAGGGaaaaa  488
            |||||||||||||||||||||||||  | |||||||||||  | || ||| ||| |||||
Sbjct  461  TTTACATGAATTAAAAAAAAAAAAAATCACTCTATTTTTTCTGCTGTACCAAGGCAAAAA  520

Query  489  aaTTTCACCATCCCCTTTAAAGG  511
            ||||  || ||| | ||||||||
Sbjct  521  AATTCTACAATCACATTTAAAGG  543
```

SNP 49

Sequence ID: **Query_45579** Length: **439** Number of Matches: **1**

Range 1: 42 to 439 Graphics ▼ Next Match ▲

Score	Expect	Identities	Gaps	Strand
725 bits(392)	0.0	397/399(99%)	2/399(0%)	Plus/Plus

```
Query  28   AAC-CAGGGCTGCTTTGGTTGAAGCGAAGATGGGACGCTTGAATGCCCTTTCGTACTACT  86
            ||| ||||||||||||||||||||||||||||||||||||||||||||||||||||||||
Sbjct  42   AACGCAGGGCTGCTTTGGTTGAAGCGAAGATGGGACGCTTGAATGCCCTTTCGTACTACT  101

Query  87   CCCCTTTTACCTCGTTAATACCCACTGACCTATCCTCGTGGAATGCAGGGCTCAAAGAAC  146
            ||||||||||||||||||||||||||||||||||||||||||||||||||||||||||||
Sbjct  102  CCCCTTTTACCTCGTTAATACCCACTGACCTATCCTCGTGGAATGCAGGGCTCAAAGAAC  161

Query  147  AATCTAAAAATCAAACATTATACAAATGCAACCTAAGGAGGAGAGTTCCTTTGAGGCCAG  206
            ||||||||||||||||||||||||||||||||||||||||||||||||||||||||||||
Sbjct  162  AATCTAAAAATCAAACATTATACAAATGCAACCTAAGGAGGAGAGTTCCTTTGAGGCCAG  221

Query  207  GGAGCTACATTATCTTATCTGTATTGCCAGCGCAGAGGCCTACTAGTACATTGTAGGGTC  266
            || |||||||||||||||||||||||||||||||||||||||||||||||||||||||||
Sbjct  222  GG-GCTACATTATCTTATCTGTATTGCCAGCGCAGAGGCCTACTAGTACATTGTAGGGTC  280

Query  267  TAAGTACATTTTTCCTGAATGAAAGGTATTAAATGGTAACTTACGTCTTTATGCACTCTA  326
            ||||||||||||||||||||||||||||||||||||||||||||||||||||||||||||
Sbjct  281  TAAGTACATTTTTCCTGAATGAAAGGTATTAAATGGTAACTTACGTCTTTATGCACTCTA  340

Query  327  TAAACTATGACGTGATCGTCTCCGTCTAACAACTACACTCAAATGCTTACCAAGCTCTTT  386
            ||||||||||||||||||||||||||||||||||||||||||||||||||||||||||||
Sbjct  341  TAAACTATGACGTGATCGTCTCCGTCTAACAACTACACTCAAATGCTTACCAAGCTCTTT  400

Query  387  AAAGGGAAGAATTCCATGGTCGTATGAGCATTCAACAGT  425
            |||||||||||||||||||||||||||||||||||||||
Sbjct  401  AAAGGGAAGAATTCCATGGTCGTATGAGCATTCAACAGT  439
```

Sample 11

SNP 10

```
Sequence ID: Query_119669   Length: 293   Number of Matches: 1

Range 1: 37 to 293  Graphics                                          ▼ Next Match ▲

Score              Expect     Identities          Gaps            Strand
468 bits(253)      7e-137     256/257(99%)        1/257(0%)       Plus/Plus

Query  4    ATA-CATCTTTCTTCTGAGTGTTACCCAGTCAAGTATAAGTAGCCAAATTATTTTTGCAC  62
            ||| ||||||||||||||||||||||||||||||||||||||||||||||||||||||||
Sbjct  37   ATACCATCTTTCTTCTGAGTGTTACCCAGTCAAGTATAAGTAGCCAAATTATTTTTGCAC  96

Query  63   ATCTTTCTGTTTCTCATGTCTTCATTTATTCAACAAGCACTTACTGGGAAGGTCTACACC  122
            ||||||||||||||||||||||||||||||||||||||||||||||||||||||||||||
Sbjct  97   ATCTTTCTGTTTCTCATGTCTTCATTTATTCAACAAGCACTTACTGGGAAGGTCTACACC  156

Query  123  TGCATAGGCAATGCTGGAAAAAGGGTTAAGTAAACCAGGACATGACAATGGTGGCAAATG  182
            ||||||||||||||||||||||||||||||||||||||||||||||||||||||||||||
Sbjct  157  TGCATAGGCAATGCTGGAAAAAGGGTTAAGTAAACCAGGACATGACAATGGTGGCAAATG  216

Query  183  ACTATCAGGTCTTCCCATGTGTTTGACTCAAACTTATTACCCTATGGTCCTTCTGACAAT  242
            ||||||||||||||||||||||||||||||||||||||||||||||||||||||||||||
Sbjct  217  ACTATCAGGTCTTCCCATGTGTTTGACTCAAACTTATTACCCTATGGTCCTTCTGACAAT  276

Query  243  GGCAGAAGGTCTGAATC  259
            |||||||||||||||||
Sbjct  277  GGCAGAAGGTCTGAATC  293
```

SNP 27

```
Sequence ID: Query_38957   Length: 505   Number of Matches: 1

Range 1: 45 to 505  Graphics                                          ▼ Next Match ▲

Score              Expect     Identities          Gaps            Strand
771 bits(417)      0.0        450/463(97%)        13/463(2%)      Plus/Plus

Query  1    GCAAGAACCTTTGTCTC-aaaaaaaCATGTATAGCTACATAATTAATAATATGCAAATAT  59
            ||||| | ||||||||| ||||||||||||||||||||||||||||||||||||||||||
Sbjct  45   GCAAG-A-CTTTGTCTCAAAAAAAACATGTATAGCTACATAATTAATAATATGCAAATAT  102

Query  60   TCTTCTTTAGGGTCACTTAGGTTTTCAACATACAGACCCAAGAGAAGGTACAGAGACTCA  119
            ||||||||||||||||||||||||||||||||||||||||||||||||||||||||||||
Sbjct  103  TCTTCTTTAGGGTCACTTAGGTTTTCAACATACAGACCCAAGAGAAGGTACAGAGACTCA  162

Query  120  TTCTCTCTCTCTTTCAAT----------cacacacacacacacacacacacacacacaca  169
            ||||||||||||||||||          ||||||||||||||||||||||||||||||||
Sbjct  163  TTCTCTCTCTCTTTCAATCACACACACACACACACACACACACACACACACACACACACA  222

Query  170  cCCCTCTTCACTATAATTATAATTACTATGTTGGCTTCCAGATCAGGGGTTAGAGCCTTG  229
            ||||||||||||||||||||||||||||||||||||||||||||||||||||||||||||
Sbjct  223  CCCCTCTTCACTATAATTATAATTACTATGTTGGCTTCCAGATCAGGGGTTAGAGCCTTG  282

Query  230  GCATGGAGACGCCTGAAAGGCACCCAAGGCAATTAGTGGTGTCCCTTCTCCACCCCCTAC  289
            ||||||||||||||||||||||||||||||||||||||||||||||||||||||||||||
Sbjct  283  GCATGGAGACGCCTGAAAGGCACCCAAGGCAATTAGTGGTGTCCCTTCTCCACCCCCTAC  342

Query  290  ATACCTTCAGGCCCTTTGCACTTGCTGCTCCCCCTCCCAGTGCACACACCCCCAGATCCT  349
            ||||||||||||||||||||||||||||||||||||||||||||||||||||||||||||
Sbjct  343  ATACCTTCAGGCCCTTTGCACTTGCTGCTCCCCCTCCCAGTGCACACACCCCCAGATCCT  402

Query  350  TGTGTGGCTCCCTCTCCCACCACATTTTGGTCTTAGCTCAAATGCCACCTCCTCAGAAAT  409
            ||||||||||||||||||||||||||||||||||||||||||||||||||||||||||||
Sbjct  403  TGTGTGGCTCCCTCTCCCACCACATTTTGGTCTTAGCTCAAATGCCACCTCCTCAGAAAT  462

Query  410  GCCTGGCCTGGCCGCCTTCAGGGCTGTCTATATGCTCACCAGC  452
            |||||||||||||||||||||||||||||||||||||||||||
Sbjct  463  GCCTGGCCTGGCCGCCTTCAGGGCTGTCTATATGCTCACCAGC  505
```

SNP 29

```
Sequence ID: Query_49711   Length: 384   Number of Matches: 1

Range 1: 48 to 384  Graphics                                          ▼ Next Match ▲

Score              Expect     Identities          Gaps            Strand
616 bits(333)      0.0        336/337(99%)        1/337(0%)       Plus/Plus

Query  1    TCTGC-CACGTATCCCAGAACTTAAAGTATAATTTTAAAAAAGATGTTTTATAATCTAAT  59
            ||||| ||||||||||||||||||||||||||||||||||||||||||||||||||||||
Sbjct  48   TCTGCACACGTATCCCAGAACTTAAAGTATAATTTTAAAAAAGATGTTTTATAATCTAAT  107

Query  60   TGGTGACTGACAACTAAGGTACGTTAGACCATTAGCAAAAGTAAATTAAGTGATAAGTAT  119
            ||||||||||||||||||||||||||||||||||||||||||||||||||||||||||||
Sbjct  108  TGGTGACTGACAACTAAGGTACGTTAGACCATTAGCAAAAGTAAATTAAGTGATAAGTAT  167

Query  120  TATGGTAGGGACCTCAGGTAATATAGGAATTGGGCAGAGAGAGACGTTAACATGGTTAAC  179
            ||||||||||||||||||||||||||||||||||||||||||||||||||||||||||||
Sbjct  168  TATGGTAGGGACCTCAGGTAATATAGGAATTGGGCAGAGAGAGACGTTAACATGGTTAAC  227

Query  180  TCCAAAAGTGGTATGACCTAGAAAATGGGTAGATAGGTTTGAGTAAGTGGAAGGAGAGAA  239
            ||||||||||||||||||||||||||||||||||||||||||||||||||||||||||||
Sbjct  228  TCCAAAAGTGGTATGACCTAGAAAATGGGTAGATAGGTTTGAGTAAGTGGAAGGAGAGAA  287

Query  240  AAGAAATTTCTAGGCATAGACGCAGTGTTTCTCAAGAAACTGAAAGACAAATATGCAGGT  299
            ||||||||||||||||||||||||||||||||||||||||||||||||||||||||||||
Sbjct  288  AAGAAATTTCTAGGCATAGACGCAGTGTTTCTCAAGAAACTGAAAGACAAATATGCAGGT  347

Query  300  CCTGCTTATGGAAAAGGGAAGACAGAAGGTGTCTTGC  336
            |||||||||||||||||||||||||||||||||||||
Sbjct  348  CCTGCTTATGGAAAAGGGAAGACAGAAGGTGTCTTGC  384
```

SNP 46

Sequence ID: **Query_53685** Length: **551** Number of Matches: **1**

Range 1: 43 to 550 Graphics ▼ Next Match ▲

Score	Expect	Identities	Gaps	Strand
909 bits(492)	0.0	503/508(99%)	1/508(0%)	Plus/Plus

```
Query  8    AAATCAAGGAATCCAAATAAAATACCACGTTTAATTCAATTTATCACGTCCAAGAAGTTA  67
            |||||||| |||||||||||||||||||||||||||||||||||||||||||||||||||
Sbjct  43   AAATCAAGAAATCCAAATAAAATACCACGTTTAATTCAATTTATCACGTCCAAGAAGTTA  102

Query  68   CAAAGGCAATGTAGAAGAAGTAAGTAAAAATGGGGAAGCCCTAATTTTTAAGTCTGCCTT  127
            ||||||||||||||||||||||||||||||||||||||||||||||||||||||||||||
Sbjct  103  CAAAGGCAATGTAGAAGAAGTAAGTAAAAATGGGGAAGCCCTAATTTTTAAGTCTGCCTT  162

Query  128  GAAAACAATTTTTTCTTATCAATGAGACTTTCTAATAGGACTCATCTTGCCATTGGCACC  187
            ||||||||||||||||||||||||||||||||||||||||||||||||||||||||||||
Sbjct  163  GAAAACAATTTTTTCTTATCAATGAGACTTTCTAATAGGACTCATCTTGCCATTGGCACC  222

Query  188  CACAAAATCTAACCACCCACCTCCACTTCTCCGGAAGGATCCCTTCCTAATGTCTGATGC  247
            |||||||||||||||||||||||||||||||||| |||||||||||||||||||||||||
Sbjct  223  CACAAAATCTAACCACCCACCTCCACTTCTCCGGGAGGATCCCTTCCTAATGTCTGATGC  282

Query  248  CTCTTCCCTCACCTCCATTCTTTGCCCACTGCTGCACATGCTTAAAGCCCAGCCACCCTT  307
            ||||||||||||||||||||||||||||||||||||||||||||||||||||||||||||
Sbjct  283  CTCTTCCCTCACCTCCATTCTTTGCCCACTGCTGCACATGCTTAAAGCCCAGCCACCCTT  342

Query  308  TCACCCACAGAGGTCATAGACCACTGCTACTATGGTTAATTCTCAGAAGTAATAAACTCA  367
            ||||||||||||||||||||||||||||||||||||||||||||||||||||||||||||
Sbjct  343  TCACCCACAGAGGTCATAGACCACTGCTACTATGGTTAATTCTCAGAAGTAATAAACTCA  402

Query  368  GAACCTGAAAGCACATAATGCCTACAAGACTGTTCACTATTTCAAACTTCTGCTCTGGTT  427
            ||||||||||||||||||||||||||||||||||||||||||||||||||||||||||||
Sbjct  403  GAACCTGAAAGCACATAATGCCTACAAGACTGTTCACTATTTCAAACTTCTGCTCTGGTT  462

Query  428  TACATGAATT-aaaaaaaaaaaaaTCACTCTATTTTTTCTGCTGTACCAAGGCaaaaaaa  486
            |||||||||| |||||||||||||||||||||||||||||||||||||||||||||||||
Sbjct  463  TACATGAATTAAAAAAAAAAAAAATCACTCTATTTTTTCTGCTGTACCAAGGCAAAAAAA  522

Query  487  TTCTACAATCCCTTTTAAAGGCATGGAC  514
            |||||||||| | |||||||||||||||
Sbjct  523  TTCTACAATCACATTTAAAGGCATGGAC  550
```

SNP 49

Sequence ID: **Query_28853** Length: **439** Number of Matches: **1**

Range 1: 45 to 439 Graphics ▼ Next Match ▲

Score	Expect	Identities	Gaps	Strand
713 bits(386)	0.0	394/397(99%)	3/397(0%)	Plus/Plus

```
Query  23   GCAGAGGCTGCTTTGGTTG-AGCGAAGATGGGACGCTTGAATGCCCTTTCGTACTACTCC  81
            |||| |||||||||||||| ||||||||||||||||||||||||||||||||||||||||
Sbjct  45   GCAG-GGCTGCTTTGGTTGAAGCGAAGATGGGACGCTTGAATGCCCTTTCGTACTACTCC  103

Query  82   CCTTTTACCTCGTTAATACCCACTGACCTATCCTCGTGGAATGCAGGGCTCAAAGAACAA  141
            ||||||||||||||||||||||||||||||||||||||||||||||||||||||||||||
Sbjct  104  CCTTTTACCTCGTTAATACCCACTGACCTATCCTCGTGGAATGCAGGGCTCAAAGAACAA  163

Query  142  TCTAAAAATCAAACATTATACAAATGCAACCTAAGGAGGAGAGTTCCTTTGAGGCCAGGG  201
            ||||||||||||||||||||||||||||||||||||||||||||||||||||||||||||
Sbjct  164  TCTAAAAATCAAACATTATACAAATGCAACCTAAGGAGGAGAGTTCCTTTGAGGCCAGGG  223

Query  202  AGCTACATTATCTTATCTGTATTGCCAGCGCAGAGGCCTACTAGTACATTGTAGGGTCTA  261
             |||||||||||||||||||||||||||||||||||||||||||||||||||||||||||
Sbjct  224  -GCTACATTATCTTATCTGTATTGCCAGCGCAGAGGCCTACTAGTACATTGTAGGGTCTA  282

Query  262  AGTACATTTTTCCTGAATGAAAGGTATTAAATGGTAACTTACGTCTTTATGCACTCTATA  321
            ||||||||||||||||||||||||||||||||||||||||||||||||||||||||||||
Sbjct  283  AGTACATTTTTCCTGAATGAAAGGTATTAAATGGTAACTTACGTCTTTATGCACTCTATA  342

Query  322  AACTATGACGTGATCGTCTCCGTCTAACAACTACACTCAAATGCTTACCAAGCTCTTTAA  381
            ||||||||||||||||||||||||||||||||||||||||||||||||||||||||||||
Sbjct  343  AACTATGACGTGATCGTCTCCGTCTAACAACTACACTCAAATGCTTACCAAGCTCTTTAA  402

Query  382  AGGGAAGAATTCCATGGTCGTATGAGCATTCAACAGT  418
            |||||||||||||||||||||||||||||||||||||
Sbjct  403  AGGGAAGAATTCCATGGTCGTATGAGCATTCAACAGT  439
```

Sample 12

SNP 10

Sequence ID: Query_9597 **Length:** 293 **Number of Matches:** 1

Range 1: 37 to 293 Graphics ▼ Next Match ▲ P

Score	Expect	Identities	Gaps	Strand
460 bits(249)	1e-134	256/259(99%)	2/259(0%)	Plus/Plus

```
Query  4    ATACAATCTTATCTTCTGAGTGTTACCCAGTCAAGTATAAGTAGCCAAATTATTTTTGCA  63
            |||| ||||| |||||||||||||||||||||||||||||||||||||||||||||||||
Sbjct  37   ATACCATCTT-TCTTCTGAGTGTTACCCAGTCAAGTATAAGTAGCCAAATTATTTTTGCA  95

Query  64   CATCTTTCTGTTTCTCATGTCTTCATTTATTCAACAAGCACTTACTGGGAAGGTCTACAA  123
            |||||||||||||||||||||||||||||||||||||||||||||||||||||||||| |
Sbjct  96   CATCTTTCTGTTTCTCATGTCTTCATTTATTCAACAAGCACTTACTGGGAAGGTCTAC-A  154

Query  124  CCTGCATAGGCAATGCTGGAAAAAGGGTTAAGTAAACCAGGACATGACAATGGTGGCAAA  183
            ||||||||||||||||||||||||||||||||||||||||||||||||||||||||||||
Sbjct  155  CCTGCATAGGCAATGCTGGAAAAAGGGTTAAGTAAACCAGGACATGACAATGGTGGCAAA  214

Query  184  TGACTATCAGGTCTTCCCATGTGTTTGACTCAAACTTATTACCCTATGGTCCTTCTGACA  243
            ||||||||||||||||||||||||||||||||||||||||||||||||||||||||||||
Sbjct  215  TGACTATCAGGTCTTCCCATGTGTTTGACTCAAACTTATTACCCTATGGTCCTTCTGACA  274

Query  244  ATGGCAGAAGGTCTGAATC  262
            |||||||||||||||||||
Sbjct  275  ATGGCAGAAGGTCTGAATC  293
```

SNP 27

Sequence ID: Query_193123 **Length:** 505 **Number of Matches:** 1

Range 1: 34 to 505 Graphics ▼ Next Match ▲ P

Score	Expect	Identities	Gaps	Strand
795 bits(430)	0.0	460/472(97%)	11/472(2%)	Plus/Plus

```
Query  3    TGGGGGAC--AGC-AGACTTTGTCTCaaaaaaaaCATGTATAGCTACATAATTAATAATA  59
            |||| |||  ||| ||||||||||||||||||||||||||||||||||||||||||||||
Sbjct  34   TGGGTGACAAAGCAAGACTTTGTCTCAAAAAAAACATGTATAGCTACATAATTAATAATA  93

Query  60   TGCAAATATTCTTCTTTAGGGTCACTTAGGTTTTCAACATACAGACCCAAGAGAAGGTAC  119
            ||||||||||||||||||||||||||||||||||||||||||||||||||||||||||||
Sbjct  94   TGCAAATATTCTTCTTTAGGGTCACTTAGGTTTTCAACATACAGACCCAAGAGAAGGTAC  153

Query  120  AGAGACTCATTCTCTCTCTCTTTCAAT--------cacacacacacacacacacacacac  171
            |||||||||||||||||||||||||||        |||||||||||||||||||||||||
Sbjct  154  AGAGACTCATTCTCTCTCTCTTTCAATCACACACACACACACACACACACACACACACAC  213

Query  172  acacacacacCCCTCTTCACTATAATTATAATTACTATGTTGGCTTCCAGATCAGGGGTT  231
            ||||||||||||||||||||||||||||||||||||||||||||||||||||||||||||
Sbjct  214  ACACACACACCCCTCTTCACTATAATTATAATTACTATGTTGGCTTCCAGATCAGGGGTT  273

Query  232  AGAGCCTTGGCATGGAGACGCCTGAAAGGCACCCAAGGCAATTAGTGGTGTCCCTTCTCC  291
            ||||||||||||||||||||||||||||||||||||||||||||||||||||||||||||
Sbjct  274  AGAGCCTTGGCATGGAGACGCCTGAAAGGCACCCAAGGCAATTAGTGGTGTCCCTTCTCC  333

Query  292  ACCCCCTACATACCTTCAGGCCCTTTGCACTTGCTGCTCCCCCTCCCAGTGCACACACCC  351
            ||||||||||||||||||||||||||||||||||||||||||||||||||||||||||||
Sbjct  334  ACCCCCTACATACCTTCAGGCCCTTTGCACTTGCTGCTCCCCCTCCCAGTGCACACACCC  393

Query  352  CCAGATCCTTGTGTGGCTCCCTCTCCCACCACATTTTGGTCTTAGCTCAAATGCCACCTC  411
            ||||||||||||||||||||||||||||||||||||||||||||||||||||||||||||
Sbjct  394  CCAGATCCTTGTGTGGCTCCCTCTCCCACCACATTTTGGTCTTAGCTCAAATGCCACCTC  453

Query  412  CTCAGAAATGCCTGGCCTGGCCGCCTTCAGGGCTGTCTATATGCTCACCAGC  463
            ||||||||||||||||||||||||||||||||||||||||||||||||||||
Sbjct  454  CTCAGAAATGCCTGGCCTGGCCGCCTTCAGGGCTGTCTATATGCTCACCAGC  505
```

SNP 29

Sequence ID: Query_9035 **Length:** 384 **Number of Matches:** 1

Range 1: 58 to 384 Graphics ▼ Next Match ▲

Score	Expect	Identities	Gaps	Strand
599 bits(324)	4e-176	327/328(99%)	1/328(0%)	Plus/Plus

```
Query  1    TATCCCAGAACTTAAAGTATAATTTTAAAAAAGATGTTTTATAATCTAATTGGTGACTGA  60
            ||||||||||||||||||||||||||||||||||||||||||||||||||||||||||||
Sbjct  58   TATCCCAGAACTTAAAGTATAATTTTAAAAAAGATGTTTTATAATCTAATTGGTGACTGA  117

Query  61   CAACTAAGGTACGTTAGACCATTAGCAAAAGTAAATTAAGTGATAAGTATTATGGTAGGG  120
            ||||||||||||||||||||||||||||||||||||||||||||||||||||||||||||
Sbjct  118  CAACTAAGGTACGTTAGACCATTAGCAAAAGTAAATTAAGTGATAAGTATTATGGTAGGG  177

Query  121  ACCTCAGGTAATATAGGAATTGGGCAGAGAGAGACGTTAACATGGTTAACTCCAAAAGTG  180
            ||||||||||||||||||||||||||||||||||||||||||||||||||||||||||||
Sbjct  178  ACCTCAGGTAATATAGGAATTGGGCAGAGAGAGACGTTAACATGGTTAACTCCAAAAGTG  237

Query  181  GTATGACCTAGAAAATGGGTAGATAGGTTTGAGTAAGTGGAAGGAGAGAAAAGAAATTTC  240
            ||||||||||||||||||||||||||||||||||||||||||||||||||||||||||||
Sbjct  238  GTATGACCTAGAAAATGGGTAGATAGGTTTGAGTAAGTGGAAGGAGAGAAAAGAAATTTC  297

Query  241  TAGGCATAGACGCAGTGTTTCTCAAGAAACTGAAAGACAAATATGCAGGTCCTGCTTATG  300
            ||||||||||||||||||||||||||||||||||||||||||||||||||||||||||||
Sbjct  298  TAGGCATAGACGCAGTGTTTCTCAAGAAACTGAAAGACAAATATGCAGGTCCTGCTTATG  357

Query  301  GAAAAGGGAAGACAGAAGGTTGTCTTGC  328
            |||||||||||||||||||| |||||||
Sbjct  358  GAAAAGGGAAGACAGAAGGT-GTCTTGC  384
```

SNP 46

Sequence ID: **Query_19557** Length: **551** Number of Matches: **1**

Range 1: 42 to 543 Graphics ▼ Next Match ▲

Score	Expect	Identities	Gaps	Strand
832 bits(450)	0.0	486/503(97%)	4/503(0%)	Plus/Plus

```
Query  8    AAAATC-AG-AATCCAAATAAAATACCACGTTTAATTCAATTTATCACGTCCAAGAAGTT  65
            |||||| || ||||||||||||||||||||||||||||||||||||||||||||||||||
Sbjct  42   AAAATCAAGAAATCCAAATAAAATACCACGTTTAATTCAATTTATCACGTCCAAGAAGTT  101

Query  66   ACAAAGGCAATGTAGAAGAAGTAAGTAAAAATGGGGAAGCCCTAATTTTTAAGTCTGCCT  125
            ||||||||||||||||||||||||||||||||||||||||||||||||||||||||||||
Sbjct  102  ACAAAGGCAATGTAGAAGAAGTAAGTAAAAATGGGGAAGCCCTAATTTTTAAGTCTGCCT  161

Query  126  TGAAAACAATTTTTTCTTATCAATGAGACTTTCTAATAGGACTCATCTTGCCATTGGCAC  185
            ||||||||||||||||||||||||||||||||||||||||||||||||||||||||||||
Sbjct  162  TGAAAACAATTTTTTCTTATCAATGAGACTTTCTAATAGGACTCATCTTGCCATTGGCAC  221

Query  186  CCACAAAATCTAACCACCCACCTCCACTTCTCCGGAGAGGATCCCTTCCTAATGTCTGAT  245
            ||||||||||||||||||||||||||||||||||| ||||||||||||||||||||||||
Sbjct  222  CCACAAAATCTAACCACCCACCTCCACTTCTCCGG-GAGGATCCCTTCCTAATGTCTGAT  280

Query  246  GCCTCTTCCCTCACCTCCATTCTTTGCCCACTGCTGCACATGCTTAAAGCCCAGCCACCC  305
            ||||||||||||||||||||||||||||||||||||||||||||||||||||||||||||
Sbjct  281  GCCTCTTCCCTCACCTCCATTCTTTGCCCACTGCTGCACATGCTTAAAGCCCAGCCACCC  340

Query  306  TTTCACCCACAGAGGTCATAGACCACTGCTACTATGGTTAATTCTCAGAAGTAATAAACT  365
            ||||||||||||||||||||||||||||||||||||||||||||||||||||||||||||
Sbjct  341  TTTCACCCACAGAGGTCATAGACCACTGCTACTATGGTTAATTCTCAGAAGTAATAAACT  400

Query  366  CAGAACCTGAAAGCACATAATGCCTACAAGACTGTTCACTATTTCAAACTTCTGCTCTGG  425
            ||||||||||||||||||||||||||||||||||||||||||||||||||||||||||||
Sbjct  401  CAGAACCTGAAAGCACATAATGCCTACAAGACTGTTCACTATTTCAAACTTCTGCTCTGG  460

Query  426  TTTACATGAATTaaaaaaaaaaaaaaCCCCCtttttttttCGG-TGGACCCAGGGaaaaa  484
            |||||||||||||||||||||||||| | |  | ||||||| | || ||| ||| |||||
Sbjct  461  TTTACATGAATTAAAAAAAAAAAAAAATCACTCTATTTTTTCTGCTGTACCAAGGCAAAAA  520

Query  485  aaTTTCACAATCCCCTTTAAAgg  507
            ||||  |||||| | ||||||||
Sbjct  521  AATTCTACAATCACATTTAAAGG  543
```

SNP 49

Sequence ID: **Query_58995** Length: **439** Number of Matches: **1**

Range 1: 38 to 439 Graphics ▼ Next Match ▲

Score	Expect	Identities	Gaps	Strand
719 bits(389)	0.0	399/403(99%)	3/403(0%)	Plus/Plus

```
Query  8    GGAAAA-GCAGAGCTGCTTTGGTTG-AGCGAAGATGGACGCTTGAATGCCCTTTCGTAC  65
            |||||| |||| ||||||||||||| ||||||||||||||||||||||||||||||||||
Sbjct  38   GGAAAACGCAGGGCTGCTTTGGTTGAAGCGAAGATGGACGCTTGAATGCCCTTTCGTAC  97

Query  66   TACTCCCCTTTTACCTCGTTAATACCCACTGACCTATCCTCGTGGAATGCAGGGCTCAAA  125
            ||||||||||||||||||||||||||||||||||||||||||||||||||||||||||||
Sbjct  98   TACTCCCCTTTTACCTCGTTAATACCCACTGACCTATCCTCGTGGAATGCAGGGCTCAAA  157

Query  126  GAACAATCTAAAAATCAAACATTATACAAATGCAACCTAAGGAGGAGAGTTCCTTTGAGG  185
            ||||||||||||||||||||||||||||||||||||||||||||||||||||||||||||
Sbjct  158  GAACAATCTAAAAATCAAACATTATACAAATGCAACCTAAGGAGGAGAGTTCCTTTGAGG  217

Query  186  CCAGGGAGCTACATTATCTTATCTGTATTGCCAGCGCAGAGGCCTACTAGTACATTGTAG  245
            |||||| |||||||||||||||||||||||||||||||||||||||||||||||||||||
Sbjct  218  CCAGGG-GCTACATTATCTTATCTGTATTGCCAGCGCAGAGGCCTACTAGTACATTGTAG  276

Query  246  GGTCTAAGTACATTTTTCCTGAATGAAAGGTATTAAATGGTAACTTACGTCTTTATGCAC  305
            ||||||||||||||||||||||||||||||||||||||||||||||||||||||||||||
Sbjct  277  GGTCTAAGTACATTTTTCCTGAATGAAAGGTATTAAATGGTAACTTACGTCTTTATGCAC  336

Query  306  TCTATAAACTATGACGTGATCGTCTCCGTCTAACAACTACACTCAAATGCTTACCAAGCT  365
            ||||||||||||||||||||||||||||||||||||||||||||||||||||||||||||
Sbjct  337  TCTATAAACTATGACGTGATCGTCTCCGTCTAACAACTACACTCAAATGCTTACCAAGCT  396

Query  366  CTTTAAAGGGAAGAATTCCATGGTCGTATGAGCATTCAACAGT  408
            |||||||||||||||||||||||||||||||||||||||||||
Sbjct  397  CTTTAAAGGGAAGAATTCCATGGTCGTATGAGCATTCAACAGT  439
```

Sample 13

SNP 10

```
Sequence ID: Query_1027  Length: 293  Number of Matches: 1

Range 1: 44 to 293  Graphics                                    ▼ Next Match  ▲ P

Score            Expect    Identities       Gaps         Strand
457 bits(247)    2e-133    249/250(99%)     0/250(0%)    Plus/Plus

Query  5    CTTTCTTCTGAGTGTTACCCAGTCAAGTATAAGTAGCCAAATTATTTTTGCACATCTTTC  64
            ||||||||||||||||||||||||||||||||||||||||||||||||||||||||||||
Sbjct  44   CTTTCTTCTGAGTGTTACCCAGTCAAGTATAAGTAGCCAAATTATTTTTGCACATCTTTC  103

Query  65   TGTTTCTCATGTCTTCATTTATTCAACAAGCACTTACTGGGAAGGTCTACAACTGCATAG  124
            ||||||||||||||||||||||||||||||||||||||||||||||||||| ||||||||
Sbjct  104  TGTTTCTCATGTCTTCATTTATTCAACAAGCACTTACTGGGAAGGTCTACACCTGCATAG  163

Query  125  GCAATGCTGGAAAAAGGGTTAAGTAAACCAGGACATGACAATGGTGGCAAATGACTATCA  184
            ||||||||||||||||||||||||||||||||||||||||||||||||||||||||||||
Sbjct  164  GCAATGCTGGAAAAAGGGTTAAGTAAACCAGGACATGACAATGGTGGCAAATGACTATCA  223

Query  185  GGTCTTCCCATGTGTTTGACTCAAACTTATTACCCTATGGTCCTTCTGACAATGGCAGAA  244
            ||||||||||||||||||||||||||||||||||||||||||||||||||||||||||||
Sbjct  224  GGTCTTCCCATGTGTTTGACTCAAACTTATTACCCTATGGTCCTTCTGACAATGGCAGAA  283

Query  245  GGTCTGAATC  254
            ||||||||||
Sbjct  284  GGTCTGAATC  293
```

SNP 27

```
Sequence ID: Query_65319  Length: 505  Number of Matches: 1

Range 1: 70 to 505  Graphics                                    ▼ Next Match  ▲ Pre

Score            Expect    Identities       Gaps         Strand
750 bits(406)    0.0       429/438(98%)     9/438(2%)    Plus/Plus

Query  1    TGTAATAGCCTACATAATTAATAATATGCAAATATTCTTCTTTAGGGTCACTTAGGTTTT  60
            ||| |||| |||||||||||||||||||||||||||||||||||||||||||||||||||
Sbjct  70   TGT-ATAG-CTACATAATTAATAATATGCAAATATTCTTCTTTAGGGTCACTTAGGTTTT  127

Query  61   CAACATACAGACCCAAGAGAAGGTACAGAGACTCATTCTCTCTCTCTTTCAATcacacac  120
            ||||||||||||||||||||||||||||||||||||||||||||||||||||||||||||
Sbjct  128  CAACATACAGACCCAAGAGAAGGTACAGAGACTCATTCTCTCTCTCTTTCAATCACACAC  187

Query  121  acacacacacacacacacacacacacac-------CCCCTCTTCACTATAATTATAATTA  173
            ||||||||||||||||||||||||||||       |||||||||||||||||||||||||
Sbjct  188  ACACACACACACACACACACACACACACACACACACCCCTCTTCACTATAATTATAATTA  247

Query  174  CTATGTTGGCTTCCAGATCAGGGGTTAGAGCCTTGGCATGGAGACGCCTGAAAGGCACCC  233
            ||||||||||||||||||||||||||||||||||||||||||||||||||||||||||||
Sbjct  248  CTATGTTGGCTTCCAGATCAGGGGTTAGAGCCTTGGCATGGAGACGCCTGAAAGGCACCC  307

Query  234  AAGGCAATTAGTGGTGTCCCTTCTCCACCCCCTACATACCTTCAGGCCCTTTGCACTTGC  293
            ||||||||||||||||||||||||||||||||||||||||||||||||||||||||||||
Sbjct  308  AAGGCAATTAGTGGTGTCCCTTCTCCACCCCCTACATACCTTCAGGCCCTTTGCACTTGC  367

Query  294  TGCTCCCCCTCCCAGTGCACACACCCCCAGATCCTTGTGTGGCTCCCTCTCCCACCACAT  353
            ||||||||||||||||||||||||||||||||||||||||||||||||||||||||||||
Sbjct  368  TGCTCCCCCTCCCAGTGCACACACCCCCAGATCCTTGTGTGGCTCCCTCTCCCACCACAT  427

Query  354  TTTGGTCTTAGCTCAAATGCCACCTCCTCAGAAATGCCTGGCCTGGCCGCCTTCAGGGCT  413
            ||||||||||||||||||||||||||||||||||||||||||||||||||||||||||||
Sbjct  428  TTTGGTCTTAGCTCAAATGCCACCTCCTCAGAAATGCCTGGCCTGGCCGCCTTCAGGGCT  487

Query  414  GTCTATATGCTCACCAGC  431
            ||||||||||||||||||
Sbjct  488  GTCTATATGCTCACCAGC  505
```

SNP 29

```
Sequence ID: Query_18153  Length: 384  Number of Matches: 1

Range 1: 36 to 383  Graphics                                    ▼ Next Match

Score            Expect    Identities       Gaps         Strand
627 bits(339)    0.0       347/350(99%)     3/350(0%)    Plus/Plus

Query  2    AAACCTG-CCATTCTGCACACGTATCCCAGAACTTAAAGTATAATTTTAAAAAAGATGTT  60
            ||||||| ||||||||||||||||||||||||||||||||||||||||||||||||||||
Sbjct  36   AAACCTGCCCATTCTGCACACGTATCCCAGAACTTAAAGTATAATTTTAAAAAAGATGTT  95

Query  61   TTATAATCTAATTGGTGACTGACAACTAAGGTACGTTAGACCATTAGCAAAAGTAAATTA  120
            ||||||||||||||||||||||||||||||||||||||||||||||||||||||||||||
Sbjct  96   TTATAATCTAATTGGTGACTGACAACTAAGGTACGTTAGACCATTAGCAAAAGTAAATTA  155

Query  121  AGTGATAAGTATTATGGTAGGGACCTCAGGTAGATATAGGAATTGGGCAGAGAGAGACGT  180
            |||||||||||||||||||||||||||||||| |||||||||||||||||||||||||||
Sbjct  156  AGTGATAAGTATTATGGTAGGGACCTCAGGTA-ATATAGGAATTGGGCAGAGAGAGACGT  214

Query  181  TAACATGGTTAACTCCAAAAGTGGTATGACCTAGAAAATGGGTAGATAGGTTTGAGTAAG  240
            ||||||||||||||||||||||||||||||||||||||||||||||||||||||||||||
Sbjct  215  TAACATGGTTAACTCCAAAAGTGGTATGACCTAGAAAATGGGTAGATAGGTTTGAGTAAG  274

Query  241  TGGAAGGAGAGAAAAGAAATTTCTAGGCATAGACGCAGTGTTTCTCAAGAAACTGAAAGA  300
            ||||||||||||||||||||||||||||||||||||||||||||||||||||||||||||
Sbjct  275  TGGAAGGAGAGAAAAGAAATTTCTAGGCATAGACGCAGTGTTTCTCAAGAAACTGAAAGA  334

Query  301  CAAATATGCAGGTCCTGCTTATGGAAAAGGGAAGACAGAAGGGTGTCTTG  350
            |||||||||||||||||||||||||||||||||||||||||| |||||||
Sbjct  335  CAAATATGCAGGTCCTGCTTATGGAAAAGGGAAGACAGAAGG-TGTCTTG  383
```

SNP 46

Sequence ID: **Query_9533** Length: **551** Number of Matches: **1**

Range 1: 42 to 551 Graphics ▼ Next Match ▲

Score	Expect	Identities	Gaps	Strand
913 bits(494)	0.0	505/510(99%)	2/510(0%)	Plus/Plus

```
Query  10   AAAATCAAG-AATCCAAATAAAATACCACGTTTAATTCAATTTATCACGTCCAAGAAGTT  68
            ||||||||| ||||||||||||||||||||||||||||||||||||||||||||||||||
Sbjct  42   AAAATCAAGAAATCCAAATAAAATACCACGTTTAATTCAATTTATCACGTCCAAGAAGTT  101

Query  69   ACAAAGGCAATGTAGAAGAAGTAAGTAAAAATGGGGAAGCCCTAATTTTTAAGTCTGCCT  128
            ||||||||||||||||||||||||||||||||||||||||||||||||||||||||||||
Sbjct  102  ACAAAGGCAATGTAGAAGAAGTAAGTAAAAATGGGGAAGCCCTAATTTTTAAGTCTGCCT  161

Query  129  TGAAAACAATTTTTTCTTATCAATGAGACTTTCTAATAGGACTCATCTTGCCATTGGCAC  188
            ||||||||||||||||||||||||||||||||||||||||||||||||||||||||||||
Sbjct  162  TGAAAACAATTTTTTCTTATCAATGAGACTTTCTAATAGGACTCATCTTGCCATTGGCAC  221

Query  189  CCACAAAATCTAACCACCCACCTCCACTTCTCCGGAAGGATCCCTTCCTAATGTCTGATG  248
            |||||||||||||||||||||||||||||||||| |||||||||||||||||||||||||
Sbjct  222  CCACAAAATCTAACCACCCACCTCCACTTCTCCGGGAGGATCCCTTCCTAATGTCTGATG  281

Query  249  CCTCTTCCCTCACCTCCATTCTTTGCCCACTGCTGCACATGCTTAAAGCCCAGCCACCCT  308
            ||||||||||||||||||||||||||||||||||||||||||||||||||||||||||||
Sbjct  282  CCTCTTCCCTCACCTCCATTCTTTGCCCACTGCTGCACATGCTTAAAGCCCAGCCACCCT  341

Query  309  TTCACCCACAGAGGTCATAGACCACTGCTACTATGGTTAATTCTCAGAAGTAATAAACTC  368
            ||||||||||||||||||||||||||||||||||||||||||||||||||||||||||||
Sbjct  342  TTCACCCACAGAGGTCATAGACCACTGCTACTATGGTTAATTCTCAGAAGTAATAAACTC  401

Query  369  AGAACCTGAAAGCACATAATGCCTACAAGACTGTTCACTATTTCAAACTTCTGCTCTGGT  428
            ||||||||||||||||||||||||||||||||||||||||||||||||||||||||||||
Sbjct  402  AGAACCTGAAAGCACATAATGCCTACAAGACTGTTCACTATTTCAAACTTCTGCTCTGGT  461

Query  429  TTACATGAATT-aaaaaaaaaaaaaTCACTCTATTTTTTCTGCTGTACCAAGGCaaaaaa  487
            ||||||||||| ||||||||||||||||||||||||||||||||||||||||||||||||
Sbjct  462  TTACATGAATTAAAAAAAAAAAAAATCACTCTATTTTTTCTGCTGTACCAAGGCAAAAAA  521

Query  488  aTTCTACAATCCCTTTTAAAGGCATGGACT  517
            ||||||||||| | ||||||||||||||||
Sbjct  522  ATTCTACAATCACATTTAAAGGCATGGACT  551
```

SNP 49

Sequence ID: **Query_56067** Length: **439** Number of Matches: **1**

Range 1: 38 to 439 Graphics ▼ Next Match ▲

Score	Expect	Identities	Gaps	Strand
713 bits(386)	0.0	398/403(99%)	3/403(0%)	Plus/Plus

```
Query  5    GGTAAA-GCAGAGCTGCTTTGGTTG-AGCGAAGATGGGACGCTTGAATGCCCTTTCGTAC  62
            || ||| |||| ||||||||||||| ||||||||||||||||||||||||||||||||||
Sbjct  38   GGAAAACGCAGGGCTGCTTTGGTTGAAGCGAAGATGGGACGCTTGAATGCCCTTTCGTAC  97

Query  63   TACTCCCCTTTTACCTCGTTAATACCCACTGACCTATCCTCGTGGAATGCAGGGCTCAAA  122
            ||||||||||||||||||||||||||||||||||||||||||||||||||||||||||||
Sbjct  98   TACTCCCCTTTTACCTCGTTAATACCCACTGACCTATCCTCGTGGAATGCAGGGCTCAAA  157

Query  123  GAACAATCTAAAAATCAAACATTATACAAATGCAACCTAAGGAGGAGAGTTCCTTTGAGG  182
            ||||||||||||||||||||||||||||||||||||||||||||||||||||||||||||
Sbjct  158  GAACAATCTAAAAATCAAACATTATACAAATGCAACCTAAGGAGGAGAGTTCCTTTGAGG  217

Query  183  CCAGGGAGCTACATTATCTTATCTGTATTGCCAGCGCAGAGGCCTACTAGTACATTGTAG  242
            |||||| |||||||||||||||||||||||||||||||||||||||||||||||||||||
Sbjct  218  CCAGGG-GCTACATTATCTTATCTGTATTGCCAGCGCAGAGGCCTACTAGTACATTGTAG  276

Query  243  GGTCTAAGTACATTTTTCCTGAATGAAAGGTATTAAATGGTAACTTACGTCTTTATGCAC  302
            ||||||||||||||||||||||||||||||||||||||||||||||||||||||||||||
Sbjct  277  GGTCTAAGTACATTTTTCCTGAATGAAAGGTATTAAATGGTAACTTACGTCTTTATGCAC  336

Query  303  TCTATAAACTATGACGTGATCGTCTCCGTCTAACAACTACACTCAAATGCTTACCAAGCT  362
            ||||||||||||||||||||||||||||||||||||||||||||||||||||||||||||
Sbjct  337  TCTATAAACTATGACGTGATCGTCTCCGTCTAACAACTACACTCAAATGCTTACCAAGCT  396

Query  363  CTTTAAAGGGAAGAATTCCATGGTCGTATGAGCATTCAACAGT  405
            |||||||||||||||||||||||||||||||||||||||||||
Sbjct  397  CTTTAAAGGGAAGAATTCCATGGTCGTATGAGCATTCAACAGT  439
```

Sample 14

SNP 10

Sequence ID: **Query_1119** Length: **293** Number of Matches: **1**

Range 1: 37 to 293 Graphics ▼ Next Match ▲ P

Score	Expect	Identities	Gaps	Strand
449 bits(243)	3e-131	255/260(98%)	3/260(1%)	Plus/Plus

```
Query  3    ATACAGTCTTGTCTTCTGAGTGTTACCCAGCTCAAGTATAAGTAGCCAAATTATTTTTGC  62
            ||||  |||| ||||||||||||||||||| |||||||||||||||||||||||||||||
Sbjct  37   ATACCATCTT-TCTTCTGAGTGTTACCCAG-TCAAGTATAAGTAGCCAAATTATTTTTGC  94

Query  63   ACATCTTTCTGTTTCTCATGTCTTCATTTATTCAACAAGCACTTACTGGGAAGGTCTACA  122
            ||||||||||||||||||||||||||||||||||||||||||||||||||||||||||| 
Sbjct  95   ACATCTTTCTGTTTCTCATGTCTTCATTTATTCAACAAGCACTTACTGGGAAGGTCTAC-  153

Query  123  ACCTGCATAGGCAATGCTGGAAAAAGGGTTAAGTAAACCAGGACATGACAATGGTGGCAA  182
            ||||||||||||||||||||||||||||||||||||||||||||||||||||||||||||
Sbjct  154  ACCTGCATAGGCAATGCTGGAAAAAGGGTTAAGTAAACCAGGACATGACAATGGTGGCAA  213

Query  183  ATGACTATCAGGTCTTCCCATGTGTTTGACTCAAACTTATTACCCTATGGTCCTTCTGAC  242
            ||||||||||||||||||||||||||||||||||||||||||||||||||||||||||||
Sbjct  214  ATGACTATCAGGTCTTCCCATGTGTTTGACTCAAACTTATTACCCTATGGTCCTTCTGAC  273

Query  243  AATGGCAGAAGGTCTGAATC  262
            ||||||||||||||||||||
Sbjct  274  AATGGCAGAAGGTCTGAATC  293
```

SNP 27

Sequence ID: **Query_217633** Length: **505** Number of Matches: **1**

Range 1: 35 to 505 Graphics ▼ Next Match ▲

Score	Expect	Identities	Gaps	Strand
804 bits(435)	0.0	462/473(98%)	10/473(2%)	Plus/Plus

```
Query  1    GGGGGACAAAGCAAAGAACTTTGTCTCaaaaaaaaCATGTATAGCTACATAATTAATAAT  60
            ||| |||||||||| | |||||||||||||||||||||||||||||||||||||||||||
Sbjct  35   GGGTGACAAAGCAA-G-ACTTTGTCTCAAAAAAAACATGTATAGCTACATAATTAATAAT  92

Query  61   ATGCAAATATTCTTCTTTAGGGTCACTTAGGTTTTCAACATACAGACCCAAGAGAAGGTA  120
            ||||||||||||||||||||||||||||||||||||||||||||||||||||||||||||
Sbjct  93   ATGCAAATATTCTTCTTTAGGGTCACTTAGGTTTTCAACATACAGACCCAAGAGAAGGTA  152

Query  121  CAGAGACTCATTCTCTCTCTCTTTCAAT--------cacacacacacacacacacacaca  172
            ||||||||||||||||||||||||||||        ||||||||||||||||||||||||
Sbjct  153  CAGAGACTCATTCTCTCTCTCTTTCAATCACACACACACACACACACACACACACACACA  212

Query  173  cacacacacacCCCTCTTCACTATAATTATAATTACTATGTTGGCTTCCAGATCAGGGGT  232
            ||||||||||||||||||||||||||||||||||||||||||||||||||||||||||||
Sbjct  213  CACACACACACCCCTCTTCACTATAATTATAATTACTATGTTGGCTTCCAGATCAGGGGT  272

Query  233  TAGAGCCTTGGCATGGAGACGCCTGAAAGGCACCCAAGGCAATTAGTGGTGTCCCTTCTC  292
            ||||||||||||||||||||||||||||||||||||||||||||||||||||||||||||
Sbjct  273  TAGAGCCTTGGCATGGAGACGCCTGAAAGGCACCCAAGGCAATTAGTGGTGTCCCTTCTC  332

Query  293  CACCCCCTACATACCTTCAGGCCCTTTGCACTTGCTGCTCCCCCTCCCAGTGCACACACC  352
            ||||||||||||||||||||||||||||||||||||||||||||||||||||||||||||
Sbjct  333  CACCCCCTACATACCTTCAGGCCCTTTGCACTTGCTGCTCCCCCTCCCAGTGCACACACC  392

Query  353  CCCAGATCCTTGTGTGGCTCCCTCTCCCACCACATTTTGGTCTTAGCTCAAATGCCACCT  412
            ||||||||||||||||||||||||||||||||||||||||||||||||||||||||||||
Sbjct  393  CCCAGATCCTTGTGTGGCTCCCTCTCCCACCACATTTTGGTCTTAGCTCAAATGCCACCT  452

Query  413  CCTCAGAAATGCCTGGCCTGGCCGCCTTCAGGGCTGTCTATATGCTCACCAGC  465
            |||||||||||||||||||||||||||||||||||||||||||||||||||||
Sbjct  453  CCTCAGAAATGCCTGGCCTGGCCGCCTTCAGGGCTGTCTATATGCTCACCAGC  505
```

SNP 29

Sequence ID: **Query_14577** Length: **384** Number of Matches: **1**

Range 1: 36 to 382 Graphics ▼ Next Match ▲

Score	Expect	Identities	Gaps	Strand
632 bits(342)	0.0	347/349(99%)	2/349(0%)	Plus/Plus

```
Query  2    AAACCATGCCCATTCTGCACACGTATCCCAGAACTTAAAGTATAATTTTAAAAAAGATGT  61
            ||||| ||||||||||||||||||||||||||||||||||||||||||||||||||||||
Sbjct  36   AAACC-TGCCCATTCTGCACACGTATCCCAGAACTTAAAGTATAATTTTAAAAAAGATGT  94

Query  62   TTTATAATCTAATTGGTGACTGACAACTAAGGTACGTTAGACCATTAGCAAAAGTAAATT  121
            ||||||||||||||||||||||||||||||||||||||||||||||||||||||||||||
Sbjct  95   TTTATAATCTAATTGGTGACTGACAACTAAGGTACGTTAGACCATTAGCAAAAGTAAATT  154

Query  122  AAGTGATAAGTATTATGGTAGGGACCTCAGGTAATATAGGAATTGGGCAGAGAGAGACGT  181
            ||||||||||||||||||||||||||||||||||||||||||||||||||||||||||||
Sbjct  155  AAGTGATAAGTATTATGGTAGGGACCTCAGGTAATATAGGAATTGGGCAGAGAGAGACGT  214

Query  182  TAACATGGTTAACTCCAAAAGTGGTATGACCTAGAAAATGGGTAGATAGGTTTGAGTAAG  241
            ||||||||||||||||||||||||||||||||||||||||||||||||||||||||||||
Sbjct  215  TAACATGGTTAACTCCAAAAGTGGTATGACCTAGAAAATGGGTAGATAGGTTTGAGTAAG  274

Query  242  TGGAAGGAGAGAAAAGAAATTTCTAGGCATAGACGCAGTGTTTCTCAAGAAACTGAAAGA  301
            ||||||||||||||||||||||||||||||||||||||||||||||||||||||||||||
Sbjct  275  TGGAAGGAGAGAAAAGAAATTTCTAGGCATAGACGCAGTGTTTCTCAAGAAACTGAAAGA  334

Query  302  CAAATATGCAGGTCCTGCTTATGGAAAAGGGAAGACAGAAGGGTGTCTT  350
            |||||||||||||||||||||||||||||||||||||||||| ||||||
Sbjct  335  CAAATATGCAGGTCCTGCTTATGGAAAAGGGAAGACAGAAGG-TGTCTT  382
```

SNP 46

```
Sequence ID: Query_60859  Length: 551  Number of Matches: 1

Range 1: 37 to 543  Graphics                                        ▼ Next Match ▲

Score           Expect  Identities       Gaps         Strand
832 bits(450)   0.0     490/509(96%)     3/509(0%)    Plus/Plus

Query  4    CAATAAAAATCAAAGAAATCAAAATAAAATACCACGTTTAATTCAATTTATCACGTCCAA  63
            || |||||||| |||||||| |||||||||||||||||||||||||||||||||||||||
Sbjct  37   CATTAAAAATC-AAGAAATCCAAATAAAATACCACGTTTAATTCAATTTATCACGTCCAA  95

Query  64   GAAGTTACAAAGGCAATGTAGAAGAAGTAAGTAAAAATGGGGAAGCCCTAATTTTTAAGT  123
            ||||||||||||||||||||||||||||||||||||||||||||||||||||||||||||
Sbjct  96   GAAGTTACAAAGGCAATGTAGAAGAAGTAAGTAAAAATGGGGAAGCCCTAATTTTTAAGT  155

Query  124  CTGCCTTGAAAACAATTTTTTCTTATCAATGAGACTTTCTAATAGGACTCATCTTGCCAT  183
            ||||||||||||||||||||||||||||||||||||||||||||||||||||||||||||
Sbjct  156  CTGCCTTGAAAACAATTTTTTCTTATCAATGAGACTTTCTAATAGGACTCATCTTGCCAT  215

Query  184  TGGCACCCACAAAATCTAACCACCCACCTCCACTTCTCCGGAGAGGATCCCTTCCTAATG  243
            ||||||||||||||||||||||||||||||||||||||||| ||||||||||||||||||
Sbjct  216  TGGCACCCACAAAATCTAACCACCCACCTCCACTTCTCCGG-GAGGATCCCTTCCTAATG  274

Query  244  TCTGATGCCTCTTCCCTCACCTCCATTCTTTGCCCACTGCTGCACATGCTTAAAGCCCAG  303
            ||||||||||||||||||||||||||||||||||||||||||||||||||||||||||||
Sbjct  275  TCTGATGCCTCTTCCCTCACCTCCATTCTTTGCCCACTGCTGCACATGCTTAAAGCCCAG  334

Query  304  CCACCCTTTCACCCACAGAGGTCATAGACCACTGCTACTATGGTTAATTCTCAGAAGTAA  363
            ||||||||||||||||||||||||||||||||||||||||||||||||||||||||||||
Sbjct  335  CCACCCTTTCACCCACAGAGGTCATAGACCACTGCTACTATGGTTAATTCTCAGAAGTAA  394

Query  364  TAAACTCAGAACCTGAAAGCACATAATGCCTACAAGACTGTTCACTATTTCAAACTTCTG  423
            ||||||||||||||||||||||||||||||||||||||||||||||||||||||||||||
Sbjct  395  TAAACTCAGAACCTGAAAGCACATAATGCCTACAAGACTGTTCACTATTTCAAACTTCTG  454

Query  424  CTCTGGTTTACATGAATTaaaaaaaaaaaaaaCCCCCtttttttttCGG-TGGACCCAGG  482
            |||||||||||||||||||||||||||||||| | |  | ||||||| | || ||| |||
Sbjct  455  CTCTGGTTTACATGAATTAAAAAAAAAAAAAATCACTCTATTTTTTCTGCTGTACCAAGG  514

Query  483  GaaaaaaaTTTCACCATCCCCTTTAAAGG  511
             |||||||||  || ||| | ||||||||
Sbjct  515  CAAAAAAATTCTACAATCACATTTAAAGG  543
```

SNP 49

```
Sequence ID: Query_28181  Length: 439  Number of Matches: 1

Range 1: 47 to 439  Graphics                                        ▼ Next Match ▲

Score           Expect  Identities       Gaps         Strand
715 bits(387)   0.0     392/394(99%)     2/394(0%)    Plus/Plus

Query  19   AGGGCTGCTTTGGTTGA-GCGAAGATGGGAACGCTTGAATGCCCTTTCGTACTACTCCCC  77
            ||||||||||||||||| ||||||||||| ||||||||||||||||||||||||||||||
Sbjct  47   AGGGCTGCTTTGGTTGAAGCGAAGATGGG-ACGCTTGAATGCCCTTTCGTACTACTCCCC  105

Query  78   TTTTACCTCGTTAATACCCACTGACCTATCCTCGTGGAATGCAGGGCTCAAAGAACAATC  137
            ||||||||||||||||||||||||||||||||||||||||||||||||||||||||||||
Sbjct  106  TTTTACCTCGTTAATACCCACTGACCTATCCTCGTGGAATGCAGGGCTCAAAGAACAATC  165

Query  138  TAAAAATCAAACATTATACAAATGCAACCTAAGGAGGAGAGTTCCTTTGAGGCCAGGGGC  197
            ||||||||||||||||||||||||||||||||||||||||||||||||||||||||||||
Sbjct  166  TAAAAATCAAACATTATACAAATGCAACCTAAGGAGGAGAGTTCCTTTGAGGCCAGGGGC  225

Query  198  TACATTATCTTATCTGTATTGCCAGCGCAGAGGCCTACTAGTACATTGTAGGGTCTAAGT  257
            ||||||||||||||||||||||||||||||||||||||||||||||||||||||||||||
Sbjct  226  TACATTATCTTATCTGTATTGCCAGCGCAGAGGCCTACTAGTACATTGTAGGGTCTAAGT  285

Query  258  ACATTTTTCCTGAATGAAAGGTATTAAATGGTAACTTACGTCTTTATGCACTCTATAAAC  317
            ||||||||||||||||||||||||||||||||||||||||||||||||||||||||||||
Sbjct  286  ACATTTTTCCTGAATGAAAGGTATTAAATGGTAACTTACGTCTTTATGCACTCTATAAAC  345

Query  318  TATGACGTGATCGTCTCCGTCTAACAACTACACTCAAATGCTTACCAAGCTCTTTAAAGG  377
            ||||||||||||||||||||||||||||||||||||||||||||||||||||||||||||
Sbjct  346  TATGACGTGATCGTCTCCGTCTAACAACTACACTCAAATGCTTACCAAGCTCTTTAAAGG  405

Query  378  GAAGAATTCCATGGTCGTATGAGCATTCAACAGT  411
            ||||||||||||||||||||||||||||||||||
Sbjct  406  GAAGAATTCCATGGTCGTATGAGCATTCAACAGT  439
```

Sample 15

SNP 10

Sequence ID: **Query_10083** Length: **293** Number of Matches: **1**

Range 1: 40 to 293 Graphics ▼ Next Match ▲ P

Score	Expect	Identities	Gaps	Strand
464 bits(251)	1e-135	254/255(99%)	1/255(0%)	Plus/Plus

```
Query  4    CCATCTTTCTTCTGAGTGTTACCCAGTCAAGTATAAGTAGCCAAATTATTTTTGCACATC  63
            ||||||||||||||||||||||||||||||||||||||||||||||||||||||||||||
Sbjct  40   CCATCTTTCTTCTGAGTGTTACCCAGTCAAGTATAAGTAGCCAAATTATTTTTGCACATC  99

Query  64   TTTCTGTTTCTCATGTCTTCATTTATTCAACAAGCACTTACTGGGAAGGTCTACAACCTG  123
            |||||||||||||||||||||||||||||||||||||||||||||||||||||| |||||
Sbjct  100  TTTCTGTTTCTCATGTCTTCATTTATTCAACAAGCACTTACTGGGAAGGTCTAC-ACCTG  158

Query  124  CATAGGCAATGCTGGAAAAAGGGTTAAGTAAACCAGGACATGACAATGGTGGCAAATGAC  183
            ||||||||||||||||||||||||||||||||||||||||||||||||||||||||||||
Sbjct  159  CATAGGCAATGCTGGAAAAAGGGTTAAGTAAACCAGGACATGACAATGGTGGCAAATGAC  218

Query  184  TATCAGGTCTTCCCATGTGTTTGACTCAAACTTATTACCCTATGGTCCTTCTGACAATGG  243
            ||||||||||||||||||||||||||||||||||||||||||||||||||||||||||||
Sbjct  219  TATCAGGTCTTCCCATGTGTTTGACTCAAACTTATTACCCTATGGTCCTTCTGACAATGG  278

Query  244  CAGAAGGTCTGAATC  258
            |||||||||||||||
Sbjct  279  CAGAAGGTCTGAATC  293
```

SNP 27

Sequence ID: **Query_220411** Length: **505** Number of Matches: **1**

Range 1: 41 to 505 Graphics ▼ Next Match ▲

Score	Expect	Identities	Gaps	Strand
789 bits(427)	0.0	454/465(98%)	10/465(2%)	Plus/Plus

```
Query  5    CAAGGC-AGACTTTGTCTC-aaaaaaaCATGTATAGCTACATAATTAATAATATGCAAAT  62
            ||| || |||||||||||| ||||||||||||||||||||||||||||||||||||||||
Sbjct  41   CAAAGCAAGACTTTGTCTCAAAAAAAACATGTATAGCTACATAATTAATAATATGCAAAT  100

Query  63   ATTCTTCTTTAGGGTCACTTAGGTTTTCAACATACAGACCCAAGAGAAGGTACAGAGACT  122
            ||||||||||||||||||||||||||||||||||||||||||||||||||||||||||||
Sbjct  101  ATTCTTCTTTAGGGTCACTTAGGTTTTCAACATACAGACCCAAGAGAAGGTACAGAGACT  160

Query  123  CATTCTCTCTCTCTTTCAAT--------cacacacacacacacacacacacacacacaca  174
            ||||||||||||||||||||        ||||||||||||||||||||||||||||||||
Sbjct  161  CATTCTCTCTCTCTTTCAATCACACACACACACACACACACACACACACACACACACACA  220

Query  175  cacCCCTCTTCACTATAATTATAATTACTATGTTGGCTTCCAGATCAGGGGTTAGAGCCT  234
            ||||||||||||||||||||||||||||||||||||||||||||||||||||||||||||
Sbjct  221  CACCCCTCTTCACTATAATTATAATTACTATGTTGGCTTCCAGATCAGGGGTTAGAGCCT  280

Query  235  TGGCATGGAGACGCCTGAAAGGCACCCAAGGCAATTAGTGGTGTCCCTTCTCCACCCCCT  294
            ||||||||||||||||||||||||||||||||||||||||||||||||||||||||||||
Sbjct  281  TGGCATGGAGACGCCTGAAAGGCACCCAAGGCAATTAGTGGTGTCCCTTCTCCACCCCCT  340

Query  295  ACATACCTTCAGGCCCTTTGCACTTGCTGCTCCCCCTCCCAGTGCACACACCCCCAGATC  354
            ||||||||||||||||||||||||||||||||||||||||||||||||||||||||||||
Sbjct  341  ACATACCTTCAGGCCCTTTGCACTTGCTGCTCCCCCTCCCAGTGCACACACCCCCAGATC  400

Query  355  CTTGTGTGGCTCCCTCTCCCACCACATTTTGGTCTTAGCTCAAATGCCACCTCCTCAGAA  414
            ||||||||||||||||||||||||||||||||||||||||||||||||||||||||||||
Sbjct  401  CTTGTGTGGCTCCCTCTCCCACCACATTTTGGTCTTAGCTCAAATGCCACCTCCTCAGAA  460

Query  415  ATGCCTGGCCTGGCCGCCTTCAGGGCTGTCTATATGCTCACCAGC  459
            |||||||||||||||||||||||||||||||||||||||||||||
Sbjct  461  ATGCCTGGCCTGGCCGCCTTCAGGGCTGTCTATATGCTCACCAGC  505
```

SNP 29

Sequence ID: **Query_57869** Length: **384** Number of Matches: **1**

Range 1: 66 to 384 Graphics ▼ Next Match ▲ P

Score	Expect	Identities	Gaps	Strand
577 bits(312)	2e-169	317/319(99%)	1/319(0%)	Plus/Plus

```
Query  4    AACTTAAAGTATAATTTTAAAAAAGATGTTTTATAATCTAATTGGTGACTGACAACTAAG  63
            ||||||||||||||||||||||||||||||||||||||||||||||||||||||||||||
Sbjct  66   AACTTAAAGTATAATTTTAAAAAAGATGTTTTATAATCTAATTGGTGACTGACAACTAAG  125

Query  64   GTACGTTAGACCATTAGCAAAAGTAAATTAAGTGATAAGTATTATGGTAGGGACCTCAGG  123
            ||||||||||||||||||||||||||||||||||||||||||||||||||||||||||||
Sbjct  126  GTACGTTAGACCATTAGCAAAAGTAAATTAAGTGATAAGTATTATGGTAGGGACCTCAGG  185

Query  124  TAATATAGGAATTGGGCAGAGAGAGACGTTAACATGGTTAACTCCAAAAGTGGTATGACC  183
            ||||||||||||||||||||||||||||||||||||||||||||||||||||||||||||
Sbjct  186  TAATATAGGAATTGGGCAGAGAGAGACGTTAACATGGTTAACTCCAAAAGTGGTATGACC  245

Query  184  TAAAAAATGGGTAGATAGGTTTGAGTAAGTGGAAGGAGAGAAAAGAAATTTCTAGGCATA  243
            || |||||||||||||||||||||||||||||||||||||||||||||||||||||||||
Sbjct  246  TAGAAAATGGGTAGATAGGTTTGAGTAAGTGGAAGGAGAGAAAAGAAATTTCTAGGCATA  305

Query  244  GACGCAGTGTTTCTCAAGAAACTGAAAGACAAATATGCAGGTCCTGCTTATGGAAAAGGG  303
            ||||||||||||||||||||||||||||||||||||||||||||||||||||||||||||
Sbjct  306  GACGCAGTGTTTCTCAAGAAACTGAAAGACAAATATGCAGGTCCTGCTTATGGAAAAGGG  365

Query  304  AAGACAGAAG-TGTCTTGC  321
            |||||||||| ||||||||
Sbjct  366  AAGACAGAAGGTGTCTTGC  384
```

SNP 46

```
Sequence ID: Query_4801  Length: 551  Number of Matches: 1

Range 1: 51 to 540 Graphics                                   ▼ Next Match ▲

Score           Expect   Identities        Gaps          Strand
817 bits(442)   0.0      475/491(97%)      2/491(0%)     Plus/Plus

Query  3    AAATCCCAAATAAAATACCACGTTTAATTCAATTTATCACGTCCAAGAAGTTACAAAGGC  62
            |||| |||||||||||||||||||||||||||||||||||||||||||||||||||||||
Sbjct  51   AAAT-CCAAATAAAATACCACGTTTAATTCAATTTATCACGTCCAAGAAGTTACAAAGGC  109

Query  63   AATGTAGAAGAAGTAAGTAAAAATGGGGAAGCCCTAATTTTTAAGTCTGCCTTGAAAACA  122
            ||||||||||||||||||||||||||||||||||||||||||||||||||||||||||||
Sbjct  110  AATGTAGAAGAAGTAAGTAAAAATGGGGAAGCCCTAATTTTTAAGTCTGCCTTGAAAACA  169

Query  123  ATTTTTTCTTATCAATGAGACTTTCTAATAGGACTCATCTTGCCATTGGCACCCACAAAA  182
            ||||||||||||||||||||||||||||||||||||||||||||||||||||||||||||
Sbjct  170  ATTTTTTCTTATCAATGAGACTTTCTAATAGGACTCATCTTGCCATTGGCACCCACAAAA  229

Query  183  TCTAACCACCCACCTCCACTTCTCCGGGAGGATCCCTTCCTAATGTCTGATGCCTCTTCC  242
            ||||||||||||||||||||||||||||||||||||||||||||||||||||||||||||
Sbjct  230  TCTAACCACCCACCTCCACTTCTCCGGGAGGATCCCTTCCTAATGTCTGATGCCTCTTCC  289

Query  243  CTCACCTCCATTCTTTGCCCACTGCTGCACATGCTTAAAGCCCAGCCACCCTTTCACCCA  302
            ||||||||||||||||||||||||||||||||||||||||||||||||||||||||||||
Sbjct  290  CTCACCTCCATTCTTTGCCCACTGCTGCACATGCTTAAAGCCCAGCCACCCTTTCACCCA  349

Query  303  CAGAGGTCATAGACCACTGCTACTATGGTTAATTCTCAGAAGTAATAAACTCAGAACCTG  362
            ||||||||||||||||||||||||||||||||||||||||||||||||||||||||||||
Sbjct  350  CAGAGGTCATAGACCACTGCTACTATGGTTAATTCTCAGAAGTAATAAACTCAGAACCTG  409

Query  363  AAAGCACATAATGCCTACAAGACTGTTCACTATTTCAAACTTCTGCTCTGGTTTACATGA  422
            ||||||||||||||||||||||||||||||||||||||||||||||||||||||||||||
Sbjct  410  AAAGCACATAATGCCTACAAGACTGTTCACTATTTCAAACTTCTGCTCTGGTTTACATGA  469

Query  423  ATTaaaaaaaaaaaaaaCCCCCCAtttttttCGG-TGGACCCAGGGaaaaaaaTTTCAAA  481
            ||||||||||||||||| | | |  ||||||| | || ||| ||| |||||||||  | |
Sbjct  470  ATTAAAAAAAAAAAAAAATCACTCTATTTTTTCTGCTGTACCAAGGCAAAAAAATTCTACA  529

Query  482  ATCCCCTTTAA  492
            ||| | |||||
Sbjct  530  ATCACATTTAA  540
```

SNP 49

```
Sequence ID: Query_5979  Length: 439  Number of Matches: 1

Range 1: 50 to 439 Graphics                                   ▼ Next Match ▲

Score           Expect   Identities        Gaps          Strand
706 bits(382)   0.0      389/392(99%)      2/392(0%)     Plus/Plus

Query  1    GCTGCTTTGGTTGAAGCGAAGATGGGACGCTTGAATGCCCTTTCGTACTACTCCCCTTTT  60
            ||||||||||||||||||||||||||||||||||||||||||||||||||||||||||||
Sbjct  50   GCTGCTTTGGTTGAAGCGAAGATGGGACGCTTGAATGCCCTTTCGTACTACTCCCCTTTT  109

Query  61   ACCTCGTTAATACCCACTGACCTATCCTCGTGGAATGCAGGGCTCAAAGAACAATCTAAA  120
            ||||||||||||||||||||||||||||||||||||||||||||||||||||||||||||
Sbjct  110  ACCTCGTTAATACCCACTGACCTATCCTCGTGGAATGCAGGGCTCAAAGAACAATCTAAA  169

Query  121  AATCAAACATTATACAAATGCAACCTAAGGAGGAGAGTTCCTTTGAGGCCAGGGAGCTAC  180
            |||||||||||||||||||||||||||||||||||||||||||||||||||||| |||||
Sbjct  170  AATCAAACATTATACAAATGCAACCTAAGGAGGAGAGTTCCTTTGAGGCCAGGG-GCTAC  228

Query  181  ATTATCTTATCTGTATTGCCAGCGCAGAGGCCTACTAGTACATTGTAGGGTCTAAGTACA  240
            ||||||||||||||||||||||||||||||||||||||||||||||||||||||||||||
Sbjct  229  ATTATCTTATCTGTATTGCCAGCGCAGAGGCCTACTAGTACATTGTAGGGTCTAAGTACA  288

Query  241  TTTTTCCTGAATGAAAGGTATTAAATGGTAACTTACGTCTTTATGCACTCTATAAACTAT  300
            ||||||||||||||||||||||||||||||||||||||||||||||||||||||||||||
Sbjct  289  TTTTTCCTGAATGAAAGGTATTAAATGGTAACTTACGTCTTTATGCACTCTATAAACTAT  348

Query  301  GACGTGATCGTCTCCGTCTAACAACTACACTCAAATGCTTACCAAGCTCTTTAAAGGGAA  360
            ||||||||||||||||||||||||||||||||||||||||||||||||||||||||||||
Sbjct  349  GACGTGATCGTCTCCGTCTAACAACTACACTCAAATGCTTACCAAGCTCTTTAAAGGGAA  408

Query  361  GAATTCCATGGTCGTATGAGCTTTTCAACAGT  392
            ||||||||||||||||||||| || |||||||
Sbjct  409  GAATTCCATGGTCGTATGAGCATT-CAACAGT  439
```

Figure S13. Alignment of the obtained sequences by Sanger sequencing compared with their synthetic sequence DNA using *Blastn* software.

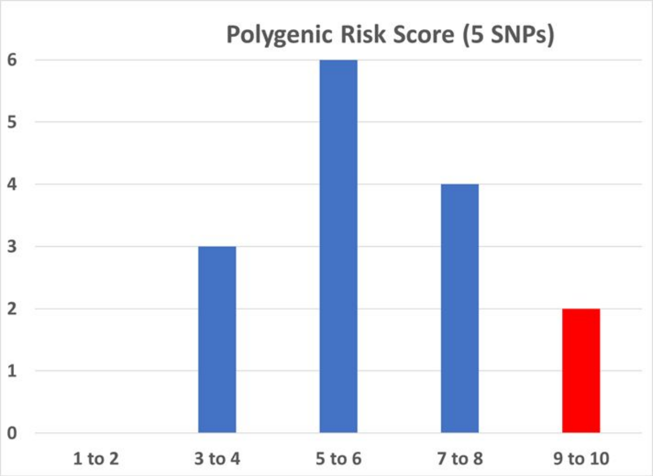

Figure S14: Plot representing data detailed in Table 3. Cases 9, 10 represent cases with a high risk of developing osteoporosis.

4. References

(1) Brázdilová, P.; Vrábel, M.; Pohl, R.; Pivoňková, H.; Havran, L.; Hocek, M.; Fojta, M. Ferrocenylethynyl Derivatives of Nucleoside Triphosphates: Synthesis, Incorporation, Electrochemistry, and Bioanalytical Applications. *Chemistry – A European Journal* **2007**, *13* (34), 9527–9533.

(2) Ménová, P.; Raindlová, V.; Hocek, M. Scope and Limitations of the Nicking Enzyme Amplification Reaction for the Synthesis of Base-Modified Oligonucleotides and Primers for PCR. *Bioconjug Chem* **2013**, *24* (6), 1081–1093.

(3) Simonova, A.; Magriñá, I.; Sýkorová, V.; Pohl, R.; Ortiz, M.; Havran, L.; Fojta, M.; O'Sullivan, C. K.; Hocek, M. Tuning of Oxidation Potential of Ferrocene for Ratiometric Redox Labeling and Coding of Nucleotides and DNA. *Chemistry* **2020**, *26* (6), 1286–1291.

(4) Ortiz, M.; Jauset-Rubio, M.; Kodr, D.; Simonova, A.; Hocek, M.; O'Sullivan, C. K. Solid-Phase Recombinase Polymerase Amplification Using Ferrocene-Labelled dNTPs for Electrochemical Detection of Single Nucleotide Polymorphisms. *Biosens Bioelectron* **2022**, *198*, 113825.

(5) Jauset-Rubio, M.; Ortiz, M.; O'Sullivan, C. K. Solid-Phase Primer Elongation Using Biotinylated dNTPs for the Detection of a Single Nucleotide Polymorphism from a Fingerprick Blood Sample. *Anal Chem* **2021**, *93* (44), 14578–14585.
